# Dioxin in the Elbe river basin: policy and science under the water framework directive 2000–2015 and toward 2021

**DOI:** 10.1186/s12302-016-0075-8

**Published:** 2016-03-29

**Authors:** Ulrich Förstner, Henner Hollert, Markus Brinkmann, Kathrin Eichbaum, Roland Weber, Wim Salomons

**Affiliations:** 1 Institute of Environmental Technology and Energy Economics, University of Technology Hamburg-Harburg, Eissendorfer Street, 21071 Hamburg, Germany; 2 Department of Ecosystem Analysis, Institute for Environmental Research, ABBt – Aachen Biology and Biotechnology, RWTH Aachen University, Worringerweg 1, 52074 Aachen, Germany; 3 POPs Environmental Consulting, Lindenfirststrasse 23, 73527 Schwäbisch Gmünd, Germany; 4 Kromme Elleboog 21, 9751 RB, Haren, Groningen Netherlands

**Keywords:** Activated carbon, Biota-EQS, Bitterfeld region, Chemical status, Dredged materials, Flood risks, Marine strategy, NGOs, RBC Elbe, Sediment management concept

## Abstract

A critical review of the last 25 years of dioxin policy in the Elbe river catchment is presented along seven main theses of the River Basin Community (RBC)-Elbe background document “Pollutants” for the Management Plan 2016–2021. In this period, polychlorinated dibenzodioxins/-furans (PCDD/Fs) and dioxin-like polychlorinated biphenyls (dl-PCBs) will play a major role: (i) as new priority substances for which environmental quality standards (EQSs) need to be derived (Directive 2013/39/EC); (ii) in the search for innovative solutions in sediment remediation (i.e., respecting the influence of mechanical processes; Flood Risk Directive 2007/60/EC); and (iii) as indicators at the land–sea interface (Marine Strategy Framework Directive 2008/56/EC). In the Elbe river catchment, aspects of policy and science are closely connected, which became particularly obvious in a classic example of dioxin hot spot contamination, the case of the Spittelwasser creek. Here, the “source-first principle” of the first cycle of the European Water Framework Directive (WFD) had to be confirmed in a controversy on the dioxin hot spots with Saxony-Anhalt’s Agency for Contaminated Sites (LAF). At the Spittelwasser site, the move from “inside the creek” to “along the river banks” goes parallel to a general paradigm shift in retrospective risk assessment frameworks and remediation techniques for organic chemicals (Ortega-Calvo et al. 2015). With respect to dioxin, large-scale stabilization applying activated carbon additions is particularly promising. Another important aspect is the assessment of the ecotoxicology of dioxins and dl- PCBs in context of sediment mobility and flood risk assessment, which has been studied in the project framework FloodSearch. Currently, the quality goals of the WFD to reach a “good chemical status” are not met in many catchment areas because substances such as mercury do and others probably will (PCDD/Fs and dl-PCB) exceed biota-EQS values catchment area-wide. So far, relating biota-EQS values to sediment-EQSs is not possible. To overcome these limitations, the DioRAMA project was initiated, which has led to improved approaches for the assessment of dioxin-contaminated sediment using in vitro bioassays and to a robust dataset on the interrelation between dioxins and dioxin-like compounds in sediments and biota.

**Electronic supplementary material:**

The online version of this article (doi:10.1186/s12302-016-0075-8) contains supplementary material, which is available to authorized users.

## Background

Two prominent objectives of the European Water Framework Directive (WFD 2000 [1]) are the catchment-wide no-deterioration status (article 4) and the reduction of priority pollutants [2]. While the former WFD-principle just entered the public discussion via a ruling of the European Court of Justice from July 1, 2015 on a river deepening project [3], the Actualized River Basin Management Plan of the River Basin Community Elbe from December 22, 2015 [4] states: “without goal-oriented measures for the reduction of primary and secondary pollution sources, the objectives of a good chemical and ecological quality in surface waters until the end of the second WFD management period and a good environmental state according to the European Marine Strategy Framework Directive (MSFD [5]) until 2020 will be strongly endangered.” Both examples demonstrate the need for comprehensive and in-depth information from policy and river basin administration with regard to WFD key issues.

The same is valid for an involvement of scientific expertise at critical steps in the WFD implementation process. Here, the River Basin Community Elbe has developed a good understanding of “historical contaminated sediments” [6] and a Sediment Management Concept [7]. On the other hand, the Elbe-typical dioxin problems (mainly originating from federal state area Saxony-Anhalt) were widely ignored by the River Basin Community Elbe, including the representatives from the Free and Hanseatic City of Hamburg [8]. This happened despite the perspective that in the WFD period 2016–2021, PCDD/Fs and dl-PCBs will play a major role: (a) as new priority substances in the list of biota-Environmental Quality Standards (EQS, i.e., effect-based and legally enforcible numerical quality criteria for assessing the chemical status of aquatic systems) and (b) as the most critical substances at the land–sea interface (i.e., between WFD and MSFD) [9].

Our contribution to the discussion regarding the first WFD period at the Elbe River will comprise different scientific aspects, by presenting more detailed biological and toxicological implications of dioxins and a review of new concepts for remediation on dioxin-contaminated sediments. Last, the study should reflect the achievements of consultants and experts in ad hoc groups, mostly in German language, as an invitation to the international scientific community to participate in the next cycles of the Elbe River Basin Management Plan.

### Thematic overview along the RBC Elbe background document “pollutants” (2015 [10])

Analyzing the policy of RBC Elbe in the field *“reduction of pollutant loads”* the preferred way will be along the themes of two *background documents* from the ad hoc working group “Pollutants” for the River Basin Management Plan Elbe (German Part, November 11, 2009 [11]) and the Actualized River Basin Management Plan Elbe 2016–2021(December 22, 2015 [4]). This approach includes three *WFD*-*related EU*-*directives* on marine strategies [5], “new” priority hazardous substances [12], and flood risks [13] (Table 1, last column).

**Table 1:** Original statements (in *italics*) in the seven chapters of the RBC Elbe background document “pollutants” [10] and further information in the present work (citations in square brackets refer to this work; last column: reference to WFD-near EU-directives, treated in this work, and key sections of this work)

Background document “reduction of pollutant loads” (RBC Elbe 21.12.2015 [10])	This work
1. Introduction, page 5	
*The document actualizes the contents of the background paper for the Deduction of Supra*-*Regional Management Objectives in the German Part of the Elbe River Basin for the Contaminant Focus” (RBC Elbe 2009a [6])*	Introduction: Dioxin from Bitterfeld (Box 1)
2. Supra-regional objectives, p 6–7	
*“To attain the objectives according to the EG*-*WFD (2000/60/EG) [4] and EG Marine Strategy Directive 2008/56/EG [5, 13] direct source*-*related or at least near*-*source measures are needed in many water bodies of the Elbe catchment”*	2008/56/EG MSFD Land-sea interface; Conclusions/outlook
3. Evaluation—chemical status p 8–15	
*As a result of an actualization of the assessment from 2013 it has been found that a good chemical status of the Elbe River cannot be met area*-*wide due to an excess of EQS of mercury (Hg) in biota” *(ca*. 60* *% from re*-*emissions*—*soils, sediment,* etc).	2013/39/EU new PHS EQS for dioxins/DLSs (RWTH Aachen et al.)
4. Catchment areas, sources, p 16–20	
*“In the Mulde catchment, the organic pollutants HCH and PCDD/Fs are on the top of the agenda. The middle Elbe is a relevant interim reservoir; its stagnant areas (cut*-*off meanders, harbors, groin fields) can easily be transferred during floodwater events”*	Prioritization (Box 3) Elbe R. basin sediment remediation (Table 4)
5. Hitherto activities, p 21–22	
*“The Sediment Management Concept of the RBC Elbe [7] should contribute to attain a good chemical and ecological status; as such it is a basis for the second RBMP Elbe according to WFD and for the achievement of the environmental objectives of MSRL”*	Dioxin stabilization with activated carbon; passive sampling (Box 4)
6. State of implementation, success, p 23–25	
*“Investigation on organic pollutants in suspended matter, sediments and floodplains of the Spittelwasser and the Lower Mulde River shows, that the massive fine sediment depots in the Spittelwasser do no more exist today”*	Radiometric mapping (Box 2 LAF vs Tauw)
7. Challenges, p 26–27	
The biggest challenges exist with the *very rare flood events* from August 2002 (HHQ^a^: upper Elbe R.), March/April 2006, January 2011 and June 2013 (HHQ: lower Elbe R.). “*Without a targeted stabilization or removal of highly contaminated historical deposits the flood*-*induced pollutant releases would remain a significant handicap in attaining the objectives of a good chemical (and biological) quality according to WFD”*	2007/60/EC Flood risks, Climate change (Box 5); FloodSearch, DioRAMA (RWTH Aachen et al.)

^a^HHQ: highest observed water level

#### (1) Introduction

Based on the risk study initially commissioned by the Hamburg Port Authority (HPA [14]), the RBC Elbe presented a first background paper on the aspect chemical contamination (RBC Elbe [6]) for the public discussion of the draft RBMP Elbe, German part (RBC Elbe 2009b [11]). A special conceptual achievement of the RBCs Pollutant Working Group was the early setting of priorities for remedial measures; here, the reduction needs for sediment-bound pollutants were calculated from mass loads in different areas of concern within a river basin (see RBMP Elbe 2009 [11] section 5.1).

#### (2) Supra-regional objectives

In a controversy between scientists (primarily authors of the HPA-study [14]) and a part of the RBC Elbe adminstration on the relation between pollution sources and river basin wide problem solutions (Box 3 “two versions”) the publication of the Sediment Management Concept [7] eventually decided for the preference of the “source-first principle” (“Sediment management concept: prioritization of measures—Box 3: two versions” section). A still open field is the pollutant transfer from the entire Elbe catchment which induces considerable risks for the marine environment and serious restrictions for the handling of sediment in the tidal areas (MSFD [5]). At the end of the present review, the largest deficiencies are stated, which can be attributed to the preoccupation of the responsible RBC Elbe partners related to the dioxin issue ([8]; Conclusions and Outlook, this work).

#### (3) Evaluation—chemical status

Among the eight newly identified priority substances of Directive 2013/39/EU from August 12, 2013 [12] are PCDD/Fs and dl- PCBs; The modified EQS for the existing list have to be applied beginning on December 22, 2015, and come into force on December 22, 2018 for the new substances [12]. The provisions of the Directive 2013/39/EU had to be transferred into national legislation—German Surface Water Ordinance (OGewV)—by September 14, 2015 [15]. “Directive 2013/39/EC—policy and science for biota-EQS of DLCs” section will deal with EQS for dioxin-like compounds (DLCs) and cell-based bioassays for detection of DLCs [16].

#### (4) Catchment areas, sources

When preparing remedial measures in the Elbe river basin, the authors of the Sediment Management Concept [7] have focused on the inventory of sediment volumes and their erodibility under different depositional conditions. In a special action within the Actualized RBMP Elbe [4] and related to the Sediment Management Concept of the RBC Elbe [7] the Federal Institute for Hydrology [17], among other studies, conducted a scientific survey on the groin fields along the Elbe River; one of the results was, that the majority of potentially remobilizable pollutant-rich fine-grained sediments occurs in the groin fields located downstream from Elbe-km 350 (“Sediment management concept: prioritization of measures—Box 3: two versions” section in this work).

#### (5) Hitherto activities

There are still very few actions which can be classified as “measures to reduce the pollution loads” in the closer sense; at best these activities could be described as “establishment of priority measures within an intensive analytical process” ([10] page 21). The proposals in “Dioxin stabilization using activated carbon technologies—Box 4: passive sampling” of this work follow Ortega-Calvo et al. [18], when introducing bioavailability-based concepts at the transition from excavation procedures to regulations of organic chemicals. However, the pretention of the RBC, that the Sediment Management Concept [7] would be a “basis for the achievement of the environmental objectives of the Marine Strategy Framework Directive” ([10] page 22, footnote 3), is not supported by practical activities at the land-sea interface (“Marine strategy framework directive 2008/56/EC—sediments and pollutants” section in this work).

#### (6) State of implementation, success

**Fig. 1:**
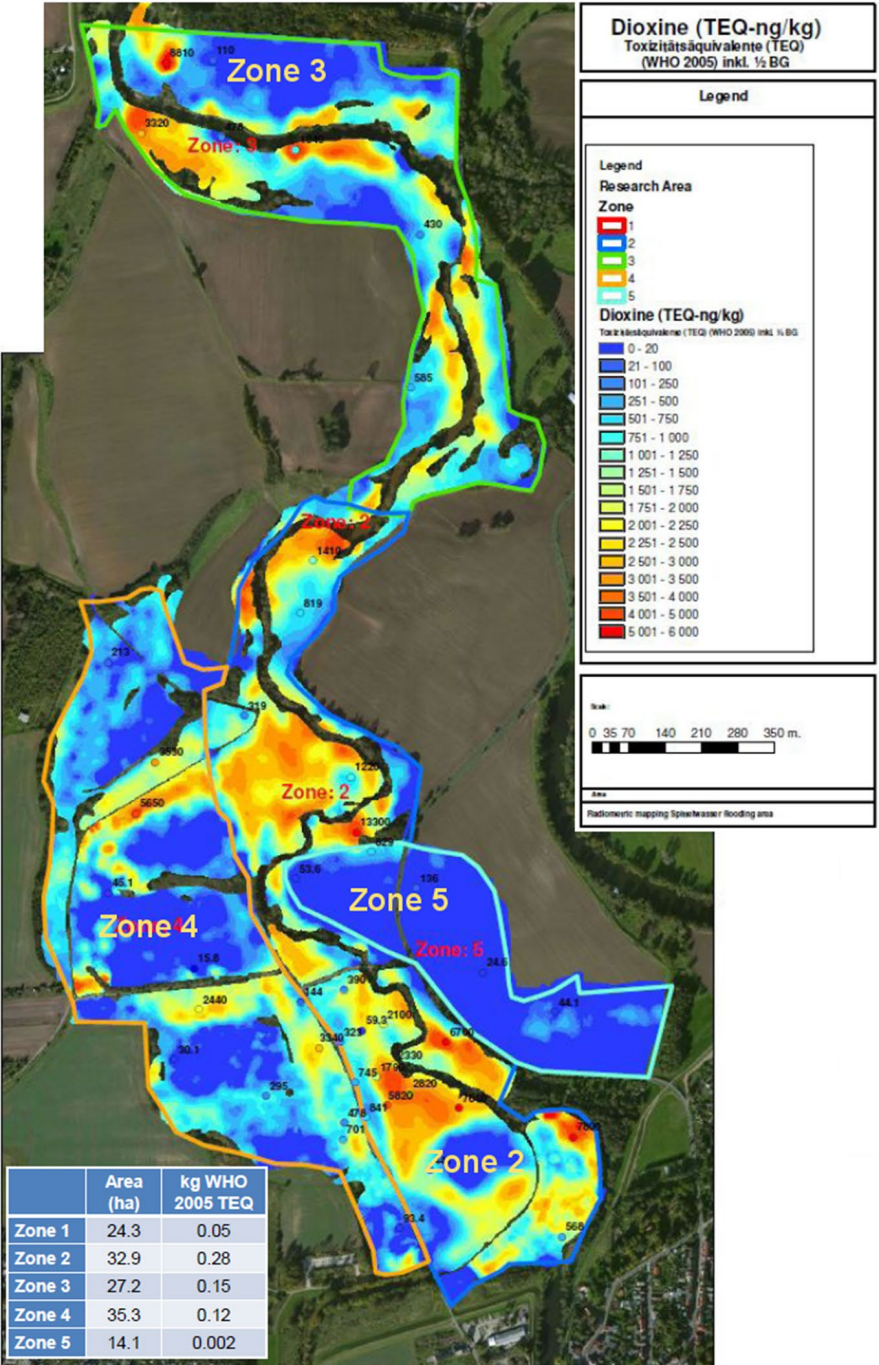
Radiometric map combining the concentration data [the last seven intervals—*yellow* to *red*—correspond to 2000–6000 ng/kg TEQ] in their areal distribution in Spittelwasser creek bank sediments or fluvisols (Tauw Soil Newsletter from July 2014 [20]) and the information on areal dioxin loads within four different zones of the Spittelwasser site (small *table left below* shows size of the zones [in hectares] and the respective loads [in kg TEQ WHO-2005]; Jacobs et al. 2013 [19]). The map of Jacobs [20] was slightly edited (legend, size)

The controversial interpretation of the “sediment depots” in the Spittelwasser creek (Fig. 1 in “Radiometric mapping along the Spittelwasser creek—Box 2: Tauw vs. LAF” section) also reflects the different views of science and local administration on the results of consultancy (Box 2 “LAF vs. Tauw [19, 20]”). Sediment cleanup increasingly moves to in situ technologies such as sediment capping, a form of in situ containment, and monitored natural recovery (MNR). With regard to the stabilization of PCDD/Fs, PCBs and other mainly sediment-bound pollutants, sorbent materials such as activated carbon progressed into a proven, reliable technology [21] (“Dioxin stabilization using activated carbon technologies—Box 4: passive sampling” section); a similar development actually happens with passive sampling as a respective assessment method ([22], Box 4). In both fields RBC Elbe has not presented own initiatives, neither in the background document [10] nor in the RBMP [4].

#### (7) Challenges

A complex legal approach in support of the chemical quality under the WFD is an assessment and management of flood risks, following Directive 2007/60/EC [13]; the special role of sediment-bound contaminants is due to the exponential increase of solid/pollutant loads with higher water flow velocities [23]. A study in cooperation with RBC Elbe “The flood-water induced remobilization of historical contaminated sediments” (BfG 2014 [17]) calculated the budgets of flood load in the Saale river, which considered both sediment inputs/outputs and the variations of the interim reservoirs. An overview on ecotoxicology in context of sediment mobility and flood risk assessment is presented in “Directive 2007/60/EC—policy and science with special reference to dioxin-like compounds and flood risks” section [24].

## Background: PCDD/Fs and dl-PCBs in sediments

Many of the current problems regarding hydrophobic substances such as polychlorinated dibenzo-*p*-dioxins and dibenzofurans (PCDD/Fs; often collectively referred to as ‘dioxins’) and polychlorinated biphenyls (PCBs) under the EU Water Framework relate to sediments mostly originating from historical release and contamination (Nizzetto et al. [25]). PCDD/Fs, PCBs, and other mainly sediment-bound pollutants are migrating together with the sediments downstream and may overtime dominate the pollution load in downstream reaches (Verta et al. [26]). The impacts of sediment-bound pollutants are particularly aggravated during storm events, when these deposits may be mobilized (Wölz et al. [27]; Weber et al. [28]). In addition to increased exposure of aquatic organisms, such as fishes, these sediments can also be transferred to floodplains where they can contaminate the food chain via grazing cattle (Lake et al. [29]; Lake et al. [30], Schulz et al. [31], Kamphues et al. [32]), Weber et al. 2015 [33].

A classic example is the dioxin hot spot in the Spittelwasser creek of the Bitterfeld Chemical Triangle, Germany, the contaminants of which could be traced in sediment samples downstream the Elbe river as far as to the Hamburg harbor area via its congeneric pattern of PCDD and PCDF (Götz et al. [34]).

### Sources of dioxins in the Elbe catchment area—Box 1: dioxin from Bitterfeld

The extreme contamination in the Elbe catchment area is largely a legacy from elemental chlorine-based metallurgical production at Bitterfeld and two other sites culminating between 1940 and 1945 [19]. The electrochemical industry in this area was closely connected with aircraft construction, which was based on hydrogen gas as a side product during chlorine alkali electrolysis (Harbodt [41]). In the early 1930s, the booming industrial branch was supplied by the light metal plants at Bitterfeld (Mulde), Aken (Elbe), and Stassfurt (Bode).

In the production process the raw materials, such as magnesium oxide and others, at first reacted with chlorine gas in a conversion furnace; in a second step, the magnesium chloride was transferred to magnesium in a melting electrolytic reaction [19]. The dioxin emissions of such facilities can be extremely high and the only semi-quantified historic emission of a magnesium production to a Norwegian fjord was estimated to 50 to more than 100 kg TEQ (Knutzen and Oehme [42]). According to the Dioxin Toolkit issued by the UNEP (2005), for one ton of magnesium produced using the fused salt electrolysis process, 9 mg I-TEQ are released into the environment via waste water; this represents an estimated 3 kg I-TEQ for the duration of the Second World War at the Bitterfeld site (Umlauf et al. [43]) as a minimum release estimate considering that technology has improved over the 60 years.

Based upon characteristic PCDD/F congeners [34, 43], it was at first detected that the extreme contamination of soils and sediments in the 60 km^2^ Mulde River and Spittelwasser floodplain (Wilken et al. [44]) was due to emissions from the Bitterfeld plants. More recent analyses by Umlauf et al. [43] suggest, that the sediment samples from the Bode und Saale can also be allocated to the Bitterfeld-Wolfen-Cluster (thermal magnesium and copper production). Whereas in the Saale catchment considerably lower concentrations of PCDD/Fs are found, the much higher suspended load (factor ten, on average) would probably compensate for this difference compared to the Mulde river (Götz et al. [45]).

The dioxin ‘hot spot’ sediments in the Spittelwasser creek came under special focus during public discussions. Peak concentrations of 140.000 I-TEQ ng/kg PCDD/Fs were measured in a 800-m-long calm water section with a sediment thickness of up to 2 m [46, 47] and approx. 20,000 m^3^ fine-grained sediment was estimated in the 1990s (1995 [45], 1997 [46] and 2000 [38]). A rough estimate suggested that just one of these ponds containing 5000 m^3^ with an average of 20,000 ng TEQ per kg sediment could pollute 5 million m^3^ of Elbe sediment to 20 ng TEQ/kg (“safe sediment value” [48]); taken the 1990s estimate of 20,000 m^3^ hot spot deposits with an average of 20,000 ng TEQ per kg sediment one would calculate a total of 0.4 kg TEQ Dioxin in the ponds of the Spittelwasser creek [14].

It was argued that the Spittelwasser acts as a flood channel of the lower Mulde River when the water discharges in this Elbe tributary exceed a 5-year recurrence flood intensity (HQ_5_) (Lindemann [38]). Later, in the SARISK Project (Büttner et al. [49]) the flow trajectories of the spring flood of 2006 were simulated and it was demonstrated that the remobilization of Spittelwasser sediment will start when the Mulde is spreading over the Radegaster Forst at water discharges exceeding 200 m^3^/s.

Well then, let mankind wage a slow,

sophisticated war of destruction against this sort of nature.

With sneaking poisons we must try to destroy it.

*Novalis, The Novices of Sais*

### Box 1: Dioxin from Bitterfeld—a common heritage

The Novalis epigram appeared in a publication of Rainer Götz, dioxin expert at the State Environment Agency of Hamburg, who could—in autumn 1989—just speculate on the origin of his PCDD/F findings in the port of Hamburg: probably waste waters from the nine big pulp and paper plants of the German Democratic Republic (GDR) [35]. One year later, on the day of the German reunification, October 3 of 1990, the magazine DER SPIEGEL titled on the situation in Bitterfeld [36]: *“This means revolt; the dioxin values are higher than formerly in Seveso; people apparently do not want to understand the facts.”* A first—and so far last!—remediation project against Bitterfeld-sourced dioxin was the collection of so-called “glibberpearls,” residues from the production of ion exchangers at the “Chemie AG” [37]; the action of 1994, which was financially supported by the Free and Hanseatic State of Hamburg, provided measurable improvement for the downstream areas of the Elbe river [38].

The findings of the true sources of the large-scale dioxin contamination in the Elbe catchment had no effect on decisions to solve the problem: The feasibility study from 1993 for the sanitation of the Spittelwasser sediments, demanded of the district office Bitterfeld [39], is still kept secret. The dioxin cases of Saxony-Anhalt were obviously not on the shortlist of the sanitation program of the Treuhandanstalt (“Trust agency”); in this action, primarily established to privatize East German enterprises, the remediation budget was cut-down from 100 billion German Mark (~50 billion EURO) to less than 10 % (~4 billion EURO) at the end of the Treuhand-activities [40]. This experience could explain the different “spirit” in Saxony-Anhalt’s administration when putting into practice the WFD in the Elbe River Basin community after 2001 [8].

### Spittelwasser remediation project (feasibility study 1993)

The sensitive flood situation of the 160 km^2^ lowland area around Bitterfeld and the particular risks from mobile dioxin-rich deposits in the Spittelwasser creek urgently called for immediate remediation measures. Prominent companies participated in the feasibility study 1993 of the District Office of Bitterfeld [39] and after an evaluation of the technical aspects the consultants presented the following proposal for the remediation of the Spittelwasser dioxin hot spots [39]:

“According to available estimation (see above) ~20,000 m^3^ of sludge with a mean dry substance content of 17 % are deposited in the 3 km long river section under consideration. Based on the properties of the sediments, which—apart from the very high water content—in most cases are characterized by high percentages of fine-grained materials and organic contents, a combination of all reasonable procedures leads to 24 different remediation concepts. For the 12 variants of a shortlist expenses for full solutions can be predicted in the range of 20–30 million German Mark (DM; ~10–15 million EURO). From an ecological, technological and economic view on these variants the consultants prefer the dry recovery and wet separation of the sediments in a sand/gravel and a fine-grain fraction (grain diameter <0.06 mm) with subsequent washing of sand and thermal treatment of the fine fraction (Variant V-6 WV). Expected costs of this variant were 20.9 million DM (~10.5 million EURO).”

The feasibility study from 1993 [39] is still relevant for similar sites of mobile historical contaminated sediments. With the more recent technical developments, e.g., for new hydraulic devices, the loss of contaminated sediments during extraction could be minimized (“Sediment management concept: prioritization of measures—Box 3: two versions” section).

### Dioxin concentrations in the Spittelwasser-Mulde-Elbe system 2006/2007

It seems that the systematic sediment survey at the Spittelwasser site has not continued after the year 2000 under Saxony-Anhalt’s new Agency for Contaminated Sites (LAF; [8]). Instead, the State Agency for Flood Protection and Water Management of Saxony-Anhalt (LHW [50]) took over the responsibility for the study of dioxin in the waters and soils of the Bitterfeld area as well as in the subsequent Elbe ecosystems originating from secondary sources in the Mulde and Saale catchments. Tables 2, 3 present data on suspended sediments from the official Dioxin Report of the LHW for the Spittelwasser-Mulde-Elbe [50]. Table 2 illustrates, that via the discharge of the Spittelwasser Creek into the Mulde (dioxin level of 750 to approx. 1000 ng I-TEQ/kg in the sediments), a significant increase from 12 ng I-TEQ/kg to 92 ng I-TEQ/kg occurs at the station Mulde/Dessau compared with the upstream Mulde station Bad Düben. A comparison of the data from the Elbe at the stations Dommitzsch and Magdeburg (Table 3) indicates that, due to the influence of the dioxin-rich confluents of Saale and Mulde, there is an increase of the dioxin concentrations in the Elbe River by a factor 7.6 in the year 2006 and by a factor 6.8 in the year 2007 [50].

**Table 2:** Σ PCDD/F in I-TEQ ng/kg in suspended matter 2006/2007 [50]. Stations Mulde (Bad Düben, Dessau) monthly mixed samples at the automated measurement stations

	Mulde (Bad Düben)		Spittelwasser		Mulde (Dessau)	
Year	ø	Min/max	ø	Min/max	ø	Min/max
2006	11	10/12	741	445/1052	56.1	16.3/81.8
2007	12	11/13	1032	583/1369	127	96.4/167

Spittelwasser: downstream from Schachtgraben: suspended matter box. Individual samples for mean values 2006/2007: Bad Düben: 2/2; Spittelwasser: 6/3; Dessau: 6/4

**Table 3:** Samples from automated measurement stations (number of datasets in italics) [50]

	2006	2007
**Elbe** Dommitzsch	11.9 *(2)*	8.0 *(1)*
**Mulde** Dessau	56.1 *(4)*	126.6 *(4)*
**Saale** Gr Rosenburg	60 *(4)*	94.4 *(4)*
**Elbe** Magdeburg	91 *(3)*	54.5 *(3)*

The State Agency for Flood Protection and Water Management of Saxony-Anhalt later became involved in the studies on dioxin-polluted sediments in the Saale and Bode rivers [51].

### Radiometric mapping along the Spittelwasser creek—Box 2: Tauw vs. LAF

Originally planned as a “hydraulic system analysis of the Bitterfeld area” under the contentious dialog of Saxony-Anhalt’s new Agency for Contaminated Sites ([52]; Box 2) it became the most interesting side product of the Tauw-Study “Spittelwasser Pollutant Load Reduction” [19, 20]: The radiometric mapping of contaminants (Sn, HCHs, DDX, and PCDD/PCDF) in the Spittelwasser floodplain, which was performed in collaboration with MEDUSA bv (Groningen, The Netherlands) from 2011 to 2013. The procedure has been successfully used over more than ten years to transform areal data of the natural gamma radiation into maps of sediment and soil structures or—as in the present case—maps of contamination levels (e.g., Van der Graaf et al. [53]).

The transformation step uses the correlation between the pollutant parameter and the respective radionuclide concentration; in the Spittelwasser case, the correlation equation for dioxin was determined from a pilot study as PCDD/Fs (ng TEQ/g) = 2.38 − 0.016*K + 0.103*U with *R*^*2*^ of 0.66 and *p* value of 0.0028 [17]. In Fig. 1, which combines the information from two sources (Jacobs et al. 2013 [19] and Jacobs 2014 [20]), the concentration data for PCDD/Fs (in ng TEQ/kg) were shown in 18 intervals from less than 20 ng TEQ/kg (this is the upper threshold value for PCDD/Fs of the RBC Elbe [4, 10]) up to 6000 ng TEQ/kg (yellow to red colors >2000 ng TEQ/kg). A first conclusion from the radioactive mapping activity of Tauw [19, 20] should have been that with a more than 100-fold exceedance of the upper threshold value for dioxin at 20 ng TEQ/kg, a sufficient initial suspicion was given for undertaking further actions. Instead, the River Basin Community Elbe declared the problem as being solved (Box 2).

In their expertise, Jacobs et al. [19] calculated an integrated mass load (to 0.3 m sediment depth) of 0.61 kg TEQ for the central Spittelwasser area between Jeßnitz (south-east) and Raguhn (north); this total load is 50 % higher than the estimated load from the former hot spots in the Spittelwasser ponds (last paragraph in “Sources of dioxins in the Elbe catchment area—Box 1: dioxin from Bitterfeld” section). With these data—even as very rough estimates—there are good reasons to consider more detailed studies both for the assessment of priority areas and the selection of appropriate remedial measures (see “Dioxin stabilization using activated carbon technologies—Box 4: passive sampling” section). With regard to areal dioxin load Zone 2 (Fig. 1 [20]), which forms the near-range left and right along the Spittelwasser course, would become the first priority for actions. According to Jacobs et al. [19] Zone 2 was preferably overflown with polluted waters from the formerly highly contaminated Spittelwasser; at regressing floodwater sedimentation of highly contaminated material mainly took place in these low-leveled areas.

### Box 2: Consultancy between science and local administration—Tauw vs. LAF

At the end of the first WFD cycle, the Background Document “Pollutants” of the Elbe RBMP [10] called it a “success,” when some information from the Tauw Report [19] suggested that the summer flood of 2002 could have eroded the most critical dioxin hot spots from the Spittelwasser river bed. Until now, against better judgement, the picture of a self-cleaning river system remained untouched within the River Basin Community Elbe administration.

The Tauw Report [19] is the outcome of a “contentious dialog,” officially installed by Saxony-Anhalt in 2009 [52] after controversial discussions on the “Risk Study” [14] and the source-first principle (Box 3 “two versions”). The report to Saxony-Anhalt’s Agency for Contaminated Sites (LAF) dates from October 21, 2013; *in the public online version of LAF from July 2014[*19*] the all*-*decisive dioxin map*—*previously presented by Tauw’s Patrick Jacobs at a RBC Elbe Workshop on December 17, 2013*—*was missing:* Did the consultants capitulate in the face of the powerful client and its no-action policy [8]?

*Tauw* has tried to defend its standards at three occasions: (1) A hint to a promising, yet unpublished neighbor study, (2) a cryptic announcement: “an examination of other measures beyond that will be recommended by the authors,” and (3) a later publication of the dioxin map (Fig. 1) in the Tauw newsletter of July 2014 (Jacobs [20]), subsequent to the online release of the final report [19].

## Sediment concepts in RBMPs

Since the year 2000, any risk assessment in European waters is made by the holistic river basin approach of the Water Framework Directive (WFD) of the European Union [1, 4]. The chemical status of water bodies is to be assessed in terms of compliance with the quality standards (QSs) and under other relevant Community legislation setting environmental quality standards (EQSs) [5]. Initial steps for measures under the WFD were: 2005 first pressure and impact analysis (Article 5), 2006 monitoring programmes to be operational (Article 8) and 2009 establishment of the programme of measures (Article 11).

### The development of a WFD concept for historically contaminated sediments

The Scientific Committee on Toxicity, Ecotoxicity and the Environment (CSTEE [54]) concluded from its report “The setting of environmental quality standards for the priority substances included in Annex X of WFD,” that “specific quality standards can and should be developed for sediment and biota.” The Expert Advisory Forum on Priority Substances and Pollution Control (EAF [55]), while developing a sequence of procedures for the program of measures, proposed the specific source/pathway ‘historical pollution from sediments’ (S11) for inclusion into an initial ‘source screening.’

An initial reference to this type of sediments and pollutants such as dioxins was given from the Elbe river basin by Förstner et al. (2004 [56]); the common characteristics of historically contaminated sediments (“HCSs”) and “historical pollutants” (“HPs”) is the limited ability for applying proactive measures to reduce their initial entrance into the aquatic environment.

Since then it is clear that the problems associated with both historic issues form a typical internal task of the river basin communities; here, a three-step strategy has been developed by Heise and Förstner (2006 [57]) for the assessment of risks on Rotterdam harbor arising from HCS in the Rhine river basin, by the identification of (i) substances of concern, (ii) areas of concern, and (iii) areas of risk with regard to the probability of polluting the sediments in the downstream reaches. The processes involved are dominated by mechanical re-suspension (Förstner et al. 2007 [58]), i.e., flood events, and this means, with regard to remedial measures, “a targeted stabilization or removal of highly contaminated historical deposits” [10].

On December 24, 2008, the Directive 2008/105/EC of the European Parliament and of the Council on environmental standards in the field of water policy was published [59, 60]; one of the amendments of the original European Water Framework Directive [1] refers to the need to improve the knowledge and data available on sources of priority substances and ways in which pollution occurs in order to identify targeted and effective control options. “Within the framework of the review of Annex X to Directive 2000/60/EC [1], as provided for in Article 16(4) of that Directive, the Commission shall consider inter alia the substances set out in Annex III to this Directive ([59] for possible identification as priority substances or priority hazardous substances. The commission shall report the outcome of its review to the European Parliament and to the Council by 13 January 2011.”

The next step in the progress of dioxin issues in aquatic systems was the publication of the Directive 2013/39/EU of the European Parliament and the Council of 12 August 2013 amending Directives 2000/60/EC and 2008/105/EC as regards priority substances in the field of water policy (Anonymous 2013 [12]). The revised EQS should primarily be considered in the River Basin Management Plans for period 2015–2021 [12].

### Spittelwasser under the water framework directive

The Spittelwasser area was chosen by the organizers of the international conference ConSoil 2000 for a case comparison and four expert teams from Denmark, Germany, the Netherlands,and the UK were invited. Evaluation of the plan was done by members of the NICOLE (Network for Industrial Contaminated Land) and CLARINET (Contaminated Land Rehabilitation Network) networks [61].

In the study of the German team (Wittmann et al. [62]), a stepwise approach combining monitoring techniques and remediation measures was identified by the environmental authorities to be used for the contaminated floodplain areas [63]. This approach provides for point excavations of critical material and also for the installation of sediment traps. It also includes the promotion of plant growth to stabilize the soils and sediments as well as support evapotranspiration. It has been argued that the design of geotechnical measures will mainly depend on the flow patterns of the water course during flood events. The plan for a pilot or test study on a part of the floodplain area was scheduled for a 4-year implementation period and 15 years for aftercare; it was calculated for initially 2.2 million EUR, not including the costs for sediment traps, excavations, and wetland construction (which would exceed the other costs by one to two orders of magnitude).

When Saxony-Anhalt’s new Agency for contaminated sites took over wider responsibilities in 2001, the offer of the Federal Ministry for Education and Research for funding, the German initiative in the Spittelwasser area was not further pursued by the Ministry of Agriculture and Environment in Saxony-Anhalt.

**Fig. 2:**
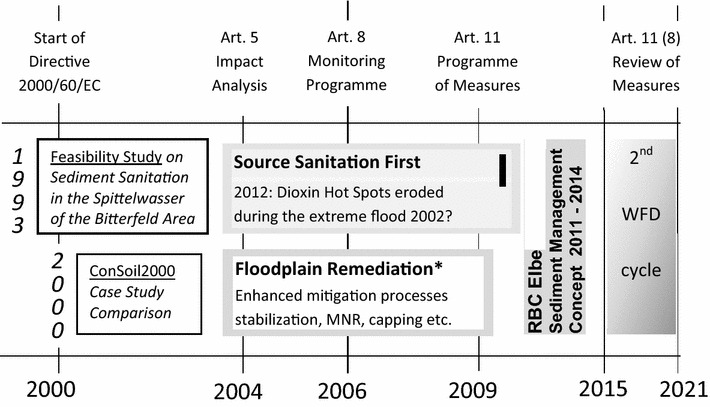
Scheme of the development of an integrated remediation concept for large-scale historical sediment contamination, Spittelwasser in the Bitterfeld District, Germany. *Time scale* is the stepwise implementation of the EU Water Framework Directive (Anonymous 2000a [1]. *Left side* “prerequisites”: (i) feasibility study from July 1993 (Anonymous 1993 [39]), (ii) Consoil2000 case study comparison (ConSoil2000 2000b [19, 61]). *In situ processes 2002–2010 (Förstner and Salomons 2010 [66])

From the involvement into the first Spittelwasser case study of 2000 [61] and from the experience of the various research projects during the following decade—e.g., KORA [64, 65]– the concept “Spittelwasser 2010” could be envisioned as two major steps ([66], Fig. 2):

1. *Source sanitation*. Excavation of approximately 20,000 m^3^ dioxin hot spot sediment from the Spittelwasser creek (“Spittelwasser remediation project (feasibility study 1993)” section).
2. *Floodplain remediation*, concerning a few tens of km^2^ of stable soils (“fluvisols”) and erodible channel sediments by new technologies such as monitored natural recovery (MNR), capping, bioremediation, phytoremediation, and embedding into a runoff control system (wetland approach in the widest sense [67]).

Within the common frame of the Large Ecological Project Bitterfeld-Wolfen (“Ökologisches Großprojekt Bitterfeld-Wolfen,” ÖGP), the managing State Agency for Contaminated Sites of Saxony-Anhalt (LAF) has spent 230 million EUR in the period from 2001 to 2010 for ground water sanitation, but no substantial responsibility was taken for the sediment issue ([66]; the first Bitterfeld sediment projects in the year 2008 did not even mention the substance group of PCDD/Fs [68, 69].

### Sediment management concept: prioritization of measures—Box 3: two versions

The sediment management concept of the River Basin Community Elbe was developed by the Ad hoc Working Group Pollutants/Sediment Management of the RBC Elbe headed by Dr. Peter Heininger (Federal Institute of Hydrology, Koblenz) in the time period of 2011–2013 (see Fig. 2 in “Spittelwasser under the water framework directive” section).

The *quality criteria* of the RBC Elbe for measures on contaminated sediments are based on a differentiation of two groups of Elbe-relevant substances; group 1, forming the regulation level “e” in the RBC Elbe Sediment Management Concept (RBC Elbe 2013 [7]), includes substances, which are explicitly regulated with respect to the protection of human health, e.g., As, Cd, Hg, Pb, HCHs, HCB, benzo(*a*)pyrene (PAH), and PCDD/Fs; other sediment contaminants are listed under the less stringent Group 2.

An initial classification—here for PCDD/Fs—follows the specific criterion (i) “the higher the ranking of the region of origin (here for either “contaminated sites” or “Sediment”; for the latter Bode, Saale or Elbe river) or source type (side structures, lock reservoirs, sedimentation zone, groin fields), respectively, the more urgent is the recommendation”; criterion (ii) is “the number of relevant substances of group 1 per source” and specific criterion (iii) is “the number of relevant substances of group 2 per source.” In a fourth step seven general criteria will be applied. The general criteria 1–4 act in the direction of an upgrade; examples are:

1. *Direct source.* The solution of a problem at the source and the elimination of the cause, respectively, had to be preferred (see Box 3: two versions).
2. *Near source.* If the causative source does no longer exist, the solution should be installed as close as possible to the original source (“stairwell cleaning from above”).
3. *Resonance 1*. Recommendation would have a positive effect on the other aspects (“hydromorphology” and “shipping”).
4. *Resonance 2.* One-shot investment affecting permanently reduced follow-up costs.

The criteria 5–7 indicate a tendency for downgrading the relevance of a certain measure, such as *“degree of difficulty/requirements for realization”* (No. 5) or *“reliability of predicting the success potential”*, i.e., due to the variability of the system (No. 6). The exclusion criterion *“missing proportionate solution potential”* (No. 7) will only be applied in exceptional cases at very well founded state of knowledge.

The prioritization scheme of the FGG-Elbe has not been applied to a full real situation, but a matrix is given in the Table 6-6 of the Sediment Management Concept (RBC Elbe 2013 [7]) for the selection of recommendations with regard to quality aspects during the sustainable handling of sediments and dredged materials, i.e., for preparing remedial measures in the Elbe river basin. We have posed our focus on dioxin-rich sites from the inventory of sediment volumes and their erodibility under different depositional conditions (Table 4).

**Table 4:** Criteria for the selection and prioritization of recommendations with regard to quality aspects during the sustainable handling of sediments and dredged materials (after “sediment management concept” of the river basin community Elbe (RBC Elbe 2013, Table 6-6 [7])

Region of origin (source type)	Substances (health)	Direct source	Near-source	Degree of difficulty	Success potential	Remediation measures
Sanitation contaminated sites *within* or *along* rivers/creeks/ditches						
*Within* **Spittelwasser** pre-2002 (1995 - ?)	α-, β-, γ-HCH; Dioxins/Furans	Yes	–	(Medium)	(High)	Excavation, incineration^d^
*Along* Spittelwasser post-2002 (2013)		**–**	Yes	Medium	Medium	*Stabilization* ^e^ *In situ, AC*
Elimination interim sediment depots						
**Saale** ^a^ (side structures)	Hg, Cd, Pb; α-, β-, γ-HCH; benzo(a)pyrene; dioxins/furans	No	Yes	Medium	??	LHW (2015)^f^
Saale^a^ (lock reservoirs)		No	Yes	Medium	??	LHW (2015)^f^
**Lower Bode river** ^a^ (sedimentation zones)	Dioxins/furans; Pb	No	Yes	Medium	??	LHW (2015)^f^
**Elbe** below km 300 (side structures)^b^	Hg, Cd, Pb, As; α-, β-, γ-HCH; HCB, B(a)pyrene; Dioxins/Furans	No	No	Medium	High	*Capping* ^g^ *Hitzacker/E*
**Elbe** below km 350 (Groin fields)^c^		No	No	Medium	??	–

In italic letters: own experience with stabilization^e^ and active capping^g^ technologies

^a^Saale and tributaries. In the navigable section of the Saale river approx. 190,000 tons of fine-grained sediments are deposited (spectrum of pollutants relevant to the river Elbe, incl. dioxins and furans), of which approx. 75 % are classified as remobilizable [(G.E.O.S. 2013 [71]; Wieprecht et al. 2013 [72]). In the Bode River 37,000 tons of fine-grained sediment were found (e.g., weir Stassfurt), 75 % remobilizable, lower Bode river high concentrations of dioxins and furans. The highest concentrations of dioxin—2220 bzw. 6650 ng I-TEQ/kg—were found in deeper layers of core sediments downstream from Stassfurt, where one of the production sites for light metals was located; see introductory section)

^b^Elbe river side structures (harbors, cut-off meanders, bays, blind channels; > 1.000, approx. 50 km^2^) comprise a total discharge potential of 20–100 Mio tons; 80 % located in the Elbe river section downstream from km 300 (Heise et al. 2013 [73])

^c^Groin fields. The Inland Elbe River exhibits 6.600 Groin fields which play a role as interim storage for the fine sediment transport, estimated for 1.3 Mio tons along the Elbe; more than 80 % of the muddy, relatively easily remobilizable material is located downstream of Elbe-km 350 (Hillebrand et al. 2014 [74])

^d^Excavation/Incineration. Feasibility Study for the Sanitation of the “Spittelwasser” Sediments (Anonymous 1993 [39]); see “Spittelwasser remediation project (feasibility study 1993)” section

^e^Solidification techniques at the TUHH 1982–2005 (examples [75–79]

^f^Initial plans for remediation measures were presented at the 23rd Chemical Colloquium of the German Federal Institute of Hydrology in Koblenz, June 11–12, 2015, by Petra Kasimir and Heinz-Jürgen John (Agency for Flood Protection and Water Management of Saxony-Anhalt [51]

^g^Demonstration plant Hitzacker/Elbe has been planned as the final step in a BMBF Research Project on Active Capping (1997–2003, Jacobs and Förstner [80–84])

The Table shows two different approaches for the remediation of the dioxin pollutants either as hot spots on the bottom of the Spittelwasser creek (“pre-2002-type”; approx. 20,000 m^3^ at hotspot, “Spittelwasser remediation project (feasibility study 1993)” section) or as areal deposits along this river’s course (“post-2002-type”), according to the findings from the study “Pollution Load Reduction Spittelwasser” of Tauw Consultants [19, 20].

This example reflects the typical development in remediation approaches described in the review on retrospective risk assessment “From bioavailability science to regulation of organic chemicals” by Ortega-Calvo et al. [18], when introducing bioavailability-based concepts at the transition from excavation procedures to regulations of organic chemicals [85]: One important tool was the explanation of bioavailability to regulators using the concepts given in this paper, which made it possible to design new remediation methods. If organic chemicals are immobilized, the flux from the soil to the pore water is low, usually too low for the contaminant to pose risks […]. Jurisdiction (in the example from Australia) now recognizes that the process of aging can be accelerated via chemically induced immobilization, which results in a rapid decline in bioavailability […]. After treatment, the bioavailable concentration of the chemical, measured as the concentration in the water phase, remained below the detection limit, and no toxicity for earthworms was observed […].

### Box 3: “Dioxin longitudinal profile 2008”—two versions

A significant step forward for the understanding of transport phenomena and dispersion of dioxin in the Elbe River was made in the “Dioxin Longitudinal Profile 2008” by Umlauf et al. [43]. The findings were: (a) No significant contribution from other PCDD/F sources was observed along a stretch of approximately 400 km (p. 30 in [43]). (b) The concentration levels as well as the downstream profile from 2002 to 2008 were rather similar, indicating minor change of the overall situation since 2002. The similarity of the PCDD/F levels is indicative of a stable interrelationship over the course of a long period of time and this observation provided strong arguments for the “source-first” approach in the study “Assessment of Risks from Particle Bound Substances in the Elbe River Basin” (Heise et al. [14]).

The conclusions of Umlauf et al. [43] in the original—English—version were: *“(1) The main dangers with respect to dioxin contamination in the Elbe are high water events occurring in the Spittelwasser*-*Mulde*-*Saale system. (2) Consequently, an improvement in the immission situation for the Elbe can only be expected after the corresponding sources have been adequately cleaned up. (3) A reduction in the pollutant loads in the Elbe would have a positive effect on the immission situation in the coastal parts of the North Sea as well”*. In the German version (Stachel et al. [70]), which was edited by the River Basin Community Elbe, the statements (2) and (3) referring to the *source*-*first principle* and to the *immission situation* of the North Sea were deleted—both would have contradicted to the LAF position in the contentious dialog (see above). Two years later, however, the Sediment Management Concept of the ad hoc Working Group (RCB Elbe [7]) gave the source-first principle the priority among seven general criteria: *“The solution of a problem at the source and the elimination of the cause, respectively, have to be preferred.”*

### Dioxin stabilization using activated carbon technologies—Box 4: passive sampling

Since the turn of the century activities at North American sediment cleanup sites increasingly move to in situ technologies such as *sediment capping*, a form of in situ containment, and *monitored natural recovery* (MNR), where natural processes are used to mitigate the transfer of particle-bound contaminants into the water phase and/or biota; the latter processes could be supported and enhanced by additives (e-MNR, e.g., [86]). *In situ* treatment is generally less disruptive and less expensive than traditional sediment cleanup technologies. There continue, however, to be gaps in our knowledge of the fate of contaminants in place, and the effects of in place and ex situ remedial strategies, *“which must be filled if management strategies are to be compared and chosen wisely”* (Apitz et al. [87]). This means, that *“a shift of emphasis is needed toward the use and communication of results from the analyses of multiple lines of evidence,”* e.g., by examining the potential impacts of large, low-probability events or combination of probabilities (e.g., the 100-year flood and the probability of erosion to a specific depth) on exposure and risk, and the associated uncertainties (Bohlen and Erickson [88]).

Extensive experimental studies and field trials have shown that when applied correctly, in situ treatment via contaminant sequestration and immobilization using a sorbent material such as AC has progressed from an innovative sediment remediation approach to a proven, reliable technology [21]; many cases in the USA and EU [89, 90] have demonstrated to decrease the bioavailability of PCBs and PAHs in soils and sediments [91]. Activated carbon reduces pore water concentrations by sequestration of the chemicals through adsorption to the AC surface and within its pore structure (Jonker & Koelmans [92]); in addition, AC has slow kinetics of contaminant desorption, which implies that fluxes of HOCs to the aqueous phase are low, and this limits contaminant mobility in the aquatic environment [90].

*Passive sampling* has been performed with different systems in which chemicals partition between the dissolved phase and a solid or liquid sampling phase without significantly affecting the soil–water or sediment–water equilibrium [22]. Primary considerations for selecting a passive sampling method (PSM) for a specific application include clear delineation of measurement goals for *C*_*fre*e_, whether laboratory-based “ex situ” and/or field-based “in situ” application is desired, and ultimately which PSM is best-suited to fulfill the measurement objectives [21] (see Box 4).

Over the past decade, pilot- or full-scale field sediment treatment projects using AC were completed or were underway at more than 25 field sites in the United States, Norway, and the Netherlands; collectively, these field projects (along with numerous laboratory experiments) have demonstrated the efficacy of AC for in situ treatment in a range of contaminated sediment conditions [21]. In general, the effectiveness of AC to reduce bioaccumulation appeared to be species-specific, i.e., bioaccumulation is known to depend on the physiology and behavior of the organisms, which drive factors such as ingestion rate, assimilation efficiency, and elimination (Janssen and Beckingham [21, 96]. *In situ* sediment treatment involves targeted placement of amendments using installation options that fall into two general approaches:

1. Directly applying a thin layer of amendments (which potentially incorporates weighting or binding materials) to surface sediment, with or without initial mixing; and
2. Incorporating amendments into a premixed, blended cover material of clean sand or sediment, which is also applied to the sediment surface [90].

*Costs* Among the projects mentioned above, field demonstrations in the lower Grasse River (Massena, NY, USA [97] and upper Canal Creek (Aberdeen, MD, USA [98, 99]) included the most comprehensive assessments and available documentation of the longer term efficacy of the in situ AC remediation approach (here mainly on PCB contamination), although similar results have been reported for many of the other field projects; these two sites received the greatest attention in the performance and cost evaluation by Patmont et al. [21] given in Table 5. The general information from these studies is twofold: (1) The costs are in the same order of magnitude for both the excavation/incineration approach of the Spittelwasser feasibility study from 1993 [39] and with the AC in situ stabilization technique, i.e., in the range from 10 to 20 million $US or € for either the hot spot 20,000 m^3^ or 20 hectares areal deposits; (2) technology development around AC applications involves input of a number of high-ranked scientists from biogeochemical and environmental engineering disciplines.

**Table 5:** Summary of low and high-range unit costs of AC application (Patmont et al. [21])

Component	Low-range unit cost	High-range unit cost
Activated carbon^a^	$50,000/hectare	$100,000/hectare
Facilitating AC placement using binder/weighting agents^b^	$0/hectare	$70,000/hectare
Facilitating AC placement by blending with Sediment or sand^b^	$0/hectare	$100,000/hectare
Field placement	$30,000/hectare	$200,000/hectare
Long-term monitoring	$20,000/hectare	$100,000/hectare^c^
Total	$100,000/hectare	$500,000/hectare

Estimated costs for a 4 % AC dose (dry weight basis) over the top 10 cm-sediment layer at a 5-hectare site

^a^Powdered activated carbon (PAC) and/or granular activated carbon (GAC), depending on site-specific designs

^b^To facilitate AC placement, binder or weighing amendment such as SediMite^R^ or AquaGate™, or clean sediment or sand (but typically not both) may be required in some applications depending on site-specific conditions and designs

^c^High-end monitoring costs of $100,000 per hectare reflects prior pilot projects and likely overestimates costs for full-scale remedy implementation

### Box 4: Passive sampling in the monitoring of dioxin under WFD and US EPA superfund

A recent review in ES&T [93] builds upon the findings of an ICES-workshop on the utility of passive sampling for the risk assessment on contaminated sediments [94] and gives the special focus on the comparison of situations under both the WFD and US EPA Superfund:

In Europe, the strict monitoring requirements laid down in the WFD and its daughter directives impede the implementation of passive sampling for regulatory purposes, whereas in the United States the use of passive sampling in the implementation of remediation processes for contaminated sediments has been encouraged by regulators [95]. The latter is due to the recognition that passive sampling-based C_free_ data, in particular of non-polar organic compounds, provides a better scientific basis for risk assessment, compared with conventional sampling and monitoring procedures. The actual use of passive sampling is limited by the lack of commercial laboratories performing passive sampler deployments and data reporting; in the near future, the scientific community will be crucial in providing guidance on the standardization of passive sampling methods [93].

## Scientific support for dioxin- and WFD-related EU-directives

During the implementation of the European Water Framework Directive it became clear that the original legislative system has to be completed by further central “directives,” three of them in the aspect “pollutants” and these with special reference to the substance group of “dioxins”:

1. The Directive 2013/39/EC from August 24, 2013 reacts on the *“actual legal situation”* (No. 3 in Table 1) at Environmental Quality Standards (EQS) for priority hazardous substances. With the PCDD/Fs a substance group is involved, which has not been regulated so far, but may provide the widest consequences.
2. The Directive 2007/60/EC of October 2007 on the assessment and management of flood risks increasingly deals with the specific *challenges from particle*-*associated priority pollutants*, such as dioxins (No. 7 in Table 1).
3. Downstream from pollutant sources, possibly until the coastal waters and the North Sea, environmental objectives can only be achieved if the pollutant loads existing in the upstream catchment are reduced or eliminated (Marine Strategy Framework Directive 2008/56/EC of June 2008).

These three directives are highly interconnected. Directive 2013/39/EC is a direct amendment of the WFD, with direct implications for the chances of achieving the “good chemical status” of surface waters. For each Priority Hazardous Substance newly defined by the directive, EQS values, i.e., effect-based numerical quality criteria for assessing the status of an aquatic system, need to be derived and enforced. The implications of including PCDD/Fs and dl-PCBS in the list of Priority Hazardous Substances may have wide consequences of our ability to reach the “good chemical status” in many European catchment areas. “Directive 2013/39/EC—policy and science for biota-EQS of DLCs” section summarizes the current legal situation and novel approaches in the context of Directive 2013/39/EC. Furthermore, it has been stated that it is imperative to closely coordinate measures in context of the Floods Directive, the MSFD and the WFD to be able to reach the individual goals of each of the legislations [100, 101]. One particularly illustrative example may be the managed realignment of dikes as a measure to (1) increase the storage capacity of floodwater, which (2) reduces the bed shear stress and the risk of erosion of contaminated sediments while at the same time (3) increasing the structural diversity of the river ecosystem. “Directive 2007/60/EC—policy and science with special reference to dioxin-like compounds and flood risks” section illustrates the implications of Directive 2007/60/EC in policy and science with a special reference to dioxin-like compounds in context of flood events. Last, sediments and suspended particles can be thought of as a transport vector for lipophilic pollutants and thus represent a direct link between WFD and MSFD, since marine systems are the ultimate recipients of the SPM loads of our rivers; this aspect is particularly important in Elbe river basin as discussed in “Marine strategy framework directive 2008/56/EC—sediments and pollutants” section (e.g., [183]).

### Directive 2013/39/EC—policy and science for biota-EQS of DLCs

#### New priority hazardous substances in the field of water policy

The implementation of a community strategy for PCDD/Fs and PCBs was first mentioned expressively in the Second progress report (Annex) (COM(2001)593) from 10.7.2007 (Anonymous 2007 [102]); before, in a Commission Staff Working Document “Impact Assessment,” the growing experience was expressed, that “many of the substances of highest concern persist in the environment for a long time; even after their use has been banned and discharge restricted, these substances continue to be found in high concentration in the environment 10, 20, or more years later, and some of them have travelled to remote areas” (Sect. 3 in Anonymous 2006 [103]). In this Working Document, PCDD/Fs and PCBs were not included in the list of priority substances under the WFD as they were considered to be historic pollutants and adequately controlled, “but may be considered for future inclusion depending on new monitoring data” [103]. The Commission Staff Working Document “Impact Assessment” [103] requires from the WFD to submit proposals covering (1) quality standards applicable to the concentrations of the priority substances in surface water, sediments or biota (Article 16(7) WFD) and (2) for priority substances controls for the progressive reduction of discharges, emissions and losses.

Directive 2013/39/EC (from August 24, 2013 [12]) reflects the latest development referring to newly identified priority substances such as PCDD/Fs and dioxin-like PCBs, and there are consequences for the chemical status under the WFD as well as for the adaptation of the German Surface Water Ordinance (OGewV) [15]. Here, the relevance of the biota-EQS criterion for a target achievement is high for mercury, as has been proven from the study of environmental specimen bank organisms (e.g., fish, molluscs, and crustaceans; Wellmitz [104]). The EQS value of 0.0065 μg TEQ/kg for dioxin and dioxin-like compounds (which is based on the maximum permitted levels in the meat of fish for human consumption defined by regulation 2011/1259/EC) was exceeded by >50 % of the tested organisms in the specimen bank (Mohaupt et al. [105]).

Three EC-Documents refer to methodological aspects: (i) Technical Guidance on the Preparation of an Inventory of Priority and Priority Hazardous Substances [106], (ii) and (iii) regulations for dioxins, dioxin-like PCBs, and non-dioxin-like PCBs in foodstuffs [107, 108]. The following chapter deals with EQS for dioxin-like compounds (DLCs) and cell-based bioassays for detection of DLCs.

#### EQS for DLCs and cell-based bioassays for detection of DLCs

As mentioned above, the European Water Framework Directive (WFD; EC 2001 [2]), as well as the subsequent directives 2008/105/EC and 2013/39/EU (EC 2008 [59], EU 2013 [13]), defined a total of 45 priority substances. These include PCDD/Fs and dl-PCBs in order to manage and reduce risks based on dioxin-like compounds (DLCs). Sediments can act as long-term sinks for these and possibly other DLCs. Due to their high lipophilicity and persistency these DLCs are known to accumulate in aquatic organisms (e.g., in fish), predominantly via dietary routes of exposure (La Rocca and Mantovani [109], Spagnoli and Skinner [110]) and have been demonstrated to cause a plethora of acute and chronic toxic effects (Mandal [111]). Furthermore, environmental quality standards (EQSs) for the concentrations of these priority substances in the water phase and biota were defined toward a better level of protection in retrospective risk assessment. EQSs, also referred to as action or trigger values, are important tools in sediment assessment frameworks (Apitz and Power [112]) for identifying effects or no effects of sediment-borne contaminants (Wenning and Ingersoll [113]). In 2013, regulation 2013/39/EU [12] entered into force, which established an EQS of 6.5 pg TEQ/gfm for DLCs in biota. Although this represents an important step, it remains unclear how these EQSs for biota translate to EQSs for environmental media, which currently cannot be determined due to the lack of experimental data (EQS dossier 2011 [114]). In particular, the role of sediments, which represent long-term sinks as well as secondary pollution sources, has not yet been sufficiently considered. Since the impact of sediment-borne contaminants on aquatic systems is highly dependent on regional factors, such as erosion risk, sediment particle size distribution, and the structure of the aquatic biocenosis, the directives 2008/105/EC and 2013/39/EU allow the European member states to establish national EQSs for biota and sediments, which can be applied instead of the established water phase EQSs (Carere et al. [115], Maggi et al. [116]).

DLCs share common structural properties. They bind to the cytosolic aryl hydrocarbon receptor (AhR), a ligand-activated transcription factor that regulates the expression of a number of genes. It is believed that the transcriptional responses to AhR activation trigger a major fraction of the documented adverse effects of DLCs (Kawajiri and Fujii-Kuriyama [117], Okey et al. [118]). In this respect, scientists increasingly discuss the role of in vitro bioassays for a biological effect-based assessment in decision making frameworks (Ahlf et al. [119], Besselink et al. [120], den Besten et al. [121]). In vitro bioassays may serve as screening tools for the detection of DLCs in various environmental matrices including foodstuffs (2014/589/EC [108], Eichbaum et al. [122]) because they possess a proven correlation and predictive ability for DLCs. They can support classical, instrumental analysis of individual DLC congeners present in complex mixtures (e.g., sediment or tissue samples) by providing more realistic, ecotoxicologically relevant information. Moreover, they allow for both, the integration of all interactions among DLC congeners and detection of inducers not monitored in compound specific instrumental analyses (Giesy et al. [123], Wernersson et al. [124]). Bio-analytical and instrumental results can be compared by using the approach of toxicity equivalent quotients (TEQs) and biological equivalent quotients (BEQs, Van den Berg et al. [125]).

Since the year 2004, successful implementations of in vitro assays for the screening of DLCs in form of the DR-CALUX assay can be found in the Dutch dredging guideline for coastal sediments, which formerly only included chemical analysis. Here, a biological equivalent quotient (BEQ) signal value of 50 ng BEQ/g dry weight (dw) sediment has been set, which—if exceeded—involves further, detailed investigations (Manz et al. [126]). In German legislation, in vitro assays as semi quantitative methods prior to quantitative instrumental analysis have only been established in the field of food analysis, where BEQs allow for simple yes/no-decisions (2012/252/EU [127]).

### Directive 2007/60/EC—policy and science with special reference to dioxin-like compounds and flood risks

In the Elbe river, the summer flood of 2002 was a catastrophic event for a large part of the catchment area (damage of ~25 billion € [BfG 2002 [128]). This case can be seen as the starting point for intensified research on mechanical effects of historical contaminated sediments (HCS, [129–131]; for example, a comprehensive study on the relocation, dilution and export of metal-polluted sediment in the Saale catchment area was performed by Hanisch et al. [132].

### Box 5: Climate change and WFD river basin management—policy and science [137]

With the development of the first river basin management planning under the water framework directive (WFD), which operationally started in 2010, the integration of knowledge about possible climate change impacts on water policy implementation concerns various technical aspects (risk characterization, monitoring, action programs) as well as the evaluation of the “good status” objective’s achievements in 2015 (Quevauviller 2011 [137]). The interface between policy and science in this field is dealt in two recent articles: (1) The obvious drawback is that for a single operational framework the scientific basis is divided between two large communities: the disaster risk reduction community and the climate change adaptation community, both of which are bound to different research and operational funding budgets [138]. (2) A review of existing gaps and future research needs based on the findings EU FP7-funded Co-ordination and support action “ClimateWater” [139].

#### Development of the EU-directive on flood risks

Research projects on flood related hazards including HCS aspects were funded by EU-DG-RTD [133] and BMBF, the latter in the RIMAX (risk management of extreme flood events) coordinated project, e.g., on dry basins and polder for flood retention [134]. During the June flood 2013 at the Elbe river the measurement program “extreme events” of the [RBC Elbe [135] included data of pollutant concentrations and discharges; at Wittenberg and Magdeburg an increase of the dioxin load up to the three to fourfold during this flood event was observed. Contrary to this statement, the draft of the flood risk management plan for the German part of the Elbe river areal unit (RBC Elbe 2014 [136]) just mentions “sediments” once—in the glossary (“the term ‘sediment dynamics’ comprises transport-, deposition-, and remobilization processes of sediments”).

#### Ecotoxicology in context of sediment mobility and, flood risk assessment

Remobilization of highly contaminated sediments is a key driver for apparent sediment toxicity in aquatic systems (Westrich and Förstner 2007 [140]) and the assessment of sediment stability has been identified as an important and emerging factor that also needs to be considered in the implementation of the WFD (Hollert et al. [141, 142]). Pollutant mobilization from soils and sediments have been described as “time bomb effects” by William Stigliani (1988 [143], 1991 [144]); here, chemical time bombs are defined as “*a chain of events resulting in the delayed and sudden occurrence of harmful effects due to the mobilization of chemicals stored in soils and sediments in response to slow alterations of the environment.”* The re-suspension of sediments certainly possesses some of these characteristics and the frequency and intensity of flood events such as the 500 year flood at the River Elbe in 2002 (Schüttrumpf and Bachmann [145]) are expected to increase in the future because of global climate change [146–148].

Several experimental laboratory and in-field methods are available to determine the critical bed shear stress for erosion, i.e., the bottom shear stress at which mass erosion of the sediment layer occurs [149–154]. Toxicity testing, however, was mostly conducted in the presence of static sediment layers and is thus only representative of average flow conditions and does not allow prediction of the effects of sediment re-suspension (e.g., [155–157]). Another approach that was commonly used as a proxy to assess the toxicity of sediments when re-suspended is that of testing sediment elutriates [158, 159]. It should be emphasized, however, that the approach is of relatively limited value for very lipophilic compounds. In particular, experimental research at the intersection between hydrodynamics and ecotoxicology has developed into a promising field that may help to overcome these limitations [160–167].

The scientific community urgently requires standardized protocols to assess the impact of sediment suspension exposure on biota. This chapter describes a number of recent projects and studies that were initiated in response to these needs.

##### a) The project framework FloodSearch

In context of the interdisciplinary project framework FloodSearch, which was funded by the German excellence initiative, methods of hydraulic engineering and ecotoxicology were experimentally combined. To this end, exposure experiments with contaminated sediments were conducted in (a) static re-suspension tanks or (b) an annular flume, an experimental facility in which rainbow trout (*Oncorhynchus mykiss*) can be exposed under simulated flood-like conditions (Cofalla et al. [161], Hudjetz et al. [164]. Schüttrumpf et al. [166], Wölz et al. [167], Brinkmann et al. [168, 170]). The investigated sediments were either spiked with a mixture of PAHs or sampled in the field, the latter of which contained different levels of DLC contamination that were aged under natural conditions. A battery of different biomarkers, i.e., measurable biological responses of fish during exposure to the contaminated sediments, was established to verify exposure to and effects of different DLCs. This battery included hepatic activities of the enzymes 7-ethoxyresorufin-*O*-deethylase (EROD), glutathione-*S*-transferase (GST), and catalase (CAT), lipid peroxidation in homogenized liver tissue, as well as the induction of nuclear aberrations (micronuclei) in peripheral erythrocytes. Furthermore, metabolites of PAHs were chemically analyzed in bile liquid of exposed fish to demonstrate uptake and metabolism of these compounds (Kammann [171], Kammann et al. [172]).

Exposure to spiked sediments led to a significantly induced frequency of micronuclei, which correlated well with the concentration of 3-hydroxybenzo[*a*]pyrene in bile liquid, a metabolite of the genotoxic PAH benzo[*a*]pyrene [168]. The enzymatic biomarkers did not indicate any significant alterations in these treatments. In fish exposed to re-suspended natural sediments, significant differences in bile metabolite concentrations as well as in 7-ethoxyresorufin-*O*-deethylase induction were observed compared to control experiments. The biliary concentrations of 1-hydroxypyrene from fish exposed to the three different contamination levels correlated well with the ratio of pyrene concentrations in corresponding bulk sediments and the ratio of particle-bound pyrene in suspended sediments. In contrast, hepatic lipid peroxidation and micronuclei formation represented the different contamination levels less conclusive in these treatments.

Using the newly established interdisciplinary methodology, the studies within the project framework FloodSearch clearly demonstrated that particle-bound DLCs, here predominantly PAHs, from sediments aged under natural conditions may become bioaccessible upon re-suspension during short simulated flood events and are readily absorbed by aquatic organisms such as rainbow trout. The associated short-term effects were clearly documented within the studies, and potential adverse long-term impacts are likely to follow.

##### b) The DioRAMA project

The DioRAMA project, a cooperation between the Institute for Environmental Research at RWTH Aachen University (Aachen, Germany) and the Department G3 (Biochemistry/Ecotoxicology) of the BfG (Koblenz, Germany), was initiated to promote interdisciplinary research involving chemical and biochemical analyses, ecotoxicology, and risk assessment (Eichbaum et al. [169]). The project was coordinated by the BfG, while experimental investigations were mostly performed at the Institute for Environmental Research. Additional collaborations were established with the University of Saskatchewan (Saskatoon, Canada) and Münster analytical solutions (mas; Münster, Germany). The experimental work within the DioRAMA project was conducted in close exchange with and adjusted to the regulatory practice of the BfG in order to derive useful tools for future application in sediment management. Furthermore, additional collaborations were established with the Thünen Institute of Fisheries Ecology (Hamburg, Germany), the Fraunhofer Institute for Molecular Biology and Applied Ecology IME (Schmallenberg, Germany), the Swiss Federal Institute for Materials Science and Technology (EMPA; Dübendorf, Switzerland), and the Centre for Fish and Wildlife Health (Bern, Switzerland). The DioRAMA project profited largely from the experiences made during the previously introduced projects FloodSearch and FloodSearch II [161, 166, 167].

One major part of the DioRAMA project focused on the establishment and validation of cell-based bioassays for the detection of DLCs in sediments (Eichbaum et al. [16]), while in the second main experiment within the project, the time- and concentration-dependent uptake of DLCs and their associated effects were investigated in rainbow trout (Brinkmann et al. [173]). Exposure experiments were conducted using suspensions of three field-collected sediments from the rivers Rhine and Elbe, which were chosen to represent different levels of contamination. Five serial dilutions of contaminated sediments from the Prossen and Zollelbe sampling sites (both in the Elbe, Germany) were tested and compared with moderately contaminated sediment from Ehrenbreitstein sampling site (in the Rhine, Germany). Fish were exposed to suspensions of these sediment dilutions under semi-static conditions for 90 days. Uptake of particle-bound PCDD/Fs, PCBs, and PAHs was determined by high-resolution gas chromatography and mass spectrometry (HRGC/HRMS) analysis of muscle tissue and high pressure liquid chromatography analysis of bile liquid. Additionally, fish responses to DLCs (EROD activity, micronuclei, and other nuclear aberrations, histopathological, and gross pathological lesions) were investigated.

Analysis of muscle tissue and of bile liquid showed that particle-bound PCDD/Fs, PCBs, and PAHs were readily bioavailable from re-suspended sediments. Uptake of these contaminants and the associated toxicological effects in fish were mostly proportional to their sediment concentrations. The changes in the investigated biomarkers closely reflected the different sediment contamination levels: cytochrome P450 1A mRNA expression and 7-ethoxyresorufin-*O*-deethylase activity in fish livers responded immediately and with high sensitivity, while increased frequencies of micronuclei and other nuclear aberrations, as well as histopathological and gross pathological lesions, were strong indicators of the potential long-term effects of re-suspension events. This study clearly demonstrates that sediment re-suspension can lead to accumulation of PCDD/Fs and PCBs in fish, resulting in potentially adverse toxicological effects. For a sound risk assessment within the implementation of the European Water Framework Directive and related legislation, we propose a strong emphasis on sediment-bound DLCs in the context of integrated river basin management plans. Currently, it is sought for practical implementations of these findings at least for the assessment of dredged materials. In addition to the experimental work conducted within the project, computational models were developed, which will be highly valuable to derive scientifically sound sediment quality standards (Brinkmann et al. [174, 175]).

### Marine strategy framework directive 2008/56/EC—sediments and pollutants

The MSFD [12] takes account of land-based as well as fluvial aspects and ensures comparable approaches and methodologies as in the WFD.

#### Marine strategy framework directive—descriptor 8: contaminants

A commission decision of 1 September 2010 on criteria and methodological standard on good environmental status of marine waters characterized the descriptor 8 “Concentrations of contaminants are the levels not giving rise to pollutions effects,” levels of pollution effects on the ecosystem, components concerned, having regard to the selected biological processes and taxonomic groups where a cause/effect relationship has been established and needs to be monitored [176]. The Task Group 8 report “contaminants and pollutions effects” was published in April 2010 under JRC European Commission and ICES [177]. By 15 July 2012, Member States had to prepare the first elements of marine strategies, namely the initial assessment (Article 8), the determination of good environmental status (GES—Article 9), and the establishment of environmental targets and associated indicators (Article 10) and to report them to the Commission by 15 October 2012 [178]. The German report to Article 12 of the MSFD obligations was published on 7 February 2014 [179]. The OSPAR Commission (Convention for the protection of the marine environment of the North-East Atlantic) accompanies the MSFD implementation in several aspects, e.g., with a literature survey on dioxins [180], an advice document on “Good Environmental Status—Descriptor 8: Contaminants” [181] and a “Regional Plan to Improve Adequacy and Coherence of MSFD Implementation 2014–2018 [182]”. Wenzel [183] has outlined the significance of the sediment contamination issue between WFD and MSFD with special reference to the Elbe river basin. On the other hand, the draft of the program of measures for the MSFD in the German North Sea and Baltic Sea [184] neglects the aspect sediment contamination and does not mention the dioxin problem in the Elbe; most likely, this is due to the influence of the Federal States Working Group Water and Wastewater (LAWA); deficiencies were observed in their respective contributions to the German versions of the program of measures for the European Water Framework and Flood Risk directives.

#### Dioxin at the land/sea interface of the Elbe river basin

According to an early dioxin balance for Hamburg [185], in the mid-1990s, approx. 23 g I-TEQ per year were extracted with dredged materials—approx. 300,000 t dry mass—from the Elbe and harbors of the city area, and were deposited on secure sites; approx. 47 g I-TEQ per year were transported in the direction North Sea. Uhlig et al. [186] from analyses of dioxin congener patterns of suspended particulate matter and sediment samples from the Elbe, Spittelwasser, Mulde, Saale, and Schwarze Elster came to the conclusion that 70–82 % of the dioxin contamination of the Elbe sediment in Hamburg can be attributed to sediments from the Mulde, whereby the study took account of both direct transport from the Mulde and also indirect transport via intermediate deposition. For the extreme summer flood of 2002, on the basis of measured SPM deposits on the floodplains of the Elbe, together with analysis results, it was estimated by Stachel et al. [187] that the contamination of the flooded soils was increased to a degree lying between 4.3 and 6.5 g WHO 1998-TEQ. Between 3.1 and 4.6 g WHO 1998-TEQ of PCDD/Fs were transported toward the North Sea over the weir at Geesthacht. Higher levels of dioxins (as compared with a neighboring reference area) were found off-shore in the North Sea South of Helgoland in the area previously used for dumping treated sewage from Hamburg. However, the PCDD/F fingerprints found there point to the Bitterfeld region rather than to impacts from both sewage dumping [43].

## Conclusions and outlook

On 1 July 2015, the European Court of Justice (ECJ) rendered its long-awaited judgment interpreting the EU Water Framework Directive 2000/60 in relation to projects such as the deepening of the Weser River in Northern Germany. The ECJ ruled that the environmental objectives of the WFD are not merely objectives for management planning with no link to or impact on individual projects; rather, Member States may not authorize projects which may cause a deterioration of the status of a surface water body unless derogation is granted [3]. Regarding the term “deterioration” the Advocate General Jääskinen recommended a strict interpretation of the WFD with reference to a substance or quality component, without affecting a mandatory classification change [188].

The so far successful initiative of the German NGO “BUND,” just in time with the second river basin management plans, confirms the ability of the WFD to adopt concrete measures for both safeguarding a good state and for preventing the deterioration of surface waters. This coincides with the sustainable development strategy, an overriding environmental goal, which is mandatory for all policy sectors and measures within the European Union [189].

From the retrospective to the first WFD phase in the Elbe catchment it seems that “PCDD/Fs and dl-PCB” can be used as a spearhead for a new understanding of problem solutions, where a fundamental change is required in the view on the implementation of the Marine Strategy Framework Directive (below). In the Elbe River basin, following the quasi-ban of dioxin issues from Saxony-Anhalt’s environmental agenda in 2009 [9] and the decision of the German Federal States along the North Sea coast to exclude dioxin from their list of “common transitory provisions for handling dredged materials in coastal waters” [190], practical all sanitation activities on dioxin and historical contaminated sediments downstream from the Bode/Saale and Spittelwasser/Mulde were blocked. This has happened despite an ambitious Sediment Management Concept of the River Basin Community Elbe from November 2013 [7] and the inherent responsibilities from WFD-near Directives with respect to the improvement of the chemical quality in surface waters:

1. Under the *Directive 2013/39/EC* [12] the relevance of the biota-EQS criterion for a target achievement of Elbe-typical dioxins and dioxin-like PCBs is particularly high. It is predicted by Mohaupt et al. [105] that in the second River Basin Management Plan, the new and extended EQS-requirements will lead to a failure of the good chemical status in all water bodies; in the third RBMP the EQS values of the eight specific substances will continue to be exceeded, however, with less actual/target status.
2. *Directive 2007/60/EC* [13] on assessment and management of flood risks, relates to the special role of sediment-bound contaminants is due to the exponential increase of solid/pollutant loads with higher water velocities. The assessment of erosion stabilities was one of the major achievements of the Sediment Management Concept of the RBC-Elbe (RBC Elbe 2013 [7]; another was the development of criteria for the prioritization of measures).
3. *The Marine Strategy Framework Directive* (MSFD, 2008/56/EC [5]) is similar to the WFD, in its stepwise implementation mode and with its main objective in the achievement of good environmental status (GES) in the marine environment by 2020. Between the source of a pollutant and its final receptor, the sea, we have the temporal storage in flood plains, lakes, artificial lakes behind dams, deltas, and man-made sedimentation traps (harbors); these are temporary receptors on the temporal scale of years to decades (Salomons [191]). Using scenarios and linking source to receptors has been carried out for *prediction of sediment quality in harbors* (Salomons and Gandrass [192]). In these cases the impact at the receptor could be defined as exceeding the standard for disposal at the sea (Heise et al. [193]); which is sufficient for the harbor manager [191]. For example in the scenario for the Rhine sediment between the barrage Iffezheim and Rotterdam, the hexachlorobenzene (HCB) source can contribute to a failure of the objectives of the WFD in the Rhine Basin [194, 195] and may require additional measures for its control [57]. All of these should be indications for a mandate to the RBC Elbe; however, no activities at the land–sea interface are reported in the Actualized RBMP (RBC Elbe 2015 [4]).

There is a characteristic difference between dioxin and other substances of the Directive 2013/39/EC, many of which can be reduced by technical measures on the basis of product regulations, approvals etc. [196]. For dioxins as historical pollutants, the proactive option is mostly missing, and the aftercare depends on the solidarity among the members of the River Basin Community.^1^

In the costly approval process of the Port of Hamburg for deepening of the Elbe river course [198], involving translocation of additional 1 million m^3^/a fine-grained sediment from the tidal Elbe, the pollutant aspect already plays the most critical role [199]. At the end of the day, under the stricter claims from the Marine Strategy Framework Directive compared to the WFD, the only convincing argument would be a significant reduction of the pollutant discharges, with special reference to the Elbe-typical PCDD/Fs, from the catchment area into the North Sea (Additional file 1).

## Additional file

10.1186/s12302-016-0075-8 A wider overview on dioxin and historical contaminated sediments in the Elbe River basin before and during the first phase of implementation of the European Water Framework Directive, including controversial issues between administration and specialist science ([200], in German).

## Footnotes

1 As to the RBC Elbe, the Free and Hanseatic City of Hamburg, according to the principles of a coordinated management (WFD Art 4 and Appendix V [4]), has a claim against an upstream riparian, by name Saxony and Saxony-Anhalt, for conducting hydraulic engineering and sanitation measures, which are practically needed to eliminate and reduce specific pollutant inputs from upstream risk areas (Breuer 2008 [197]).
