# Supplementary material for: Dioxin in the Elbe river basin: policy and science under the water framework directive 2000–2015 and toward 2021
Source: Environ Sci Eur. 2016 Mar 29;28(1):9. doi: 10.1186/s12302-016-0075-8 (PMC5044960; doi:10.1186/s12302-016-0075-8)
Supplement: Supplementary file 1 — 10.1186/s12302-016-0075-8 A wider overview on dioxin and historical contaminated sediments in the Elbe River basin before and during the first phase of implementation of the European Water Framework Directive, including controversial issues between administration and specialist science ([200], in German). [file 12302_2016_75_MOESM1_ESM.docx]

**Dioxin und Sedimentaltlasten im Elbeeinzugsgebiet unter der WRRL 2000-2015 –**

**Kontroversen zwischen Behörden und Fachwissenschaften**

Ulrich Förstner, Hamburg*

1. **Altlastensanierung nach der Wende – neue Pflichten gegenüber den Elbe-Unterliegern**
2. **Herkunft der Dioxin-Altlasten und ihre Verwaltung in den 1990er Jahren**

2.1 Herkunft der Dioxin-Altlasten in Sachsen-Anhalt und in der Unterelbe

2.2 Überwachung der Dioxin Hot Spots im Spittelwasser durch das STAU D/W

2.3 Machbarkeitsstudie zur Sedimentsanierung des Spittelwassers (1993)

**3. Fallstudienvergleich ConSoil2000 und nachfolgendes „dioxinfreies“ EU-Teilprojekt**

3.1 UK; 3.2 Danmark; 3.3 Niederlande; 3.4 Deutschland – ausgewählte Schwerpunkte

3.5 Weiterführung des NL-Beitrags im EU-Projekt „WELCOME“ (1/2001 – 12/2003)

**4. Sachsen-Anhalt sucht seine Rollen unter der WRRL und in der FGG Elbe (2005)**

**5. Landesanstalt für Altlastenfreistellung gegen das „Source First-Prinzip“ und „Dioxin“**

- 1. LAF-Kritik an HPA-Studie: „Keine Rücksicht auf sensible Situation in Sachsen-Anhalt“
  2. Bewirtschaftungsplan: FGG Elbe entzieht Dioxin den Prioritätsstatus im ÖGP Bitterfeld

5.3 Dioxin-Längsprofilaufnahmen 2008 – FGG Elbe lässt Hinweis auf „Source First“ streichen

**6. Interne LAF-Studien zu Altlasten der Bitterfeldregion mit vielen Ungereimtheiten**

6.1 Machbarkeitsstudie „Schadstoffreduzierung“ im Kerngebiet der Chemieregion – ohne Dioxin

6.2 Unsachgemäße Durchführung einer Unterhaltungsmaßnahme – auch hier fehlt Dioxin!

**7. Untätigkeit der LAF bei dioxinhaltigen Sedimentaltlasten: Verantwortung verweigert**

7.1 Verpflichtungen aus der Machbarkeitsstudie zur Sanierung der Spittelwassersedimente

7.2 Umgang mit mobilen Sedimentaltlasten – ein typischer Fall für das Vorsorgeprinzip

7.3 Wettbewerblicher Dialog „Systemverständnis“ – relevante Erkenntnisse oder Hinhaltetaktik?

**8. Sedimentmaßnahmen im Elberaum: Fortschritte und Defizite im internationalen Vergleich**

8.1 Kriterien zur Auswahl und Priorisierung von Maßnahmen (FGG Elbe 2013)

8.2 Fortschritte im Sedimentmanagementkonzept, Defizite bei Maßnahmen im Elberaum

8.3 Vergleiche mit Maßnahmen bei Dioxin-Altlasten im UK, in Finnland und in den USA

8.4 Maßnahmen für die Binnenelbe – Bergbau, Capping, MNA, Stabilisierung (Literatur)

**9. Prioritäre organische Schadstoffe beim Übergang vom Binnenland in die Nordsee**

**10. Historisch kontaminierte Sedimente unter der WRRL – Rückblick und Ausblick****

**Anlage zu: Dioxin in the Elbe River Basin (Environmental Sciences Europe 2015)**

*****Adresse des Autors

Prof. (i.R.) Dr. Ulrich Förstner
Technische Universität Hamburg-Harburg (TUHH)
Institut für Umwelttechnik und Energiewirtschaft
D-21071 Hamburg
E-mail: [u.foerstner@tu-harburg.de](mailto:u.foerstner@tu-harburg.de)

__________

** Meinem Mentor, dem Sedimentforscher und Umweltgeochemiker Professor Dr. rer.nat. Dr. h.c. mult. German Müller (9.2.1930 – 21.12 2007) in dankbarer Erinnerung gewidmet. Kurzfassung

Die Gegend um Bitterfeld in Sachsen-Anhalt ist die überregional bedeutendste Sekundärquelle für Dioxin im Elbeeinzugsgebiet. Vor allem die extrem dioxinhaltigen Ablagerungen im Spittelwasser galten nach der Wende als labile, erosionsgefährdete Altlasten. Für ihre Sanierung wurde 1993 eine Machbarkeitsstudie in Auftrag gegeben, deren Empfehlungen aber nicht realisiert wurden. Im Gegen­teil – seit 2000 kämpfte die neu installierte Landesanstalt für Altlastenfreistellung in Sachsen-Anhalt (LAF) gegen das Thema „Dioxin“ und das „Source First Prinzip“. Aus der grundsätzlichen Verwei­gerungshaltung entwickelten sich im Zuge der Umsetzung der EU-Wasserrahmenrichtlinie konkrete Aktionen gegen die neuen Schadstoffstrategien im Flusseinzugsgebiet der Elbe.

Zwei Auftragsstudien der LAF und deren Partner GICON aus dem Jahr 2008 behandelten praktische Beispiele von dioxinhaltigen Sedimenten – ohne Dioxine zu nennen: (1) Bei einer Unterhaltungs­maßnahme im Schachtgraben kam es deshalb durch unsachgemäße Methoden zu einer starken Mobi­lisierung von dioxinreichen Schwebstoffen. (2) Ein Maßnahmenpaket für die Rückhaltung von HCH aus der Bitterfelder Kernregion vor dem Eintritt in die Mulde hätte schon viel früher und ganz oben auf der Agenda dieser Landesanstalt stehen müssen. Auch wenn es sich um unveröffentlichte Studien handelt, so sind diese dennoch durch die völlig absurde Nicht-Erwähnung der Bitterfeld-typischen Schadstoffgruppe der Dioxine/Furane drastisch entwertet.

Im Bewirtschaftungsplan für das deutsche Teileinzugsgebiet der Elbe von November 2009 und in der Längsprofilaufnahme 2008 für Dioxine und PCB in Feststoffen (2011) wurden nachträglich wichtige Passagen verändert; die Themen deuten auf Absprachen zwischen LAF und Flussgebietsgemeinschaft Elbe hin. Man muss nach der Auswertung aller verfügbaren Informationen und bei der Würdigung der Entwicklungen seit 2000, die in dem vorliegenden Bericht dokumentiert sind, leider vermuten, dass die Landesanstalt für Altlastenfreistellung von Sachsen-Anhalt und das vorgesetzte Ministerium für Landwirtschaft und Umwelt (MLU) die absehbare Wirkung von Hochwasserereignissen auf die mobilen, extrem dioxinbelasteten Sedimente im Spittelwasser billigend in Kauf genommen haben.

Gegen Ende der Umsetzung des ersten Zyklus der EU-Wasserrahmenrichtlinie im Elbeeinzugsgebiet wurde ein neuer Dioxinfall im Unterlauf der Bode/Saale bekannt. Eine andere Teilbehörde des MLU von Sachsen-Anhalt, der Landesbetrieb für Hochwasserschutz und Wasserwirtschaft, hat dazu alle wissenschaftlich relevanten Daten ins Internet gestellt. Die LAF hält dagegen weiterhin wichtige Informationen über die Dioxinbelastungen in der Kernregion Bitterfeld-Spittelwasser zurück, u.a. zu dem Tauw-Gutachten von 2013, das speziell für die „Ableitung von Maßnahmeerfordernissen“ erstellt worden ist. Die radiometrischen Befunde von Tauw über Dioxinanreicherungen entlang des Spittel­wasser­ufers wurden in deren „Soil Newsletter“ vom Juli 2014 separat veröffentlicht: [http://­medusa-online.com/wp-content/uploads/2014/10/Mapping-sediment-bound-organic-pollutants.pdf](http://medusa-online.com/wp-content/uploads/2014/10/Mapping-sediment-bound-organic-pollutants.pdf) .

Im zweiten Teil der Übersicht über Dioxin und Sedimentaltlasten im Elbeeinzugsgebiet werden drei Themen außerhalb der LAF Problematik behandelt, die für die Umsetzung der Wasserrahmenrichtlinie von Interesse sind: Kapitel 8 „Sedimentmaßnahmen im Elberaum“, Kapitel 9 „Schadstoffe beim Über­gang vom Binnenland in die Nordsee“ und Kapitel 10 „Erkenntnisfortschritte bei Gewässersedimenten vor der Umsetzung des WRRL-Konzepts (Literaturübersicht).

*Kapitel 8 Maßnahmen.* Ein besonderes Verdienst des veröffentlichten Sedimentmanagementkonzeptes der FGG Elbe bzw. der Internationalen Kommission zum Schutz der Elbe (IKSE besteht darin, dass es vorab die Kriterien zur Auswahl und Priorisierung von Maßnahmen festgelegt hat, u.a. die „Priorität bei der Quellsanierung“ (Abschnitt 8.1). Es gibt besondere Defizite bei Maßnahmen im Elberaum, die teilweise durch die besondere Konzentration auf den Hamburger Hafen, aber auch durch aktuelle Hin­halte­taktik der LAF ausgelöst sind (Abschnitt 8.2). Nach der Durchsicht der internationalen Literatur wurden drei Beispiele für Vergleiche mit den Sedimentaltlasten in der Bitterfeld-Region ausgewählt, bei den ebenfalls extreme Dioxinbelastungen zu Beginn der 1990er Jahre bekannt wurden (Abschnitt 8.3). Für den Binnenelberaum kommen in erster Linie Verfahren in Frage, die bereits im Bergbau erprobt wurden und sog. naturnahe Methoden wie Capping, Monitored Natural Attenuation und eine Behandlung mit Aktivkohle (für PCDD, PCBs, Quecksilber, Abschnitt 8.4)

*Kapitel 9 Meeresschutz*. In den kommenden Jahren wird sowohl über die „Erweiterung der prioritären Stoffe“ als auch durch die Meeresstrategie-Richtlinie eine signifikante Aufwertung der Rolle von Dio­xinen/Furanen in diesem kritischen Umweltbereich eintreten. Dem sollten auch die Nordsee-Anlieger mit einem Dioxin-Richtwert in dem „Gemeinsamen Übergangsbestimmungen zum Umgang mit Baggergut in den Küstengewässern“ (GÜBAK 2009, aktualisiert Oktober 2013 Rechnung tragen.

*Kapitel 10 Literaturübersicht für WRRL*. (1) Frühe Entwicklungen in der Wasserforschung und im Gewässerschutz. (2) Frühe Erfahrungen im investigativen Bereich (Beispiel: Neckar und Rhein). (3) Ausrichtung von Verbundforschungsvorhaben 1985 bis 1995. (4) Elbesedimentforschung, vorwiegend Bestandsaufnahmen) in den 1990er Jahren. (5) Erfahrungen mit flächenhaft kontaminierten Feststoffen (Beispiele von der Mittelelbe). (6) Sedimentdynamik und Schadstoffmobilität in Fließgewässern (SEDYMO 2002-2006). Die Schadstoffremobilisierung bei der Erosion historisch kontaminierter Sedimente spielte auch in zwei großen flussgebietsübergreifenden Studien am Rhein (Heise et al. 2004, Auftraggeber: Port of Rotterdam) und an der Elbe (Heise et al. 2005, Hamburg Port Authority) eine dominierende Rolle. (7) Untersuchungen von mobilisierenden Einflussfaktoren (Beispiele aus JSS 2007-2012) zeigten charakteristische Wirkungen von organischen Substanzen auf biostabilisierende Prozesse an Sedimenten durch mikrobielle Anordnungen (Mikrobiologie und Technik). Solche Effekte müssen besonders in Folge der zunehmenden Wahrscheinlichkeit von extremen Hochwässern berück­sichtigt werden.

**Investigations on dioxin and historical contaminated sediments in Saxony-Anhalt**

***Motivation***

Twenty-five years after the German re-unification there is still an open controversy on the dioxin question and the appropriate management of historical contaminated sediments between Saxony-Anhalt and the downstream Federal States like Lower Saxony and Hamburg. With a general agreement in place in 2001, the German Federal Government was acquitted of its obligations by paying a lump sum of one billion EUR and the state of Saxony-Anhalt took over the sole responsibility for the remediation of contaminated sites.

In an article „Sediment research, management and policy“ in the Journal of Soils and Sediments we have informed the inter­national community (Förstner & Salomons 2010): *“Within the common frame of the Large Ecological Project Bitterfeld-Wolfen, the managing agency LAF of Saxony-Anhalt has spent 230 million EUR in the period 2001 to 2010 for ground water sanitation, but any responsibility for sediment issues was rejected“*. The reply of the manager of LAF and his associates from GICON Consultants Company and Centre of Environmental Research Leipzig-Halle (UFZ; Keil et al. 2011) was „*The LAF is far from “rejecting (its) responsibilities” for the financing of sediment management measures when they are necessary, adequate, and appropriate for the elimination of risks to downstream recipients. In summary, we argue that Förstner & Salomons should have been more attentive in their appraisal of scientific facts and management activities accomplished at this site.”*

Meanwhile, a considerable amount of new information on the activities (and non-activities) of the Agency LAF in Saxony-Anhalt has been assembled in the 88-page report (“Materialien”, in German), which will be attached to the forthcoming publication “Dioxin in Historical Contamina­ted Sediments – Elbe River Basin” in the Journal “Environmental Sciences Europe”.

At the beginning of the 2^nd^ WFD cycle, it becomes obvious, that the previous efforts for reducing the pollution load in large parts of the Elbe River Basin is insufficient and the attainment of the “good” ecological and chemical status of suface waters according to the WFD and the “Good Environ­mental Status” with respect to the Marine Strategy Frame­work Directive will be endan­gered. All indicators show that this is mainly due to the presence of historical contaminated sediments.

Hamburg/Buchholz, 28 May 2015

Ulrich Förstner

**1. Altlastensanierung nach der Wende – neue Pflichten gegenüber den Elbe-Unterliegern**

Im Herbst 1990 berichtete „Der Spiegel“ [1] aus Bitterfeld: „*In der Umgebung der Aluminium-Schrott­schmelze wurden Spitzenwerte von 3745 Nanogramm Dioxin pro Kilogramm Boden gefunden* (der aktu­elle Zielwert für uneingeschränkte landwirtschaftliche Nutzung ist 5 ng I-TEQ/kg TM [UF]).  *Greppin ist das am meisten vergiftete Dorf der Welt. Auf der Chemiedeponie „Freiheit III“, einer ehemaligen Braunkohlegrube, liegt der Chemiemüll offen herum und müsste abgetragen werden – Milliarden würde das kosten …“* Aber dann am Ende: *„In der Nacht zum 3. Oktober 1990 verwandeln sich alle Dioxine, alle Chemie- und Braunkohleabfälle, das verseuchte Wasser und die verpestete Luft zur ‘Altlast‘ – und für die ist nicht mehr die arme DDR zuständig, sondern allein das reiche Bonn …“.*

Heinrich Bonnenberg, von 1991 bis 1994 Direktor für Umweltschutz/Altlasten bei der Treuhandanstalt in der ehemaligen DDR, stellte später bei einem Vortrag [2] fest, dass er es als seine vordringlichste Aufgabe empfand *„aktenkundig und über die Presse publik zu machen, dass es sich um örtlich sehr eng eingrenzbare Schäden bei der einschlägigen Industrie handelte, also um vereinzelte hot spots der Belastung von Boden und Grundwasser“.* Dies erklärt, warum von den erwarteten 100 Milliarden DM zur Beherrschung der Altlastenprobleme am Ende der Treuhandphase nur noch 4 Milliarden EURO an Ausgaben angefallen waren (Wismut und Braunkohle wurden separat abgerechnet). Wichtige Projekte – die Dioxinaltlasten gehören sicher dazu – mussten auf einen späteren Zeitpunkt verschoben werden.

In Sachsen-Anhalt gab es dazu eine günstige Gelegenheit, als die Finanzierungsverpflichtungen des Bundes mit dem im Herbst 2001 abgeschlossenen sog. "Generalvertrag" pauschal mit 1 Mrd. EURO abgegolten wurden und das Land damit die alleinige Verantwortung für die Altlastenfinanzierung übernahm [3]. Mit geschickten Bankanlagen und guten Zinsen konnten mittelfristig um die zwei Mrd. EURO zur Verfügung stehen^^[[1]](#footnote-1)^^ – allerdings mit weiteren Pflichten unter der neuen EG-Wasserrahmen­richtlinie, einer flussgebietsübergreifenden Umweltpolitik, deren fünfzehnjährige Umsetzungsphase in 2000 begann [4]. Ein Rechtsgutachten der Freien und Hansestadt Hamburg stellte dazu fest [5]:

*„Aus dem Verfassungsgrundsatz bundesfreundlichen Verhaltens bei der Erfüllung inter- und supra­na­tio­naler Verpflichtungen ergibt sich, dass alle beteiligten Bundesländer innerhalb einer Flussgebiets­gemeinschaft verpflichtet sind, die koordinierte Bewirtschaftung zielführend und effektiv zu betreiben. Nach diesen Grundsätzen hat im Fall der Flussgebietsgemeinschaft Elbe insbesondere die Freie und Hansestadt Hamburg gegen die Oberlieger-Länder (namentlich Sachsen und Sachsen-Anhalt) einen Anspruch auf die Durchführung von Wasserbau- und Sanierungsmaßnahmen, die zur Eliminierung und Reduzierung bestimmter Schadstoffeinträge aus den oberstromigen Risikogebieten sachlich not­wendig sind.“*

Die Forderungen der Unterlieger nach Sanierungsmaßnahmen konzentrierten sich nach der Wende genau auf die oben genannten *Dioxineinträge aus der früheren Leichtmetallproduktion in Bitterfeld.* Ein spezieller Grund dafür war auch, dass über die *Kongenerenmuster der Dioxine/Furane* die Ver­frachtung dieses Extremgiftes mit aufgewirbelten Altsedimenten bis in den Hamburger Hafen [6] und inzwischen bis vor Helgoland zweifelsfrei nachgewiesen werden konnte.

Positive Signale für eine flussgebietsübergreifende Kooperation waren die *Machbarkeitsstudie des Landkreises Bitterfeld* von 1993 [7], die *Bergung von 1.640 t hochkontaminierten Ionenaustauscher­harzen*, bei der die Freie und Hansestadt Hamburg im Jahr 1994 Kosten in Höhe von fast 700.000 DM übernahm [8, 9], die seit 1995 durchgeführten regelmäßigen Gewässer­untersuchungen der Fachbe­hör­den des Landes Sachsen-Anhalt, u.a. auf Dioxine/Furane in den extrem belasteten Schwebstoffen und Sedimenten des Spittelwassers [10, 11], sowie die engagierte Mitarbeit dieser Behörden bei der Vorbereitung des internationalen Fallstudienvergleichs Bitterfeld anlässlich der Tagung ConSoil 2000 in Leipzig [12].

Diese Entwicklung wurde jäh unterbrochen, als im Jahr 2000 die neuinstallierte Landesanstalt für Altlastenfreistellung von Sachsen-Anhalt (LAF) ihre Tätigkeit aufnahm. Mit ihrer demonstrativen Schwerpunktsetzung im traditionellen Grundwasserbereich, begründet durch die Mitwirkung im renommierten EU-WELCOME-Verbund „Megasites“, versuchte die LAF ihre Verantwortung für das neue Fachgebiet „Sedimentaltlasten“ und die damit verbundene Dioxinproblematik abzuwehren. In der Hauptphase der Umsetzung der Wasserrahmenrichtlinie lehnte die LAF den methodischen Ansatz der Risikostudie „feststoffgebundene Schadstoffe in der Elbe“ kategorisch ab und stellte sich gegen die übergreifenden offiziellen Sedimentmanagementkonzepte im Elberaum. Daraus entwickelte sich der hier dokumentierte Fall einer behördlichen Machtdemonstration, mit der eigenmächtigen Veränderung von Berichtspassagen, der Entwertung von Auftragsstudien durch Nichtberücksichtigung der Stoff­­­gruppe Dioxine und dem Einsatz von untauglichen Methoden. Bei dem zentralen Spittelwasserprojekt verlegte man sich aufs Nichtstun – bis ein Extremhochwasser ganze Arbeit leistete.

Inzwischen wurde ein weiterer Dioxinfall im Unterlauf der Bode bekannt (Kasten 1, Seite 15), wäh­­rend Hamburg wieder einmal plant, Sedimente in der Tide-Elbe umzulagern [13]. In den kommenden Jahren wird sowohl über die Erweiterung der prioritären Stoffe [14] als auch durch die Meeresschutz­strategie-Richtlinie [15] eine signifikante Aufwertung der Rolle von Dioxinen/Furanen in diesem kriti­schen Umweltbereich eintreten. Man darf gespannt sein, ob es der neuen Leitung der Landesanstalt für Altlastenfreistellung in Sachsen-Anhalt gelingt, nach 15 Jahren dieses absurden Widerstandes gegen den Geist der Wasserrahmenrichtlinie, eine kooperative Strategie, auch bei der Dioxinproblematik, unter der Flussgebietsgemeinschaft Elbe zu entwickeln.

Der zweite Teil der Übersicht zum Thema „kontaminierte Sedimente im Einzugsgebiet der Elbe“ zeigt die Entwicklung von Sedimentmaßnahmen im Elberaum (Kapitel 8), prioritäre organische Schadstoffe beim Übergang vom Binnenland in die Nordsee (Kapitel 9) und – in chronologischer Abfolge – die Erkenntnisfortschritte bei Gewässersedimenten vor der Umsetzung des WRRL-Konzepts (Kapitel 10):

*(1) Frühe Entwicklungen in der Wasserforschung und im Gewässerschutz.* Die Deutsche Forschungs-gemeinschaft startete 1970 eine systematische Erforschung kontaminierter Sedimente und Schweb-stoffe, parallel zu den Aktivitäten der Bundesanstalt für Gewässerkunde. Die Ergebnisse dieser Arbeiten wurden zur Grundlage für die Überwachungsprogramme der westdeutschen Bundesländer.

*(2) Frühe Erfahrungen im investigativen Bereich (Beispiel: Neckar und Rhein).* Der Haupteinsatz von Sedimentanalysen in der frühen Phase galt dem Aufspüren von Umweltvergehen, d.h. der Einleitung von Schadstoffen, die in der Wasserphase schwer nachzuweisen sind und nur kurzzeitig auftraten.

*(3) Ausrichtung von Verbundforschungsvorhaben 1985 bis 1995.* Ausgehend von den Befunden unter (1) und (2) setzte Mitte der 1980er Jahre die Förderung von sedimentorientierten Verbundforschungs-projekten durch den BMBF und die DFG ein. Deutlich über die Bestandsaufnahmen hinaus ging das nieder¬ländische Forschungsprogamm über Böden und Sedimente als „Chemische Zeitbomben“.

*(4) Elbesedimentforschung, vorwiegend Bestandsaufnahmen, in den 1990er Jahren.* Nach der deut­schen Wiedervereinigung rückte die Sanierung der Elbe als Gemeinschaftsaufgabe von Deutschland und Tschechien in den Vordergrund. Erstmals bestand die Möglichkeit den Istzustand der Schadstoff­belastung der gesamten Elbe mit modernen Methoden systematisch zu erfassen.

*(5) Erfahrungen mit flächenhaft kontaminierten Feststoffen(Beispiele von der Mittelelbe).* Bereits im Vorfeld der EU-Wasserrahmenrichtlinie fanden auch die Sedimentkontaminationen im Ober- und Mittellauf der Flüsse größere Beachtung. Es handelt sich um Überflutungssedimente mit z.T. hohen flächenhaften Schadstoffbelastungen. wie im Niederungsgebiet Mulde/Spittelwasser bei Bitterfeld.

*(6) Sedimentdynamik und Schadstoffmobilität in Fließgewässern (SEDYMO 2002-2006).* Die Schad­stoff­remobilisierung bei der Erosion historisch kontaminierter Sedimente spielte auch in zwei großen flussgebietsübergreifenden Studien am Rhein (Heise et al. 2004, Auftraggeber: Port of Rotterdam) und an der Elbe (Heise et al. 2005, Hamburg Port Authority) eine dominierende Rolle.

*(7) Untersuchungen von mobilisierenden Einflussfaktoren (Beispiele aus JSS 2007-2012)* . Die Unter-suchungen unter AquaTerra, Modelkey und FloodSearch zeigten charakteristische Wirkungen von organischen Substanzen auf biostabilisierende Prozesse an Sedimenten durch mikrobielle Anordnun­gen (Mikrobiologie und Technik). Solche Effekte müssen besonders in Folge der zunehmenden Wahr­scheinlichkeit von extremen Hochwassern berücksichtigt werden.

**2. Herkunft der Dioxin-Altlasten und ihre Verwaltung in den 1990er Jahren**

Die Chemie AG in Bitterfeld und die Filmfabrik Wolfen stellten im Herbst 1991 einen Antrag auf Freistellung von Altlastenkosten und bereits Ende 1992 benannte die Gemeinsame Arbeitsgruppe Bund/Land diese Unternehmen als sogenannte „ökologische Großprojekte“ (ÖGP) [8]:

*„Der ÖGP-Arbeitskreis Bitterfeld-Wolfen umfasste: neben den freigestellten Unternehmen, den sog­e­nannten Projektträgern, die Vertreter der zuständigen Ordnungsbehörde (hier: Landkreis Bitterfeld) und der Fachbehörden (hier: Staatliches Amt für Umweltschutz Dessau/Wittenberg [STAU], Landes­amt für Umweltschutz Halle, Umweltbundesamt) sowie den „Konzepterarbeiter“ (Dr. Jochen Groß­mann, der 1994 die Fa. GICON gründete); letzterer hatte die Aufgabe, Sanierungsstrategien aufzu­stellen und diese durch einen Kostenrahmen zu untersetzen“.*

***2.1 Herkunft der Dioxin-Altlasten in Sachsen-Anhalt [16]***

Zu Beginn des 20. Jahrhunderts war die elektrochemische Industrie u.a. in Bitterfeld eng mit dem Flugzeugbau verknüpft, bedingt durch das bei der Chloralkalielektrolyse als Nebenprodukt anfallende Wasserstoffgas. Seit Anfang der 1930er-Jahre wurde der boomende Industriezweig durch die Leicht­metallproduktion in Bitterfeld sowie in den Tochterwerken Aken und Staßfurt mit den benötigten Leichtmetall-Legierungen beliefert. In dem Herstellungsprozess wurden zunächst die Rohstoffe (u. a. Magnesiumoxid) in Chlorierungsöfen mit Chlorgas umgesetzt, um Magnesiumchlorid für die schmelz­elektrolytische Herstellung des metallischen Magnesiums zu erhalten [17].

***2.2 Überwachung der Dioxin Hot Spots im Spittelwasser durch das STAU D/W [10]***

In den Stellungnahmen des damaligen Staatlichen Amtes für Umweltschutz Dessau/Wittenberg (STAU D/W [11]) wurde abgeschätzt, dass die hochkontaminierten Feinkornsedimente im Spittel­wasserbach bei episodischen Hochwässern von etwa 5jähriger Wiederkehr (HQ_5_) aufgewirbelt werden. Die Ergebnisse der Untersuchungen von Februar und April 2000 bestätigten die Erfahrungen der Jahre 1995 bis 1997, nämlich das Ansteigen der Kontamination in den Ablagerungen des Spittelbachs mit zunehmender Tiefe. In den Teufen unterhalb 30 – 40 cm sind die Dioxinwerte 5- bis 10mal höher als in den oberen Schichten [10]. Eine Reduzierung war sowohl im oberflächennahen Bereich als auch in der Tiefe nicht festzustellen. Spitzen von 1.050 mg/kg Zinnorganika und 140.000 I-TEQ ng/kg für PCDD/PCDF wurden einem 800 m langen Stillwasserbereich im Spittelwasserbach zugeschrieben, der eine Mächtigkeit bis zu ca. 2 m Sediment hatte [10]. Eine Überschlagsrech­nung zeigte, dass mit 5.000 m³ Schlamm aus dem größten und am stärksten belasteten Spittelwasser-Teich – bei geschätzten 20.000 ng TEQ/kg TS an Dioxin im Durchschnitt – 5 Mio. Kubikmeter Elb­sediment auf eine Konzen­tration von 20 ng TEQ/kgTS (*safe sediment value [18]*) kontaminiert werden könnten [19].

***2.3 Machbarkeitsstudie zur Sedimentsanierung des Spittelwassers (1993)* [7]**

Eine wichtige Grundlage bei der Entwicklung eines Sanierungskonzeptes für die „Hot Spot“-Kontami­nationen im Spittelwasserbach war die Erstellung der „modellhaften Machbarkeitsstudie zur Sediment­­sanierung des Spittelwassers im Landkreis Bitterfeld“ vom Juli 1993 im Auftrag des Landratsamtes Bitterfeld, Dezernat Umweltschutz, Naturschutz und Abfallwirtschaft, ausgeführt durch die Ingenieur­gemeinschaft UBS Umweltberatung Schwerin und IGB Verfahrenstechnik Hamburg [7].

Für die kurzfristige und dauerhafte Unterbindung des Austrags schadstoffbelasteter Sedimente aus dem Spittelwassergebiet wurden die in Tabelle 1 aufgeführten technischen Maßnahmen favorisiert.

**Tabelle 1** Schwerpunktbereiche von technischen Maßnahmen zur kurzfristigen und dauer­haften Unterbindung des Austrags schadstoffbelasteter Sedimente aus dem Spittel­wasser­gebiet (nach [7])

| **Technische Maßnahmen zur Minderung des Sedimentaustrags** | | | **Geschätzte Kosten** |
| --- | --- | --- | --- |
| **I** | **Polderung** | Das Niederungsgebiet wird durch Einsatz von Pump­verfahren gezielt entwässert und mit nicht kontaminiertem Material abgedeckt | 40 Mill. EURO |
| **II** | **Sediment- entnahme** | Das Flussbett wird vollständig entschlammt (~20,000 m^3^ stichfester Schlamm). Der Aushub gilt als besonders überwachungsbedürftiger Abfall | *14 Mill. EURO incl. Ausrüstung* |
| **III** | **Sediment-abdeckung** | IIIa: mineralische Deckschicht;  IIIb: künstliche Deckschicht (Geotextil)  - Maßnahme sofort durchführbar; kein Abfallproblem  - Langzeitstabilität nicht gesichert (Morphodynamik) | *IIIa:5 Mill. EURO*  *(100 EURO/m²)*  *IIIb: 3 Mill. EURO*  *(60 EURO/m²)* |
| **IV** | **Fluss- verlegung** | Das Gewässer erhält ein neues Bett; unbelastetes Sediment wird als Abdeckung und zur Verfüllung bzw. Sicherung des alten Flussbetts/Sediments verwendet | *12 Mill. EURO*  *incl. Ausrüstung* |

Bei den Verfahren I, II und IV wird das Naturschutzgebiet stark verändert, bei I sogar vollständig zerstört (durch die Entwässerungsmaßnahme würden außerdem die Grundwasserverhältnisse und die Schadstoffherde erheblich beeinflusst). Bei den Varianten III und IV wäre die Langzeitwirkung auf­grund der stark schwankenden Hydrodynamik schwer abschätzbar; bei III besteht ein hoher Bedarf an unbelastetem Material zur Abdeckung. Bei der Variante IV ist der Verbleib des anfallenden Bagger­gutes abfall- und bodenschutzrechtlich abzusichern. Es besteht hier ein erhebliches Kostenrisiko, da das Volumen der anfallenden kontaminierten Massen nahezu unkalkulierbar ist.

Bei der Variante II, die insgesamt am aussichtsreichsten erscheint, werden neben den kontaminierten Anteilen auch nichtkontaminierte Flusssedimente entfernt, die eine Abdichtwirkung gegenüber dem Grundwasser aufweisen (s.o.). In einer Hausmitteilung vom 15.01.1997 des damaligen Staatlichen Amtes für Umweltschutz Dessau/Wittenberg werden die Aussagen zu den Schwerpunktbereichen der Schlickablagerung nach den Unterlagen von UBS/IGB-VT bestätigt.

Nach der Auswertung der technischen Aspekte schlossen die Gutachter mit folgendem Vorschlag [7]:

*„Eine Kombination aller sinnvollen Verfahrensweisen führt zu 24 verschiedenen Sanierungskonzepten. Sie beruhen auf den Eigenschaften der Sedimente, die überwiegend außer einem sehr großen Wasser­gehalt einen sehr hohen Prozentsatz an Feinkorn und hohe Organikgehalte enthalten. Für die 12 in die engere Wahl zu ziehenden Varianten sind für die Gesamtlösungen zwischen 20 bis 30 Mio. DM an Kosten zu veranschlagen. Nach dieser Abschätzung lagern in dem betrachteten knapp 3 km langen Gewässer­abschnitt rd. 20.000 m³ Schlick mit einem mittleren Trockensubstanzgehalt von 17%. Die Gutachter bevorzugen von den in Frage kommenden Sanierungsvarianten sowohl aus ökologischer, technischer und wirtschaftlicher Sicht die trockene Entnahme und eine Nasstrennung der Sedimente in eine Sand-Kies- und in eine Feinkornfraktion (Korndurchmesser < 0,06 mm) mit anschließendem Waschen der Sande und Kiese bzw. thermischer Behandlung der Feinfraktion (Variante V-6 WV); sie ist mit Kosten in Höhe von 20,9 Mio. DM zu veranschlagen“.*

Ein endgültiges Konzept, das allen Schwierigkeiten der Sedimentsanierung im Sinne der damaligen Aufgabenstellung gerecht werden konnte, war im Rahmen einer kostenlosen Leistung noch nicht zu erwarten. Die Mitwirkung der führenden Firmen spricht aber für das enorme deutschlandweite Inter­esse an dem Standort Bitterfeld und seinen Problemen zur damaligen Zeit [7]^^[[2]](#footnote-2)^^.

**3. Fallstudienvergleich ConSoil2000 und nachfolgendes „dioxinfreies“ EU-Projekt**

Diese und weitere Informationen aus dem Staatlichen Untersuchungsamt Dessau lagen im Jahr 2000 vor, als die Veranstalter des 7th ConSoil Congress Leipzig am Standort Bitterfeld einen internationalen Fallstudienvergleich zur Sanierung großflächiger Altlasten organisierten. Beiträge aus Dänemark, Deutschland, Niederlande und U.K. wurden von den EU-Altlastenorganisationen CLARINET (Conta­minated Land Rehabilitation Network) und NICOLE (Network for Industrially Contaminated Land) begutachtet und bei der Tagung vorgestellt [20]. Bei der Auswahl des Projekts engagierten sich Dr. F. Walkow vom Landkreis Bitterfeld und Prof. Dr. U. Stottmeister vom UFZ Leipzig-Halle.

Der Fallstudien­vergleich in der Spittelwasserniederung bei Bitterfeld befasste sich zum ersten Mal mit der kom­plexen Problematik historisch kontaminierter Sedimente, die durch das fließende Wasser einer ständigen Erosion insbesondere bei Hochwasserereignissen und Umlagerung entlang des gesamten Flusses unterworfen werden. Die Funktionsanalyse derartiger „Altlasten“ war aufgrund fehlender Angaben bisher nicht mög­lich. Zukünftige Maßnahmen müssen jedoch diese Eigenschaften berück­sichtigen.

Nachfolgend werden die Ergebnisse der deutschen und der niederländischen Projektgruppen, die von den Gutachtern besonders hoch eingestuft wurden, ausführlicher dargestellt: der deut­sche Beitrag wegen seiner umfassenden Betrachtung der Bewirtschaftungs- und Organisa­tionspläne, der nieder­ländische Beitrag wegen der Entwicklung systemorientierter integrier­ter Problemlösungen. Zuvor werden die Highlights der britischen und dänischen Beiträge im Original zitiert.

***3.1 Britischer Beitrag [21]***

*River and riverbank.* Experience from the Doe Lea Brook in the UK (*ausführlich im Kapitel 9.3 der vorliegenden Dokumentation*) suggests that dispersion of sediment is considerably more rapid than any biodegradation of dioxin on the sediment partic­les.… In order to mitigate this risk, the contaminated sediment must either be removed or con­tai­ned and treated in situ.

*Woodland.* The woodland areas in the south of the Bitterfeld region and to the north of Jessnitz are probably best treated by use of either MNA, phytoremediation or bioremediation or a combi­nation of these techniques. (1) In areas of low contamination and abundant natural vegetation, active manage­ment of the natural attenuation process and monitoring could be conducted. This could include the encouragement of rapid plant growth. (2) In areas of higher contamination, where trees and plants are currently growing, it may be necessary to use more intensive approaches such as: a) planting specific species of trees which can tolerate high organic and metal contamination and can accumulated these contaminants slowly and: b) using *in situ* bio­remediation, possibly by introducing the white rot fungi *Panerochaete* in wood chips into the surface soils.

***3.2 Dänischer Beitrag [22]***

*Flood control.* A system should be installed in the Spittelwasser and the Mulde junction and could be constructed in different ways. One possible solution is to constructing/re-inforce the dikes along the Mulde and build a higher water sluice at the outlet of the Spittelwasser to prevent backwater from the Mulde and flooding. Upstream the sluice, a settling basin (L=25 m, W=8 m, D=3 m, bank steepness = 1:2) for the particulate matter of the Spittelwasser should be estab­lished. In another solution, the dis­charge can be achieved by constructiong a pipeline from the sluice, parallel to the Mulde, and with outlet 1-2 km downstream; the diameter of the pipeline should be approx. 140 cm.

*Burying and/or diluting contaminated sediment on the floodplain.* If the detailed site characterization confirms that the major dioxin contamination is located within the top 5-15 cm of the soil, the level of contamination in the topsoil can be significantly reduced by ploughing to 0.7 to 0.8 meter below grade. This kind of ploughing requires special equipment. After ploughing, surface contamination can be further reduced by moulding (dilution).

***3.3 Niederländischer Beitrag [23] – Schwerpunkt „Behandlungsalternativen“***

Für den Fallstudienvergleich hat die niederländische Projektgruppe verschiedene Alternativen für die Sanierung des Niederungsgebiets vorgeschlagen, die allerdings die drei Problem­bereiche nicht so strikt trennt wie das beispielsweise bei dem deutschen Beitrag der Fall ist. Die Kosten sind der Tabelle 2 zu entnehmen.

Die Alternative 1 begründet sich durch die Anregung von natürlichen Prozessen. Es handelt sich um eine Kombination von Grundwassersammlung in den natürlichen Wasserläufen des „Buschwaldes“ und einem System von Hydro-Capping. Letzteres besteht aus Bitterfeld-Ton, der mit Flusswasser verdünnt und bei Hochwasser in das Spittelwasser und vor dort in die Über­flutungsbereiche ausgespült wird. Die kontaminierte Fläche ist etwa 200 Hektar groß. Bei der Gesamtmenge von 250.000 m³ würde innerhalb von fünf Jahren eine Abdeckung von 0,10 bis 0,15 m entstehen, wobei entsprechend den morphologischen Verhältnissen die kontaminierten Stellen am stärksten von der Maßnahme profitieren würden.

**Tabelle 2** Darstellung der Kosten der Alternativen 1 bis 3 im niederländischen Vorschlag [23]

| **Aktivitäten** | **Alternative 1** | **Alternative 2** | **Alternative 3** |
| --- | --- | --- | --- |
| Grundwassersammlung | 1.500.000 € |  |  |
| Bio-Sammelbecken – 2 Einheiten |  | 5.000.000 € | 5.000.000 € |
| Hydro-Capping | 3.500.000 € |  |  |
| Neue Oberflächenschicht |  | 15.000.000 € |  |
| Vollständige Sanierung |  |  | 100.000.000 € |
| Hydrologische Bewirtschaftung | 6.000.000 € | 6.000.000 € | 6.000.000 € |
| Rückgewinnung von Ackerland | 10.500.000 € | 10.500.000 € |  |
| Forschung + Monitoringprogramm | 14.000.000 € | 14.000.000 € | 7.500.000 € |
| *Summe* | *35.500.000 €* | *50.500.000 €* | *118.500.000 €* |

Alternative 2 ist ein stärker technisch beeinflusster Ansatz, bei dem Bio-Becken ausgegraben werden und eine Lage sauberer Feststoffe auf den kontaminierten Sedimenten aufgebracht wird. In Anlehnung an die Tradition des Braunkohletagebaus sollen zwei Becken mit einer Fläche von ca. 1 Hektar und einer Tiefe von 20 m erstellt werden. Darin wird eine Wasser­strömung wie in einem (Bio-)Karussell geführt und der Abbau der Restkontaminanten in dem oberflächennahen Grundwasser wird durch Zu­gabe von Nährstoffen und die aeroben Bedin­gungen gefördert. Das Ausmaß der Bio-Becken erfordert zusätzliche hydrologische Berech­nungen und Abbauversuche im Pilotmaßstab.

Die 3. Alternative beinhaltet ein Ausgraben des gesamten kontaminierten Auenbodens bis in eine Tiefe von 0,5 m. Das Material muss auf einer Sonderabfalldeponie abgelagert werden.

***3.4 Deutscher Beitrag [24] – Schwerpunkt „Organisation“***

Der *deutsche Beitrag* zeigte auf, dass im Unterschied zur konventionellen Altlastenproblematik die Risiken aus dem Niederungsgebiet Spittelwasser vor allem in der Verfrachtung und Ablagerung von kontaminierten Feststoffen innerhalb eines Flusseinzugsgebiets liegen.

Vor dem Hintergrund der angestrebten nachhaltigen Entwicklung des Gebietes und der bestehenden Erfolgsrisiken sowie unter dem Aspekt, dass die Maßnahmen dem Grundsatz der Verhältnismäßigkeit entsprechen sollen, erschien es sinnvoll, die in Tabelle 3 dargestellten Einzelmaßnahmen zu kombinie­ren und schrittweise umzusetzen. Im Mittelpunkt des Regulierungsprojektes (Teilschritt 02) standen die Förderung des Pflanzenwachstums zur mechanischen Stabilisierung der Sedimente und die Anwen­dung von Prozessen des natürlichen Schadstoffrückhalts- und -abbaus [24].

Das Interesse an *kontrollierten natürlichen Schadstoffminderungsprozessen* hat deutlich zugenommen, seit diese bei der Bewertung von (Neu-)Schadensfällen berücksichtigt werden müssen. So sieht die aktuelle EU-Umwelthaftungsrichtlinie (EU-UmwHaftRL [25]), die durch das Umweltschadensgesetz [26] in nationales Recht umgesetzt wurde [27], ausdrücklich die Option „natural recovery“ vor. Der Einsatz von Natural Attenuation in Flussauenböden und -sedimenten wurde im Überflutungsbereich des Spittelwassers untersucht [28] und die Resultate gingen in den Leitfaden „Natürliche Schadstoff­minderungsprozesse an großräumigen Bergbaukippen/-halden und Flussauensedimenten“ [29] ein.

Erforderlich ist eine zielgerichtete *Kompetenzbündelung* durch Zusammenstellung eines Pro­jekt­­teams, dem erfahrene Fachleute der Arbeitsbereiche Recht, Planung, Technik, Controlling und Öffentlich­keitsarbeit angehören. Auf Behördenseite waren das Staatliche Amt für Umwelt Dessau-Wittenberg als fachtechnische Überwachungsbehörde und das Regierungspräsidium Dessau als Aufsichtsbehörde in die Vorbereitung des Projektes einzubeziehen.

Für die fachliche Beratung und die sachkundige Evaluation vorliegender Ergebnisse sollten das betroffene Landesamt für Umweltschutz Sachsen-Anhalt und die zuständige Biosphären­reser­vats­verwaltung „Mittlere Elbe“ Dessau in die Projektdurchführung einbezogen werden.

Die Zusammenarbeit der Projektbeteiligten sollte auf einem abgestimmten Projektstrukturplan basie­ren, der die Gliederung des Projektes in Unterprojekte, Phasen, Aktivitäten, Termin, Kos­ten etc. um­fasst und die Grundlage für das zentrale Projektmanagement mit seinen Planungs-, Steuerungs- und Koordinierungsaufgaben bildet.

In die Organisationsstruktur ist als zentraler Ansprechpartner ein entscheidungsbefugter Ver­treter der zuständigen Behörde einzubeziehen, der die weitere Koordinierung mit den zustän­digen Fachreferaten innerhalb der Behörden übernimmt.

**Tabelle 3** Projektvorschlag der deutschen Arbeitsgruppe [24]

|  | | | **Geschätzte Kosten/Zeit** |
| --- | --- | --- | --- |
| **1** | **Monitoring-system** | Das strömungsabhängige Schadstofftransport­verhalten soll mit hydrodynamischen und luftgestützten Methoden überwacht werden | *400.000 EURO*  *1. bis 48. Monat* |
| **2** | **Regulierungs-projekt** | (1) Einsatz von Modellen zum Sediment- und Schadstofftransport  (2) Einrichtung von Sedimentfallen; punktuelle Sedimententnahme  (3) Nutzung von “Natural Attenuation”; Stabilisierung mit Pflanzen | *Projekte (1) + (3) 530.000 EURO*  *12. bis 30. Monat* |
| **3** | **Erprobung** | Betrifft vor allem die Funktionsfähigkeit und Wirkung der Sedimentfallen. Datenerhebung für die Prognose des Schadstoffaustrags | *250.000 EURO*  *30. bis 40. Monat* |
| **4** | **Dauerbetrieb** | Erfolgskontrolle des Gesamtkonzeptes, z.B. in einem GIS | *770.000 EURO*  *24. bis 48. Monat* |
| **5** | **Nachsorge** | Die begonnenen Untersuchungen sind konti­nuierlich und langfristig durchzuführen (nach Beispiel anderer Dauerbeobachtungsflächen) | *225.000 EURO*  *(15,000 EURO/a ~ 15 Jahre)* |

Der Organisator des deutschen Projekts, Dipl.-Ing. Uwe Wittmann vom BMBF-Projektträger Jülich für Abfallwirtschaft und Altlasten berichtete über die Pläne zur Weiterführung der Projekte aus dem Fall­studienvergleich:

*„Aus Sicht des PT ist der Ländervergleich sehr gelungen, verdeutlicht dieser doch erstmals in kompak­ter Form die unterschiedlichen Herangehensweisen der Länder. Bereits während der Präsentation aber auch beim Pressegespräch wurde jedoch insbesondere durch die Beiträge der Holländer deutlich, dass nicht der Ländervergleich im Vordergrund stand sondern die Absicht, ein inter­nationales Projekt in Bitterfeld unter der Leitung der Holländer zu installieren. Am Rande der Konferenz informierte der Ministerialrat* [zuständig für Altlastensanierung im Umweltministerium Sachsen-Anhalt] *den Unterzeichner über die vom Land beabsichtigte Vorgehensweise: sie beinhaltet die Vorbereitung eines Projektes, das sich an den deutschen Vorschlag anlehnt und in das Ökologische Großprojekt eingebunden werden soll. Hierzu müssen im Umweltministerium noch entsprechende Abstimmungen erfolgen …“* (Berlin, den 28.09.2000)*.*

***3.5 Weiterführung des Niederländischen Beitrags im EU-Projekt „WELCOME“ (1/2001-12/2003)***

Der Fall „Bitterfeld/Spittelwasser“ hatte bei der *niederländischen Arbeitsgruppe* ein weiterreichendes Interesse gefunden (Teamleiter Bert Satijn: *„Es gibt noch viele Bitterfelds vor allem in Osteuropa“*). Nicht zuletzt deshalb hatte sich das niederländische Team einen großen Teil seiner Projektarbeit vom Centre for Soil Quality, Management and Knowledge Transfer, sponsern lassen [30].

Aufbauend auf diesen Erfahrungen wurde vom *TNO Institute of Energy, Environment und Process Innovation* das EU-FP5-Forschungsvorhaben *WELCOME* initiiert, koordiniert und von 1/2001 bis 12/2003 an den drei Standorten Bitterfeld, Rotterdam/Antwerpen und Tarnowskie Góry (PL) in 13 Teilprojekten durchgeführt [30]. *Bitterfeld* stand für den Schwerpunkt „Grundwasser“ und speziell für Sanierungsstrategien bei Altlastenprojekten mit großräumigen Grundwasserschäden; federführend waren das Umweltforschungszentrum Leipzig-Halle, die GICON Ingenieur Consult GmbH und die Landesanstalt für Altlastenfreistellung Sachsen-Anhalt [31].

Ausblick auf Kapitel 4

Im Herbst 2000 wurde der Altlastenbereich in Sachsen-Anhalt umgestaltet. Bei der einseitigen Fixie­rung der neuen Landesanstalt für Altlastenfreistellung auf den Grundwasserbereich war klar, dass sich das o.g. *Projekt nicht an den deutschen Vorschlag anlehnen* würde^^[[3]](#footnote-3)^^. Die Begründung, dass man Rotterdam wegen dort nur geringer Dioxinkonzentrationen für das Teilprojekt „Prioritäre Stoffe in Sedimenten von Megasites“ ausgewählt habe [32], erscheint weit hergeholt. Diese Zuordnung lässt sich aber damit erklären, dass es für das deutsche Bitterfeld-Konsortium entscheidend war; künftig nichts mit Dioxin und Sedimentaltlasten zu tun zu haben.

**Kasten 1 Sediments and the European Water Framework Directive (**Förstner JSS 2(2002)54

The very successful installation of the 'Demand-driven European Sediment Research Network (SedNet)' [i] provides a stable common platform for both researchers and practitioners in this field. Demand-driven? Surely, demand within a new market first has to be developed. Until now, very few 'problem owners' face a large majority of potential 'solvers', as has been indicated from an inquiry among SedNet participants. At first, 'harmonization' of sediment quality objectives and management performance is needed for both the European river basins and with respect to the most sensitive areas within these basins – floodplains, estuaries and coastal zones. Distinctions such as 'problem owners' and 'problem solvers' should then become obsolete.

The self-declared problem owners to date are mostly located at the mouth of the large rivers and they are in a rather uncomfortable situation as they have to pay the expenses for all former, actual and future shortcomings in the emission control within their catchment area. According to available information, there should be many more 'interim owners' of sediment problems in the upstream river basin. Many of them, however, ignore their problems or claim to follow a procedure called 'sediment relocation'; among the latter, the problems for the management by the 'end owners' can be aggravated further.

In fact, it is not really a transfer of contaminated sediment to its original site, but rather a down-locating (as re-cycling of waste materials mostly is a down-cycling), and with the dispersion of pollutants there is an unecological increase of entropy. Although the large-scale effect of natural and technical resuspension processes is well-known – for example, in the Elbe River typical patterns of dioxin congeners from the Bitterfeld area can be detected in the sediments of the Port of Hamburg more than 300 km downstream – sediment problems in river basins are still regulated locally, sometimes by means of dubious threshold values.

Here, a clear deficiency of the European Water Framework Directive (WFD) becomes evident. The WFD aims at achieving a good ecological potential and good surface water chemical status in European river basins until the year 2015 by a combined approach using emission and pollutant standards. These consider priority pollutants from diffuse and point sources, but neglect the role of sediments as a long-term secondary source of contaminants. Such a lack of information may easily lead to unreliable risk analyses with respect to the – pretended – 'good status'.

The requirements for a river basin-wide sediment concept will be even more challenging than the actual Water Framework Directive. It will include inventories of interim depots within the catchment area (underground and surficial mining residues, river-dams, lock-reservoirs), integrated studies on hydromechanical, biological and geochemistry processes, risk assessments on sedimentary biocoenoses and, last but not least, development of decision tools for sustainable technical measures on a river basin scale including sediment aspects. Many of these could be promising themes for interdisciplinary research in the European Commission's 6^th^ Framework Program.

**Kommentar zu den Entwürfen des Bewirtschaftungsplans und des Maßnahmen­programms gemäß Artikel 13 bzw. 11 WRRL für den deutschen Teil der Flussgebietseinheit (**Förstner 2009)

Während die sedimentrelevanten Konzepte im Bewirtschaftungsplan der FGG Elbe überwiegend dem Stand der wissenschaftlichen Diskussionen in der EU entsprechen, spiegeln die dazugehörigen Maßnahmenprogramme noch immer die Einstellung weiter Kreise der europäischen Wasserpolitik wieder, die der Sedimentthematik keine Bedeutung bei der Umsetzung der Wasserrahmenrichtlinie beimessen wollen. So enthalten weder der Umweltbericht zum Entwurf des Maßnahmenprogramms gemäß Art. 11 der WRRL für die FGG Elbe noch der standardisierte Maßnahmenkatalog der Bund/Länder-Arbeitsgemeinschaft Wasser (LAWA) mit seinen 99 umsetzungsbezogenen und 8 konzeptionellen Maßnahmen Hinweise auf die praktische Vorgehensweise bei Sedimentaltlasten („historisch kontaminierte Sedimente“). *(Umweltwiss Schadstoff Forschung 21 [2009]:323)*

**4. Sachsen-Anhalt sucht seine Rollen unter der WRRL und in der FGG Elbe (2005)**

Es deutet alles darauf hin, dass das Land Sachsen-Anhalt mit der Einrichtung der Landesanstalt für Altlastenfreistellung (LAF) eine organisatorische Struktur schaffen wollte, um im Zweifelsfall unab­hängig von den Verpflichtungen aus der Wasserrahmenrichtlinie handeln zu können. Angesichts der bei der LAF bereits akkumulierten Finanzmittel wurde die bisherige Bündelungsfunktion durch das Regierungspräsidium Dessau für den Altlastenbereich auf die LAF in Magdeburg übertragen; noch einschneidender war es, dass nach dem Abschluss der Vereinbarung zwischen der Bundesanstalt für vereinigungsbedingten Sonderaufgaben (BvS) und dem Land Sachsen-Anhalt zur sog. „Pau­schalisie­rung“ der Finanzierungsverpflichtungen die LAF praktisch die gesamte Finanzverantwortung für den Altlastensektor in diesem Bundesland übernehmen konnte [8]. Für die Flussgebietsgemein­schaft Elbe, die bei der Formulierung ihres ersten Bewirtschaftungsplans nach der EU-Wasserrahmenrichtlinie (WRRL) auf viel Konsensbereitschaft seitens der Ländervertreter angewiesen war, bedeutete der Auf­tritt dieses potenten „Außenseiters“ eine zusätzliche Belastung.

Zwischen 2000 und 2005 entwickelte sich die Landesanstalt für Altlastenfreistellung zu einer festen Größe in der Umweltpolitik von Sachsen-Anhalt und auch die mitteldeutschen Umwelt-Consulting­firmen wie GICON und deren Partner suchten die Nähe zu der finanzkräftigen Anstalt. Von den Mitteln des Bundes sind zwischen 2001 und 2009 über 200 Mio. € in das Ökologische Großprojekt Bitterfeld-Wolfen geflossen, bis 2006 fast ausschließlich in den Grundwasserbereich. Dazu gehörten unter anderen [33]:

- kritische Belastung durch: BTEX, LHKW, Chlorbenzol, HCH, Chloraniline, Chlorphenole
- seit 2006 hydraulische Abstromsicherung mit Reinigungsanlagen für kontaminiertes Wasser
- seit 2007 Quellensanierungsmaßnahmen
- ab 2009 „Natural Attenuation“ (d.h. stimulierte Selbstreinigung) mit entsprechendem Monitoring

***Umsetzung der EU-Wasserrahmenrichtlinie in Sachsen-Anhalt: Ministerium für Landwirtschaft und Umwelt und LAF gehen eigene Wege***

Am 9. Juni 2005 fand in Magdeburg eine Tagung der Landesanstalt für Altlastenfreistellung und des Ingenieurtechnischen Verbands Altlasten (ITVA) zum Thema „Freistellungsfinanzierte Altlasten-sanierung – Neue Herausforderungen in fachlicher, administrativer und finanzplanerischer Hinsicht“ statt [34]. In ihrem Grußwort stellte die damalige Umweltministerin Petra Wernicke die gewachsenen Verbindungen zur EU heraus: „Die europäische Diskussion zur Wasserrahmenrichtlinie ist in das EU-Projekt WELCOME eingeflossen, das der Arbeit der LAF wichtige Impulse gegeben hat“ [35]. Über die Fortschritte berichteten die Vorträge der maßgeblichen Repräsentanten des WELCOME-Projekts:

- „Sanierung industrieller Großstandorte unter Geltung der Wasserrahmenrichtlinie“ von Dr. Jochen Großmann (GICON GmbH) am Beispiel Bitterfeld [36],
- „Das IMS – Managementsystem zur strukturierten Entwicklung von Sanierungsszenarien“ von Dr. Huub H.M. Rijnaarts (TNO, Niederlande) am Beispiel Hafen Rotterdam [37] .

Rückblickend fällt auf, dass die beiden Autoren trotz dieser klassischen Standortbeispiele zwei nahe liegende Begriffe konsequent vermieden haben: beim Thema „EU-WRRL“ den Begriff „Oberflächen-wässer“, und beim Thema „Altlasten Rotterdam-Hafen“ den Begriff „Sediment bzw. Baggergut“. Es waren genau die Begriffe unerwünscht, mit denen LAF und GICON in der entscheidenden Phase der WRRL-Umsetzung, der Erstellung des Bewirtschaftungsplans für das deutsche Einzugsgebiet der Elbe, eine eigene Expertise für *Sedimentaltlasten* hätten ausweisen können. Bei den Zuständigkeiten für die Umsetzung der WRRL wurde der Eindruck erweckt, als sei aus der Sicht des Ministeriums für Landwirtschaft und Umwelt in Sachsen-Anhalt bereits in 2005 alles geklärt [38]:

*„Zur Umsetzung der Wasserrahmenrichtlinie in Sachsen-Anhalt wurde eine Projektorganisation auf-gebaut. Diese Projektorganisation trifft Festlegungen wer bei uns im Land zu dem Thema WRRL was macht. Die Gesamtprojektleitung einschließlich der Gesamtkoordination obliegt dem Ministerium für Landwirtschaft und Umwelt des Landes Sachsen-Anhalt. Eingebunden sind als weitere Behörden das Landesverwaltungsamt und der Landesbetrieb für Hochwasserschutz und Wasserwirtschaft (LHW)“.*

***Dioxin im Fokus des Landesbetriebs für Hochwasserschutz und Wasserwirtschaft***

Bereits damals stand jedoch der LHW [39] mit seiner fachlichen Kompetenz in der Dioxinproblematik eindeutig hinter den überregionalen Bewirtschaftungszielen der Flussgebietsgemeinschaft Elbe:

Ergebnisse aus dem Bericht vom Mai 2010 [39] zeigen, dass durch den Zufluss des Spittelwassers mit 750 (2006) bis ca. 1000 ng I-TEQ/kg (2007) Dioxin in schwebstoffbürtigen Sedimenten an der Station Mulde/Dessau verglichen mit der oberhalb gelegenen Muldemessstelle Bad Düben ein deutlicher Kon­­zentrations­anstieg von 12 auf im Mittel 85 ng I-TEQ/kg erfolgt. Die Gegenüberstellung der Daten der Elbe an den Stationen Dommitzsch und Magdeburg (Kasten 2) ergibt, dass durch den Einfluss der dioxinbelasteten Nebengewässer Saale und Mulde ein Anstieg der Dioxingehalte in der Elbe von 12 auf 91 ng I-TEQ/kg im Jahr 2006 und von 8 auf 55 ng I-TEQ im Jahr 2007 zu verzeichnen war.

In Übereinstimmung mit dem Ansatz der ad hoc Arbeitsgruppe Schadstoffe der FGG Elbe kam der LHW [39] zu dem Fazit: *„Der für das Jahr 2007 ermittelte Jahresdurchschnittswert im Spittelwasser entspricht dem etwa 275-fachen der Zielvorgabe von 3,75 I-TEQ ng/kg Futtermittel. Auch für die Elbe und Mulde ist die Ziel­vorgabe für Futtermittel hinsichtlich aller ermittelter Werte, selbst der Minima­l­werte an allen Stellen und überwiegend deutlich überschritten“*.

**Kasten 2: Dioxinbelastung der Elbe und ihrer Zuflüsse in Sachsen-Anhalt 2006/07 (LHW** [**39**]**)**

In die Umwelt freige­setzt, besitzen Dioxine Halbwertszeiten von bis zu 100 Jahren. Zu­nehmend tritt die Belastung der Elbe durch PCDD/F in Blickpunkt des Meeresschutzes und Dioxine zäh­len zu den Stoffen, deren Eintrag mittelfristig gänzlich unterbunden werden soll^^[[4]](#footnote-4)^^. Es wurde errechnet, dass durch die Flutwirkung des Extremhochwassers vom August 2002 im Vordeichsgelände der Elbe­aue bis zum Wehr Geesthacht landseitig schätzungsweise 4,3 bis 6,3 g Toxizitätsäquivalente (TEQ-WHO) Dioxin an Schweb­stoff gebunden abgelagert wurden; zwischen 3,1 und 4,6 g WHO-TEQ sollen danach über das Wehr Geest­hacht mit dem Tidestrom in Richtung Nordsee transportiert worden sein^^[[5]](#footnote-5)^^.

Gemäß EG-Wasserrahmenrichtlinie (WRRL) sind für das Erreichen eines guten Zustandes der Ober­flächenwasserkörper Umweltqualitätsnormen für eine Vielzahl von Schadstoffen einzuhalten. In der Richtlinie 2008/105/EG vom 16.12.08 sind die Dioxine im Anhang III als Stoffe aufgeführt, die einer Überprüfung zur möglichen Einstufung als „prioritäre Stoffe“ oder „prioritär gefährliche Stoffe“ zu unter­ziehen sind. Die Flussgebietsgemeinschaft (FGG) Elbe hat überregionale Bewirtschaftungsziele für den Belastungsschwerpunkt Schadstoffe festgelegt. Neben anderen Schadstoffen wurden auch die Dioxine als Schadstoffe mit überregionaler Bedeutung in der FGG Elbe identifiziert. Die sich daraus ergebenden Reduzierungsanforderungen für Dioxine betragen für die Mulde 97 % (siehe unten).

Die Dioxinbelastung von Spittelwasser und Mulde wird vom Landesbetrieb für Hoch­was­ser­schutz und Wasserwirtschaft Sachsen-Anhalt überwacht. Ergebnisse aus dem Bericht vom Mai 2010 werden in den Tabellen 4 und 5 zusammengefasst. Tabelle 4 zeigt, dass durch den Zufluss des stark mit PCDD/F belasteten Spittelwassers an der Station Mulde/Dessau verglichen mit der oberhalb gelege­nen Mulde­messstelle Bad Düben^^[[6]](#footnote-6)^^ ein deutlicher Konzentrationsanstieg auf im Mittel 127 I-TEQ ng/kg erfolgt. Auch die Kon­generenverteilung lässt diese Überprägung der Mulde vom Ober- zum Unter­lauf deutlich nach­vollziehen^6^; Ähnlichkeiten in der Verteilung der Kongenere sind von der Leine über die Mulde/­Jeßnitz (beide Stationen sind in Tab. 5 nicht dargestellt) bis zur Messstelle Mulde/Dessau erkennbar.

|  | 2006 | 2007 |
| --- | --- | --- |
| Elbe  Dommitzsch | 11,9  *(2)* | 8,0  *(1)* |
| Mulde  Dessau | 56,1  *(4)* | 126,6  *(4)* |
| Saale  Gr Rosenburg | 60  *(4)* | 94,4  *(4)* |
| Elbe  Magdeburg | 91  *(3)* | 54,5  *(3)* |

Tabelle 5 wie Tabelle 4, Proben von AMB *(Anzahl der Datensätze)*

|  | Mulde (Bad D.) | | Spittelwasser | | Mulde (Dessau) | |
| --- | --- | --- | --- | --- | --- | --- |
| Jahr | ø | min/max | ø | min/max | ø | min/max |
| 2006 | 11 | 10/12 | 741 | 445/1052 | 56,1 | 16,3/81,8 |
| 2007 | 12 | 11/13 | 1032 | 583/1369 | 127 | 96,4/167 |

Tabelle 4 Σ PCDD/F in I-TEQ ng/kg in schwebstoffbürtigen Sedimenten 2006/2007. Stationen Mulde (Bad Düben, Dessau) Monatsmischproben an den Automatischen Messsstationen Beschaffenheit (AMB), Spittelwasser unterhalb Schachtgraben: Schwebstoffkasten Sammelbehälter Monatsmischproben. Ein­zelproben für Mittelwerte 2006/2007: BD: 2/2; Sp:6/3; De: 6/4.

In der Tabelle 5 wird die Beeinflussung der Beschaffenheit der Elbe durch Mulde und Saale gezeigt. Die Gegenüberstellung der Daten der Elbe AMB Dommitzsch und Magdeburg ergibt, dass durch den Einfluss der dioxinbelasteten Nebengewässer ein Anstieg der Dioxingehalte in der Elbe um das 7,6-fache im Jahr 2006 und um das 6,8-fache im Jahr 2007 zu verzeichnen war.

**5. Landesanstalt für Altlastenfreistellung gegen das „Source First-Prinzip“ und „Dioxin“**

Am 29.12.2005 gab *„Der Spiegel“* ein Zwischenfazit über die Situation in Bitterfeld [40] aus Anlass einer Warnung des Umweltbundesamtes vor dem Verzehr von HCH-kontaminierten Fischen [41]:

1) „Seit einer ersten Bestandsaufnahme 1991 gelten 20 000 Kubikmeter Schlick in der *Spittelwasser-Aue* als extrem kontaminiert; wahrscheinlich sind große Mengen dieses Sediments mit der Flut 2002 über die Mulde in die Elbe gelangt“; „immer wieder Versuche, die Altlast in den Griff zu bekommen“:

2) „Das Landratsamt Bitterfeld ließ 1993 eine *Machbarkeitsstudie zur Sanierung* [7] anfertigen – für 20 bis 30 Millionen Mark, so die damalige Schätzung, wäre die Aue zu retten. Doch das Regierungs­präsidium legte sich quer, die Studie blieb unter Verschluss ….“

3) „Im Jahr 2000 erarbeitete ein *internationales Expertenkonsortium* fünf Projektvorschläge [20], deren Umsetzung zwischen 6 und 40 Millionen Euro gekostet hätte. Beim billigsten Projekt wäre der Fluss begradigt und das alte Sediment abgedeckt worden – doch passiert ist erneut nichts ….“

4) „In 2004 gab die Arbeitsgemeinschaft für die Reinhaltung der Elbe erneut eine *"Empfehlung zur Sedimentsanierung des Spittelwassers"* [40] ab. Innerhalb von zwei Monaten könnten Hydraulikbagger die Fläche abtragen und Lkws das Sediment auf eine *Sonderdeponie* bringen. Für grob geschätzt fünf Millionen Euro ….“ „Das Projekt verlief wie immer im Sande ….“.

Interessant war, dass „Der Spiegel“ in diesem Beitrag niemals den Begriff *„Dioxin“* benutzte. Sollte es der LAF, der Flussgebietsgemeinschaft Elbe oder dem Umwelt­forschungszentrum gelungen sein, dem Nachrichtenmagazin dieses „Unwort“ auszureden …?

Nach 2007 wurde die Gangart der LAF gegen die Autoren der beiden HPA-Elbestudien [42, 43] rauer und obwohl das Thema „Dioxin“ zum Tagesgeschäft gehörte [44-50], entzündete sich an dieser Stoffgruppe bei der Risikostudie „Feststoffgebundene Schadstoffe im Elbeeinzugsgebiet“ [42] ein Dissens (Abschnitt 5.1), der zu Manipulationen am Bewirtschaftungsplan der FGG Elbe ([51] „Dioxin“, Abschnitt 5.2) und an der FGG-gesponserten Version der Publikation „Dioxin Längs­profilaufnahme 2008“ ([52 ]„Source First“, Abschnitt 5.3) führte.

***5.1 LAF-Kritik an der HPA-Studie: „Keine Rücksicht auf sensible Situation in Sachsen-Anhalt“***

Mitte Oktober 2007 haben das Ministerium für Landwirtschaft und Umwelt und die LAFvon Sachsen-Anhalt unabhängig voneinander ihre Stellungnahmen zum Entwurf der HPA Elbe-Risikostudie [42] abgegeben. Während die durchweg konstruktiven Hinweise des MLU zügig in die Endfassung der Studie eingearbeitet werden konnten, lautete das Fazit der LAF:

*„Die Schwäche des Berichtes besteht insbesondere darin, dass hier gefordert wird, was als Ergebnis einer vergleichenden Bewertung abzuleiten wäre… Von einer hohen Konzentration allein und direkt auf Wirkungen bzw. Risiken in räumlich weit entfernten Gebieten zu schließen und hieraus abzuleiten, welche Maßnahmen sinnvoller Weise zu ergreifen sind, ist nicht zulässig und bedarf einer umfassen­den Begründung …. Die LAF hält den Bericht in der gegenwärtigen Form nicht für akzeptabel!“*

In ihrer Antwort vom 16.11.2007 stellten die Autoren der Risikostudie [42] klar, dass die von LAF geforderten Frachtbilanzen bereits in dem bestehenden Netz von Messstationen für die Herleitung überregionaler Handlungsziele erfasst wurden. Eine deutlich größere Detaillierung würde aber einen unverhältnismäßigen Aufwand bedeuten und letztlich evtl. notwendige Sofortmaßnahmen verhindern:

*„Frachtberechnungen erfolgten grundsätzlich an Messstellen, für die neben der Konzentration auch die Abflüsse bekannt waren. Das Bild ist mit Ausnahme der unteren Elbe einheitlich und robust. Kleinskalige und höher aufgelöste Daten würden daran nichts ändern, sondern a) die finanziellen Aufwendungen in die Höhe treiben und b) die Diskussion um Maßnahmen verzögern“*.

Am 21. November 2007 kam es zu einem Gespräch zwischen LAF/GICON und der Erstautorin der Risikostudie im Beisein des Leiters der Ad hoc Gruppe „Sedimentmanagementkonzept Elbe“. Die LAF/GICON hatte das Dioxin-Thema inzwischen auf eine staatspolitische Dimension angehoben und forderte nun eine *„Berücksichtigung der sensiblen Situation in Sachsen-Anhalt“*. Diese Einschätzung einer besonderen Lage in diesem Bundesland fand keine Zustimmung und so blieb die Risiko-Studie „Feststoffgebundene Schadstoffe im Elbeeinzugsgebiet“ [42] ein wissenschaftlicher Bericht – ohne Logo der FGG Elbe (siehe Abschnitte 5.2 und 5.3).

***5.2 Bewirtschaftungsplan Elbe: FGG Elbe entzieht Dioxin den Prioritätsstatus im ÖGP Bitterfeld***

Bei der Erarbeitung des flussgebietsbezogen Bewirtschaftungsplans wurde die Kontroverse um das Thema „Dioxin“ ins Groteske getrieben. Ausgangspunkt war das ambitionierte *Hintergrundpapier der FGG Elbe* [53] zur überregionalen Ableitung von Bewirtschaftungszielen: Danach müssen die Frach­ten von HCH und Dioxin aus der Mulde am stärksten reduziert werden, um die Zielvorgaben bzw. Qualitäts­normen der vorrangig betrachteten Umweltbereiche einhalten zu können:

- 99 Prozent für β-HCH – Schutz der menschlichen Gesundheit vor den Folgen des Verzehrs schad­stoffbelasteter Fische (5 μg/kg). Die maximal erlaubte Schadstoffbelastung von Speisefischen ist in mehreren Verordnungen geregelt. Bewertungsmaßstab sind über Modellvorstellungen ermittelte Sedimentrichtwerte, die von maximal erlaubten Konzentrationen in Fischen abgeleitet wurden.
- 97 Prozent für Dioxine – Schutz der menschlichen Gesundheit vor den Folgen der Verwendung belasteter Futtermittel in der landwirtschaftlichen Produktion (4 ng/kg). Die EU hat in einer Reihe von Richtlinien die Höchstgehalte für unerwünschte Stoffe in Futtermitteln geregelt. Anlass zur Aufnahme dieses Aspektes sind Schadstoffgehalte deutlich über den zulässigen Höchstmengen in Futter- und Lebensmitteln in mehreren Bundesländern aus landwirtschaftlichen Betrieben, die zu einem hohen Anteil in aktuellen bzw. früheren Überflutungsbereichen der Elbe wirtschaften.

In den Tabellen 6 (MLU [54]) und 7 (FGG Elbe [51]) wird die Zuordnung der *Schadstoffbeispiele* zu den *Maßnahmen* „Reduzierung punktueller und diffuser Stoffeinträge aus dem Bergbau, Altlasten und Altstandorten“ am Beispiel „ÖGP Bitterfeld – Sicherungs- und Sanierungsvorhaben“ verglichen:

Tabelle 6 Schadstoffe mit überregionaler Bedeutung (Dioxine/Furane wie in der MLU-Vorlage [54] hervorgehoben!) im Ökologischen Großprojekt-Bitterfeld-Wolfen, MR Hans Peschel, 23. April 2009

| **Schadstoffe mit über­regionaler Bedeutung** | **Art der Maßnahme** | **Maßnahme** |
| --- | --- | --- |
| As, Pb, Cd, Cu, Hg, Zn, ***Dioxine/Furane****, HCB, HCH, Organozinn | Reduzierung punktueller und diffuser Stoffeinträge aus dem Bergbau, aus Altlasten und Altstandorten | Sanierungsvorhaben  Ökologisches Großprojekt Bitterfeld/Wolfen |

Tabelle 7 Beispiele für Maßnahmen zur Reduzierung der Schadstoffbelastung. Aus: Bewirtschaf­tungsplan FGG Elbe vom 11. November 2009 [51], Auszug aus Tabelle 7.1 (Seite 201)

| **Art der Maßnahme** | **Maßnahme** | **Größter Effekt für ..** |
| --- | --- | --- |
| Reduzierung punktueller und diffuser Stoffeinträge aus Berg­bau, Altlasten und Altstandorten | Ökol. Großprojekt Bitterfeld/­Wolfen – Siche­rungs- und Sanierungsvorhaben | HCH |

In der Tabelle 7 wird beim Maßnahmenbeispiel Ökologisches Großprojekt Bitterfeld-Wolfen nun nur noch „HCH“ („größter Effekt für …“) aufgeführt, die stärker feststoffgebundenen Schadstoffbeispiele einschließlich der *Dioxine/Furane* aus der MLU-Vorlage sind entfernt worden. Der Originaltext [51] lautet (Seite 200, letzter Absatz): *„In der Tabelle 7-1 sind beispielhaft Maßnahmen des 1. Bewirt­schaftungs­plans aufgeführt, die zu einer spürbaren Reduzierung der Schadstoffbelastung beitragen werden. Bei den in der Tabelle genannten Schadstoffen handelt es sich um die, für die der höchste Reduzierungs­effekt erzielt wird. In der Regel werden weitere Schadstoffe oder -gruppen mit erfasst.“*

An einer anderen Stelle in diesem ersten Bewirtschaftungsplan für den deutschen Teil des Elbe­ein­zugs­gebiets ist eine ausführliche Tabelle^^[[7]](#footnote-7)^^ mit „Dioxinen/Furanen“ erhalten geblieben und man muss so ernsthaft nach dem Sinn dieser Aktion fragen….. Während jedoch die „Streichaktion Dioxin“ eher einen symbolischen Effekt suchte, war die nachfolgend beschriebene Manipulation frontal gegen das strategische Prinzip „Source First“ gerichtet, gegen die umweltpolitische Forderung, *der Sanierung der Verschmutzungsquelle erste Priorität einzuräumen.*

***5.3 Dioxin-Längsprofilaufnahmen 2008 – FGG Elbe lässt Hinweis auf „Source First“ streichen***

Mit den ersten Diskussionen über Maßnahmen musste die Landesanstalt für Altlastenfreistellung erkennen, dass sie bald das Gesetz des Handelns im größeren Rahmen verlieren würde. Bei der Aus­schreibung des Wettbewerblichen Dialogs (Abschnitt 7.3) konnte sie zwar ihr vorgeschobenes Thema „Erarbeitung eines Systemverständnisses“ durchsetzen und mit einem *„mich interessieren Ihre Sedi­mente nicht“* der aktuellen Gemeinschaftsthematik stellvertretend ihr Missfallen ausdrücken.

Inzwischen war aber nicht mehr abzustreiten, dass die Unterelbe mit den Dioxinkongeneren­mustern aus dem Raum Bitterfeld/Spittelwasser ein sehr effizientes zeitliches und räumliches Indikator­system aufwies. Bereits 2007 berichteten Götz und Kollegen [55] über sehr hohe Dioxin-Konzentrationen in datierten Elbe-Sedimentkernen von Pevestorf und Heuckenlock/-Hamburg, die den 1950er und 1940er Jahren zugeordnet werden konnten. Die Höhe der Dioxin- Konzentrationen und die Ähnlichkeit der Dioxin-Muster mit den (undatierten) Sedimentkernen des Spittelwassers zeigen den kontinuierlichen Zusammenhang mit Hochwasserereignissen über einen langen Zeitraum an.

Erst recht mit der Studie von Umlauf et al. [56] und Stachel et al. [57] „Dioxin-Längsprofil­­aufnahmen“ von 2011 wurde die Langfristigkeit der Kongenerenmuster im Hauptstrom der Elbe das Argument für (1) überregionale Zusammenhänge der „Hot Spots“ und damit (2) für einen „Source first-Ansatz“ bei Maßnahmen gegen die Ausbreitung von Dioxin im Elberaum. Die ganz eindeutige Aussage steht unter *„4.4.3 High water events“* in der englischsprachigen Version (Umlauf et al. [56]) und lautet wie folgt:

[i]*„The main hazard with respect to dioxin contamination in the Elbe are high water events occurring in the Spittelwasser-Mulde-Saale system.* [ii] *Consequently, an improvement in the immission situation for the Elbe can only be expected after the corresponding sources have been adequately cleaned up.* [iii] *A reduction in the pollutant loads in the Elbe would have a positive effect on the immission situation in the coastal parts of the North Sea as well.“*

Der Text des Autorenkollektivs Umlauf et al. wurde nun von der *FGG Elbe* in anderer Namensreihung mit einer „Redaktionellen Bearbeitung“ (Dr. F. Krüger, Fa. ELANA) als deutschsprachige Version ver­öffentlicht ([57]). Nun lautet die Zusammenfassung *„3.5.3 Hochwasserereignisse“*ganz anders:

[i]*„Die eigentliche Gefahr des Dioxin-Eintrags in die Elbe dürfte hauptsächlich mit den Aus­wirkungen von Hochwasserereignissen im Spittelwasser-Mulde- und Saale-Fließsystem sowie durch Remobilisierungsvorgänge innerhalb der Elbe selbst in Zusammenhang stehen.* [ii] In Sachsen-Anhalt wird derzeit im Rahmen der Erarbeitung eines landesweiten Sediment­mana­ge­mentkonzeptes ein Projekt zur Frachtreduzierung überwiegend schwebstoffgebunde­ner Schadstoffe im Spittelwasser/Schachtgraben durchgeführt und in der Saale wurden weitere *Untersuchungen zur Identifizierung der Quellen angestoßen* (LHW-Bericht [39]). [iii] Diese vorbenannten Untersuchungen werden *zukünftig eine genauere Bewertung der Eintrags­situation in die Elbe* ermöglichen. Darauf aufbauend sollen standortspezifische *Maßnahmen­szenarien* betrachtet werden, *die zu einer Verbesserung der Immissionssituation in der Elbe beitragen können.“*

Die Veränderungen der Sätze [ii] und [iii] in der Zusammenfassung „Hochwasserereignisse“ der von der FGG Elbe gesponserten Version sind gravierend: In (ii) dient der Begriff „Sources/Quellen“ dem FGG-Bearbeiter lediglich als Stichwort, um auf zwei in *Sachsen-Anhalt* laufende bzw. angestoßene Projekte hinzuweisen, u.a. auf die Studie von Tauw [16], von der die LAF erwartete, dass sie eine Gegenposition zum „Prinzip Source First“ einnehmen würde. Auch im Satz [iii] wird die Sache selbst – Küstengebiete, neue Meeresschutzstrategie-Richtlinie – gänzlich ignoriert und durch eine Art Werbung für die Zielsetzungen der in [ii] genannten Projekte ersetzt. Die LAF und ihre Magdeburger Partnerinstitution FGG Elbe hatten offensichtlich erkannt, dass von der Thematik „Meeresschutz“ her Probleme ins Haus standen, nachdem die Kongenerenmuster der Bitterfeld- und Staßfurt-Dioxine bereits bis vor Helgoland verfolgt werden konnten [56/57].

Nur zwei Jahre lang blieben die Manipulationen durch die FGG Elbe bei der Thematik „Source First“ wirksam. Ende 2013 präsentierte die Flussgebiets­gemeinschaft Elbe ihr *Sedimentmanagementkonzept* ([58]; gleichlautend für die IKSE [59]). Bei den *speziellen Kriterien für Maßnahmen* legten sich FGG Elbe und IKSE auf die Linie bzw. das Prinzip „Source(s) first“ fest: Nachdem das Kriterium 1 *„die Lösung eines Problems an der Quelle bzw. die Beseitigung der Ursache ist zu bevorzugen“* am Spittel­wasser *ad absurdum* geführt wurde, muss die FGG Elbe künftig bei den anstehenden Maßnah­men – u.a. Sanierung im Flusslauf der Bode – auf dem Kriterium 2 insistieren: „besteht die ursächliche Quelle nicht mehr, sollte die Lösung möglichst quellnah erfolgen“ (*„Die Treppe von oben reinigen“* [58/59]).

Fazit Kapitel 5

Die Angriffe gegen „Dioxin“ (Abschnitte 5.1, 5.2) und der Versuch, den Ansatz „Source First“ zu unterdrücken (Abschnitt 5.3) hatten unterschiedliche Auswirkungen. Im ersten Fall könnte die FGG Elbe den aktualisierten Bewirtschaftungsplan (2015) entsprechend anpassen. Im zweiten Fall wurde versucht, nachträglich das Ergebnis des Wettbewerblichen Dialogs des Landes Sachsen-Anhalt zu rechtfertigen. Man muss davon ausgehen, dass die Flussgebietsgemeinschaft Elbe hier einer Forde­rung der Landesanstalt für Altlastenfreistellung gefolgt ist. Zwei Fragen bleiben offen: (1) Wurden die vier Autoren von diesen Änderungen informiert, und (2) welche Rolle hat der von der FGG-Elbe beauftragte Bearbeiter des Ispra-Reports, Dr. F. Krüger, ein Ko-Autor bei unserer HPA-Risikostudie [43], bei dieser Manipulation gespielt?

**6. Interne LAF-Studien zu Altlasten der Bitterfeldregion mit vielen Ungereimtheiten**

Es gibt gute Gründe, sich vor Dioxin zu fürchten – dem *Überall-Gift*, wie es „Der Spiegel“ in einem Rückblick auf die großen Dioxinskandale in den 1950er bis 1980er Jahren (Love Canal, Seveso, Times Beach) nannte, verknüpfend mit dem Anblick des ukrainischen Oppositionspolitikers Wiktor Juschto­schenko nach dem Vergiftungsanschlag auf ihn als Präsidentschaftskandidaten im Herbst 2004 [60]. Wir hatten in Hamburg zwei Beispiele, die uns mit dem Ausmaß von Dioxin-Katastrophen bekannt machten, aber auch gleichzeitig mit den Fähigkeiten, wie Politik, Verwaltung und Wissenschaft mit solchen Ausnahmesituationen gemeinsam umgehen können:

Letzteres gilt vor allem für den Fall der Altdeponie Hamburg-Georgwerder, dem höchsten Müllberg der Republik, an dessen Fuß im November 1983 das Seveso-Dioxin 2,3,7,8-TCDD in aussickerndem Öl gefunden wurde [61]. Unmittelbar nach den ersten Befunden wurde ein umfangreiches Sanierungs-/Sicherungsprogramm in Gang gesetzt und es entwickelte sich daraus das weltweit größte Projekt im Altlastenbereich [62, 63]. Die *Maßnahme* ist eine Kombination von Oberflächenabdichtung (mit begrenzter Wirkungsdauer) und einer hydraulischen Sanierung, bei der das kontaminierte Wasser durch eine Schutzbrunnengalerie abgepumpt wird;. Die Investitionskosten lagen bei ca. 92 Mio. €, die Betriebskosten 1984 – 1994 bei ca. 7 Mio. und ca. 12 Mio. € wurden auf dem Vergleichswege von 7 Firmen bezahlt [63].

Das Fazit war, dass die Langfristigkeit der potenziellen Schadstoffausbreitung in Deponien präventive Ansätze notwendig macht, bei denen die Forderung nach dem Stand der Technik nicht nur auf die Problem­lösungen beschränkt ist, sondern auch die Entwicklung und Anwendung adäquater Prüf­verfahren für Langzeit­prognosen einbezieht; die Eignungsuntersuchungen selbst müssen sich an dem nötigen Aufwand für Sanierungen *nach vorangegangener Fehlentwicklungen* messen lassen [64]. Aus diesen Erfahrungen folgte, dass sich die deutsche Abfallwirtschaft grundsätzlich an dem *schwei­zeri­schen Konzept* und Leitbild der Endlagerqualität orientierte, dessen zentraler naturwissen­schaft­lich-technischer Grundsatz lautet: „Organische Stoffe gehören nicht in ein Endlager“ [65, 66].

Maßgebend für den Umgang mit *Dioxin-Altlasten der chemischen Industrie* wurden die Erfahrungen der Firma Boehringer Ingelheim in Hamburg. In den Jahren 1985 und 1986 fand man auf dem 85.000 m² großen Werksgelände und in dessen Umfeld massive Kontaminationen durch chlororganische Ver­bindungen, u.a. Dibenzodioxine und -furane; auch im Sediment des angrenzenden Moorfleeter Kanals wurden die sog. „Boehringer-spezifischen Schadstoffe (BSS)“ aus der früheren Produktion von *T-Säure* festgestellt [67]. Bei der Entwicklung der Maßnahmen war die speziell gegründete Boehringer-Tochter DEKONTA federführend [67]:

- 1989 wurde der Hamburger Umweltbehörde ein erstes Sanierungskonzept vorgelegt; dies sah die vollständige Sanierung des Geländes und des verunreinigten Umfeldes vor, d. h. den Gebäud­e­abbruch sowie eine *thermische Dekontamination* des hoch belasteten Bodenmaterials. Die Reinigung der tiefer gelegenen Kontaminationen sollte durch eine mikrobiologische Boden- und Wasserreinigung erfolgen; technische Probleme beim Dauerbetrieb der thermischen Anlage und insbesondere das *Scheitern der mikrobiologischen in-situ-Versuche* führten 1994 zum Abbruch.
- 1995 wurde ein neues Konzept zur Sicherung der Altlast durch Einkapselung sowie zur Sicherung und Sanierung der Grundwasserfahne erstellt (u.a. durch die Firma IGB, die auch an der *Machbar­keitsstudie Sanierung Spittelwasser von 1993* [7] maßgeblich beteiligt war). Für die Einkapselung der Altlast waren eine umlaufende Dichtwand und ein Asphalt-Deckel verwendet; trotz einiger Komplikationen – u.a. wurde im Moorfleeter Kanal eine ergänzende Spundwand für die Ufer­sicherung gerammt – wurde die Maßnahme 1998 abgeschlossen.

Insgesamt zahlte *Boehringer Ingelheim* alleine 167 Millionen Euro; die Einnahmen aus der Verpach­tung des Geländes decken die Betriebskosten für die Bewirtschaftung des Dichtwandtopfes sowie die Fahnensicherung in Höhe von rund 500.000 Euro jährlich [67].

Neben diesen gut dokumentierten „Erfolgsgeschichten“ muss hier auch das Beispiel einer großen Deponie/Altlast genannt werden, die *seit 1983 unter Dioxinverdacht* steht und nicht zuletzt wegen der politischen Dimensionen immer wieder in die Schlagzeilen geriet [68, 69]: Die Sicherheit der Deponie Ihlenberg in Mecklenburg war umstritten, seit die DDR 1979 den Betrieb in Schönberg gegründet hatte; jahrelang versuchten ostdeutsche Ökogruppen und Westgrüne einen Lieferstopp durchzusetzen [70]. Der ehemaligen *„Deponie Schönberg“* fehlte von Beginn an alles, was zu einem Mindeststandard von Sicherheit beigetragen hätte [68]; bis heute ist nicht bekannt, was dort vergraben liegt [71]. Der von 1979 bis 2005 befüllte Altteil, auf dem auf 60 Hektar rund 18 Millionen Kubikmeter abgelagert wurden, wird seit 2011 „versiegelt“; seitdem erfolgt die Ablagerung unter heutigen technischen Standards auf einer neuen Müllhalde nebenan [72]. Es ist geplant, den Standort der Deponie Ihlenberg mittels einer *„Multifunktionalen Abdichtung“* baulich getrennt und nach dem Konzept „Deponie auf Deponie“ weiter zu führen [73]. Man kann sich nur wundern, wie schnell die konzeptionellen Fort­schritte der 1990er Jahre – die Deponie als klassisches Beispiel im Leitbild „Nachhaltigkeit“ [74] – wieder einem *marktwirtschaftlichen Kurzzeitdenken* weichen mussten.

Der Chemiepark in Bitterfeld war in den frühen 1990er Jahren, nachdem mehr als 300 Millionen Mark für die Sanierung des 590 Hektar großen verseuchten Geländes nach Sachsen-Anhalt geflossen waren, ein bevorzugtes Ziel von illegalen Müllablagerungen. Das FOCUS Magazin schrieb im August 1994 [75]: *„Viele Firmen haben Narrenfreiheit. Man kann unbemerkt 10 000 Liter von irgendwelchem Zeug in den Gulli kippen“*, behaupten Umweltexperten und Mitarbeiter der ChemiePark GmbH. Die Berliner Ingenieurgemeinschaft Technischer Umweltschutz (ITU) fand in den Abwässern und in den Sedimenten der Kanäle rund um den Chemie-Park außergewöhnlich hohe Werte für DDT, Dioxin und Lindan. *„Es handelt sich um aktuelle Einleitungen“*, sagte ein ITU-Mitarbeiter. *„sie erfolgten zwischen ein Uhr nachts und fünf Uhr früh.“*. Die für 320 Millionen erstellte Kläranlage arbeitete nicht nur uneffektiv, *„die geklärten Abwässer fließen über den kleinen Fluss Schachtgraben, in dem sich 20 000 Tonnen giftiger Schlick ablagerten, in den Fluss Mulde und von dort in die Elbe …“* [75].

***6.1 Machbarkeitsstudie „Schadstoffreduzierung“ im Kerngebiet der Chemieregion – ohne Dioxin***

Die Landesanstalt für Altlastenfreistellung und die Großmann Ingenieur Consulting GmbH (GICON), die sich bis dahin im Wesentlichen mit der Wasserphase von Altlasten befasst hatten, wurden im August 2007 im Ökologischen Großprojekt Bitterfeld/Wolfen erstmals konkret mit dem Problem der dioxinhaltigen Sedimentaltlasten konfrontiert [76]: *„Neben der flächenhaften Verbreitung von Zinn­organika oberhalb der Qualitätsnormen wurde auch auf die Problematik von Dioxinen und Furanen in Mulde und Elbe hingewiesen; eine Ursachenermittlung kann mit dem bisherigen Kenntnisstand nicht abgeleitet werden. Aus diesem Grund sollen ausgewählte Oberflächenwasser-Messstationen auf Zinnorganika und Dioxine/Furane im Sediment … untersucht werden“*.

Eine Studie der P-D ChemiePark GmbH/LAF vom Oktober 2007 berichtet von der Reinigung von Kanälen und Absperren von nicht betriebenen Stichkanälen zur Unterbindung der Remobilisierung von ggf. relevanten Altsedimenten [77]. Eine erste umfassende Studie *„Analyse und Bewertung der tech­nischen Möglichkeiten für eine Problemlösung im Bereich der Oberflächenwässer des Grund­wasse­r­körpers ­in Bitterfeld“* wurde durch LAF/GICON in Auftrag gegeben [78]. Betrachtungsgebiet ist das Wassersystem bestehend aus der Ableitung Freiheit III, dem Landgraben, dem Schachtgraben und dem Spittelwasser im Bereich des Grundwasserkörpers Bitterfeld-Wolfen. Die Vorstudie sollte die Chancen für technische Maßnahmen ausloten, Grenzwertüberschreitungen von Hexachlorcyclohexan (HCH) in der Mulde zu verringern [79].

Der Verzicht auf die Aufnahme der Stoffgruppe der Dioxine/Furane lässt sich nur als *„Marotte“* von LAF und GICON erklären, denn die Jahresmittelwerte 2006 für PCDD/F aus Zentrifugenproben von Schwebstoffen aus dem Schachtgraben (Jeßnitz) und Spittelwasser (unterhalb Schachtgraben) lagen ca. 50fach über den Werten der Mulde (Ablauf Stausee; 744 bzw. 741 gegenüber 12,7 ng I-TEQ/kg [39]).

Das hier skizzierte Maßnahmenprogram begründet sich überwiegend auf Routinetechniken und hätte angesichts des hohen Gefährdungspotentials der beteiligten Schadstoffe, insbesondere der PCDD/Fs, mit besonderem Nachdruck realisiert werden müssen. Aus der Sicht eines Bürgers von Sachsen-Anhalt muss es völlig unverständlich sein, dass seine neugegründete und finanziell üppig ausgestattete LAF erst 2008 begann, sich mit einer Vorstudie in das Thema „Sedimentaltlasten“ einzuarbeiten und damit für ihr engstes Umfeld die notwendigen Sicherungs- und Sanierungsmaßnahmen zu organisieren.

***6.2 Unsachgemäßer Umgang mit einer Unterhaltungsmaßnahme – auch hier fehlt Dioxin!***

Für das Jahr 2008 vermerkt der *Dioxinbericht des Landesbetriebs für Hoch­wasserschutz und Wasser­wirtschaft Sachsen-Anhalt* ([LHW) vom März 2012 [39] deutlich erhöhte Dioxingehalte in Schweb­stoffproben aus dem Spittelwasser und aus der Mulde, die nicht durch natürliche hydrologische Effekte erklärt werden können: Im Spittelwasser unterhalb des Schachtgraben stiegen die Jahresmittelwerte der Dioxingehalte aus AMB/Schwebstoffkastenproben von *1034 ng I-TEQ/kg*(3 Proben) in 2007 auf *1608 ng I-TEQ/kg* (5 Pr.) in 2008 und gehen in 2009 auf *1070 ng I-TEQ/kg* (3 Pr.) und 2010 auf *832 ngI-TEQ/kg* (3Pr.) zurück. LHW (2012 [39]) brachte die Anomalien direkt mit den Unterhaltungsarbeiten in Verbindung, die im Schachtgraben in den Monaten Juli/­August 2008 durchgeführt worden waren.

Tatsächlich findet sich in dem Leserbrief von Keil, Großmann und Weiß [81] auf den JSS-Artikel „Sedi­­ment research, management and policy“ [80] eine entsprechende Passage: „*In 2008, watercourse maintenance measures in the Spittelwasser system were undertaken. As part of these measures, sub­stan­tial amounts of sediment were removed, and the proper disposal of these contaminated sediments was financed by the LAF“^[[8]](#footnote-8)^*. Die Zielsetzung der Maßnahme in der Zeit vom 14.07.2008 – 08.10.2008 lautete: *„Die Sofortmaßnahme diente der Wiederherstellung der Abflussleistung des Schachtgrabens. Die auf der Gewässersohle abgelagerten Sedimente waren nicht Teil der Gewässer­unterhaltungs­maß­nahme …. Eine Beseitigung von potentiellen Schadstoffquellen bzw. die langfristige Verminderung von Schadstoffeinträgen war nicht Bestandteil der Unterhaltungsmaßnahme.“*

Die *Abschlussdokumentation*, die an *keiner Stelle den Begriff „Dioxin“ nennt*, bestätigt gerade dadurch indirekt den Vorsatz bei der Planung und Durchführung eines *Umweltfrevels ersten Grades*:

- Die wichtigste Begrenzung beim Umgang mit hochtoxischen Ablagerungen sind die Verluste durch die Resuspension im Gewässer. Der Einsatz eines Baggers ist bei Dioxinkonzentrationen, die in der Größenordnung der Analysendaten des STAU aus dem Spittelwasser ([11], Abschnitt 2.2) liegen dürften, nicht zulässig. Die Selbsteinschätzung *„Zusammenfassend kann festgestellt werden, dass die eingesetzten Entnahmetechnologien bei entsprechenden Überwachungs­maßnah­men geeignet sind …“* ist nur ein weiterer Hinweis auf das fehlende Problembewusstsein bei den verantwortlichen Akteuren. Beispiel­haft für eine professionelle Vorgehensweise ist eine Umlage­rungstechnik genannt, die im Hamburger Hafen eingesetzt wurde ([82]; Kasten 3).

**Kasten 3: Möbius Press- und Fördersystem für gewässerschonende Sedimententnahme**

Bei der subaquatischen Deponierung kontaminierter Sedimente kommt der Umlagerungstech­nik eine besondere Bedeutung zu, da es bereits in dieser Phase zu maßgeblichen Schadstoff-Freisetzungen aus dem kontaminierten Sediment kommen kann. Hierfür sind dann vor allem die Resuspension schad­stoff­­belasteter Feinpartikel oder die Rücklösung von Schadstoffen durch den Kontakt mit dem Ober­flächen­wasser verantwortlich.

Für die Ausführung der subaquatischen Lagerung der Sedimente im Sporthafen von Hitzacker­/­Elbe wurde das von der Möbius Baugesellschaft GmbH & Co. entwickelte, patentierte Möbius-Press- und Fördersystem (MPF) vorgesehen. Es handelt sich um eine Technik, die das Frei­setzungsrisiko minimiert, indem das Sediment ohne Zusatz von Wasser in relativ gut konsoli­dierter Form ausgehoben, gefördert und am Bestimmungsort eingebaut werden kann. Die An­lage be­steht aus der Baggereinheit (Hydraulik­bagger), dem Aufgabetrichter, der Pressein­heit, der geschlos­se­nen Rohrleitung (ø 600 mm) und der Einbaueinheit (Abb. 5). Die Bagger- und die Pressein­heit ist auf einem Ponton installiert, die Rohrleitung ø 600 mm z.T. schwim­mend und an Land verlegt, die Einbaueinheit wiederum schwimmend an Drähten geführt.

Das Sediment wird mit dem Hydraulikbagger aufgenommen und in den Aufgabetrichter der Pressein­heit eingebracht. Das Hüllrohr ø 1,0 m durchstößt das zu fördernde Sediment und schließt es in der Pum­penkammer ein. Der Presskolben ø 1,0 m, Hublänge 2,0 m, presst anschließend das Sediment durch die Rohrleitung ø 600 mm zur Einbaueinheit. Die Einbau­einheit ist mit Winden, einem Schwebe­rohr und einer Einbaudüse ausgerüstet. Der Austritt des Sedimentes aus der Düse geschieht unter Wasser, so dass das Baggergut subaquatisch auf dem Grund der Einbaufläche abgelagert wird. Da der Transport des Baggergutes ohne zusätz­liches Wasser geschieht, muss in der Einbaufläche eine Was­ser­haltung installiert werden, um die Schwimmtiefe der Einbaueinheit zu gewährleisten.


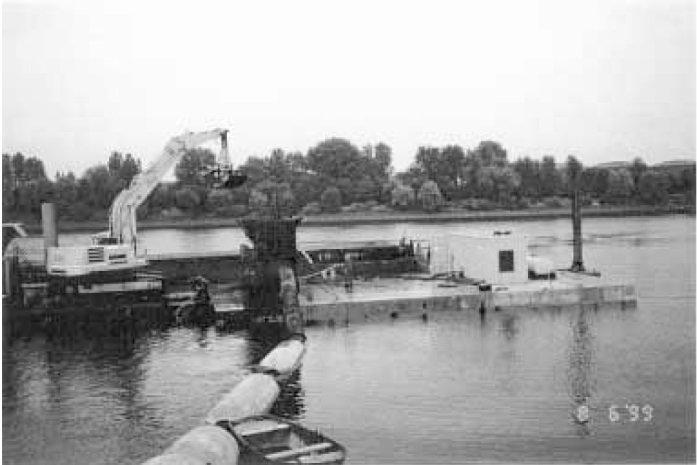

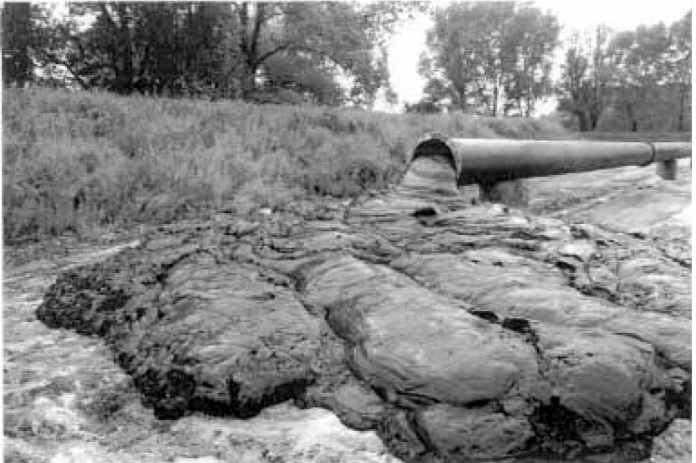


**Abb. 1** ***Entnahme/Umlagerung von Baggergut mit Hilfe des MPF-Verfahrens.*** Der Greif­bagger beschickt den Auf­gabetrichter in der Bildmitte. Das Baggergut wird durch die Rohr­leitung im Vordergrund gefördert; (rechts): mit dem MPF-Verfahren gefördertes Sediment auf einem Spülfeld. Das Material besitzt auf­grund des niedrigen Wassergehalts eine sehr hohe Viskosität (Fotos: Möbius GmbH)

Bei dem geplanten Einsatz im Sporthafen von Hitzacker/Elbe wurde zuerst eine Berechnung der Massenermittlung des Ausbau- sowie des Einbauvolumens durchgeführt, die gezeigt hat, dass das zu baggernde Sediment in dem abgedämmten Zulaufkanal untergebracht werden kann. Als Referenz­objekte wird auf die mit diesem System erfolgreich ausgeführten Arbeiten im Vulkanhafen Hamburg (ca. 120.000 m³), im Holzhafen (ca. 20.000 m³) und Spülfeld Berndsdorf Landkreis Ueckermünde (20.000 m³) sowie kleinere Einsätzen im Mittellandkanal verwiesen.

- Da allen bei der Planung beteiligten Behörden klar sein musste, dass es sich bei der Maßnahme im Schachtgraben nicht um eine Routine-Aktion handeln würde, hätte auf jeden Fall der Landes­betrieb für Hochwasserschutz und Wasserwirtschaft (LHW) mit seiner speziellen Expertise für Dioxine einbezogen werden müssen. Es gab eine mysteriöse Unterbrechung der Baumaßnahmen „aufgrund von außergewöhnlichen analytischen Befunden“ und danach wurde ein renommiertes Insti­tut herangezogen, das später auch im Auftrag des LHW die Dioxinanalytik für die Sediment­proben aus Saale und Bode durchführte (siehe Kasten 2 auf Seite 19).

Für die Elbe-Unterlieger bleibt ein ernüchterndes *Fazit*: Die LAF, die bislang keine Maßnahmen zur Verringerung der von ihrem Boden ausgehenden Dioxingefahr durchgeführt hat, benutzt ihre erste praktische Aktivität im Bereich der Sedimentaltlasten um mit einer unsachgemäßen Billigtechnik die Dioxinproblematik im Unterelberaum weiter zu verschärfen. Wo bleibt die Flussgebietsgemeinschaft Elbe, die genau für solche Situationen eingerichtet wurde. Die im Jahresmittel 2008 überschlägig 30%-ige Zunahme der Dioxineinträge in die Elbe gegenüber den bereits hohen Dioxinfrachten aus dem Land Sachsen-Anhalt hätten vordringlich den unmittlbar Betroffenen mitgeteilt werden müssen. Rechtlich vorgegeben ist die Einhaltung einer Meldepflicht gegenüber den Unterliegerstaaten.

7. **Untätigkeit der LAF bei dioxinhaltigen Sedimentaltlasten: Verantwortung verweigert**

Als junger Sedimentforscher an der Universität Heidelberg wurde der Autor Ende der 1970er Jahre von der Staatanwaltschaft Baden-Baden angesprochen, die einen Fall verfolgte, bei dem zwei Mit­arbeiter einer Metallfabrik erfolgreich versucht haben sollten, Produktionsreste dergestalt am Ufer des Rheins abzulagern, dass diese beim nächsten Hochwasser weggeschwemmt werden konnten. Bei der Durchsicht des damals erstellten Gutachtens kam eine erstaunliche Professionalität, mit der sich verschiedene Dienststellen, vor allem die Wasserschutzpolizei, an der Beweissicherung beteiligt haben, zu Tage. Es konnte im Wasser eines nahegelegenen Altrhein-Sees nachgewiesen werden, dass dort eine signifikante Verschmutzung – um den Faktor 10 gegenüber dem Rheinwasser erhöhte Gehalte von Kupfer und Zink – bedingt durch die Elution der illegal abgelagerten Zinklaugen-Rückstände erfolgt war. Vielleicht noch interessanter war die Suche nach dem Motiv für diese Handlung: Aus den akribisch gesammelten Materialspuren konnte feststellt werden, dass die Zusammensetzung der abgelagerten Schlämme deutlich von der zwischen der lokalen Firma und dessen belgischen Vertragspartner vereinbarten Qualität abgewichen war. Man konnte danach spekulieren, dass wohl dieser Anteil der Gesamt­lieferung einer bestimmten Qualitätsnorm nicht entsprochen hat und deshalb eine Verschiffung nicht vorgenommen wurde. Soviel zu einer privat­wirtschaftlichen Aktion – in Kapitel 7.2 wird dargestellt wie vermutlich derselbe physikalische Prozess unter amtlicher Regie im Spittelwasser genutzt wurde.

***7.1 Verpflichtungen aus der Machbarkeitsstudie zur Sanierung der Spittelwassersedimente***

In der Machbarkeitsstudie zur „Sedimentsanierung des Spittelwassers“ [7] waren die Gutachter der Auffassung, dass bei diesen Gewässer-Altlasten die Kriterien eines großen Gefährdungspotentials gegeben sind, vor allem weil Schadstoffe wie Dioxin hochtoxisch und im Falle von Hochwasser­ereignissen sehr mobil sind, und sowohl die terrestrischen Ökosysteme im näheren Umfeld des Spittel­wassers als auch die aquatischen Lebensgemeinschaften in den Fließgewässern, Mulde und Elbe bis zur Nordsee gefährden können: *„Fachlich ist somit der Handlungsbedarf aufgrund von Vorsorge­erfordernissen eindeutig zu begründen“* [7]. Im Abschnitt *„Handlungspflichten aus rechtlicher Sicht“* der Machbarkeitsstudie befindet sich eine ebenso eindeutige Mahnung an die zuständigen Behörden ([7] S. 81):

*„Für die Gutachter steht zweifelsfrei fest, dass nicht nur aus ökologischer Sicht die drin­gende Notwendigkeit für eine Entnahme der Spittelwassersedimente zwecks Sanierung des Gewässers besteht, sondern auch Handlungspflichten und -befugnisse für die zuständigen Behörden aufgrund des Sicherheits- und Ordnungsgesetzes von Sachsen-Anhalt in Verbin­dung mit den Naturschutz- und Wassergesetzen gegeben sind. Untätigkeit könnte den Vor­wurf der Amtspflichtverletzung auslösen“.*

***7.2 Umgang mit mobilen Sedimentaltlasten – ein typischer Fall für das Vorsorgeprinzipimente***

Aus gutem Grund hat der vorliegende Rückblick auf den Umgang mit dioxinhaltigen Sediment­alt­lasten in Sachsen-Anhalt einige Jahre vor der WRRL-Umsetzung begonnen. Dadurch wird offen­kundig, dass die *Fehlentwicklungen* nicht so sehr vom Wechsel der Finanzquellen – von Bitterfeld nach Bonn [1] – verursacht waren, sondern vielmehr als eine Folge von späteren *Umorganisationen* in den Behörden (Kapitel 2 und 4) auftraten. So wurden offensichtlich die regel­mäßigen Untersuchungen zur Entwicklung der Spittelwassersedimente aus den 1990er Jahren [9-11] über 2000 hinaus nicht fort­geführt; damit ging das Wissen über die höchst sensible Situation in diesem international bekannten Fallbeispiel *vor Ort* verloren und es wurde erst im Zuge der Tauw-Studie [16]) *eher beiläufig entdeckt*, dass inzwischen ein Großteil der dioxinreichen Sedimente im Spittelwasser (noch 1997 geschätzte 20.000 m³ Sedimentmenge) weggeschwemmt worden war.

Bereits Ende der 1990er Jahre wurde nach den *regelmäßigen Untersuchungen des Staatlichen Amtes für Umweltschutz* (STAU) abgeschätzt, dass die hochkontaminierten Feinkornsedimente im Spittel­wasserbach bei episodischen Hochwässern von etwa 5jähriger Wiederkehr (HQ_5_) aufgewirbelt werden. Aus dem Ausbreitungsmodell des Umweltforschungszentrums aus dem Jahr 2006 im Kasten 4 erhält einen geradezu filmischen Eindruck über die zeitliche Entwicklung eines Hochwassers im Spittel­wassergebiet, ausgelöst durch die Überflutung des Salegaster Forsts durch die Mulde [83] .

**Kasten 4: Dioxin-Altlast im Spittelwasser als ‚Zeitbombe‘ bei extremen Hochwasserereignissen**

Bei der Verfrachtung von Schadstoffen in Oberflächengewässern spielt die mechanische Aufwirbelung von Sedimenten eine dominierende Rolle. Es sind vor allem Hoch­wasser­ereignisse, die durch die erhöhten Sohlschubspannungen einen exponentiellen Anstieg der Schweb­­stoff­konzentrationen im Wasser und deren Ausbreitung über große Entfernungen bewirken.

Das Spittelwasser und seine Umgebung sind Teil der Muldeaue. Strecken­weise ist das Spittelwasser als Flutrinne der Mulde aufzufassen, da eine Querverbindung über einen Durchstich gegeben ist, in dem das Wasser ganzjährig über ein Wehr zuströmt. Die Mulde kann dann zur Remobilisierung von Spittel­wasser­­sedimenten beitragen, wenn bei Abflüssen von >200 m³/s sich die Mulde über den Rade­gaster Forst ausbreitet und auch durch die Spittel­wasserniederung fließt.

In dem SARISK Projekt^^[[9]](#footnote-9)^^ wurden am Bei­spiel des Früh­jahrhochwassers 2006 die Fließwege und Über­­schwemmungsflächen der Spit­telwasser­niederung simuliert. Die Überflutung beginnt im Bereich der Greppiner Wiesen (unten rechts in der Übersichts­karte des Spittelwassergebiets). Nach wenigen Stunden erreicht das Wasser den Zuflussbereich der Lobber in das Spittelwasser (rechter Zufluss süd­lich von Jessnitz und wird dort über das Spittel­wasser abgeleitet.

Überflutung 8 Stunden

**Q = 207 m³/s**

Überflutung 4 Stunden

**Q = 180 m³/s**

Überflutung 24 Stunden

**Q = 276 m³/s**

Überflutung 12 Stunden

**Q = 234 m³/s**

In dem Tauw/LAF-Abschlussbericht „Frachtreduzierung Spittelwasser“ vom 21. Oktober 2013 [16] wird ein umfassendes Bild der Situation gezeichnet: *„Unter Hochwasserbedingungen weist der Spittelwasserstrom einen höheren Anteil an der Gesamtfracht der Mulde auf, was aber zumindest teilweise auch auf die Überströmung von Muldewasser in das Spittelwasser und die mitgeführte Hintergrundbelastung zurückzuführen ist. Neben der Hintergrundbelastung der Mulde ist davon auszugehen, dass die Mobilisierung von belasten Partikeln aus den Überflutungsflächen sowohl im Salegaster Forst als auch in den Auenflächen entlang des Spittelwasserlaufs einen Anteil an der hochwasserbezogenen Schadstofffracht hat.“*

Die exponierte Situation im Spittelwasser und die Folgen der relativ häufig auftretenden Hochwässer musste den verantwortlichen Dienststellen klar sein – offen war lediglich der *Eintrittszeitraum und die Intensität des Flutereignisses*. Es handelte sich um ein klassisches Beispiel für die Anwendung des *Vorsorgeprinzips,* das schon früh Bestandteil des Umweltrechts war und bei allen langfristigen und schwer prognostizier­baren Entwicklungen Pate stand. Sein unbestreitbarer Vorteil im Vergleich zu dem „Schaden­verhütungs- und Nachbesserungsprinzip“ ist die *frühzeitige Reaktion* [84]. Hochwässer mit den exponentiell gesteigerten Erosionseffekten und Feststofffrachten sind in ihrer *Ubiquität* die typischen Fälle für dieses Prinzip und dessen Bedeutung wird eher noch steigen, wenn – wie zu befürchten ist – die *Eintrittshäufigkeit von Extremhochwässern im Zuge des Klimawandels weiter zunimmt* [85, 86].

Aber was hilft das beste Prinzip, wenn die Administration es nicht mehr kennt oder nicht mehr ernst nimmt oder bewusst verdrängt. Letzteres konnte gar nicht so einfach sein, gehörten doch die Partner der Landesanstalt für Altlastenfreistellung im gemeinsamen WELCOME-Projekt (Abschnitt 3.5) zu den Pionieren auf dem Gebiet der Sedimentsanierung (Rulkens [87]), zuerst bei der Auswahl von viel­versprechenden Prozessen bei der *Reinigung und Stabilisierung* (*POSW I*, 1992 [88]), anschließend bei der Entwicklung und Anwendung von *praktischen Verfahren* (*POSW II*, 1997 [89]. Im eigentlichen WELCOME-Projekt haben haben die Kollegen von der Universität Wageningen mit dem Beitrag *„Management Scenarios for Contaminated Sediments at Megasites“* [90] das Gegenstück zu der Arbeit von Großmann et al. [91] über „Integrierte Managementstrategien in Grundwässern von Alt­lasten­großstandorten“ beigesteuert.

***7.3 Wettbewerblicher Dialog „Systemverständnis“ – relevante Erkenntnisse oder Hinhaltetaktik?***

Mit der Begründung *„von einer hohen Konzentration allein und direkt auf Wirkungen bzw. Risiken in räumlich weit entfernten Gebieten zu schließen und hieraus abzuleiten, welche Maßnahmen sinnvoller Weise zu ergreifen sind, ist nicht zulässig …“* war die LAF im Herbst 2007 gegen die Anwendung des „Source First Prinzips“ am Spittelwasser ins Feld gezogen (Kapitel 5.1). Ein zweiter Angriffspunkt war das *Hintergrundpapier der FGG Elbe* [55] vom April 2009 zur überregionalen Ableitung von Bewirtschaftungszielen mit dem Schwerpunkt Schadstoffe (Kapitel 5.2).

Bereits mit Datum von 29. Mai 2009 hatte die LAF einen wettbewerblichen Dialog mit dem Titel *„Frachtreduzierung überwiegend schwebstoffgebundener Schadstoffe der* [im Grundwasser­körper VM 2-4 gelegenen] *Fließgewässer mit dem Schwerpunkt Spittelwasser/Schacht­graben“* [92] ausschreiben lassen; Interessenten hatten u.a. die Begriffe *„Daten­grund­lage“* und *„Systemverständnis“* sowie die *regionale Einschränkung* [in eckigen Klammern oben] zu beachten.

Die Unterstützung des Autors galt einer Bietergemeinschaft, die die Kombination einer *Sofort­maß­nahme* – Source First – für die Dioxin Hot Spots im Spittelwasser mit *Langzeitmaßnahmen* an stärker belasteten Überflutungssedimenten propagierte (mit eigenen Beiträge aus dem Fallstudienvergleich Bitterfeld [20], der Risikostudie Elbe („Maßnahmen“ [93] und dem KORA-Verbund („Natürliche Schadstoffminderungsprozesse“ [94]). Die Bietergemeinschaft unter Führung der WISUTEC GmbH kam nicht zum Zuge.

Die Studie der Tauw GmbH Berlin [16], die den Zuschlag beim Wettbewerblichen Dialog der LAF erhalten hatte, zeigt, warum die Durchführung von überregionalen Maßnahmen bei Altsedimenten ungleich komplexer ist als im Falle rezenter Einleitungen. *„Damit wird auch eine Ausweisung eng begrenzter Gebiete höchster Belastung im Laufe der Zeit immer schwieriger, da sich alle betrachteten Schadstoffparameter in unterschiedlicher Weise über das Flussgebiet verteilt haben, vor allem mit Akkumulationen in Sedimenten von Stillwasser­zonen, Hafenbecken und Buhnenfeldern sowie auf Überflutungsflächen entlang der Elbe und ihrer Nebenläufe*“. Für den engeren Untersuchungsbereich Bitterfeld gelten folgende Prioritäten [16]:

- Maßnahmenkonzepte mit dem Ziel der Quellenreduktion zur Entlastung der Unterlieger müssen auf *frachtbasierten Ansätzen* beruhen, und nicht auf konzentrationsbasierten Ansätzen, da letztere einen aktuellen Zustand nur ortsbezogen charakterisieren, aber keinen Anhaltspunkt bezüglich der Wirkung auf unterstromige Gewässer geben.
- Es ist aus den Ergebnissen der vorliegenden Studie zu schließen, dass *Maßnahmen* in der Spittel­wasserniederung, die eine merkliche positive Beeinflussung für die Unterlieger haben sollen, nur *auf die Auswirkungen des Muldewasserübertritts im Hochwasserfall ausgerichtet* sein können.
- Die Untersuchungen der Spittelwasseraue zeigen, dass die *Belastungsschwerpunkte in der Aue entlang dem Spittelwasserlauf* und im Bereich von ehemaligen Wasserläufen liegen, während die größten Flächenanteile eine weitaus geringere Belastung aufweisen. Prinzipiell wären hier somit *punktuell fokussierte Maßnahmen denkbar*.

Zwischen der ehemaligen Einmündung des Schachtgrabens in das Spittelwasser und der Einmündung des Spittelwassers in die Mulde werden die Bereiche als relevant bezüglich potenzieller Schadstoff­ablagerungen betrachtet, für die flächenhaft relativ große Überflutungshöhen ausgewiesen sind. *„In diesen Zonen ist davon auszugehen, dass im Zuge zurückgehender Pegel in der späteren Phase eines Hochwassers Sedimentationsprozesse einsetzen, die u. U. zu einer Ablagerung und Akkumulation schwebstoffgebundener Schadstoffe führen können. Insbesondere im Bereich lokaler Tiefstellen, wie z.B. reliktischen Flussmäandern, wird von einer hohen Wahrscheinlichkeit einer Akkumulation von Schwebstoffen und ggf. schwebstoffbürtigen Schadstoffen ausgegangen … “*. Hier wurde von Tauw [16] eine radiometrische Detailuntersuchung zur flächenhaften Dioxinbelastung durchgeführt.

Die radiometrische Kartierung mittels des MEDUSA-Verfahrens ist dafür ausgelegt, indirekt Merk­male der Bodenstruktur, Korngrößenverteilung und chemischen Zusammensetzung in den oberflächen­nahen Schichten (ca. 0,5 m) von Sedimenten und Böden zu bestimmen. Anhand der Strahlungsver­tei­lung werden Probenahmepunkte festgelegt, dann die Korrelationen der Messgrößen zur Strahlungs­intensität der gemessenen natürlichen Radioisotope ermittelt und daraus schließlich Schadstoffkarten abgeleitet. Ein praktisches Beispiel wird von Jacobs et al. [16] beschrieben:

„Die Untersuchungszone 2 liegt nördlich von Jeßnitz und umfasst einen Flussauenbereich von 38,3 ha Größe. Diese Zone stellt den Nahbereich links und rechts des Spittelwassers in der breiten Niederung nördlich von Jeßnitz dar. Untersuchungszone 2 besteht aus Brachland bzw. lichtem Uferwald. Die Relevanz dieser Fläche hinsichtlich der Untersuchung potenzieller Schadstoffakkumulationen wird als sehr hoch eingeschätzt, da dieser Bereich höchstwahrscheinlich während historischen Flutereignissen direkt durch das mit dem Spittelwasser zuströmende belastete Wasser überflutet wurde. Bei zurück­weichendem Hochwasser kam es in diesen vergleichsweise tief gelegenen Bereichen der Spittel­wasserniederung dann höchstwahrscheinlich zur Sedimentation von stark belasteten Feststoffen kommen.“ In der Folie Nr. 13 eines Workshop-Beitrags von Jacobs [95] „Auen­untersuchung mittels Gammaspektrometrie liegen die Dioxinkonzentrationen in der Zone 2 zwischen 2.500 und 4.000 ng WHO-2005 TEQ/kg, d.h. 125- bis 200fach über dem oberen Schwellenwert der FGG-Elbe (2014).

Fazit Tauw-Studie (Jacobs et al. 2013/2014)

Die Frage „Hinhaltetaktik“ oder „reölevante Erkenntnisse“ kann mit „sowohl .. als auch“ beantwortet werden. Die *„Erarbeitung eines Systemverständnisses“* für die Oberflächengewässer im Bereich des Ökologischen Großprojekts (ÖGP) Bitterfeld-Wolfen, ist in der Tauw-Studie vorbildlich gelungen. Dahinter bleiben die Erwartungen an die „*Ableitung von Maßnahmeerfordernissen“,* dem Hauptziel der Studie [16] zurück und am Ende steht nur der Hinweis *„eine Prüfung hierüber hinausgehender Maßnahmen wird durch die Verfasser empfohlen…“*. Man muss LAF und Tauw fragen: Warum wurden die Konzentrationsangaben für Dioxin von MEDUSA nicht in die Studie aufgenommen?

Prof. (em.) Dr. Ulrich Förstner 27. Mai 2015

Institut für Umwelttechnik und Energiewirtschaft

Eissendorfer Str. 40

D-21071 Hamburg

e-mail: u.foerstner@tuhh.de

Leitender Regierungsdirektor

Dr. Peter Heininger

Bundesanstalt für Gewässeerkunde

Am Mainzer Tor 1

**D-56068 Koblenz**

**Dioxin im und am Spittelwasser – Informationen der Fa. Tauw GmbH Berlin** .

Sehr geehrter Herr Dr. Heininger,

Ihr Ad hoc Team "Schadstoffe" hat bei der Abfassung des Hintergrundpapiers "Ableitung über­regionaler Bewirtschaftungsziele" (FGG Elbe 2009) und der Vorabfestlegung auf "Kriterien zur Auswahl und Priorisierung von Maßnahmen" (FGG Elbe/IKSE Sedimentmanagementkonzept 2013/2014) zwei entscheidende Standards gesetzt – vorbildlich für die internationale WRRL-Umsetzung. Nun habe ich jedoch im Hintergrunddokument zum aktualisierten Bewirtschaftungs­plan der FGG Elbe, Teilaspekt Schadstoffe (05.11.2014) unter Kapitel 6 Stand der Umsetzung und Erfolge (!) beim Beispiel Mulde: Dessau den Satz gefunden "Gezielte Untersuchungen zu den organischen Schadstoffbelastungen in Schwebstoffen, Gewässersedimenten und Auen des Spittelwassers und der Unteren Mulde zeigten, dass die in den 1990er Jahren beschriebenen massiven Fein­sediment­ablagerungen im Spittelwasser heute nicht mehr vorhanden sind" Punkt. Es war zu erwarten, dass diese Formulierung in der Fluss­gebietsgemeinschaft Elbe und in der inter­essier­ten Öffentlichkeit zu Fehldeutungen führen würde.

Dr. Jacobs und seine Kollegen von Tauw Berlin haben in ihrer Studie "Frachtreduzierung Spittelwasser" zunächst von Maßnahmen berichtet, die ihr Auftraggeber LAF aus Gründen der Verhältnismäßigkeit oder anderen Motiven (u.a. Bearbeitung durch andere Auftragnehmer) nicht akzeptieren würde; am Ende der Studie steht "eine Prüfung darüber hinausgehender Maßnahmen wird durch die Verfasser empfohlen." Vermutlich ist Ihrem Team entgangen, dass die Autoren durchaus verwertbare Hinweise gegeben haben, insbesondere auf die "Auenuntersuchungen mittels Gamma­spektrometrie", u.a. mit der Feststellung "prinzipiell wären hier somit punktuell fokussierte Maß­nahmen denkbar" und Angaben zur Prozessen und genauer Lokalisierung.

Nun liegt uns aus dem Internet ein Bild vor, eine Vortragsfolie (beigefügt), bei der man sehr schön die Belastungsschwerpunkte in den Auen entlang des Spittelwassers verfolgen kann. Die gelb-rötlichen Bereiche von 3000-6000 ng TEQ/kg (0 bis 0,3 m unter Geländeoberkante) machen in der Zone 2 etwa 1/3 der Fläche von 32,9 ha aus, d.h. rd. 100.000 qm oder - bei der angenommenen Tiefe von 0,3 m - 30.000 Kubikmeter Sediment/Fluvisol; das wären bei 1 kg = 1 cubic-dm bei Mittelung der Konzentrationsdaten 0,135 kg TEQ Dioxin (Jacobs et al. geben in der Tabelle 6.10 auf Seite 198 0,28 kg TEQ WHO für die gesamte Zone 2 an. Insgesamt kommen Jacobs et al. bei allen fünf Zonen und einer Fläche von 133,8 ha auf 0,61 kg TEQ WHO. Soviel zu "links und rechts neben dem Spittelwasser".

"Im Spittelwasser" wurden 1997/2000 vom Staatlichen Amt für Umweltschutz in Dessau 20.000 Kubikmeter dioxinreiches Sediment vermutet/geschätzt mit durchschnittlich ca. 20.000 ng TEQ/kg TS.; das wären 0,4 kg TEQ Dioxin, das durch die Untätigkeit der LAF und des MLU (Machbarkeitsstudie von 1993 ab 2001 in Sachsen-Anhalt nicht weiterverfolgt, 20,9 Mio. DM eingespart) vermutlich bei dem Extremhochwasser von 2002 mobilisiert und weggeführt wurde (Jacobs et al. 2013/14). Es gibt keine Beweise, dass genau die alten Spittelwassersedimente nun links und rechts in den Spittelwasserauen liegen. Aber fest steht, dass aufgrund der Dioxin­konzentrationen die auf den gelb-rot bezeichneten Flächen abgelagerten Sedimente/Fluvisole ebenso sanierungsrelevant sind wie die bis 2000 im Spittelwasser überwachten Sedimente.

Mit freundlichen Grüßen

(Prof. Dr. Ulrich Förstner)

cc Dr. Patrick Jacobs, Tauw GmbH Berlin


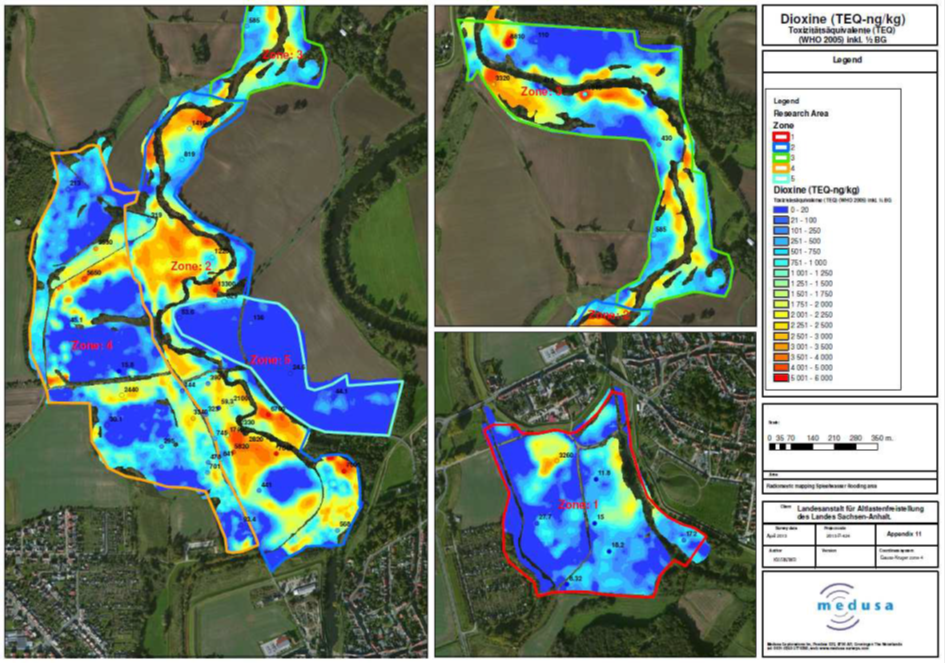


Jacobs P (2013) Frachtreduktion Spittelwasser – Machbarkeitsstudie im Auftrag der Landes­anstalt für Altlastenfreistellung Sachsen-Anhalt. Folienserie anl. Workshop „Sedimentmanage­ment der FGG Elbe“, Folie 13. Hamburg, 17. Dezember 2013. Tauw GmbH NL Berlin [95]

**8. Dioxin-Maßnahmen im Elberaum: Fortschritte und Defizite im internationalen Vergleich**

Für den Aufstieg der Sedimentpraxis in Europa und speziell im Einzugsgebiet der Elbe war unter der Wasserrahmenrichtlinie das ursprünglich forschungsnah-pragmatisch und dann stärker administrativ orientierte europäische Sedimentnetzwerk SedNet [96, 97] wichtig. Ende 2006 übernahm Dr. Peter Heininger (Bundesanstalt für Gewässerkunde [98]), die Rolle des Rapporteurs für das Beispiel Elbe in der SedNet-Studie „Sediment Management: an essential element of River Basin Management Plans“ [99]; er organisierte das Hintergrundpapier „Ableitung überregionaler Bewirtschaftungsziele – Schad­stoffe“ einer Ad hoc Gruppe der Flussgebietseinheit Elbe [53] und leitete ab Herbst 2009 das „Sedi­ment­­managementkonzept“ Sachsen-Anhalt, der Schlüsselregion im IKSE-Konzept [58, 59, 100].

Während der „Sedimentmanagementplan Rhein“ der Internationalen Kommission zum Schutz des Rheins ([59], Kapitel 9) die Belastungssituation zwischen Ober- und Unterliegern, speziell zwischen Staustufen im Oberrhein und den Entsorgungsprobleme im Hafen von Rotterdam, in den Mittelpunkt stellte und dabei bereits konkrete Problemlösungen entwickelte, war durch den Widerstand der LAF gegen den Entwurf der „Risikostudie Elbe“ [43] vom Ende November 2007 (Abschnitt 5.1), gefolgt unter anderen von den eigenmächtigen Veränderungen an FGG-Dokumenten durch LAF/FGG-Elbe (Abschnitte 5.2 und 5.3) eine vergleichbare Entwicklung im Einzugsgebiet der Elbe zunächst blockiert.

Die FGG Elbe hat ab 2011 die Arbeiten an ihrem Sedimentmanagementkonzept intensiviert und begonnen, umfassende Untersuchungsprogramme im Vorfeld von Maßnahmen durchzuführen: die Bestandsaufnahmen hinsichtlich der Mengen, Schadstoffkonzentrationen und Erosionsstabilitäten von Sedimenten in typischen Ablagerungsbereichen, u.a. Auensedimente [101], Buhnenfelder der Elbe [102], Elbe-Seitenstrukturen (Häfen, Altarme, Buchten, Altwässer [103]), im Muldestausee [104] sowie Hauptgewässer, Seitenstrukturen und Nebenflüsse der Saale [105]. Bei den Letztgenannten sind von besonderem Interesse die Untersuchungen an Kernprofilen von Altsedimenten des Saale-Neben­flusses der Bode [106](Kasten 6) .

***8.1 Kriterien zur Auswahl und Priorisierung von Maßnahmen (FGG Elbe, Anonym 2013 )***

Ein besonderes Verdienst des nunmehr veröffentlichten *Sedimentmanagementkonzeptes* der FGG Elbe bzw. der Internationalen Kommission zum Schutz der Elbe (IKSE [58,59]) besteht darin, dass es vorab die Kriterien zur Auswahl und Priorisierung von Maßnahmen festgelegt hat. Im Kapitel 5.3 wurde bereits das *Kriterium 1 „Priorität bei der Quellsanierung“* erwähnt. Das Kriterium 7 bei den All­gemei­nen Kriterien (7.1 auf Seite 54) lautet „das Ausschlusskriterium ‚Fehlen verhältnismäßiger Lösungs­möglichkeiten‘ wird nur im Ausnahmefall bei sehr gut gesichertem / begründeten Kenntnis­stand ange­wandt“; es zielt vermutlich auf die LAF und deren Absicht, von diesem Ausschlusskriterium und den Ausnahmeregelungen reichlich Gebrauch machten (Abschnitt 7.2).

**Kasten 6: Ein neuer Fall in Sachsen-Anhalt: Dioxine in den Sedimenten der Bode [106, 107]**

Bei den Erhebungen, die zwischen 2011 und 2013 im Auftrag des Landesbetriebs für Hochwasser-schutz und Wasserwirtschaft in Sachsen-Anhalt durchgeführt wurden, sind die Untersuchungen an Sedimentprofilen^[[10]](#footnote-10)^ von Altsedimenten des Saale-Nebenflusses der Bode von besonderem Interesse: es wurden ca. 37.500 t Ablagerungen unterschiedlicher Konsistenz festgestellt, wovon nach den Erhebungen des IWS Universität Stuttgart^[[11]](#footnote-11)^ ca. 75% mobilisierbar sind.

Analysendaten von drei Probeentnahmestationen an der Bode (Tabelle 8) zeigten deutlich erhöhte bis sehr hohe Dioxinkonzentrationen. Die höchsten Dioxingehalte – 2220 bzw. 6650 ng I-TEQ/kg – wurden in den tieferen Proben der Kerne aus den Ablagerungen flussabwärts von Staßfurt gefunden, wo von 1940 bis 1945 eine Produktionsstätte für Leichtmetalle stand (Abschnitt 2.1). An der Probenahmestation „Eisen­bahnbrücke Löbnitz“ fanden sich die höchsten Gehalte in einer mittleren Sedimenttiefe von 0,1 - 0,5 m. Eine weitere Station flussabwärts, in der Alten Bode bei Neugatters­leben, lagen die Dioxin­konzen­trationen nur noch um das 2,5fache bis 3,5fache über dem oberen Schwellenwert von 20 ng I-TEQ/kg für Dioxin. Daraus ergibt sich das Bild eines fortwährenden Flussabwärtstransports von dioxinreichen Sedimente, das sich auch noch in den Sedimenten der unteren Saale verfolgen lässt.

Eine kontrollierte Entfernung schadstoffhaltiger Sedimente, die am Spittelwasser bisher nicht stattfand, wird für die Saale zumindest angedacht. Die G.E.O.S. Studie [106] sieht solche Möglichkeiten im Zusammenhang mit dem Rückbau funktionsloser Wehre u.a. im Bereich der Bode

**Tabelle 8** Dioxin in Sedimenten (Altsedimenten) der Bode (Anonym [106]. Zum Vergleich: a) Spittel­wasser mit einer (groben) Abschätzung der Sedimentmengen (ConSoil Case Study 2000 [20]; Spittel­wasser (Anonym [16]) enthält den Befund, dass ein Großteil der Hot Spot Sedimente aus dem Jahr 2000 [15] ausgeschwemmt wurde, vermutlich während des Augusthochwassers 2002.

| Flussbereich | Datenbank ID GEOS | Probenahme- datum | Entnahme­tiefe (m) | I-TEQ | Menge (t) |
| --- | --- | --- | --- | --- | --- |
|  |  |  |  | ng/kg TR | Länge (km) |
| zwischen Hohenerxieben und Staßfurt | Bo5b | 12.06.2012 | 0,0 – 0,1 m | 95,5 | 3.266 t über 3,5 km |
|  | Bo5b | 12.06.2012 | 0,1 – 0,5 m | 57,7 |  |
|  | Bo5b | 12.06.2012 | > 0,5 | 2222,8 |  |
|  | Bo5b | 04.01.2013 | > 0,5 | 6650,0 |  |
| Eisenbahnbrücke Löbnitz | Bo4c | 19.06.2012 | 0,0 – 0,1 m | 356,8 | 3.050 t über 2,0 km |
|  | Bo4c | 19.06.2012 | 0,1 – 0,5 m | 1145,5 |  |
|  | Bo4c | 19.06.2012 | > 0,5 | 248,3 |  |
| Alte Bode Neugattersleben | Bo4a | 19.06.2012 | 0,0 – 0,1 m | 54,3 | 15.930 t über 1,0 km |
|  | Bo4a | 19.06.2012 | 0,1 – 0,5 m | 50,8 |  |
|  | Bo4a | 19.06.2012 | > 0,5 | 68,4 |  |
| Spittelwasser ^a)^ |  | *2000* |  | *~ 20.000* | *~ 20.000 t* |
| Spittelwasser (LAF 2013) |  | 2013 |  | 750 | 2.700 t |

In den *Aspekt Qualität* gehen der Schutz der Umwelt und der menschlichen Gesundheit ein (Kapitel 3, Anlage A2-3 in [58, 59]). Es wird eine Aufteilung der Elbe-relevanten Schadstoffe in zwei Gruppen vorgenommen; Gruppe 1 enthält alle Stoffe, die zum Schutz der menschlichen Gesundheit explizit geregelt sind; Gruppe 2 alle anderen. Zur Gruppe 1 (Regelungsebene „e“ in Anlage A2-3[58, 59]) gehören As, Cd, Hg, Pb, HCHs, HCB, Benzo(a)pyren (PAK) und Dioxine/Furane. Unter Beachtung dieser Einteilung kommen folgende Kriterien zur Anwendung:

Eine erste Klassifikation – hier für Dioxine/Furane – folgt dem Kriterium (i): *Je höher im Ranking für eine Herkunftsregion* (hier für “Sanierung Altlasten” wie Spittelwasser bzw. für “Beseitigung von Altsedimentdepots“, letzteres für Bode, Saale oder Elbe, oder für einen Quelltyp: Seitenstrukturen, Stauhaltungen, Sedimentationszonen, Buhnenfelder usw. *desto dringlicher ist die Empfehlung*. Das Kriterium (ii) beruht auf der die Zahl der pro Quelle relevanten *Schadstoffe der Gruppe 1*; je mehr Stoffe dieser Art eine Quelle aufweist, desto dringlicher die Handlungsempfehlung. Kriterium (iii) bezieht sich auf die Zahl der pro Quelle relevanten *Schadstoffe der Gruppe 2*; je mehr Schadstoffe eine Quelle aufweist, desto dringlicher die Handlungsempfehlung. In einem vierten Schritt erfolgt die *Anwendung der allgemeinen Kriterien 1 – 7* (die allgemeinen Kriterien 1-4 wirken *aufwertend*, die Kriterien 5-7 tendenziell *abwertend* für die Relevanz einer Maßnahme):

1. Die Lösung eines Problems an der *Quelle* bzw. die Beseitigung der *Ursache* ist zu bevorzugen.

2. Besteht die ursächliche Quelle nicht mehr, sollte die Lösung *möglichst quellnah* erfolgen (*„die Treppe von oben reinigen“*).

3. Resonanzwirkung 1: Empfehlung wirkt sich positiv auf einen der beiden *anderen Aspekte* oder auf beide gleichzeitig aus.

4. Resonanzwirkung 2: Einmalige Investition bewirkt *dauerhaft geringere Folgekosten*.

5. *Schwierigkeitsgrad* / Aufwand der Realisierung.

6. Sicherheit / Unsicherheit in der Abschätzbarkeit der *Erfolgsaussichten*, z.B. infolge Variabilität des Systems.

7. Das *Ausschlusskriterium „Fehlen verhältnismäßiger Lösungsmöglichkeiten“* wird nur im Aus­nahme­fall bei sehr gut gesichertem / begründetem Kenntnisstand angewandt.

Einen erstaunlichen Satz findet man am Ende dieser Übersicht *Allgemeine Kriterien*: *“Der Aspekt der technischen und wirtschaftlichen Machbarkeit ist generell bei der Aufstellung der Maßnahmen­pro­gramme für den 2. Bewirtschaftungsplan zu prüfen”!* Was hat man denn früher gemacht – vor der Aufstellung derartiger Kriterien?

In der Tabelle 9 wird auf der Grundlage dieser Kriterien eine vorläufige Aufstellung für prioritär dioxin­haltige Sedimentaltlasten gegeben (die *englische Version* enthält wesentlich mehr Details).

| Herrkunftsregion (Quell-Typ) | Stoffe Gruppe 1 (Gesundheit) | Quelle Direkt | Quelle  Nähe | Reso-  Nanz | Schwierig-keitsgrad | Erfolgs­potenzial |
| --- | --- | --- | --- | --- | --- | --- |
| *Sanierung kontaminierter Standorte innerhalb und entlang von Flüssen, Bächen und Kanälen* | | | | | | |
| Mulde/Spittel-  wasser ÖGP Bitterfeld-Wolfen | **1**  α-, β-, γ-HCH *Dioxine/Furane* | ja | - | ja | sehr hoch | ?? |
| *Entfernung von Zwischenlagern von kontaminierten Sedimenten* | | | | | | |
| Saale  (Seitenstrukturen) | Hg, Cd, Pb  α-, β-, γ-HCH Benzo(a)pyrene  *Dioxine/Furane* | nein | ja | nein | mittel | ?? |
| Saale  (Staustufen) |  | nein | ja | nein | mittel | ?? |
| Untere Bode  (Sedimentations-zonen) | **1**  *Dioxine/Furane* 4: Pb | nein | ja | nein | mittel | ?? |
| Elbe (0-300 km)  (Seitenstrukturen) | Hg, Cd, Pb, As  α-, β-, γ-HCH HCB, B(a)pyrene  *Dioxine/Furane* | nein | nein | nein | mittel | hoch |
| Elbe (0-350 km)  (Buhnenfelder) |  | nein | nein | nein | mittel | ?? |

**Tabelle 9**: Kriterien zur Auswahl und Priorisierung von Empfehlungen hinsichtlich Qualitätsaspekte bei der nachhaltigen Behandlung von Sedimenten und Baggergut (nach „Sedimentmanagement­konzept der Flussgebietsgemeinschaft Elbe“, Anonym 2013b,[58, 59] Table 6-6)

Dioxine/Furane treten demnach im Elberaum nach diesen Kriterien vier Mal in hoher Priorität auf:

- Im System Mulde/Spittelwasser im Ökologischen Großprojekt Bitterfeld-Wolfen als Altlast zusammen mit α-, β-, γ-HCH in Priorität 1, bei sehr hohem Realisierungsaufwand. *Hier verdienen vor allem die in der Tauw-Studie beschriebenen Ergebnisse der radiometrischen MEDUSA-Untersuchungen an Überflutungssedimenten entlang des Spittelwassers Beachtung (Kapitel 7.3)!*
- In den Seitenstrukturen und Stauhaltungen der Saale als Altsedimentdepots zusammen mit Hg, Cd und Pb, α-, β-, γ-HCH und Benzo(a)pyren in Priorität 2a u. 2b, bei mittlerem Aufwand
- In den Sedimentationszonen der Unteren Bode in Priorität 1, mittlerer Aufwand
- In den Seitenstrukturen (km 0-300 km) und Buhnenfeldern (km 0-350 km) der Elbe zusammen mit Hg, Cd, Pb und As, α-, β-, γ-HCH, HCB und B(a)pyren in Priorität 1a und 1b, Seitenstrukturen (Häfen, Altarme mit mittlerem Schwierigkeitsgrad, aber teilweise guten Erfolgsaussichten). *Dazu passen die im Abschnitt 9.3 dargestellten Untersuchungen zur Kombination von Subaquatischen Deponierung und Capping von dioxinhaltigen Sedimenten mit dem Beispiel Hitzacker-Elbe.*

***8.2 Fortschritte im Sedimentmanagementkonzept, Defizite bei Maßnahmen im Elberaum***

Es muss darauf hingewiesen werden, dass die meisten dieser Aktivitäten unter dem breiten Begriff *„Management“* („Bewirtschaftung“) mehr oder weniger weit im Vorfeld der eigentlichen „Maßnah­men“ bzw. „Problemlösungen“ stehen [108]. Bis heute sind auf diesen Sachverhalt die Berichte zum Start der Maßnahmenphase der Wasserrahmen­richtlinie, insbesondere der *Katalog der Länderarbeits­gemeinschaft Wasser* (LAWA) mit seinen 99 umsetzungsbezogenen und 8 konzeptuellen Maßnahmen [33], noch nicht eingegangen. Einen ersten Anhaltspunkt zu sedimentbezogenen Sanierungsmaßnah­men, wie sie zur Umsetzung der WRRLim deutschen Teil der Flussgebietseinheit Elbe für notwendig erachtet wurden, gab die *Auftragsstudie der Hamburg Port Authority und der FGG Elbe* ([93], Kap. 6, S. 237-332).

In den großen sedimentnahen Problembereichen im Elberaum sind die Sanierungsmaß­nahmen, die nach der Wende im *Braunkohletagebau* und bei den *Hinterlassenschaften der sowjetischen Uran­gewinnung* – über 300 Mio. m³ Haldenmaterial und 160 Mio. m³ radioaktive Schlämme – mit einem Kostenaufwand von letztlich jeweils etwa 10 Milliarden € durchgeführt worden sind, inzwischen nahezu abgeschlossen [93]. Im Hamburger Hafen war in Kooperation mit dem *Institut für Partikel­technologie der Technischen Universität Hamburg-Harburg* in den frühen 1980er Jahren eine weltweit führende Verfahrenskombination für die Aufbereitung kontaminierter Sedimente entwickelt worden [109]. Das hatte aber nach der politischen Wende keinen Widerhall im weiteren Einzugsgebiet gefun­den (eher die Erwartung, dass die „Nachreinigung“ der Elbe ohnehin in Hamburg stattfindet).

Während am Rhein durch die niederländischen Schlickinseln relativ kostengünstige Problemlösungen entwickelt wurden, die auch für dringende Situationen im Binnenland genutzt werden konnten (siehe Kasten 8), wurde für den Elberaum oberhalb Hamburg die Frage nach Maßnahmen immer weiter in die Zukunft verschoben^[[12]](#footnote-12)^. Die Hinweise auf die Konzepte für naturnahe Sanierungsmaßnahmen, die u.a. in dem *amerikanischen Superfundprogramm* mit großem Aufwand und unter Mitarbeit führender Fachwissenschaftler umgesetzt werden [111], wurden bei uns schnell als *„U.S.-lastig“* abgetan. Eine solche Meinung erscheint recht überheblich angesichts der Tatsache, dass sich die amerikanische Umweltbehörde seit über 20 Jahren in steigender Intensität mit Sedimenttechnologien befasst und zur Zeit allein im Superfund Programm weit über 100 größere Fälle bearbeitet werden [112-120].

***8.3 Vergleiche mit Maßnahmen bei Dioxin-Altlasten im UK, in Finnland und in den USA***

Nach der *Durchsicht der internationalen Literatur* gibt es drei Beispiele für lohnende Vergleiche mit den Sedimentaltlasten in der Bitterfeld-Regionen und deren Auswirkungen auf nachgelagerte Fluss­gebiete. Auch bei diesen Beispielen wurden *extreme Dioxinbelastungen ebenfalls zu Beginn der 1990er Jahre* bekannt – die ganz unterschiedlichen Ursachen spielen in den weiteren Verfahren keine größere Rolle – und haben zuerst umfangreiche Monitoringaktionen nach sich gezogen, die inzwischen abgeschlossen sind. Was hier besonders interessiert sind Maßnahmen im Sinne von Problemlösungen, und dabei könnten die Unterschiede in den drei Beispielen nicht größer sein.

1. ***River Doe Lea, Rother R, Don R. (Derbyshire, UK; [121])***

Die Dioxinbelastung des Flüsschens Doe Lea, die von der Firma Coalite (Bolsover), einem Hersteller von verschiedenen Chemikalien auf der Grundlage von Kohleteer, ausging, erreichte nach 9 km den Fluss Rother, nach 29 km den Fluss Don; von dort ging es weiter, mit abnehmenden Konzentrationen, über die Flüsse Ouse und Humber zur Nordsee. Die Tabelle 10 zeigt die räumlichen (flussabwärts) und zeitlichen Entwicklungen der Dioxingehalte in den Flusssedimenten während der 1990er Jahren.

| **Location** | **River** | **km from source** | **Oct 1991** | **Mar 1993** | **Mar 1995** | **Feb 1996** | **Feb 1997** | **Sep 1997** |
| --- | --- | --- | --- | --- | --- | --- | --- | --- |
| Coalite Chemicals | Doe Lea | U/S | 10 | 9 | 18 | 11 | 15 | 15 |
| Buttermilk Lane | Doe Lea | 0 | 64000 | 45300 | 1200 | 550 | 290 | 180 |
| Netherthorpe | Doe Lea | 4.4 | 26000 | 12300 | 450 | 330 | 540 | 540 |
| Remishaw | Rother | 9.0 | 15000 | 300 | - | 79 | 53 | 53 |
| Canklow | Rother | 27.1 | 17000 | 426 | - | 93 | 96 | 360 |
| Rotherham | Don | 29.3 | 570 | - | - | 180 | 310 | 140 |
| Kilnhurst | Don | 37.0 | - | - | - | 110 | 140 | 110 |
| Thome | Don | 74.9 | - | - | - | 9 | 8 | 9 |

Tabelle 10: Dioxinkonzentrationen in ng/kg I-TEQ für die Flüsse Doe Lea, Rother und Don flussabwärts von der Firma Coalite Chemicals (Edwards [122] nach Environment Agency).

Im Februar 1995 gab es ein größeres *Hochwasserereignis*, bei dem die Dioxinkonzentrationen im Doe Lea Fluss um ca. 95 % reduziert wurden; sie lagen aber immer noch um das 10- bis 50fache über den Hintergrunddaten für Dioxin in den Flüssen Großbritanniens (AEA Technology [123]).

Die wechselvolle Geschichte von Überlegungen zur Sanierung der Doe Lea Region wird im Kasten 7 (nach Stevens [121], eigene Unterstreichungen betreffen Maßnahmenvorschläge) nachgezeichnet. Am Ende steht als „Best Practical Environmental Option“ eine *Non-Intervention* [124, 125]. Trotz der Probleme mit landwirtschaftlichen Produkten [126] fehlt in der Übersichtsstudie über Doe Lea Region von 2009 [127] im Kontext mit Umsetzung der WRRL jeder Hinweis auf das Thema *„Dioxin“*.

**Box 7: Why is the water quality in the River Doe Lea so poor? A dioxin case in UK [121]**

The pollution was first noticed in the summer of 1991 when locally produced milk was found to be contaminated with dioxins. The source of the pollution was believed to arise from a leak at the biological effluent treatment plant (BETP) at Coalite Chemicals. Coalite Chemicals produced by-products from the coal readily mined in the close vicinity. The NRA (1995) report that this included creosote, chlorinated oils and chlorinated phenols; concentrations within the activated sludge of the Coalite BETP were 210000 i-TEQ ng/kg and as a result, a solid case against Coalite Chemicals was formed (NRA, 1995 [124]).

The hydrological characteristics of rivers caused the plume of dioxins to spread downstream into the Sheffield and South Yorkshire Navigation (SSYN). Advection of sediment downstream of the Doe Lea, Rother and Don resulted in deposition of dioxin sediment in the low velocity areas of the waterways. NRA [124] suggested that the highest dioxin concentrations had settled behind the abundance of weirs in the network and that this supported the „do-nothing‟ approach to remediation.

In order to treat the dioxin pollution upon its occurrence in 1991, nine management options were considered in depth to control the dioxin pollution in the river. These included: (1) In situ „do nothing‟ approach, (2) water injection dredging, (3) capping, (4) disposal to British Waterways landfill, (5) disposal to commercial landfill, (6) thermal treatment, (7) detoxification, (8) Humber disposal and (9) bioremediation.

Scott Wilson[125], whilst discussing the relative merits of a method of non-intervention, suggested that river dredging and landfill was the BPEO for the polluted sections of river. The report concluded that dredging and landfill disposal offered the *‘most rapid progress, lowest overall risk, lowest cost solution and with acceptable risks’*.

A paper by British Waterways (in [121]) agreed with the BPEO suggestion of Scott Wilson [125], though questioned the feasibility of dredging the non-navigable sections of the river. From personal study, it is clear that accessibility to the river can be problematic due to excessive overgrowth of rural areas and extensive engineered channelisation of the river in the industrial lower reaches. British Waterways did conclude however, that landfilling options would provide the least risk to human health. Whilst raising concerns of the liability of British Waterways owning the landfill site and future concerns; it was concluded in agreement that dredging and landfill would be the BPEO for this case.

Modelling work undertaken by Scott Wilson [125] found that the recovery time for the „do-nothing‟ approach was much longer for navigable sections of the waterway. The reduced velocity and advective fluxes of navigable sections would ensure remediation took decades and greatly increased the risk of a dioxin contaminated plume expanding over an even larger area of the Sheffield and South Yorkshire Navigation (SSYN, Scott Wilson [125]). The report continued to conclude how bioremediation was unfeasible due to the *‘practical implementation of such a scheme to a problem of this magnitude’*.

In 1998, the Environment Agency completed a follow up sampling investigation and found that dioxin levels had again built up in the BETP of the Coalite site. Whilst not contributing to the watercourse, the potential for another outbreak was increased as the higher concentrations of dioxins were again present. The EA concluded that there were still sources of dioxin production on site and included this into their management package (Environment Agency, 1998 in [121]).

As the pollutant was advected downstream, the concentrations within the Doe Lea reduced to manage­able levels. The problem was by no means solved as sediment deposition of dioxin pollutants in slow flowing waterways downstream now have the dioxins embedded within the river bed. The reduced levels were deemed to not to pose a significant risk to the aquatic environment. A reduction of 70% dioxin concentration was seen in the Doe Lea from 1992 to 1996 (Environment Agency, 1997 in [121]). This resulted in the do-nothing recommendation being suggested by NRA [124] but to continue further investigation and long term monitoring of sediment.

1. ***River Kymijoki, South-Eastern Finland [128])***

Sediments from the River Kymijoki, draining into the Gulf of Finland, have been heavily polluted by the pulp and paper industry and by chemical industries. A wood preservative, known as Ky-5, was manufactured in the upper reaches of the river between 1940 and 1984 causing severe pollution of river sediments with polychlorinated dibenzo-p-dioxins (PCDD) and dibenzofurans (PCDF). The total volume of contaminated sediments was estimated to reach 5×10^6^ m³ and hot spots with extremely high concentrations (1,060 ng I-TEQ g^−1^ d.w.) were located immediately downstream from the pollution source (approximately 90,000 m³). Sediment contamination was accompanied by changes in benthic assemblages, but direct effects were masked by many factors.

The total burden of Ky-5 derived PCDD/Fs was estimated as ca 5,960 kg (17.3 kg as I-TEQ) in the river and 1,770 kg (12.4 kg as WHO-TEQ) in the Gulf of Finland (Fig. 3). The total historical sum of approximately 29 kg (I-TEQ) is comparable with an approx. equal sum (20 kg I-TEQ) distribu­ted with the Ky-5 product to the sawmill sites in Finland and exceeds the annual atmospheric emissions of 17 western European countries of approximately 13.7 kg/year in 1985 and from 2.0 to 3.7 kg/year in 2005. Most of the PCDD/Fs eroding from the hot spot area accumulate in the lower course of the river. The annual PCDD/F flux to the Gulf of Finland has been estimated to vary from 13 kg to 24 kg/year (44–75 g/year I-TEQ) and the load to the gulf as 1,770 kg or 12.4 kg WHO-TEQ, see Fig. 3).

With no remediation 25–50% of the dioxins deposited in the immediate sedimentary basins would be transported downstream within the next 30 years. Removing and/or capping these sites (36,000 m^3^, 40% of contaminated sediments between the source and Keltti would significantly reduce the PCDD/F transport downstream. The highest transport percentages indicate circumstances of increased high flows (probability of one occurrence within 100–500 years based on data from 30 years of monitoring) because of climate change (see, e.g., Weber et al. 2008 [129]).

***
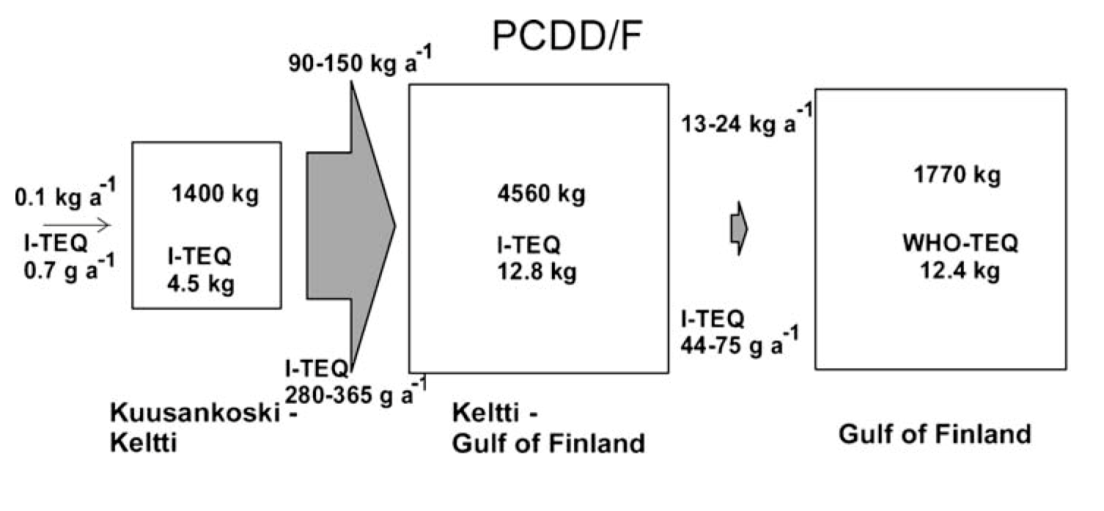
***

**Fig. 3** A schematic diagram showing the burden of PCDD/Fs in different river regions and annual transport between the pools and to the Gulf of Finland in 2001. The size of the arrows is proportional to the quantity (Verta et al. 2008 [128])

1. ***NE United States: Upper Hudson River (PCB) und Lower Passaic River (Dioxin)***

Unter den Projekten zur Sedimentsanierung, die von der U.S.-amerikanischen Umweltbehörde im Rahmen des *Superfundprogramms* seit den 1980er Jahren genehmigt und geplant wurden, ragen die beiden Projekte am *Hudson River* (Hauptsanierungsphase bis 2016) und am *Passaic River* (ab 2016) aufgrund ihrer Kosten von deutlich *über einer Milliarde U.S.-Dollar* heraus.

*Hudson River*. Seit den 1940er Jahren bis 1977 hat die Firma General Electric polychlorierte Biphenyl (PCB) als Isolierflüssigkeiten bei der Herstellung von Kondensatoren und Transformatoren eingesetzt und dabei sind ca. *70.000 kg* der hochgiftigen Substanzen in die Sedimente des oberen Hudson River gelangt. Nach einem „No-Action-Beschluss“ in 1984 begann die U.S.-Umwelt­behörde 1990 mit einer umfassenden Bestandsaufnahme, die im Jahr 2002 zu der Entscheidung führte, dass General Electric für die Sanierung von „Hot-Spot-Sedimenten“ auf einer *Flußstrecke von 40 Meilen von Ft. Edward bis Troy* am oberen, nicht tidebeeinflussten Hudson verantwortlich sein sollte. Zwischen 2009 und 2014 wurden in zwei Phasen und drei Flussabschnitten *~2 Mill. Kubikmeter PCB-kontaminiertes Sediment* ausgebaggert und entwässert; 7.6 % der Fläche erhielt*„Capping“* (Übersicht *„Hudson River PCBs Summer 2014“* [130] einschließlich *„Environmental Dredging“* [131]: *Where Are We?* [132]).

*Passaic River.* Der Lower Passaic hat mit *~29 kg 2,3,7,8-TCDD in ca. 5 Mill. m³ kontaminiertem Sedi­ment* ungefähr 80 % der Dioxinfrachten in den *Newark Bay Complex* im Staat New Jersey eingetragen [134]; an diesem Abschnitt befindet sich die *Diamond Superfund Site*, wo in den 1940er Jahren die Produktion u.a. von DDT begonnen hatte [135]. In den 1950er und 1960er Jahren wurde die Anlage zur Herstellung des *Entlaubungsmittels „Agent Orange“* genutzt ([136], Bopp et al. [137]); dabei ent­standen *8 kg 2,3,7,8-TCDD,* die im Fluss entsorgt wurden.

Nach den Untersuchunge der U.S.-Umweltbehörde EPA (Übersicht *Lower Passaic River Restoration Project* [138]) wurde der *Standort 1984 auf die Nationale Prioritätenliste* gesetzt. Im Jahr 1987 star­tete die EPA eine vorläuige Sanierung an der Lister Avenuue Site, bestehend aus einer Abdichtung mit Capping und Untergrundschlitzwänden sowie der Sammlung und Behandlung von Grundwasser. Die Informatonen aus einer neuen großeren Studie zeigten, dass die untersten 13 Fluss-km bis zu einer Sedimenttiefe von 5 m die Hauptverschmutzungsquelle für den 27 km langen Unteren Passaic sind. Am 11. April 2014 präsentierte die *EPA einen „Vorschlagsplan“* mit ihren bevorgten Sanierungs­maßnahmen für den höchstbelasteten Abschnitt, „*Capping mit Baggern für Hochwasserschutz und Schiffahrt“* mit einer *„Off-Site Lagerung der Baggerguts“*. Die Kosten für diese Alternative 3, bei der das Krebsrisiko aus dem Verzehr von Flussfischen und –krebsen in den kommenden dreißig Jahren um den Faktor 10 verringert werden sollte, würde 1,73 Milliarden U.S. $ betragen [139, 140]. Es gibt inzwischen einen Alternativplan „Sustainable Remedy“ der sich auf die Sanierung von 25 Hot Spots konzentriert, nur ca. 40% des EPA-Plans kosten würde und von ca. 100 Firmen unterstützt wird.

**Box 8: 2014 Hudson River PCB Forum, Marist College Boathouse, November 11, 2014 [133]**

*Welcoming Remarks:* Dutchess County Executive, Marcus Molinaro opened the 2014 Hudson River PCB Forum by taking us back to his days as a Village Trustee of Tivoli. He explained that in the early 1990s Tivoli, like many other Hudson River municipalities, had disregarded the value of the Hudson River as their “backdoor” and were more focused on consumer-related aspects of economic growth. He explained that soon after this he realized that the Hudson River was the unifying factor for the Hudson Valley and the connection between the upriver and downriver communities, despite their disagree­ments about the proposed clean up. He called for a comprehensive cleanup of the Hudson River because it has given and will continue to give life to New York State. The Hudson River is a great environmental resource with an important heritage and history to preserve. He stressed the importance that the Hudson River as a tourist location – another important reason to invest and protect the river for the current and future generations.

*Unfinished Business.* Andy Bicking, Director of Public Policy, Scenic Hudson, expressed his appreciation for the Marist professional staff, Mark Molinaro and his fellow organizations Clearwater, Riverkeeper, and NDRC. Communities and organizations along the Hudson River have watched the PCB clean up for the last five years, and now, within the next 18 months the legacy GE will leave of the Hudson River will be decided. Researchers have concluded that if necessary steps, such as cleaning the 136 acres of contaminated water sediments and dredging of the Champlain Canal, are taken now, the water quality and overall health of the Hudson River will be greatly improved. He continued by saying how the 1.3 million pounds of PCBs that were dumped into the Hudson River from the 1940s to 1976 have brought an end to Hudson River fish consumption and to the fishing industry, as well as preventing the NYS Canal Corporation’s from honoring its obligation to dredge navigational channels. Mr. Bicking concluded by explaining how it makes perfect sense to address all of these issues before dismantling the facilities and equipment to free the Hudson River from contamination.

*Where Are We?* Abigail M. Jones, Staff Attorney for Riverkeeper and CAG member, gave an update on the status of the current in-river PCB cleanup by GE, who is entering into its final year of dredging and will be completely out of the river, including decommissioning its support facilities, by 2016. She also spoke about the NYS Department of Health fish consumption advisory which has limited or prohibited human consumption of PCB-contaminated fish from the Hudson River since the late 1970s. Ms. Jones presented a brief overview of the timeline of the present cleanup and noted that, in the five years GE has been dredging in the Hudson River, the company has dredged far more PCB-contami­nated sediment than it expected and with little to no exceedances of water and air quality, as monitored by EPA. Ms. Jones also noted that GE and EPA are in the beginning stages of drafting a cleanup plan for the “floodplains” area of the Hudson River, and that Riverkeeper and the other environmental organization will be involved in this process – and also encourage the public to get involved – to ensure that the floodplains cleanup is sufficiently protective of human and environmental health.

*Next Steps:* Manna Jo Greene, Environmental Director, Hudson Sloop Clearwater and CAG member, moderated the panel and question and answer discussion. She first focused next steps – on the importance of signing a municipal resolution. She noted that 15 years ago the Friends of a Clean Hudson, a coalition of two dozen river and environmental groups, went out into municipalities and educated the people about the true health impacts, such as neurological damage, from eating Hudson River fish due to PCB contamination. After educating the citizens, 69 municipalities and 150 organizations passed resolutions calling for the remediation of PCB-contaminated hot spots in the upper Hudson. Ms. Greene then transitioned to today’s outstanding issues – the remediation of an additional 136 acres of contaminated sediment as defined by the Federal NRD Trustees as beneficial to the recovery of the river’s natural resources. She also stressed the need to dredge the navigational channel, to ensure full use of the river by deep draught shipping vessels und she explained that it will be in the company’s interest, as well as NYS taxpayers’ interest, for GE to participate in a voluntary settlement, while the remediation facilities are still mobilized and operable.

***8.4 Maßnahmen für die Binnenelbe – Bergbau, Capping, MNA, Stabilisierung (Literatur)***

Die Entwicklung bei der Behandlung von kontaminierten Feststoffen geht zunehmend in die Richtung *naturnaher, relativ kostengünstiger Methoden*. Im Vorder­grund stehen dabei die *passiven In-situ-*Metho­den – das sind Behandlungsverfahren direkt im Unter­grund oder im abgelagerten Sediment ohne Energie­eintrag und mit geringem Gefährdungspotential für das Personal.

1. *Bergbauabfälle und aufgelassene Gruben* stellen aufgrund der enormen Feststoff- und Wasser­men­­gen prioritäre Umweltprobleme dar.
2. Die *In-situ-Abdeckung kontaminierter Sedimente* als Sanierungsmaßnahme kann eine effiziente und wirtschaftliche Alternative zu Multikomponenten-Maßnahmen wie Ausbaggern und Behan­deln bzw. Ausbaggern und Deponieren bieten.
3. Sofern die Beseitigung der belasteten Sedimente nicht aus nutzungsbedingten Gründen erforder­lich ist, besteht die Neigung, diese Ablagerungen im gegenwärtigen Zustand zu belassen – typische Ausgangslage für den Ansatz *„kontrollierter natürlicher Rückhalt und Ab­bau von Schadstoffen“*.

Die drei Maßnahmen lassen sich schwerpunktmäßig *verschiedenen Regionen eines Flussein­zugs­gebiets* zuordnen (Abb. 4): (1) Die Bergbaugebiete mit den *typischen Problemen der sauren Wässer* befinden sich häufig in den Bergbaugebieten im Oberlauf der Flüsse. (2) Mittel­große und kleine Boots­häfen, bei denen für die umweltschonend entnommenen Sedimen­te ein *Unterwasserdepot mit einer aktiven Abdeckung* empfohlen wird, liegen vorwiegend am Unter- oder Mittellauf des Haupt­flusses. (3) ebenfalls im Mittel- und Unterlauf treten verstärkt die *Überflutungs­sedi­mente* auf; ein starker Ein­fluss von Bergbauregionen führt vor allem zur Kontamination mit Schwer­metallen.

***Abb. 3:*** *Sedimentprobleme in einem Flusseinzugsgebiet (nach Shea 1988 [141], [142])*

1. ***Bergbau (aus [93])***

Besondere Aufmerksamkeit gilt weltweit jenen Bergbau-Altlasten, in denen sich – wie bei der Auf­wirbelung von Sedimenten – bei der *Oxidation von Sulfidmineralen* saure Lösungen bilden, die vor allem durch die Mobilisierung von Schwermetallen sowohl die Oberflächen- als auch Grundwässer intensiv und langfristig belasten. Im Elbe-Einzugsgebiet sind davon vor allem die Gewässer in den beiden großen *Braunkohle-Tagebauregionen von Mitteldeutschland und der Lausitz* betroffen [143, 144]; maßgebliche Forschungsarbeiten stammen von *Prof. Uwe Grünewald von der BTU in Cottbus* ([145-147], Abschnitt 6.6.1 in dem Beitrag „Maßnahmen“ der Risikostudie Elbe [93]).

In weiten Bereichen des Mitteldeutschen und Lausitzer Braunkohletagebaus ist die bergtechnische Grundsanierung – die Herstellung der Gewässer – bereits weit vorangeschritten und ihr Ende absehbar; auch die Planung und die *Errichtung der Wasserbauwerke einschließlich der Flutung der Bergbau­folge­seen* sind bezüglich der noch zu treffenden Entscheidungen und ihrer Umsetzung in die wasser­wirtschaftliche Realität transparent [148]. Klärungsbedarf besteht aber noch für die nachfolgenden Zeitpläne, die bergbaubedingte wasserwirtschaftliche Nachsorge: *1. Stabilisierung der Wasserstände* mit einem Zeitrahmen von 5 bis 15 Jahren (Luckner [149]) und *2. Stabilisierung der Wassergüte*, die zwischen etwa 5 bis 25 Jahre (Luckner [150]) in Anspruch nehmen wird.

Unter den vielfältigen *ökotechnologischen Maßnahmen zur* Steuerung des Stoffhaushaltes in Gewässern, die vor allem von Klapper [151, 152] zusammenfassend beschrieben wurden, gibt es auch Methoden zur *„Sedimentkonditionierung“*, d.h. zur Verbesserung der Sedimentbeschaffenheit bezüg­lich der Nähr- und Schadstoffbindung sowie der Besiedelbarkeit für Makrozoen und Makrophyten. Die am häufigsten verfolgte Strategie zielt auf eine *Erhöhung des Redoxpotenzials in der obersten Sedi­mentschicht*, d.h. in der Schlamm-Wasser-Kontaktzone. Aus den tieferen, stets anaeroben Sediment­schichten aufsteigende Fe^2+^- und Mn^2+^-Ionen werden oxidiert [153, 154] und bilden eine *Sperrschicht für Phosphor*, hindern ihn am Austritt ins überstehende Wasser; zugleich werden die Lebensbedingun­gen für Fischnährtiere verbessert.

Zur Restauration versauerter Seen hat sich die *Einarbeitung von Natriumkarbonat* als langzeitig wir­kendes Neutralisationsmittel gegenüber der Alkalisierung mittels Branntkalk als überlegen erwiesen. Insgesamt hat sich die *Lagerung von sulfidreichen Abraummaterialien unter Wasser* als langfristig beste Methode zur Verhinderung einer Metallfreisetzung erwiesen [155]. Es gibt keine Anzeichen, weder von chemischen Daten noch von mikroskopischen Untersuchungen an derart gelagerten sulfidi­schen Bergbauresten, dass Sulfidphasen unter diesen Bedingungen auf dem Seegrund chemisch reagie­ren. Von besonderer Bedeutung ist der Befund, dass dies *auch für sehr flache Seen* gilt, in denen diese Ablagerungen häufig aufgewirbelt werden und dabei in Kontakt mit sauerhaltigen Wässern kommen [152].

Die Darstellung der In-situ Techniken zur Reduzierung der vielfältigen Probleme aus dem Erzbergbau basiert u.a. auf einem Beitrag *„Geochemische In-situ Stabilisierung von Bergbaualtlasten“* [156] von *Dr. Michael Paul*, Mitglied der Unternehmensleitung der WISMUT GmbH, die für die Sanierung der Hinterlassen­schaften des ehemaligen Uranbergbaus in Sachsen und Thüringen verantwortlich war.

Der erfolgreiche Einsaz von Sanierungstechnologien bei der Aufarbeitung von Hinterlassenschaften der ehemaligen WISMUT SDAG, wie sie im Abschnitt 6.6.1.2 im Kapitel „Maßnahmen“ der HPA-Risiko-Studie [93] beschrieben wurden, lässt sich auch an den *Qualitätsverbesserungen bei den Gewäs­sersedimenten* verfolgen (Zerling et al. [157]). Ein Beispiel sind die Urangehalte in den Sedi­menten der Weiße Elster im Längsverlauf von Bad Elster flussabwärts bis Halle-Ammendorf, die zu vier Zeiten (1991, 1992, 1994 und 2000) von der *Sächsischen Akademie der Wissenschaften* unter­sucht wurden (Czegka et al. [158]). Der starke Anstieg der Daten 1991/92 für Uran in den Sedi­ment­proben­ unterhalb von Berga war auf die Einleitungen aus dem Gera-Ronneburg-Distrikt zurückzufüh­ren; der nachfolgende Rückgang der Urangehalte ist eine Folge von Verdünnungs­prozessen mit gerin­ger belas­teten Sedimenten. Nach der Inbetriebnahme einer Wasseraufbereitungs­anlage am Standort Ronneburg-Culmitzsch in 1998 gelangten keine Uran-belasteten Sickerwässer mehr in die Weiße Elster.

Es konnten aber nicht alle Kontaminationsquellen des Altbergbaus im Elbeeinzugsgebiet in gleicher Weise saniert oder gesichert werden. Hierbei spielen insbesondere geogen bedingte Einträge aus ande­ren Bergbaugebieten, in denen nicht Uran abgebaut wurde, z.B. bedingt durch radioaktive Anomalien in den Schichten des Perms im Einzugsgebiet des Mansfelder Landes (Kupferschiefer-Bergbau) eine Rolle. So variieren die Urangehalte im Kupferschiefer dieses Gebiets von 6,8 – 673 mg/kg (Hammer [159]. Nach Haack und Plimer [160] enthalten die Halden 40 – 150 mg/kg U. Daher finden sich im Mündungsbereich der Zuflüsse aus dem Einzugsgebiet des Mansfelder Landes – *Salza, Schlenze* – Urankonzentrationen, die jene aus dem Mündungsbereich der Weißen Elster überschreiten (Baborow­ski & Bozau [161], Baborowski et al. [162], Schreck et al. [163]) und teilweise beträchtlich über den Zielvorgaben zum Schutz der Oberflächengewässer liegen. Sanierungsstrategien sollten vor allem für eine der Hauptursachen der Belastung der Schlenze (und damit der Saale), die *Entwässerungsgallerie Schlüsselstollen*, entwickelt werden.

Trotz des enormen finanziellen Aufwands und der fortschrittlichen Technologien, die im *Sanierungs­pro­­jekt WISMUT* eingesetzt wurden, stellen vor allem die *Halden und Absetzbecken* ein beträchtliches Risiko für die Wasserqualität dar; dies gilt nicht nur für die Reste des jüngeren Uranbergbaus, sondern auch für die Ablagerungen aus hunderten Jahren Bergbau und Verhüttung im Erzgebirge. Insbesondere bei extremen Niederschlags- und Hochwasserereignissen findet eine *Erosion von abgelagerten Fest­stof­fen* statt, die sich weit flussabwärts ausbreiten können.

Die *Flut im August 2002* hat vor allem große Anteile der mit Arsen und Schwermetallen hochbelaste­ten Muldesedimente flussabwärts auf Überflutungsflächen – die häufig landwirtschaftlich genutzt werden – verlagert. Insgesamt weisen die Hochflutsedimente z.T. höhere Konzentrationen als die Flusssedimente auf. Konkret überschreiten ihre Gehalte in der Freiberger Mulde für Cd, Cu, Pb, Zn und für As in allen Teilsystemen die Grenzwerte der Klärschlammverordnung. Detailanalysen in den besonders stark belasteten Abschnitten Freiberg, Aue und Crossen zeigen, dass die *‘Nachlieferung’* von Arsen und verschiedenen Metallen weiterhin stattfindet und sich der Vorflutzustand wieder ein­stellt (Klemm et al. [164]). Flussabwärts tritt in den *Hochflutsedimenten* eine allmähliche Konzentra­tionsabnahme für die Problemelemente ein, so dass im Bereich der Vereinigten Mulde die Belastung geringer als in den entsprechenden Flusssedimenten der Quellflüsse ist. Auch in der Mulde zeigen die sich neu bildenden Sedimente analoge Belastungen wie vor der Flut (Broekaert et al.[165]).

1. ***Aktives Capping (Demonstrationsanlage Deuben und Pilotanlage Hitzacker/Elbe)***

In dem BMBF-geförderten *Gemeinschaftsprojekt ConSed mit australischen Institutionen* untersuchten das Institut für Wasserforschung am Forschungszentrum Karlsruhe und der Arbeitsbereich Umwelt­schutztechnik der TUHH die Möglichkeiten des *„Capping“*, d.h. der Abdeckung von kontaminierten Sedimenten, eine Technik, die seit Mitte der 1990er Jahre etwa zeitgleich in den USA, Kanada, Aus­tra­lien und Deutschland diskutiert wurde. An unserem TUHH-Institut hatte *Dr. Patrick Jacobs* seit 1997 die Auswahl geeigneter Materialien vorgenommen und eine Demonstrationsanlage vorbereitet, um mit einer Kombination von *Subaquatischer Deponie und Aktiven Capping* u.a. dioxinbelastete Sedimente aus dem *Sporthafen Hitzacker an der Elbe* vor Ort zu stabilisieren (Kasten 8a/b; [166-172]). Dem eigentlichen Bauprojekt unter der Federführung der *Fa. Josef Möbius Bau-Gesellschaft GmbH & Co., Hamburg* [82], bei dem die gewässerschonende Verfrachtung des kontaminierten Sediments in ein subaquatisches Depot und die Wirksamkeit einer aktiven Abdeckung untersucht werden sollte, gingen Pilotexperimente an einem *Test-See bei Deuben in Sachsen-Anhalt*, gemeinsam mit der Arbeitsgruppe von *Prof. Dr. Ulrich Stottmeister* (UFZ Leipzig-Halle) voraus.

Am 29.01.2002 erteilte der Landrat des Landkreises Lüchow-Dannenberg die *wasserrechtliche Plan­genehmigung* für die Einrichtung eines Testfeldes zur *subaquatischen Sedimentablagerung und Abdeckung* im Bootshaven Hitzacker. Unter den insgesamt 29 Nebenbestimmungen ist vor allem die Begrenzung der Bauzeit auf die Zeitraum Januar bis März oder August bis Oktober im Hinblick auf die naturschutzfachen Anforderungen hervorzuheben. Trotz günstiger technischer Voraussetzungen, einem großzügigen lokalen Sponsor, der renommierten Wasserbaufirma Josef Möbius als Antragsteller und der Empfehlung von *Prof. Heinrich Reincke*, dem Leiter der ARGE Elbe, entschied das Bundes­ministerium für Forschung und Technologie gegen die Durchführung des Projekts.

**Kasten 9a/b: Geländetest und Pilotanlage zur Untersuchung der Aktiven Barriere (nach [93])**

A) Geländetest zur Effizienz der Aktiven Barriere [166]

Das Konzept der zeolithbasierten Sedimentabdeckung wurde unter Feldbedingungen erprobt. Ziel war es zum einen, die Schwermetallrückhaltung übeer einen längeren Zeitraum zu untersuchen und zum anderen die Eignung eines spezialisierten Monitoring-Programms zu belegen. Zu diesem Zweck wurde ein Testmodul bestehend aus zwei Kammern in ein sog. Enclosure in einem See bei Deuben, Sachsen-Anhalt, eingebracht. Eine Kammer enthielt ein schwermetallkontaminiertes Sediment, das mit einer Sand-Zeolith-Barriere abgedeckt war. Die zweite Kammer diente als Referenz und enthielt dasselbe Sediment, jedoch ohne Abdeckung (Abb. 5). Die Entwicklung der Porenwasser­zusam­mensetzung wurde über einen Zeitraum von einem Jahr mit einem speziell dafür entwickelten Dialyse­­proben­nehmer (Abb. 5 rechts) untersucht.

Die in der Felduntersuchung über die ortsfeste und zeitaufgelöste Bestimmung an Tiefenprofilen gewonnenen Daten zur Ausbreitung der Konzentrationsfronten verschiedener Schwermetalle in einem zeolithbasierten ABS wurde über eine zweite, unabhängige Datenbasis mit geochemischen Transport­modelen mit dem Computerprogramm PHREEQC bestätigt. Die Extrapolation über die in Laborver­suchen zu realisierenden Zeiträume hinaus, die mit Hilfe dieser Modelle durchgeführt wurde, zeigte, dass auch bei nur geringen Zeolithgehalten in der Barriere unter günstigen Bedingungen Standzeiten von mehreren Jahrzehnten zu erwarten sind. Die Effizienz der Schwermetallrückhaltung kann jedoch unter ungünstigen Bedingungen vor allem durch den Einfluss konkurrierender Metall-Ionen oder kom­plexierender organischer Moleküle wie Huminstoffen, sehr weitreichend kompensiert werden.

B) Demonstrationsprojekt Hitzacker/Elbe (Möbius/TUHH [82])

Nach den ersten beiden Stufen des internationalen Gemeinschaftsprojektes zum Sediment-Capping (neben den beiden deutschen Projekten der TU Hamburg-Harburg und des Forschungszentrums Karls­ruhe waren in dem ConSed-Programm noch australische Forschergruppen beteiligt), sollte ein groß­maß­stäbliches Pilotprojekt durchgeführt werden, in dem die ausgewählten Barriere-Materialien und das spezi­elle Überwachungssystem unter Praxisbedingungen über einen Zeitraum von 2-3 Jahren getestet werden konnten. Als Teststandort wurde der Sportboothafen von Hitzacker/Elbe ausgewählt, der mit hochbelastetem Elbesediment stark verschlickt war und deswegen über lange Zeiträume nicht benutzt werden konnte. Für die Unterbringung von ca. 10.000 m³ Hafenschlick war vorgesehen, den zur Elbe führenden Zugangskanal auf ein Niveau oberhalb des mittleren Wasserstandes zu verfüllen und anschließend mit Boden zu überdecken (Abb. 6 links); der Zu­gang für die Sportboote sollte künftig über den Elbenebenfluss der Jeetzel erfolgen, der oberhalb des Hafens in die Elbe mündet. Es war beabsichtigt, das Testfeld (200 m², rechts oben in der geplanten Verfüllung) in der für einen Zeit­raum von 2 Jahren offen zu erhalten und anschließend wie das restliche Gebiet zu verfüllen.

*Aufbau des Testfelds und Überwachung des weiteren Untersuchungsgebiets*

Innerhalb des Testfeldes sollten 8 Versuchs-Enclosures installiert werden, in denen verschiedene Ab­deckungen auf dem umgelagerten Sediment aufgebracht werden. Die Enclosures bestehen aus zylin­drischen Rohrstücken aus Kunststoffe mit einem Durchmesser von 2 m. Die einfachste Form der Abdeckung ist eine homogene Mischung einer reaktiven Komponente in einer weitgehend inerten Sandmatrix. Darüber hinaus können geschichtete Abdeckungen zu einer Optimierung hin­sichtlich der Rückhaltung spezifischer Schad­stoffe oder Schadstoffgruppen erzielen. Insbesondere zur Optimierung können solche Barrieren verwendet werden, die spezielle Geotextilien als Trägere und Stützmaterial einsetzen.

**
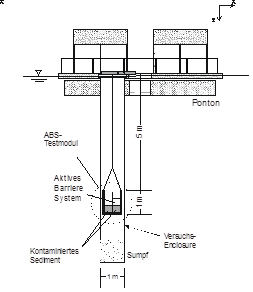
**

**Abb. 5** (links) Schnitt durch Ponton und Versuchsenclosure mit ABS-Testmodul, das in 6 m Tiefe inner­halt des Enclosures fixiert wurde. (rechts) Verwendung eines Dialyseprobenehmers zur Bepro­bung der Porenlösungen im Sediment und Abdeckung (aktives Barrieresystem). Nach Einbringung durch Taucher kann die Probenahme und Befüllung von der Gewässeroberfläche durchgeführt werden.

**Abb. 6** Skizze des verfüllten Zugangkanals zum Bootshafen Hitzacker/Elbe mit Capping-Testfeld und Überwachungs-Einrichtungen. Rechts: Schnitt durch das Testfeld. Aus: Antrag Möbius GmbH/TU Hamburg-Harburg [82]

***(c) Monitored National Attenuation (aus [93])***

Für die Anwendung des Natural Attenuation Ansatzes bei Gewässersedimenten im engeren Sinne, d.h. den gering bis mäßig kontaminierten Ablagerungen in einem Oberflächengewässer, wird als wichtig­stes Kriterium die *mechanische Stabilität gegen eine Aufwirbelung* heranzuziehen sein. Die Entschei­dung wird in erster Linie auf der Grundlage von Untersuchungen zur kritischen Scherfestigkeit und bestimmten Annahmen über die Wahrscheinlichkeit und Auswirkungen von hydrodynamischen Vor­gängen, im Allgemeinen von Hochwasserereignissen gefällt werden. Gegebenenfalls sind Überlegun­gen zu zusätzlichen *Sicherungsmaßnahmen* – Verfestigung des kontamininierten Materials oder Abdeckung – anzustellen; bei einer Abschirmung durch Abdeckung oder Verdünnung durch natürliche Sedimentation muss eine mögliche Vermischung durch sedimentlebende Organismen beachtet werden.

Es ist zu beachten, dass erst nach einer günstigen Prognose hinsichtlich der mechanischen Stabilität des Sediments auch die *chemisch-biologischen NA-Prozesse*, d.h. eine zusätzliche Risikominderung durch Abbau und Umwandlung bzw. verstärkte Feststoffbindung der Schadstoffe, ins Kalkül gezogen werden können. Diese letztgenannten Effekte sind in hydrodynamisch stärker beeinflussten Gewässer­sedimenten nur schwer nachzuweisen.

Umgekehrt sind die Verhältnisse bezüglich des NA-Ansatzes auf Auenböden. Insbesondere bei den nur gering mit kontaminierten Sedimenten beaufschlagten, episodisch überfluteten Bodenprofilen sollte die Diskussion über eine Sanierung mit Natural Attenuation Prozessen zuerst einen potenziellen *Abbau von Schadstoffen* in der ungesättigten Bodenzone in Betracht ziehen und durch entsprechende Profildaten abschätzen. Für den Nachweis von Natural Attenuation Effekten können *ökotoxikologische Methoden* – Biotest für gelöste und feste Phasen – hilfreich sein (s.u.).

Im Mittelpunkt des Maßnahmenkapitels [93] stand der *Einsatz naturnaher Methoden bei flächenhaften Kontaminationen* von Sedimenten und Auenböden:

- Bei Gewässersedimenten im engeren Sinne ist die *mechanische Stabilität gegen eine Aufwirbelung* das wichtigste Kriterium für die Anwendung naturnaher Sanierungsverfahren. Erst dann können auch *chemisch-biologische MNA-Prozesse* ins Kalkül gezogen werden, d.h. zusätzliche Risiko­minderung durch Abbau und Umwandlung bzw. verstärkte Feststoffbindung der Schadstoffe.
- Umgekehrt sind die Verhältnisse bezüglich des MNA-Ansatzes auf *Auenböden*. Insbesondere bei den nur wenig mit kontaminierten Sedimenten beaufschlagten, episodisch überfluteten Boden­profilen ist zuerst ein potenzieller Abbau von Schadstoffen in der ungesättigten Bodenzone durch entsprechende Profildaten nachzuweisen.

**Tabelle 11** Standorteigenschaften und Bedingungen, die besonders günstige Voraus­setzungen für die Anwendung von Capping und Monitored Natural Recovery darstellen (nach Anonym 2005d [116])

| **In-Situ Capping** | **Monitored Natural Recovery** |
| --- | --- |
| **Allgemeine Standorteigenschaften** | |
| - Geeignete Arten und Mengen von Materialien zur Herstellung der In-situ-Abdeckung sind leicht verfügbar - Wassertiefe ist ausreichend um die Abdeckung mit den vorgesehenen Nutzungszielen einzu­rich­ten (z.B., Schifffahrt, Hochwasserschutz) - Wahrscheinlichkeit von Beschädi­gungen an der Abdeckung, z.B. beim Anlegen von größeren Schiffen, ist gering oder ist kontrollierbar | - Die beabsichtigte Landnutzung oder neue Strukturen sind mit einer MNR-Maßnahme verträglich. - Die MNR-Prozesse werden mit hinreichender Sicherheit mit einer Geschwindig­keit ablaufen, um die Bioverfügbarkeit/Toxizität der Schad­stoffe in einem akzeptab­len Zeit­rahmen durch Festlegung bzw. biologischen und abiotischen Abbau auf das erwartete Maß zu reduzieren. |
| **Menschliche und ökologische Umwelt** | |
| - Die erwartete menschliche Exposition ist bedeutend und wird nicht durch intensive institutionelle Kontrollen überwacht - Die langfristige Verringerung der Risiken über­wiegt mögliche Nachteile bei den ökologischen Lebensbedingungen, oder diese Bedingungen werden durch die Abdeckung erst hergestellt | - Die Sedimentablagerung ist ausreichend stabil und wird nach aller Wahrscheinlichkeit auch längerfristig so stabil bleiben - Der Standort enthält empfindliche, einzig­artige Bereiche, die durch In-situ Abdeckung oder Ausbaggern irreversibel geschädigt werden könnten |
| **Hydrodynamische Bedingungen** | |
| - Die hydrodynamischen Bedingungen (z.B. Hoch­­wässer, Eisschrammen) gefährden die Abdeckung wahrscheinlich nicht oder diese Voraussetzungen können durch entsprechende konstruktive Designs geschaffen werden - Das Ausmaß des Grundwasserzuflusses in die Abdeckung ist gering und führt aller Wahr­schein­­lichkeit nach nicht zu unzulässigen Schadstoff­freisetzungen | - Die Sedimentbildung erfolgt in den Kontami­na­tions­gebieten - Die hydrodynamischen Bedingungen (z.B. Hoch­wässer, Eisschrammen) werden wahr­scheinlich den Ablauf der MNR-Prozesse nicht beeinträch­tigen |
| **Sediment-Charakteristiken** | |
| - Das Sediment besitzt eine ausreichende Stabi­lität um die Abdeckung zu tragen (z.B. höhere Dichte, niedriger Wassergehalt, spez. Einbau­) | - Das Sediment ist hinreichend erosionssicher (z.B. „kohesiv“ oder verfestigt) |
| **Schadstoff-Charakteristiken** | |
| - Die Schadstoffe bewegen sich nur sehr lang­sam durch die Abdeckung - Die Schadstoffe bedecken auch benachbarte Gebiete (z.B. als Vereinfachung des Capping-Vorganges) | - Die Schadstoffe zeigen bereits einen raschen biologischen Abbau oder eine Umwandlung zu weniger toxischen Formen - Die Schadstoffkonzentrationen sind niedrig und über das Gebiet diffus verbreitet - Die Schadstoffe besitzen eine geringe Bioakku­mulationstendenz |

Übersichten über internationale Beispiele geben Magar & Wenning [173] und Evison et al. [174] u.a. zu folgenden Aspekten (Abschnitt 6.8.1.4): (1) *Kontrolle der Schadstoffquelle*, (2) Nachweis von *natürlichen Sedimentabdeckungen*, (3) *wirksame In-situ Prozesse*, (4) *Enhanced Monitored Natural Recovery* [eMNR] und (5) *Monitoring von In-situ-Prozessen*.

Im Rahmen des *BMBF-Verbundes KORA* („kontrollierter natürlicher Rückhalt und Abbau von Schad­stoffen bei der Sanierung kontaminierter Böden und Grundwässer“, 2003-2007) wurden speziell für die Zielmedien „Gewässersedimente“ und „Auenböden“ *Handlungsempfehlungen* erarbeitet [28, 29]. Eine vorläufige Version für den Aspekt *„MNA in Sedimenten“* ist im Abschnitt 6.8.2 wiedergegeben [93]: Prüfkriterien für MNA (6.8.2.1), die Abgrenzung/Definition von Grundfällen/Szenarien (6.8.2.2) und Bewertungskonzepte für MNA in Sedimenten (6.8.2.3) werden dort nach der EPA-Handlungs­anweisung (Anonym 2005d [116]) dargestellt. Das *DVWK-Kompendium* „Methoden zur Erkundung, Untersuchung und Bewertung von Sedimentablagerungen und Schwebstoffen in Gewässern“ [175] ist eine Grundlage für die Planung von Sanierungsmaßnahmen für kontaminierte Sedimente.

***Beispiel der Jeßnitz/Spittelwasser Überflutungszone*** *[176, 177]:* ***Schlussfolgerung und Ausblick***

Das natürliche Selbstregenerierungspotenzial von Böden und Sedimenten im Spittelwassergebiet reicht nicht aus, um mittelfristig eine landwirtschaftliche Nutzung oder Trinkwasser­gewinnung zuzulassen. Vor allem wegen der extrem hohen Gehalte an PCDD/F (> 10.000 ng/kg I-TEQ) in den oberen Boden­bereichen würde bei einer Förderung der Dispersion lediglich zu einer weiteren Ausbreitung der Kontaminationen flussabwärts führen und so, bei Werten über 5 ng/kg I-TEQ), eine uneingeschränkte landwirtschaftliche Nutzung verbieten. Außer einer Ausgrabung und sicheren Lagerung des ­hoch­kontaminierten Materials, geringer oder ­nicht-invasive Verfahren wie pH-Kontrollen oder der Einsatz von Capping-Techniken sind in der Spittelwasserüberflutungszone besonders erfolgsversprechend.


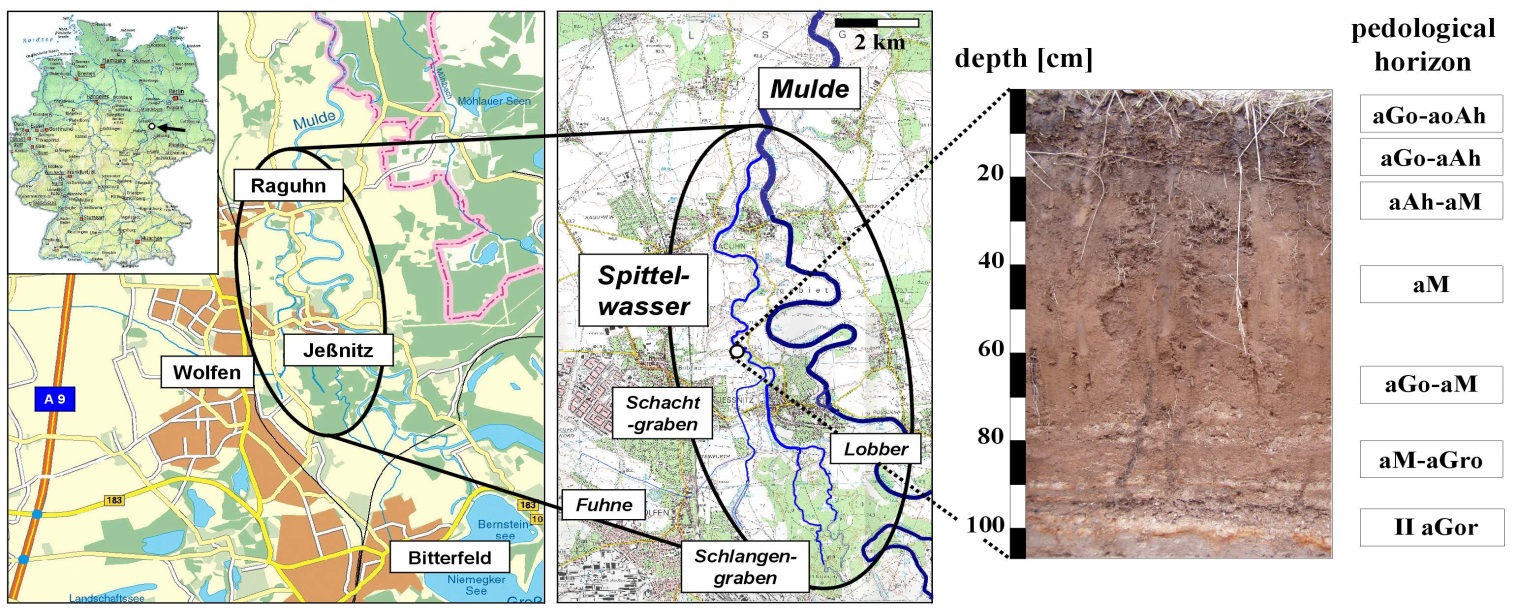


**Fig. 7**: Lage des Untersuchungsgebiets mit dem Fluviosol-Profil 'Jeßnitz' [177]

***(d) Activated Carbon Amendment Technologies ( [110] und aktuelle Literatur 2015)***

Die grundsätzliche Eignung von „Black Carbon“, Kohle und Kerogen in Sedimenten und Böden für die extensive Sorption von organischen Verbindungen wurde von *Gerard Cornelissen*, Norwegian Geotechnical Institute, und Kollegen 2005 untersucht [178]. Im Februar 2011 erschien ein Übersichts­beitrag von *Upal Ghosh* und Kollegen [179] über den Einsatz dieser Zuschlagstoffe als neue Richtung bei der Sanierung von Sedimenten. In der Tabelle 12 werden die Schritte zur ökologischen Bewertung dieses Ansatzes dar­gestellt

Tabelle 12: Beispiele aus der Zeitschrift Environmental Science & Technology (2011-2013) über Unter­suchungen zur Charakterisierung positiver und negativer Effekte bei der Stabi­lisierung von kontaminierten Gewässer­sedimenten mit Aktiv­kohle (aus Förstner U (2013) Vom Wasser [110])

|  | Titel (übersetzt) und Autoren |
| --- | --- |
| 1 | *Ökotoxikologische Effekte bei Makroinvertebraten – unbelas­tete und belastete Proben* (Kupryianchyk et al. 2011) |
| 2 | *Sorption von organischen Chemikalien an frische/freiland- gealterte Aktivkohle in Sedimenten* (Oen et al. 2012) |
| 3 | *Modellierung des Zielkonflikts zwischen PAH-Toxizitäts­redu­k­­tion und negativen Effekten* (Kupryianchyk et al. 2012) |
| 4 | *Bioturbation und gelöste organische Substanzen verstärken die Schadstoffmobilisierung* (Kupryianchyk et al. 2013) |
| 5 | *Biologische Wirkungen durch Zusätze von Aktivkohle bei der Sedimentsanierung* (Review, Janssen/Beckingham 2013) |

1 – Universität Wageningen/IMARES; 2 – Norwegian Geotechnical Institute, University of Baltimore, Stanford University, Norwegian Institute for Agricultural and Environmental Research, Lindum Ressurs og Gjenvinning (Drammen), Universität Stockholm, University of Life Sciences (UMB, As/Norwegen); 3 und 4 – Universität Wageningen/IMARES; 5 – ETH Zürich, Universität Tübingen

Bereits vor diesen Untersuchungen analysierten van Noort & Koelmans [180] den Aspekt des Nicht­gleichgewichts von organischen Verbindungen in Sediment-Wasser-Systemen als besonders kritischen Faktor beim praktischen Einsatz dieser Methode. Die Autoren [180] verweisen darauf, dass die *Tech­ni­schen Leitlinien der Allgemeinen Umsetzungsstrategie für die EU-Wasserrahmenrichtlinie (Docu­ment No. 27* [181]) bislang keine quantitativen Angaben ermöglichen, ab wann Sedimente eine aus­reichend geringe Schadstoffquelle für Oberflächengewässer darstellen.

Eine Übersicht von Patmont et al. [182] fasst *25 Sedimentsanierungsprojekte* zusammen und zeigt die wesentlichen Fortschritte auf dem Gebiet der Aktivkohleanwendung. Mit den beschriebenen Methoden lassen sich Kohlenwasserstoffverbindungen unter verschiedenen Bedingungen immobilisieren, wobei besonders eine erhöhte Wirksamkeit aufgrund des fortschreitenden Massentransfers auffällt. Einen Entscheidungrahmen für die Bewirtschaftung kontaminierter Sedimente und zur Rolle der Aktivkohle­technologien haben kürzlich Kupryanschyk et al. [183] gegeben (Fig 8).

Abbildung 8: Entscheidungsrahmen für die Bewirtschaftung kontaminierter Sedimente [183]

Die Hauptnachteil der beiden traditionellen Strategien, *Baggern und Capping*, sind ihre beträchtlichen Auswirkungen auf die Umwelt und die Notwendigkeit von hohen Investitionen (besonders beim Bag­gern mit nachfolgender Sedimentbehandlung und –lagerung). Außerdem kann es Jahre oder Jahrzehnte dauern bis eine ausreichende Qualitätsverbesserung erreicht ist [183]. Wie bei USEPA [116, 184] und ITRC [185] diskutiert, ist es an vielen Standorten günstig *Reinigungstechnologien für spezifische Zonen* einzusetzen; man kann so langfristig einen ausreichenden Schutz erzielen, kurzfristig negative Effekte minimieren und ingesamt eine bessere Kosten-/Nutzenrechnung erhalten.

Die chemischen Vorgänge wiederum bestimmen die Reaktivität und Bioverfügkeit von Schadstoffen im Sediment. Die Messung der spezifischen Bindung von Stoffen an Sedimentkomponenten, bspw. amorphen Kohlenstoff oder von spezifischen Formen an reduziertem Kohlenstoff, und der beteiligten Verteilungskoeffizienten sind schwierig. Deshalb bilden die Entwicklung von *passiven Beprobungs-Methoden* einen besseren Zugang zu den Zieldaten wie Reaktivität, Bioakkumulation und Toxizität. PSMs verbessern risikobasierte Entscheidungen, weil die Bioverfügbarkeit von Schadstoffen in den Sedimenten direkt quantifiziert werden kann. Künftige Wünsche umfassen einen standardisierten Ansatz für PSM-Messungen, die Korrektur von Nichtgleichgewichtsbedingungen, einen Leitfaden für die Auswahl and Umsetzung von PSMs und die Übersetzung von gesammenlten PSM-Daten [195]^^[[13]](#footnote-13)^^.

**9. Prioritäre organische Schadstoffe beim Übergang vom Binnenland in die Nordsee**

Die großen Flusshäfen wie Rotterdam und Hamburg besitzen vor allem im Hin­blick auf die Sediment­qualität eine Scharnierfunktion im Einzugsgebiet-Küsten-Kontinuum (Salomons [198]). Es besteht deshalb ein umweltpolitischer Zusam­men­hang zwischen den Richtwerten, die z.B. für die Ent­schei­dung ‚Landdeponie vs. Verklappung’ von Baggergut herangezogen werden, und den Zielvorgaben für Immissionswerte, mit denen längerfristig die Schadstoffeinträge aus dem Oberlauf reduziert werden sollen. Die Politik des Hafens von Rotterdam wurde früh darauf ausgerichtet, dass die ‚CTT’-Werte [199] von kritischen Stoffen für eine Verklappung von Baggergut in der Nord­see auch die Messlatte für die Maßnahmen im Einzugsgebiet gemäß Artikel 16 WRRL sein sollten (s.u.).

Für die Risikobewertung sind theoretisch folgende Parameter zu berücksichtigen (Babut et al. 2006 [200]): (a) Lage der kontaminierten Fläche/der Emission in Bezug auf das Einzugsgebiet; (b) Ausmaß der Kontamination; (c) Stabilität des Sedimentes und (d) Volumen des kontaminierten Materials. Das *Risiko* ergibt sich aus der Verknüpfung dieser Faktoren. Kein einzelner Para­meter ist allein risiko­bestim­mend. Eine hohe Kontamination, die nur ein kleines Sedimentvolumen betrifft, stellt eine geringere Gefahr dar als eine weit­läufige hohe Kontamination. Wenn jedoch letztere in einem sehr erosions­stabi­len Gebiet liegt, während das kleinere Volumen beständig resuspendiert wird, ergibt sich eine andere Risikoverteilung. *Frachtbetrachtungen* können diese unterschiedlichen Parameter bis zu einem gewissen Grad integrieren. Auch Korrelationen zwischen Konzentrations­erhöhungen der Kon­taminationen suspendierten Materials und Flutereignissen geben Hinweise auf mögliche Resuspens­ionen. Ebenso können Vergleiche von experimentell gemessenen kritischen Schubspannungsgeschwin­digkeiten und natürlich auftretenden Sohlschubspannungen hilfreich sein, um die Erodier­bar­keit eines Gebietes abschätzen zu können [201].

**Tabelle 12:** Ableitung von Risiken durch Hexachlorbenzol in Sedimenten aus den Staustufen des Hochrheins und des Oberrheins für den Hafen Rotterdam auf der Basis von Einzelindikationen (HQ_X_ Häufigkeit des Abflussereignisses in X Jahren; BAU – normale Abflussbedingungen [Business as usual]; +/- kein offensichtl. Effekt, + kleiner Effekt, ++ signifikanter Effekt, +++ großer Effekt) [202]

|  | Indikation von Resuspendierungen | | | | | | Risiko  Hafen Rotterdam |
| --- | --- | --- | --- | --- | --- | --- | --- |
|  | Abfluss | | Erosions-potenzial | | Fracht-zunahme | |  |
| **Gebiet** / *Substanz* |  |  |  |  |  |  |  |
| **Staustufen des Hochrheins und des Oberrheins** | | | | | | | |
| *Hexachlorbenzol HCB* | BAU | +/- | | + | | vorhanden | |
|  | >HQ_1_ | + | | +++ | | hoch | |
|  | >HQ_10_ | ++ | |  | | hoch | |
|  | >HQ_50_ | +++ | |  | | hoch | |

Am Beispiel des Rheins konnten für die Betrachtung des Risikos die in Frage kommenden, zahlreichen „Areas of Concern“ unter Berücksichtigung der Informationen zu transportierten Frachten und Kon­zen­trationen sowie zur Erodierbarkeit der Sedimente auf wenige „Areas of Risk“ eingegrenzt werden ([202], Tabelle 12). Ein solches war das Oberrheingebiet bereits bei Fluten mit jährlicher Wiederkehr­wahrscheinlichkeit. Sedimente in diesem Gebiet mit der höchsten Priorität wiesen eine hohe HCB-Belastung auf, die auf direkte industrielle HCB-Einleitungen bis in die 80er Jahre hinein durch die Firma Dynamit Nobel zurückzuführen ist [203].

Die Sedimentqualitätskriterien, die zur Risikobewertung der Rheinsedimente herangezogen wurden, basierten auf dem niederländischen CTT-System (‚Che­mistry Toxicity Test’). Dieses gibt die Grenz­werte für Schadstoffe in Hafen­sedi­menten Rotterdams an, bei deren Überschreitung eine Verbringung von Baggergut in die Nordsee nicht mehr erlaubt und stattdessen eine teure Deponierung durch­zuführen ist. Ein Hinweis auf das Potenzial einer „Area of Concern“ zur Überschreitung des lokalen Schwellenwertes im Hafen von Rotterdam, falls kontaminierte Sedimente resuspendiert und fluss­abwärts verfrachtet werden, lässt sich daraus ableiten, dass man die Verdünnung durch die Schweb­stoffe, die vor allem aus den Zuflüssen stammen, in Rechnung stellt [204, 205]: X^A^ = X^*^ SM^KB^/SM^A^ , wobei X^A^ die Konzentration des Stoffes an der Lokalität A ist, bei welcher der entsprechende CTT-Wert bei Rotterdam überschritten würde; X ist der CTT-Wert der betreffenden Substanz; SM^KB^ ist die Schwebstoffracht bei Kleve-Bimmen (nahe der deutsch-holländischen Grenze); SM^A^ ist die Schweb­stofffracht an der Lokalität A.

Das Ergebnis unserer Studie für den Port of Rotterdam [202] fiel zeitlich zusammen mit den Plänen der Wasserschifffahrtsverwaltung des Landes Baden-Württemberg, ca. 300.000 m³ des HCB-kontami­nierten Materials, das sich in der nördlichsten Staustufe Iffezheim abgelagert hatte, über den Damm in den Rhein zu verspülen. Dies wurde als einzige Möglichkeit gesehen, die Sicherheit des Dammes zu gewährleisten, nachdem die Kapazität bisher genutzter Lagerstätten ausgeschöpft war. Die Ergebnisse der Rheinstudie bestätigten jedoch die Befürchtungen der Hafenbehörde Rotterdam, diese Verspülun­gen würden zu einer Verschlechterung der Sedimentqualität im Hafengebiet und damit zu finanziellen Mehraufwendungen bei Entsorgung des Baggerguts führen [206].

In der Presse wurde das Bild eines Skandals aus den späten 1980ern mit Hexachlorbenzol aus Rhein­felden wiederbelebt, einer „Chemie-Altlast, die vom Hochrhein bis zur Nordsee wandert“ [207]; wegen der geplanten Umlagerung von Sedimenten aus der Staustufe Iffezheim und der Verletzung des Loyalitätsprinzips in der Gemeinschaft durch Deutschland wollten die Niederlande gegen Baden-Württemberg vor der Europäischen Kommission klagen (Anonym 2005 [208]). Bei späteren Bagger­arbeiten wurden die Sedimente per Schiff in die Niederlande verbracht [209].

**Box 10: Sedimentmanagementplan Rhein – Internationale Kommission zum Schutz des Rheins**

Der zugrunde liegende konzeptionelle Ansatz der IKSR, deren Zusammenfassung 2009 veröffentlicht wurde [210] baut auf den Empfehlungen des Europäischen Sediment­netzwerkes SedNet und zweier Studien zur Sedimentbelastung des Rheins [212] und der Elbe [42] auf. Der mehrstufige Prozess umfasst folgende Schritte (nach Heise in [202]):

- Zunächst werden die für das Einzugsgebiet relevanten *Schadstoffe* ermittelt und die durch diese Schadstoffe kontaminierten Gebiete identifiziert.
- Im zweiten Schritt wird ermittelt, welche *Mengen kontaminierte Sedimente* in dem untersuchten Bereich vorliegen.
- In dem dritten Schritt wird untersucht, inwiefern durch eine *Remobilisierung* kontaminierter Sedi­mente der gute Gewässerzustand für stromabwärts gelegene Gebiete beeinträchtigt werden kann bzw. beeinträchtigt wird. Hierbei spielt die Bewertung des Remobilisierungsrisikos durch Hochwasser, Windeinwirkung und durch anthropogene Eingriffe (Baggerung, Umlagerung, Schiffsverkehr) eine wichtige Rolle.

Der einzige Schadstoff, der für die schlechte Einstufung der Oberrheinsedimente verantwortlich ist, ist *Hexachlorbenzol*. HCB gehört zur Liste prioritärer gefährlicher Stoffe, für die nach WRRL ein sog. „phasing out“ angestrebt wird. Eine Sanierung wird nach heutigem Wissensstand für Sedimentations­bereiche in den Stauhaltungen Marckolsheim und Rhinau, wo in Teilbereichen hoch belastete und leicht remobilisierbare Sedimente lagern, vorgeschlagen. Die Sanierung bei Marckolsheim und Rhinau ist notwendig, damit der andauernde Eintrag in die unterhalb liegenden Stauhaltungen unterbrochen wird. HCB gelangt über den Schwebstofftransport in der Größenordnung von (im Mittel) 10-20 kg pro Jahr bis in die Nordsee. Durch die Sanierung der beiden o.g. Stauhaltungen können dagegen nach vorläufigen Schätzungen mehrere 100 kg HCB entfernt werden. Eine Minderung des Problems für die Unterlieger wird daraus langfristig zu erreichen sein. Nach Sanierung der Quellen der Belastung ist mit einem weiteren deutlichen Rückgang der HCB-Belastung in den Sedimenten der unterhalb gelegenen Stau­haltungen zu rechnen. Der Erfolg der Sanierung soll über ein begleitendes Monitoringprogramm dokumentiert werden.

Um die Kosten der Sanierung der hochbelasteten Sedimentbereiche der beiden Stauhaltungen Marckolsheim und Rhinau möglichst niedrig zu halten muss eine relativ aufwändige *Voruntersuchung* mit professionellem Bohrgerät (Rasteruntersuchung) und Kernprobenahmen erfolgen, um die hoch belasteten Sedimentschichten (-horizonte) einzugrenzen. Nach vorläufigen Schätzungen muss man in der Stauhaltung von Marckolsheim von 160 000 bis maximal 260 000 m³ belastetem Sediment aus­gehen. Eine Möglichkeit der Sanierung besteht in einer sicheren *subaquatischen Unterbringung* des Materials im Küstenbereich (z. B. Deponie „IJsseloog“). Die Unterbringung des Baggermaterials einschließlich Transportkosten wird dabei zwischen 20 und 32 € Netto pro m³ Baggergut kalkuliert.

Eine andere Alternative ist eine teilweise *subaquatische Sicherung* des remobilisierbaren Sedimentes unter der Bedingung, dass der ordnungsgemäße Wasserabfluss aufrecht erhalten werden kann. Auch eine Lagerung des Baggergutes in unmittelbarer Nähe (Inseln, Molen) analog zur Staustufe Iffezheim sollte geprüft werden. Die Kosten sind überschlägig mit rund 8 € Netto pro m³ zu veranschlagen. Weiter sollten Möglichkeiten der Abdeckung (*Capping*) der belasteten Sedimente untersucht werden und geprüft werden, ob im Zusammenhang mit aus anderen Gründen notwendigen Baggerungen eine Deponierung möglich ist.

Zur *Verminderung bzw. Vermeidung der Sedimentationen* in den Staustufen und für einen aus­geglichenen Sedimenthaushalt wird eine Verknüpfung von strombaulichen Maßnahmen (z. B. Bau von Trennmolen oder Leitwände) und operativen Maßnahmen (Optimierung der Steuerung von Wehranlagen, der Umlagerungstechnik sowie der Baggerstrategie) vorgeschlagen. Voraussetzung ist allerdings, dass vorher die notwendigen Sanierungsmaßnahmen durchgeführt wurden, um zu vermeiden, dass belastete Sedimente noch schneller als bisher flussabwärts transportiert werden.

**Meeresschutz**

Es wird insgesamt immer deutlicher, dass die bisherigen Anstrengungen zur Schadstoffreduzierung nicht ausreichend sind und die Erreichung des „guten“ ökologischen sowie chemischen Zustands der Gewässer gemäß WRRL sowie auch des „guten“ Umweltzustands gemäß Meeresstrategie-Rahmen­richtlinie (MSRL [211]) gefährdet ist: „Der Schadstofftransfer aus dem gesamten Elbegebiet führt zu erheblichen Risiken für die Meeresumwelt und zu gravierenden Einschränkungen im Umgang mit Sedimenten, insbesondere im Tidebereich. Im Ergebnis lassen sich die wesentlichen elberelevanten anorganischen und organischen Schadstoff-Hauptherkunftsbereich verorten“.

Dass in den kommenden Jahren sowohl über die Erweiterung der prioritären Stoffe als auch durch die Meeresstrategie-Richtlinie eine signifikante Aufwertung der Rolle von Dioxinen/Furanen in diesem kritischen Umweltbereich eintreten wird, scheint – auf den ersten Blick – die Akteure im wichtigsten Praxisbereich von Gewässersedimenten wenig zu kümmern: die „Gemeinsamen Übergangs­bestim­mun­gen zum Umgang mit Baggergut in den Küstengewässern“ (GÜBAK 2009, aktualisiert Oktober 2013 [212]) erwähnen „Dioxin“ weder auf der (langen) Liste von Richtwerten noch auf den Melde­formularen. Bei näherem Hinsehen liegt der Vorteil dieser Einschränkung bei der Hafenverwaltung von Hamburg, die in den kommenden Jahren u.a. als Folge der Fahrrinnenvertiefung in der Elbe zusätzlich 1 Mio. m³/a Feinmaterial aus der Tideelbe umlagern möchte:

*„Bei der Beurteilung der Umlagerung von Baggergut ist eine Abwägung vorzunehmen zwischen den Notwendigkeiten des quantitativen Sedimentmanagements, ökologisch verträglichen Grenzen des Um­lagerns und der erforderlichen Sanierung im Elbegebiet“[213].*

Die aufwändige Antragstellung wird es nicht leicht haben, den Plan gegen das gewachsene Misstrauen im Lager des Meeresschutzes durchzusetzen. Dioxin ist die elbetypische Substanz, aber ausgerechnet am Anfang und am Ende der Ausbreitungskette dieser nunmehr auch offiziell als „prioritäre Stoffe im Bereich der Wasserpolitik“ eingestuften Substanzgruppe wird so getan, als gäbe es damit kein Problem mehr (siehe auch Gutachten von Murray & Breedveld [214] für WSV/Hamburg Port Authority).

Bei der Vorgehensweise der Hamburger Hafenverwaltung liegt das Problem darin, dass die Kriterien „ökologisch vertretbare Grenzen“ und „Sanierung im Elbegebiet“ nach den Erfahrungen mit den Elbe-Oberliegern zeitlich extrem lange auseinander liegen können. Zwar betreibt Hamburg eine beträcht­liche Förderung von „Maßnahmen“ vor allem im Oberlauf der Elbe [215, 216], aber als mittelfristiger Beobachter wird man den Eindruck nicht los, dass der natürliche Vorschub der Schadstofffronten und die bei jeder Fahrrinnenvertiefung bzw. neuen Umlagerungsstrategie in Kauf genommene Konzentra­tionserhöhung bei kritischen Substanzen schneller voranschreiten als alle freiwilligen Aktionen gegen Schadstoffe oberhalb des hamburgischen Staatsgebiets.

**10. Historisch kontaminierte Sedimente im Elberaum – Bewertung des ersten WRRL-Zyklus**

Das Thema „Sedimentaltlasten“ und speziell die Stoffgruppe der Dioxine und Furane waren schon im Vorfeld der EU-Wasserrahmenrichtline, dann während der mehrstufigen Bearbeitung in den Behörden der Elbeanliegerstaaten und schließlich bei der Bewertung des 1. WRRL-Zyklus durch die FGG Elbe (*„nur ein ganzheitliches Sedimentmanagement im gesamten Einzugsgebiet der Elbe und mit effektiven quellenbezogenenMaßnahmen ist Erfolg versprechend….“*) eine besondere Herausforderung an die Kommunikationsfähigkeit der Akteure. Bereits die Stellungnahmen zur Studie *„Bewertung der Risiken durch feststoffgebundene Schadstoffe im Elbeeinzugsgebiet“* [43] enthielten entweder kaum erfüll­bare Erwartungen oder wünschten nur begrenzte Informationen bei ihren eigenen Schwerpunktthemen:

- Der Auftraggeber Hamburg Port Authority wollte im ersten Anlauf eine *Nicht*aufnahme der Kapitel „Dioxin“ und „Maßnahmen“ durchsetzen (*„gemäß Angebot sind Empfehlungen zur Art der zu ergrei­fen­­den Maßnahmen nicht Ziel dieser Studie …“*);
- die Landesanstalt für Altlasten­freistellung von Sachsen-Anhalt wollte die Studie grundsätzlich nicht akzeptieren – mit Ausnahme des Kapitels „Maßnahmen“ (*„insgesamt sehr gut geeignet, um Defizite aufzuzeigen, abzuarbeiten und Konsequenzen abzuleiten“*) und
- die Flussgebietsgemeinschaft Elbe bat die Autoren, das Logo der FGG Elbe vom Deckblatt des Papiers zu entfernen und die ‚FGG Elbe‘, bislang im Vorwort mit aufgeführt, herauszunehmen. Hauptkritikpunkt der AG Oberflächengewässer in der Flussgebietsgemeinschaft Elbe war, dass *„die Fülle des Materials in der Studie nicht in klare, erfüllbare Aussagen umgesetzt werden konnte“*.

Was ist wirklich neu bzw. schwierig für langgediente Umweltverwaltungen in ihrem Verhältnis zur Fachwissenschaft? Während die allgemeinen Vorbehalte abschließend angesprochen werden, ist zu­mindest bei der Frage nach dem Neuigkeitswert des WRRL-Ansatzes schon jetzt klar, dass die *ad hoc Gruppe „Schadstoffe“* der Flussgebietsgemeinschaft Elbe ihre Aufgaben vorbildlich gelöst hat:

- Mit dem *Hintergrundpapier* vom 2. April 2009, in dem die elbespezifischen Schadstoffprobleme zusammengefasst und, darauf aufbauend, eine Ableitung überregionaler Bewirtschaftungsziele für die Oberflächengewässer im deutschen Teil der Flussgebietseinheit Elbe für den Belastungs­schwer­punkt Schadstoffe entwickelt wurden.
- In dem *Sedimentmanagementkonzept* vom 25.11.2013 der FGG Elbe bzw. der Intern. Kommission zum Schutz der Elbe wurden vorab die Kriterien zur Auswahl und Priorisierung von Maßnahmen festgelegt. *Kriterium 7 lautet „das Ausschlusskriterium ‚Fehlen verhältnismäßiger Lösungsmöglich­keiten‘ wird nur im Ausnahmefall bei sehr gut gesichertem/begründeten Kenntnisstand angewandt“;* fraglich ist, ob damit auch die inflationäre Nutzung von Ausnahmeregelungen begrenzt wird.

Auch wenn der Blick z.Z. in erster Linie auf diese administrativen Neuigkeiten und Schwierigkeiten fällt, bleibt festzuhalten, dass die Praxis der kontaminierten Sedimente von Beginn an mehr oder weniger wissenschaftsgetrieben war. Schon in den 1970er Jahren wurden Hot Spots von Schadstoffen mit Schwebstoff- und Sedimentenproben aufgespürt und anhand von Sedimentprofilen konnte die zeitliche Entwicklung von Schadstoffeinträgen verfolgt werden. Methodenentwicklungen in allen Bereichen der Analytik, Fortschritte bei der Probennahme, statistische Auswerteverfahren, Daten­sicherung etc., bis hin zu Sanierungsverfahren gab und gibt es weiterhin auch ohne Anbindung an eine übergeordnete Richtlinien-Struktur.

Bei einer Recherche zum aktuellen Stand des Wissens beim Thema „kontaminierte Sedimente im Ein­zugsgebiet der Elbe“ die hier am Ende des ersten Arbeitszyklus der Flussgebietsgemeinschaft Elbe vor­genommen wird, muss man zunächst die Erkenntnisfortschritte in wichtigen Teilaspekten vor der Umsetzung des WRRL-Konzepts Revue passieren zu lassen. Bei den nachstehenden Beispielen, die hier chronologisch angeordnet sind, wird man feststellen, dass auch unterhalb des Schirms einer Wasser- oder Feststoffrichtlinie die fachliche Qualitätskontrolle für einen Plan, ein Verfahren oder eine Maßnahme einen immer wieder optimierten Stand des Wissens hervorbringt.

1. ***Frühe Entwicklungen in der Wasserforschung und im Gewässerschutz***

Bereits in der frühen Phase der Kommission für Wasserforschung, die durch den Senat der Deutschen Forschungsgemeinschaft 1957 eingerichtet worden war, entstanden in den Eckbereichen „Sedimenttransport“ und „Sedimentqualität“ erste Ansätze für das zentrale Thema „Historische kontaminierte Sedimente in Flusseinzugsgebieten“ (de Haar [217]). Mit dem Schwerpunktprogramm „Schadstoffe im Wasser“ (1970-1977 [218]) setzte eine systematische Erforschung kontaminierter Sedimente und Schwebstoffe ein, parallel zu den Aktivitäten der Bundesanstalt für Gewässerkunde [219]. Die Ergebnisse dieser Arbeiten wurden zur Grundlage für die Überwachungsprogramme der westdeutschen Bundesländer.

In den 1980er-Jahren wurde die Thematik „Kontaminierte Sedimente“ von den wissenschaftlich-technischen Gesellschaften aufgegriffen. Die sedimenthydraulischen Aspekte wurden vom Deutschen Verband für Wasserwirtschaft und Kulturbau ([220-224] jetzt Deutsche Vereinigung für Wasserwirtschaft, Abwasser und Abfall, DWA) vertreten; der Arbeitskreis „Sedimenttransport in Fließgewässern“ erstellte unter anderem ein Methodenhandbuch zur Erkundung, Untersuchung und Bewertung von Sedimentablagerungen und Schwebstoffen in Gewässern [225]. Die Kompetenz der Wasserchemischen Gesellschaft in der GDCh lag im Bereich der Gewässergüte; ein Arbeitskreis im Hauptausschuss III befasste sich mit Qualitätskriterien für Sedimente [226] und veröffentlichte ein Kompendium über ökotoxikologische und chemische Testmethoden [227].

***(ii) Frühe Erfahrungen im investigativen Bereich (Beispiel: Neckar und Rhein)***

Der wichtigste Einsatz von Sedimentanalysen in dieser frühen Phase der Sedimentforschung galt dem Aufspüren von Umweltvergehen, d.h. der Einleitung von persistenten Schadstoffen, die in der Wasser­phase schwer nachzuweisen waren und nur kurzzeitig auftraten. Anfang der 1970er führte das Institut für Sedimentforschung eine umfangreiche Bestandsaufnahme von Schwermetallen in den Ablagerun­gen von Elbe, Rhein, Donau, Weser, Main, Ems und ihren Nebenflüssen durch [228] und Der Spiegel [229] spitzte die Ergebnisse wie folgt zu: *„Schürfen im Schlamm der deutsche Ströme könne schon jetzt wirtschaftlicher sein als die Ausbeutung metallarmer Lagerstätten“*. Zuerst konzentrierte sich jedoch das Interesse auf die Einleitungen aus einer Pigmentfabrik an der Enz in Baden-Württenberg, die nachweislich für extreme Kontrationen von Cadmium in den Sedimenten der Stauhaltung von Lauffen und den flussabwärts folgenden Neckarwehren verantwortlich war; diese Emissionen konnten sofort durch abwassertechnische Maßnahmen unterbunden werden; die.Ablagerungen in den Stauhal­tun­gen stellen aber ein zentrales Langzeitproblem dar.

Die Erforschung der mechanischen Sedimenteigenschaften wurde zu einem Schwerpunkt am Institut für Wasserbau (IWS) der Universität Stuttgart [230]. Für den Bereich des mittleren Neckars und die spezielle Cadmiumproblematik der Stauhaltungen untersuchte das IWS das Hochwasserereignis von 1994 [231] und danach setzt sich die unterstromige Schwebstoffgesamtfracht wie folgt zusammen: ca. 88 % (200.000 t) von Oberstrom eingetragene Schwebstoffe, ~10 % (23.000 t) erodierte gering belas­tete Jungsedimente und ca. 2 % (5.000 t) erodierte hoch belastete Altsedimente; letztere sind für 38 % der gesamten unterstromigen Cadmiumfracht verantwortlich. In der Folge wurde das Untersuchungs­raster verbessert und die Analysen auf Organismen erweitert, damit konnte auch die weitere Ausbrei­tung von Cadmium im Rheineinzusgebiet verfolgt werden. An der Bundesanstalt für Gewässerkunde gab es Befunde, dass Cadmium im Herbst und Winter mit den Sedimenten aus dem staugeregelten Neckar verstärkt ausgetragen und rheinabwärts verfrachtet wird [232]. Auch die Toxizität der Schweb­stoffe und Sedimente des Neckars zeigt bemerkenswerte jahreszeitliche Schwankungen [233].

***(iii) Ausrichtung von Verbundforschungsvorhaben 1985 bis 1995***

Ausgehend von den frühen Befunden aus Bestandsaufnahmen von Schadstoffen in Sedimenten und Biota setzte Mitte der 1980er die Förderung von sedimentorientierten Verbundforschungsprojekten durch den BMBF und die DFG ein. Mit den Wechselwirkungen zwischen Schadstoffen und Partikeln in Gewässern befasste sich das BMBF-Programm „Umweltverhalten von Sedimenten“ (1991-94); die Ergebnisse wurden in einem Statusseminar *„Sediments and Toxic Substances“* anläßlich des SETAC-Kongresses in Potsdam 1993 präsentiert und zusammen mit zwei Übersichtsarbeiten „Sorption Pro­per­ties of Biofilms“ und „Bioassays on Sediment Toxicity“ als Buch veröffentlicht [234].

Eine bewußte Zusammenführung der sedimentorientierten Fachgebiete Hydromechanik, Biologie und Geochemie erfolgte auf regionaler Ebene in dem DFG-Sonderforschungsbereich "Wechselwirkungen zwischen abiotischen und biotischen Prozessen in der Tideelbe" (1986-1996). Von den 57 Beiträgen des internationalen Symposiums *„Suspended Particulate Matter in Rivers and Estuaries“* in Reinbek/­Hamburg 1994 [235] stellten 19 Beiträge den Stand der Forschung zu sediment- und schadstoffbezo­ge­nen Fragestellungen in Deutschland dar.

Deutlich über die Bestandsaufnahmen hinaus ging das niederländische Forschungsprogamm über Böden und Sedimente als *„Chemische Zeitbomben“* (1991-1994). Das sowohl in den Niederlanden als auch in Deutschland umstrittene Projekt basierte auf den Arbeiten von William Stigliani [236, 237] am International Institute for Applied System Analysis (IIASA), Laxenburg/Österreich^[[14]](#footnote-14)^.

Stigliani konnte erstmals zeigen, wie stark die Steuerprozesse ein geochemisches System verändern und gegebenenfalls zu einer massiven Freisetzung von Schadstoffen führen. Diese Effekte werden dadurch sichtbar, dass die Aufnahmefähigkeit des Feststoffs durch direkte Sättigung erschöpft wird, weisen aber zum Anderen auch auf die (technischen) Möglichkeiten hin, bestimmte Feststoffphasen als "Puffer“ oder „Barrieren“ einzusetzen. Vor allem Systeme, die hohe Anteile abbaubarer organischer Substanzen enthalten, zeigen typische nichtlineare, verzögerte Entwicklungen und sind – anders als mineralogisch-geochemische Systeme – mit den klassischen Methoden nicht ausreichend beschreibbar, modellierbar und prognostizierbar [238-243]. Die komplexen Vorgänge bei simultanen Freisetzungs- und Festlegungsprozessen von Schadstoffen, wie sie beim Übergang von Flüssen ins Meer stattfinden, wurden von Jihua Hong in einer Dissertation am Beispiel des Elbe-Ästuars mit Laborexperimenten und Modellrechnungen untersucht [244].

1. ***Elbesedimentforschung, vorwiegend Bestandsaufnahmen, in den 1990er Jahren***

Nach der deutschen Wiedervereinigung rückte die Sanierung der Elbe als Gemeinschaftsaufgabe der Bundesrepublik Deutschland und der Tschechischen Republik in den Vordergrund. Erstmals bestand die Möglichkeit den Istzustand der Schadstoffbelastung der gesamten Elbe mit modernen Methoden systematisch zu erfassen, das über die Gewässersedimente zu erwartende Gefährdungspotential zu ermitteln, Sanierungsschwerpunkte aufzuzeigen und Sanierungsmaßnahmen einzuleiten. In mehreren BMBF-Verbundvorhaben im Rahmen des Leitprojektes „Elbe 2000“ erfolgte seit 1992 eine Analyse der Belastung des Elbeeinzugsgebiets mit Schwermetallen, gekoppelt mit orientierenden Unter­suchungen zur organischen Belastung (BMBF [245]). Wegen der Herkunft der Belastungen wurde der Schwerpunkt auf die Untersuchung der Nebenflüsse gesetzt. Auf Grund der großen regionalen Varianz der geogenen Hintergrundbelastungen im Elbeeinzugsgebiet wurden seit 1995 im BMBF – Verbund­vor­haben *„Geogener Background“* auch detaillierte Untersuchungen zu geogenen Hintergrundwerten durchgeführt [246].

Eine breite Diskussion der Ergebnisse im Hinblick auf die Ableitung weitergehender Empfehlungen für die Schadstoffbewertung und Möglichkeiten der Sanierung der Elbe sowie des Elbeeinzugsgebiets erfolgte u.a. auf dem:

- 6. Magdeburger Gewässerschutzseminar „Die Elbe im Spannungsfeld zwischen Ökologie und Öko­nomie“, 8. - 12. November 1994. Cuxhaven [247, 248]
- 7. Magdeburger Gewässerschutzseminar „Ökosystem Elbe - Zustand, Entwicklung und Nutzung, gekoppelt mit dem BMBF-Statusseminar „Ergebnisse der Elbeforschung“, 22. - 25. Oktober 1996. Budweis [249, 250]
- 8. Magdeburger Gewässerschutzseminar „Gewässerschutz im Einzugsgebiet der Elbe“, 20. - 23. Oktober 1998. Karlsbad [246]

Trends und Verteilungsmuster der Schadstoffbelastung von Sedimenten aus östlichen Bundes­wasser­straßen wurden von Heininger & Pelzer [251] untersucht. Durch Prange et al. [252] konnte das Ele­ment­verteilungsmuster in Elbsedimenten in Zusammenhang mit Teileinzugsgebietsbesonderheiten dargestellt werden, wobei zur vergleichenden Bewertung der Ergebnisse die Normierung der Konzen­trationen u.a. mit Scandium erfolgte. Die Ermittlung der geogenen Hintergrundwerte im Elbeeinzugs­gebiet sowie Saale-Einzugsgebiet (A. Müller et al. [253]) ermöglicht die Aufstellung von realistischen Zielvorgaben für die Schwermetallbelastung der Schwebstoffe bzw. Sedimente; die Autoren quanti­fizierten auch die Senkenfunktion des Muldestausees und der Stauhaltungen in der Weißen Elster für schwermetallbelastete Sedimente.

1. ***Erfahrungen mit flächenhaft kontaminierten Feststoffen(Beispiele von der Mittelelbe)***

Beim Umgang mit kontaminierten Sedimenten und Baggergut standen bis in die 90er Jahre Problem­lösungen für Häfen am Unterlauf großer Flüsse im Vordergrund. Bereits im Vorfeld der EU-Wasser­rahmenrichtlinie fanden auch die Sedimentkontaminationen im Ober- und Mittellauf der Flüsse größe­re Beachtung. Die Verschmutzungen werden an den Schwebstoffen und Böden angereichert und mit dem fließenden Wasser entlang des Flußlaufes transportiert und ab- und umgelagert. Daraus resultieren z.T. hohe flächenhafte Schadstoffbelastungen in Böden und Sedimenten (Tabelle 14).

| **Kategorie** | **Problem** | **übliche Maßnahmen** |
| --- | --- | --- |
| Sedimentbildung in Vorflutern | Ansammlung schadstoffhaltiger Sedimente in industriellen Vor­flutern z.B. der chemischen Industrie, von Hüttenwerken | Analyse der Schadstoffbelastungen,  Monitoringmaßnahmen, ggf. Ent­nahme von Schadstoffherden |
| Hochwasser­sedimente | Oderflut 1997, Rheinhochwasser 1992: Kontamination der Fluss­sedi­mente und der vom Hoch­wasser betroffenen Flächen [254, 255] | Analyse der Schadstoffbelastungen,  Monitoringmaßnahmen, Nutzungs­­einschränkungen, Entnahme von Schadstoffherden bei Notwendigkeit |
| Überflutungs­sedimente in Deichvorländern | Auen und Marschen als Senke für belastete Sedimente [256-260] | Analyse der Schadstoffbelastungen,  Monitoringmaßnahmen, Nutzungs­einschränkungen |
| Spülfelder für  Hafenschlick | Stoffeintrag in das Grund- und Ober­flächenwasser aus Hafen-schlick-Spülfeldern [261, 262] | Analyse der Schadstoffbelastungen, Monitoring, Sicherungsmaßnahmen |

**Tabelle 13**: Beispiele für Erfahrungen mit flächenhaft kontaminierten Feststoffen [24]

Bei extremen Hochwasserereignissen kann es zu massiven Zerstörungen im Siedlungsbereich kom­men. So wurden bei dem Elbe-Hochwasser im August 2002 auch kommunale Klärsysteme nachhaltig gestört und hoch belastete Abwässer mit dem Überflutungswasser vermischt. Das mit gelösten und festen organischen Komponenten angereicherte Überflutungswasser überschwemmte Böden und Sedi­mente, die ihrerseits stark mit Schadstoffen kontaminiert sind, wie z.B. im Niederungsgebiet Mulde/­Spittelwasser bei Bitterfeld [263- 267]. Für die organischen Schadstoffe aus dem Raum Bitterfeld wurde als unmittelbare Flutfolge festgestellt, dass hochbelastetes Sediment aus der Vereinigten Mulde und dem Spittelwasser ausgeräumt und auf umliegende Flächen verfrachtet worden ist (Franke et al. [268]). Über die dioxinreichen Sedimente im Spittelwasser [10-12] und die frühen Vorschläge für Maßnahmen [7] wurde im Kapitel 2 berichtet.

In den Stillwasserbereichen der Mittelelbe lagern erhebliche Mengen an durch kommunale und indu­s­tri­elle Einleitungen geprägten schwebstoffbürtigen Sedimenten. Durch Schwartz & Kozer­ski [269] wurde gezeigt dass ein Großteil der in zahlreichen vorangegangen Niedrig- und Mittelwasserphasen abgelagerten Sedimente während des extremen Hochwassers remobilisiert werden kann. Dadurch können neben den frischen Sedimenten auch erhebliche Mengen an mit Nähr- und Schadstoffen stärker beladenen älteren Sedimenten zurück in das Flusssystem gelangen.

Bei den Untersuchungen von Schwebstoffen entlang der Elbe wurden von Pepelnik et al. [270] unge­fähr ein Jahr nach dem Hochwasserereignis wieder extreme Verhältnisse vorgefunden: Durch lang an­dauerndes Trockenwetter war es zu besonders niedrigen Abflussraten und zu einer starken Algenblüte gekommen. Dadurch wurde eine starke biologische Entkalkung ausgelöst, zusammen mit einer Verrin­gerung der meisten Elementkonzentrationen in den Schwebstoffen und Sedimenten.

Eine übergreifende Bewertung der während des *„Jahrhunderthochwassers“* 2002 in die Elbe eingetra­genen Stoffe erfolgte durch Vergleich mit Ergebnisse von Hochwasseruntersuchungen der Jahre 1995, 1999, 2000 und 2003 sowie von Untersuchungen während der Niedrigwasserperiode 2003 (Baborow­ski et al. [271]):

- während des Augusthochwassers 2002 wurden ungewöhnlich hohe Konzentrationen an *Kolloiden* transportiert;
- innerhalb der gemessenen Spurenmetalle stellen *As* und *Pb* die Problemelemente des August-Hoch­wassers 2002 für den unterhalb der Messstelle Magdeburg gelegenen Gewässerabschnitt dar;
- für den Unterlauf der Elbe sind *Schwermetalleinträge aus der Saale* bezogen auf die Elemente Cu, Hg, Ni, U und Zn von Bedeutung;
- der Schwermetalltransport bei Hochwasser erfolgt *elementspezifisch zeitlich und räumlich* unter­schiedlich.

Die Ergebnisse zeigen noch vorhandene Belastungen durch Schwermetalle und Arsen im Einzugs­gebiet der Elbe. Hierbei handelt es sich um typische Bergbaubegleitelemente: *„mit Einträgen aus den Hinter­lassenschaften des Altbergbaus ist auch bei künftigen Extremereignissen zu rechnen“* [271].

1. ***Sedimentdynamik und Schadstoffmobilität in Fließgewässern (Sedymo 2002-2006)***

Von diesen stark mit anorganischen und organischen Schadstoffen kontaminierten Auenböden/-sedi­menten geht eine akute Gefährdung durch Schadstoffverlagerung aus, die auf zwei unterschiedlichen Wegen erfolgen kann [272]:

1. *Biochemische Mobilisierung.* Durch den Eintrag leicht zersetzbarer organischer Komponenten mit dem Überschwemmungswasser werden organische und oxidische Bodenkomponenten mobilisiert bzw. aufgelöst, so dass Schadstoffe freigesetzt und mit dem abströmenden Porenwasser ausgetragen werden.

2. *Erosion.* In Abhängigkeit von Fließgeschwindigkeit und struktureller Stabilität des Boden-/Sed­i­ment­­körpers kommt es zum Abtrag von kontaminierten Boden-/Sedimentpartikeln. Das Spektrum umfasst kolloidale sowie tonig bis schluffige Partikel, die mit dem abströmenden Wasser verfrachtet und in entfernt liegenden Flussauen und Hafenbecken abgelagert werden.

In einem Teilprojekt anlässlich des BMBF-Forschungsprogramms „Nach dem Elbe-Hochwasser 2002“ wurden die beiden Mechanismen mit unterschiedlichen, neuartigen experimentellen Ansätzen an dem Kernprofil einer Überflutungsfläche im Bereich der Mittelelbe untersucht [272]. Die sog. SETEG-Experimente, die unter Mitwirkung der Arbeitsgruppe von Prof. Westrich, Institut für Wasserbau der Universität Stuttgart [273, 274] durchgeführt wurden, zeigen eine relativ geringe Erosionsstabilität der obersten Sedimentschichten. Die Säureneutralistionskapazität gibt einen ersten Hinweis für die Schwer­metall­stabilität in der Kornmatrix gegenüber (bio-) chemischen Prozessen, die zu einer Säure­bildung führen. Als Maß für die Säureneutralisationskapazität wurde der Verbrauch von Hydroxo­nium-Ionen gewählt, der durch das Sediment bei einem pH-stat-Versuch bei pH 4 über 24 Stunden beobachtet wird. Das Fazit der Untersuchungen war, dass Maßnahmen gegen eine Mobil­isierung von Schadstoffen aus Böden und Sedimenten bei Flutereignissen mit hoher Priorität an der Verbesserung der Erosionsstabilität ansetzen muss. Eine verringerte Erodierbarkeit von kontaminierten Feststoffen erfolgt bereits durch natürliche, sog. „diagenetische“ Effekte (bspw. Kompaktion, Verfesti­gung, Rekri­stallisation, Mitfällung), kann aber z.B. bei Überflutungssedimenten durch Bepflanzung auch die bio­chemische Schadstoffmobilisierung weiter reduzieren [275].

Die erwähnten Experimente zur Erosionsstabilität standen im Zentrum des BMBF-Verbundprojekts „Feinsedimentdynamik und Schadstoffmobilität in Fließgewässern“ (Sedymo 2001-2006 [276, 277]). In 13 Teilprojekten wurden u.a. zwei sedimentbezogene Fragenkomplexe der EG-Wasserrahmen­richtlinie (WRRL) bearbeitet:

I. *Fragen der Sedimentstabilität und der Auswirkungen von Erosionsereignissen*: Eine Veränderung dieser Stabilität führt nicht nur zwangsläufig zu Materialverlust und Umlagerung, einhergehend mit einer direkten erosiven Zerstörung bzw. Überschüttung des Benthos, sondern beeinflußt durch die erhöhte Trübung direkt die Benthosgemeinschaft, deren Monitoring ein Bestandteil der WRRL ist.

II. *Fragen der Charakterisierung von Prozessen*, die zur Umsetzung und zum Transfer von Schadstof­fen in den Sedimenten und an den Schwebstoffen führen: Hierzu benennt die WRRL verschiedene prioritäre Stoffe, zu denen u.a. die sedimentrelevanten organischen Halogen­verbindungen, Schwer­metalle und Eutrophierungsstoffe zählen.

Im Verlauf der Durchführung des Verbundprojektes sind eine Reihe neuer, für diese Thematik charak­teristische Aufgabenstellungen hinzugetreten, insbesondere im Hinblick auf die Schadstoff­remobilisie­rung bei der Erosion historisch kontaminierter Sedimente, die u.a. in zwei großen flussgebietsübergrei­fenden Studien am Rhein (Heise et al. 2004 [202], Auftraggeber: *Port of Rotterdam*) und an der Elbe (Heise et al. 2005 [42], *Hamburg Port Authority*).

Bei der Umsetzung von Mobilitätsdaten sei auf die Klassiker „Nutzung von partikulärer Materialien“ [278], „konzeptioneller Rahmen für eine flussgebietsübergreifende Sedimentbewirtschaftung [279] und „Sedimentregulierungen und Monitoringprogramme in Europa“ [280] hingewiesen. Auch der Aspekt der *Qualitätssicherung bei der Untersuchung von Sedimentproben* [281-285] hat durch die sedimentdynamischen Betrachtungen eine neue Dimension erhalten (Kasten 11 nach [281]).

**Kasten 11 Das Konzept der Rückverfolgbarkeit für verschiedene *Sedimenteigenschaften* [281]**

Durch die Mechanismen der bevorzugten Anreicherung von persistenten Schadstoffen resultieren für die Schwebstoffe und Sedimente mehrere Funktionen im Hinblick auf die Gewässerchemie und –öko­lo­gie (Abb. 8): Bei ausreichender Fixierung der Kontaminanten an den Partikeln und anhaltender Ablagerungstendenz der Feststoffe können Sedimente als Schadstoffsenken wirken: eine zeitliche Abfolge von konservierten Ablagerungsperioden kann die Entwicklung der regionalen Schadstoff­belas­tung dokumentieren – Sedimente als ‘Gedächtnis’ des Gewässers. Für die Gewässerökologie bedeutet die Bindung von Schadstoffen an die Feststoffphasen grundsätzlich eine Entgiftung der Lösungsphase; allerdings können auch feststoffgebundene Schadstoffe toxische oder kanzerogene Wirkungen hervor­rufen, die bei immissionsbezogenen Zielvorgaben für das Schutzgut Sedimente berücksichtigt werden müssen – Sedimente als ‘Lebensraum’ (2. Spalte in Abb. 8). Die Neigung zur Feststoff­bindung beinhaltet auch den Aufbau eines Schadstoffpotentials, aus dem bei veränderten Gewässer­bedingun­gen giftige Substanzen freigesetzt werden können: bei Überlastung oder Abbau wichtiger pH- oder Redoxpuffer kann eine solche Remobilisierung plötzlich und intensiv erfolgen und damit den Cha­rak­ter einer ‘chemischen Zeitbombe’ annehmen – Sedimente als ‘Sekundärquellen’ für Schad­stoffe (3. Spalte in Abb. 8). Die gemeinsame Untersuchung der (bio-)chemischen und hydrauli­schen Stabi­lität von Sedimenten spielt im Rahmen der flussgebietsübergreifenden Gewässer­bewirt­schaf­tung eine immer größere Rolle.

Abb. 8: Chemische Analysen für drei wichtige Sedimentfunktionen unter besonderer Berück­sichtigung der Rückverfolgbarkeit einzelner Bearbeitungsschritte (Förstner [281]).

X = Sedimentspezifische Eigenschaft; RM = Referenzmaterial; Doc = Dokumentierte Methode, TRIAD = Chemischer Teil des Ansatzes der Sediment-Triade für die ökologische Gefährdungs­abschätzung (Chapman [286]). AVS/ΣSEM = Acid Volatile Sulfide / Sum Simultaneously Extractible Metal (DiToro et al. [287]). * Originalprobe (Aufbereitung nach Mudroch und Bourbonniere [288]); Porenwasseruntersuchungen: Carr und Nipper [289]; sequentielle Extraktion: Quevauviller [290]). ** Kombination von Untersuchungen an Original-Sedimentkernen zur chemischen und hydraulischen Stabilität (Haag et al. [291]).

Das zentrale Konzept der ‘Traceability’ verlangt, dass Umweltmessdaten sich (i) auf definierte Referenzen (Materialien und Methoden), (ii) über eine ununterbrochene Kette von Vergleichsmöglich­keiten, (iii) mit festgestellten Unsicherheiten, rückverfolgen bzw. rückführen lassen (Quevauviller [292]).

1. ***Untersuchungen von mobilisierenden Einflussfaktoren (Beispiele aus JSS 2007-2012)***

Das Erosionsverhalten der feinkörigen, kohäsiver Sedimente, die aufgrund ihrer physikochemischen Eigenschaften als Hauptträger von Schadstoffen fungieren, ist für natürliche Strömungsfelder schwie­rig zu erfassen. Laborexperimente bieten die Möglichkeit, Erosionsversuche unter definierten reprodu­zierbaren Rahmenbedingungen durchzuführen (siehe oben [225]). Die nachfolgend wiedergegebenen Beispiele, die aus der Zeitschrift „Journal of Soils and Sediments“ (2007-2012) ausgewählt wurden, simulierten die Freisetzung von Schadstoffen bei unterschiedlichen Geässerbedingungen, vor allem bei Hochwasserereignissen.

Die von Gerbersdorf et al. [293] am Institut für Wasserbau der Universität Stuttgart gemessenen Para­meter können beispielhaft für die hier aufgeführten Fallstudien gelten: Die kritische Erosionsschub­span­nung wurde mit einem Strömungskanal zur Ermittlung des tiefenabhängigen Erosionsverhaltens von Sedimenten („SETEG“, Be02 in [225]) an Sedimentkernen aus Stauhaltungen des Neckars gemes­sen, an unterschiedlichen räumlichen Positionen und zu verschiedenen Jahreszeiten, um die Übertrag­barkeit der Daten, u.a. hinsichtlich der biologischen Stabilisierung durch extrazelluläre polymere Sub­stanzen (EPS), abzusichern; weiterhin wurde tiefenabhängig die Korngrößenverteilung, die Kationen­austauschkapazitäten (CEC) sowie die Konzentrationen an gesamtem organischen Kohlenstoff (TOC), Proteinen und Kohlenwasserstoffen (wasser- und harzextrahierbar) bestimmt. Die Ergebnisse aus den fünf Fallbeispielen sind:

(1) Die Studie von Gerbersdorf et al. [293]) zeigte, dass der Erosionswiderstand von interpartikulären Kräften, dem Wechselspiel von biologisch produzierten Substanzen als aktive Oberflächen, der Bindungskapazität und der Ladungsdichte der Sedimente bestimmt wird. Will man eine realistische Risikobestimmung vornehmen, muss man das Stabilisierungspotenzial der Mikroorganismen und auch die wechselseitigen Änderungen der biologischen und physiko-chemischen Eigenschaften berücksichtigen.

(2) Bei Versuchen mit einem Reaktortyp namens SPEED (Solid Phase Extraction with External Desorp­tion) konnten Smit et al. [294] nachweisen, dass Flutereignisse zu einer Verstärkung der Desorption persistenter organischer Schadstoffe (POP) von Sedimenten in das umgebende Wasser führt. Die hydraulische Verweilzeit (HRT, 10 – 750 min) und die Partikelgröße bestimmen den Konzentrationsgradienten und damit auch die Desorptionsrate. Die Autoren nehmen an, dass die nichtstationäre Diffusion den wichtigsten Prozess für den Massentransfer in die Wasserphase darstellt.

(3) De Weert et al. [295] untersuchten in Experimenten mit einem Kontinuierlichen Durchflussreaktor die Desorption, den Massentransfer und den biologischen Abbau unter abgesetzten und resuspen­dierten Sedimentverhältnissen von verzweigtem Nonylphenol, das als Modellbeispiel für aerob abbaubare und anaerob persistente Substanzen eingesetzt wurde. Die Ergebnisse zeigen, dass Nonylphenol bei niedrigen Konzentrationen kontinuierlich aus dem Sediment in den Wasserkörper desorbiert und dort nahezu vollständig abgebaut wird.

(4) Die Studie von Wölz et al. [296] ist Teil von Untersuchungen über die Gefahren durch Schadstoffe an überfluteten Standorten und Rückhaltegebieten, die bei Hochwasserereignissen am Rhein über­flutetet werden. Die Laboruntersuchungen dienten zunächst dazu, biologische Effekte an Schweb­stoffen zu erfassen und bestimmte Substanzklassen zu identifizieren und zu quantifizieren. Es zeigt sich, dass die Konzentrationen der Schadstoffe mit dem Wasserdurchfluss ansteigen. Gefahren sind nicht nur durch persistente, unpolare Stoffe sondern gleichermaßen durch weniger persistente und stärker polare Substanzen zu erwarten.

(5) In der Untersuchung von Brinkmann et al. [297] wurden Regenbogenforellen (Oncorhynchus mykiss) über 5 Tage in einem Gerinne mit PAK-dotiertem Sediment simulierten Flutbedingungen ausgesetzt. Eine Folge von verschiedenen molokularen, biochemischen und histologischen Mar­kern wurde verwendet um die Hypothese zu überprüfen, dass die Resuspension von Sedimenten zu einer Remobilisierung von PAK und damit zu nachteiligen Effekten bei den aquatischen Organis­men führen kann. Erste Ergebnisse zeigen, dass die Ausscheidungsprodukte über die Gallenwege die empfindlichsten Marker für eine PAK-Exposition bilden.

Die Studie von Gerbersdorf et al. [293] und die Untersuchungen unter AquaTerra [295], Modelkey [296] und FloodSearch [297] zeigten charakteristische Wirkungen von organischen Substanzen auf biostabilisierende Prozesse an Sedimenten durch mikrobielle Anordnungen (d.h. Mikrobiologie und Technik). Der Hauptvorteil des experimentellen Ansatzes von FloodSearch an der RWTH Aachen besteht darin, dass alle wesentlichen Prozessparameter (Hydraulik, Sedimenterosion, Bioverfügbarkeit von sedimentgebundenen Kontaminenten und ökotoxikologische Wirksamkeit sowohl *in vitro* als auch *in vivo*) in einer einzigen Modellanlage simuliert werden [298].

Aus einer erweiterten Sicht empfehlen Gerbersdorf et al. [299], diese Befunde als Grundlage für einen integrierten Ansatz der beteiligten Disziplinen wie bspw. die Ökotoxikologie zu verwenden und ein Bewertungsschema zu entwickeln, das sich an die „Sedimentqualitätstriade“ von Chapman [286] anlehnt (der ausdrücklich eine Ausweitung seines Konzepts und dessen Komponenten angeregt hatte).

Fazit: Die hohe Relevanz der Sedimentremobilisierung, die dadurch erhöhte Bioverfügbarkeit von Schadstoffen aus kontaminierten Altsedimenten und ihre nachteiligen Wirkungen auf Ökosystem und menschliche Gesundheit müssen besonders in Folge der zunehmenden Wahrscheinlichkeit von extre­men Hochwassern berücksichtigt werden (Hollert et al. 2014 [300]).

**Nachwort**

Die Übersicht zu Dioxin und Sedimentlasten im Elbeeinzugsgebiet unter der Wasserrahmenrichtlinie 2000-2015 zeigt zuerst eine wissenschaftliche Erfolgsgeschichte auf, in einer Disziplin, die eigentlich von der europäischen Gewässeradministration gar nicht vorgesehen war – die Sedimentforschung. Mein akademischer Lehrer German Müller (1930-2007), an dessen Werk ich mit dieser Übersicht erinnern möchte, hatte seit Anfang der 1960er Jahre bei der Qualitätsprüfung von Gewässern die Zusammensetzung der Feststoffe der Wasserphase grundsätzlich gleichberechigt gegenübergestellt.

Für die Bewertung hinsichtlich eines „guten Zustands“ über Flusseinzugsgebiete hinweg erschienen Sedimente anfänglich als zu „sperrig“; man erinnert sich noch an vielen Ansätze landauf und landab, Wasserdaten in Sedimentwerte umzurechnen, und umgekehrt. Einige ahnten, dass es mit eigen-ständigen Datensätzen für Sedimente und Biota überhaupt keine guten Zustände mehr geben würde – was sich inzwischen, u.a. durch Untersuchungen am Umweltbundesamt, immer klarer abzeichnet.

Dass mit den jüngsten Erweiterungen der WRRL im Bereich der prioritären Stoffe den festen Matrizes immer mehr der Vorrang eingeräumt wurde, war nur möglich durch die Überzeugungsarbeit von einer wachsenden Zahl von renommierten Wissenschaftlern im Dienste der Brüsseler Administration, an der Spitze, für viele Jahre, Philip Quevauviller. Es war aber auch ein Verdienst der ad hoc Arbeitsgruppe Schadstoffe bei der Flussgebietsgemeinschaft Elbe (Leitung: Peter Heininger) mit ihrem „Hintergrund-papier zur Ableitung der überregionaler Bewirtschaftungsziele“.

Der aktualisierte Bewirtschaftungsplan der FGG Elbe zeigt, dass das Thema „historisch kontaminierte Sedimente“ deutlich ins Zentrum der Wasserrahmenrichtlinie gerückt ist und mit ihrem Sediment-managementkonzept von 2013, wieder ausgearbeitet von der ad hoc AG Schadstoffe, nimmt die Elbe erneut eine Vorreiterrolle in der internationalen WRRL-Community ein. Dieser Erfolg sollte aber nicht darüber hinwegtäuschen, dass die eigentlichen Herausforderungen noch vor dem Flusseinzugsgebiet Elbe liegen. Die Nagelprobe wird die Meeresschutz-Richtlinie sein und unverzichtbar ist dabei die Verknüpfung mit dem Hochwasserschutz nach den diversen Jahrhundertfluten – bereits fünf in diesem Jahrtausend. Das Verschlechterungsverbot wird vermutlich eine immer größere Rolle spielen. In den Amtsstuben in Magdeburg und Hamburg kann man die Akte „Dioxin“ noch nicht schliessen.

Um die Hindernisse bei der Bewältigung eines der vordringlichsten Umweltprobleme, der Sanierung historisch kontaminierter Sedimente, zu überwinden, bedarf es einer pragmatischen Vorgehensweise, bei der das Know How von Forschungsinstitutionen, international tätigen Consultingfirmen und wissenschaftlich-technischen Gesellschaften zusammengeführt wird. Beim Übergang in die zweite Phase der Wasserahmenrichtlinie sollte sich auch die Politik stärker dieses Themas annehmen.

**Dank**

Der auf Erkenntnisvertiefung und Nachhaltigkeit ausgerichtete wissenschaftliche Ansatz besitzt ein besonderes Gewicht bei der Beurteilung von langfrstigen Entwicklungen. Die vorliegende Zusammen­stellung zeigt sowohl die negativen Folgen einer eigenmächtigen Behördenmentalität als auch die Potenziale, die eine über vierzig Jahre gewachsene Kultur an Fachwissen bereitstellen kann. Hier sei an wichtige Stationen und Akteure auf dem Weg zu dem Thema dieses Berichts^[[15]](#footnote-15)^ erinnert:

Die umweltbezogene Sedimentforschung und die Entwicklung des Praxisgebiets „Sedimentaltlasten“ in Deutschland haben ihre Wurzeln in der Deutschen Forschungsgemeinschaft. Mit dem Schwerpunkt-programm „Schadstoffe im Wasser“ (1970-1977 [218]) begann eine systematische Erforschung kontaminierter Sedimente und Schwebstoffe, parallel zu den Aktivitäten der Bundesanstalt für Gewässerkunde. Die Kommission für Wasserforschung wurde 1984 neu ausgerichtet und wurde bis zu seinem Tod im Jahre 1998 von Herrn Dr. Ulrich de Haar in der DFG-Geschäftsstelle betreut.

Unter maßgeblicher Mitwirkung von Ulrich de Haar wurden in zwei Rundgesprächen die Möglich-keiten für ein DFG-Schwerpunktprogramm „Sedimentdynamik und Schadstoffmobilität“ untersucht:

- „Hydromechanische und chemische Aspekte des Schwebstofftransportes in staugeregelten Fließ-gewässern“ (Juli 1987, Leitung: Prof. Dr. H.H. Hahn, Karlsruhe)
- „Die Schwebstoffdynamik in Fließgewässern und ihre Bedeutung für den Transport umweltrele-vanter Schadstoffe“ (März 1993, Prof. Dr. W. Symader, Trier)

Im Februar 2000 wurde mit Unterstützung der Kommission für Wasserforschung (Vorsitz: Prof. Dr. Helmut Kobus) und Vermittlung der DFG-Geschäftstelle (Dr. Ute Weber) eine Verbundprojektskizze zum Thema „Feinsedimentdynamik und Schadstoffmobilität in Fließ-, Ästuar- und Küstengewässern“ bei den BMBF-Projektträgern für die Bekanntmachung „Flußeinzugsgebietsmanagement“ eingereicht. „SEDYMO“ mit 19 Teilprojekten wurde ab 2002 beim Projektträger Karlsruhe, Wassertechnologie und Entsorgung (Betreuerin: Dr. Verena Höckele) durchgeführt und im März 2006 mit einem inter-nationalen Symposium offiziell abgeschlossen. Der kollegiale Dank gilt den Mitkoordinatoren dieses nationalen Forschungsverbundes, Gerhard Jirka, Lutz Arend Meyer-Reil und Bernhard Westrich.

Für die europäischen und teilweise weltweiten Forschungsarbeiten war die Kooperation mit Wim Salomons besonders wichtig, die 1984 mit mehreren gemeinsamen Buchprojekten begann, in dem niederländischen Programm „Chemical Time Bombs“ (1990-1994) fortgeführt wurde und über das von Wim Salomons in Geesthacht koordinierte Vorhaben „PoR II Dredged Material in the Port of Rotterdam – Interface between the Rhine Catchment Area and North Sea“ (1998-2000) einerseits in das europäische Sedimentnetzwerk „SedNet“ im 5. EU-Forschungsrahmenplan (2001-2005) und andererseits in die Flussgebietsstudien zu Sedimentbelastungen und deren Risiken für die Häfen von Rotterdam (Heise et al. 2004, mit B. Westrich und W. Salomons, finanziert von Port of Rotterdam) und Hamburg (Heise et al. 2005 und 2008, finanziert von der Hamburger Hafenbehörde) einmündete.

Seit Mitte der ersten WRRL-Phase bedienen sich die Behörden in der Flussgebietsgemeinschaft Elbe zunehmend außeruniversitärer Kooperationen mit Publikationen in deutscher Sprache. Unser Dank gilt deshalb Henner Hollert und der Springer Open Access Zeitschrift „Environmental Sciences Europe“, die uns hier einen breiteren internationalen Erfahrungsaustausch ermöglicht haben.

**Literatur I**^[[16]](#footnote-16)^

[1] Anonym (1990) Das gibt Revolte. Neue Hiobsbotschaften aus Bitterfeld und Umgebung: Die Dioxinwerte liegen höher als einst in Seveso. Die Fakten will niemand so recht wahrhaben. Der Spiegel 40/1990, S. 46-54. [http://www.spiegel.­de­/­spiegel/print/d-13500306.html](http://www.spiegel.de/spiegel/print/d-13500306.html)

[2] Bonnenberg H (2010) Meine Erfahrungen bei der Treuhandanstalt und ihren Folgegesellschaften. Vortrag anlässlich der Anhörung der Bundestagsfraktion DIE LINKE. Marie Elisabeth Lüders Haus des Bundestags, Berlin, 19. April 2010. 16 S. <http://www.treuhandanstalt.com/uploads/media/Treuhandanstalt_DIE_LINKE__01.pdf>

[3] Anonym (2010a) 10 Jahre Landesanstalt für Altlastenfreistellung in Sachsen-Anhalt. Magdeburg, 32 S. [http://www.laf-lsa.de/fileadmin/Bibliothek/Politik_und_Verwaltung/MLU/LAF/Dokumente­/20100127_Broschuere_LAF_fuer_Inter­net.pdf](http://www.laf-lsa.de/fileadmin/Bibliothek/Politik_und_Verwaltung/MLU/LAF/Dokumente/20100127_Broschuere_LAF_fuer_Internet.pdf)

[4] Anonym (2000a) Richtlinie 2000/60/EG des Europäischen Parlaments und des Rates vom 23.10.2000 zur Schaf­fung eines Ordnungsrahmens für Maßnahmen der Gemeinschaft im Bereich der Wasserpolitik (ABl. L 327 vom 22.12.2000, S. 1)

[5] Breuer R (2008) Sedimentmanagement für die Elbe. Rechtsgutachten, 234 S. Nomos Verlag Baden-Baden. [http://www­.­tideelbe.de/files/sedimentmanagement_fur_die_elbe.pdf](http://www.tideelbe.de/files/sedimentmanagement_fur_die_elbe.pdf)

[6] Götz R, Steiner D, Friesel P, Roch K, Walkow F, Maaß V, Reincke H (1996) Dioxin in the River Elbe – investigations of their origin by multivariate statistical methods. Organohalogene Compounds 27: 440-443

[7] Anonym (1993a) Machbarkeitsstudie zur Sanierung des Spittelwassersediments. UBS Schwerin, IGB Hamburg im Auf­trag des Landratsamtes Bitterfeld, Juli 1993. (Unveröffentlicht).

[8] Lindemann M (2000) Das ökologische Großprojekt Bitterfeld-Wolfen – eine Bilanz aus fachlicher Sicht. Altlasten Spektrum 4/2000, S. 208-214

[9] Anonym (1992a) Alles durch den Ausguss – die ostdeutschen Behörden schaffen es nicht, ihre Umweltprobleme unter Kontrolle zu bringen. Der Spiegel 9/1992, 24.02.92 [http://magazin.­spiegel.de/EpubDelivery/spiegel/pdf/­13687070](http://magazin.spiegel.de/EpubDelivery/spiegel/pdf/13687070), S. 82-85.

[10] Anonym (1997a) Aktuelle Bewertung der Gefahrensituation des Spittelwassers I, 8/1997. Staatliches Amt für Umwelt­schutz Dessau/Wittenberg. (unveröffentlicht).

[11] Anonym (2000b) Aktuelle Bewertung der Gefahrensituation des Spittelwassers II, 10/2000. Staatliches Amt für Um­welt­schutz Dessau/Wittenberg. (Unveröffentlicht).

[12] Lindemann M, Wittmann U (2000) Fallstudie Spittelwasser in der Region Bitterfeld. Eine Altlast – vier Konzepts: Ein Nachtrag. TerraTech 6/2000, S. 20-21

[13] Anonym (2014a): Sedimentmanagement Tideelbe - Strategien und Potenziale - Systemstudie II. Ökologische Auswir­kungen der Unterbringung von Feinmaterial. Band 1 (2), Endbericht. Im Auftrag des Wasser- und Schifffahrtsamtes Hamburg. Bundesanstalt für Gewässerkunde, Koblenz, BfG-1763. DOI: 10.5675/BfG-1763

[14] Anonym (2013a) Richtlinie 2013/39/EU des Europäischen Parlaments und des Rates vom 12.08.2013 zur Änderung der Richtlinien 2000/60/EG und 2008/105/EG in Bezug auf prioritäre Stoffe im Bereich der Wasserpolitik. ABl. L 226 vom 24.08.2013, 17 S.

[15] Wenzel C (2012) Die EG-Meeresstrategie-Rahmenrichtlinie (MSRL) – Bedeutung für die Bewertung von Sediment­kontaminationen. In: Magdeburger Gewässerschutzseminar 2012 „Die Elbe und ihre Sedimente“. Hamburg 10./11. Oktober 2012, S. 72-76. 15 Folien: [http://www.elsa-elbe.de/assets/download/MGS-2012/Vortraege/4-MGS-Vortrag­_Wenzel.pdf](http://www.elsa-elbe.de/assets/download/MGS-2012/Vortraege/4-MGS-Vortrag_Wenzel.pdf)

[16] Jacobs P, Krautter N, Diesner K (2013) Frachtreduzierung Spittelwasser – Abschlussbericht. Oberflächenwasser-, Sediment- und Auenuntersuchungen; Bewertung und Ableitung von Maßnahmeerfordernissen. Projektleitung: Dr. Patrick Jacobs, Tauw GmbH, Berlin; 21. Oktober 2013. Im Auftrag der Landesanstalt für Altlastenfreistellung des Landes Sachsen-Anhalt. Berlin, 232 Seiten. <http://www.laf-lsa.de/wasserrahmenrichtlinie/> (25.07.2014)

[17] Harbodt K (2006) History until 1945. In: Friedrich HE, Mordike BL (Eds) Magnesium Technology – Metallurgy, Design Data, Applications. 685 p. Springer, Berlin-Heidelberg-New York

[18] Evers EHG, Laane RWPM, Groenefeld GJJ (1996) Levels, temporal trends and risks of dioxins and related com­pounds in the Dutch aquatic environment. Organohalogen Compounds. 28, 117 - 122

[19] Förstner (2011) Was ist aus der Dioxin-Altlast im Spittelwasser geworden? Die Rolle der Landesanstalt für Altlasten­freistellung von Sachsen-Anhalt bei der Umsetzung der EG Wasserrahmenrichtlinie im Flusseinzugsgebiet der Elbe, dargestellt am Beispiel des Pilotprojekts Bitterfeld-Wolfen, Dokumentation, 16 Seiten. [http://www.dioxindb.de/­doku­mente/Dioxin_Spittel­wasser_Dokumentation_Foerstner_2011](http://www.dioxindb.de/dokumente/Dioxin_Spittelwasser_Dokumentation_Foerstner_2011)

[20] Anonym (2000c) Contaminated Soil 2000, Case Study: Comparison of Solutions for a Large Contamination Based on Different National Policies. 7th International FZK/TNO Conference on Contaminated Soil. Leipzig. 163 p.

[21] Anonym (2000d) Case study of contaminated land management at Bitterfeld by UK team (Bob Harris, Mary Harris, Naomi Earl, Richard Swannell & Jonathan Smith. 18 Seiten

[22] Anonym (2000e) National approaches for a Bitterfeld Case – Danish Contribution (Tom Heron [team leader], Lizzi Andersen & Lars Kaalund). 16 Seiten

[23] De Wit N, Rijnaarts H, Faber J, Ykema K, Ouboter St, Satijn B (2000) Solutions for the Spittelwasser Pollutions – The Dutch Contribution for the Case Comparison Bitterfeld. A plan to be presented at ConSoil 2000 – FZK Inter­national Conference on Contaminated Land, 18-22 September 2000, Leipzig

[24] Anonym (2000f) Fallstudie Bitterfeld. Umgang mit Kontaminationen in Flusseinzugs- und Überschwemmungs­gebieten am Beispiel des Niederungsgebietes „Spittelwasser“ Bitterfeld. Deutscher Beitrag zum Fallstudienvergleich ConSoil 2000 vom 18.09.-22.09.2000 in Leipzig, 27 S. (Arbeitsgruppe des BMBF-Projektträgers für Abfallwirtschaft und Altlastensanierung: Wittmann U [Projektorganisation], Förstner U [Wissenschaftlicher Projektleiter], Gier S, Delschen Th, Franzius V, Frauenstein J, Fuchs E, Gläßer W, Lindemann M, Müller R, Meiners G, Wetzel V, Wilke B

[25] Anonym (2004a) EU-UmwHaftRL – EU-Umwelthaftungsrichtline. Richtlinie 2004/35/EG des Europäischen Parlaments und des Rates vom 21.04.2004 über Umwelthaftung zur Vermeidung und Sanierung von Umweltschäden. Amtsblatt der Europäischen Union Nr. L143 vom 30.04.2004. S. 56-75

[26] Anonym (2007a) USchadG – Umweltschadensgesetz. Gesetz zur Umsetzung der Richtlinie des Europäischen Parla­ments und des Rates über Umwelthaftung zur Vermeidung und Sanierung von Umweltschäden vom 10. Mai 2007. Bundesgesetzblatt Teil I Nr. 19, S. 666-671

[27] Michels J, Stuhrmann M, Frey C, Koschitzky H-P (Hrsg., 2008) Handlungsempfehlungen mit Methodensammlung. Natürliche Schadstoffminderung bei der Sanierung von Altlasten. VEGAS, Institut für Wasserbau, Universität Stutt­gart, DECHEMA e.V. Frankfurt. ISBN-13 978-3-89746-092-0. 363 Seiten

[28] Gerth J, Förstner U (2011) Kontaminierte Gewässersedimente – 6.1.8 Projekt Flussauensedimente im KORA-The­men­verbund. In: Kern U, Westrich B (Hrsg.) Kontaminierte Gewässersedimente – Strategie, Fallbeispiele, Empfehlungen. DWA-Themen T 3/2011. Deutsche Vereinigung für Wasserwirtschaft, Abwasser und Abfall e.V. Hennef, S. 119-135

[29] Hoth N, Rammlmair D, Gerth J, Häfner F (2008) Leitfaden “Natürliche Schadstoffminderungs-prozesse an großräumi­gen ergbaukippen/-halden und Flussauensedimenten. Empfehlungen zur Untersuchung und Bewertung der natür­lichen Quelltermminimierung. 124 Seiten + Anlage 1 (Förstner U: „Erkenntnisse zu Monitored Natural Recovery an Fluss­sedi­menten“, 19 S.). BMBF-Förderschwerpunkt „Kontrollierter natürlicher Rückhalt und Abbau von Schadstoffe bei der Sanierung kontaminierter Grundwasser und Böden“ (KORA), TU Bergakademie Freiberg und DECHEMA, Freiberg/Frankfurt, November 2008

[30] Rijnaarts H, ter Meer J (2005) The WELCOME Integral Management Strategy for large scale historical soil and ground­water contamination. TNO, Knowledge for Business, NATO-CCMS, June 2005, Ottawa. 23 Folien. [http://­www­.­cluin.org/ottawa/](http://www.cluin.org/ottawa/); [www.welcome.intro.ppt](http://www.welcome.intro.ppt)

[31] Großmann J, Ritter A, Sievers J, Keil M, Bielke A, Weiß H (2005) Entwicklung einer integrierten Management­strategie (IMS) für Altlastengroßstandorte und Anwendung auf den Modellstandort des Ökologischen Großprojektes Bitterfeld. Altlasten Spektrum 4/2005, 181-186

[32] Anonym (2005a) EU-Forschungsvorhaben WELCOME (Water, Environment and Landscape Management at Conta­minated Megasites). Abschlussbericht 2005

[33] Heininger P (2009) Maßnahmen zur Reduzierung der Schadstoffbelastung. Ad hoc-AG „Schadstoffe“ der AG OW der FGG Elbe. Begleitung der Anhörung „Bewirtschaftungsplanung im deutschen Einzugsgebiet der Elbe“, Hamburg, 24.04.2009. Folie #29. Bundesanstalt für Gewässerkunde, Koblenz. [http://fgg-elbe.de/Veranstaltungen/magdeburg/­heininger_23-04-2009.pdf](http://fgg-elbe.de/Veranstaltungen/magdeburg/heininger_23-04-2009.pdf)

[34] Anonym (2005b) Freistellungsfinanzierte Altlastensanierung – Neue Herausforderungen in fachlicher, administrativer und finanzplanerischer Hinsicht. Tagung der Landesanstalt für Altlastenfreistellung des Landes Sachsen-Anhalt und des Ingenieurtechnischen Verbandes Altlasten, 9. Juni 2005 Magdeburg. 185 S. [http://www.sachsen-anhalt.de/­LP­SA­/-fileadmin/­Elementbibliothek/Bibliothek_Politik_und_Verwaltung/Bibliothek_LAF/PDF/­Tagung_09.06.2005_n.pdf](http://www.sachsen-anhalt.de/LPSA/-fileadmin/Elementbibliothek/Bibliothek_Politik_und_Verwaltung/Bibliothek_LAF/PDF/Tagung_09.06.2005_n.pdf)

[35] Wernicke P (2005) in Zitat [34] Seiten I-III

[36] Großmann J (2005) in Zitat [34] Seiten 45-54

[37] Rijnaarts HHM (2005) in Zitat [34] Seiten 55-64

[38] Milch W (2005) „Altlastensanierung in Sachsen-Anhalt unter Berücksichtigung der Wasser­rahmenrichtlinie und der neuen Wassergesetzgebung des Landes“. In: Zitat [34], Seiten 3-12

[39] Anonym (2010b) Landesbetrieb für Hochwasserschutz und Wasserwirtschaft Sachsen-Anhalt Gewässerkundlicher Landesdienst (LHW). Nr. 1/2010, 35 S. [http://www.sachsen-anhalt.de/LPSA/­fileadmin/Elementbibliothek/Master­Bibliothek/Landesbetriebe/LHW/neu_PDF/5.1/Dokumente_GLD/Dioxinbericht-2007-fertig.pdf](http://www.sachsen-anhalt.de/LPSA/fileadmin/Elementbibliothek/MasterBibliothek/Landesbetriebe/LHW/neu_PDF/5.1/Dokumente_GLD/Dioxinbericht-2007-fertig.pdf)

[40] Anonym (2005c) Chemischer Zoo – seit der Jahrhundertflut kommen die Hinterlassenschaften der DDR-Giftküche Bitterfeld an die Oberfläche. Der Spiegel v. 23.12.2005, S. 36

[41] Böhme M, Krüger F, Ockenfeld K, Geller W (Hrsg., 2005) Schadstoffbelastung nach dem Elbe-Hochwasser 2002. Broschüre UFZ – Umweltforschungszentrum Leipzig-Halle GmbH, Department. Fließgewässerökologie. 101 S. <http://www.ufz.de/data/HWBroschuere2637.pdf>

[42] Heise S, Claus E, Heininger P, Krämer T, Krüger F, Schwartz R, Förstner U (2005) Studie zur Schadstoffbelastung der Sedimente im Elbeeinzugsgebiet. Ursachen und Trends. Im Auftrag der Hamburg Port Authority, Nov. 2005, 168 S.

[43] Heise S, Krüger F, Förstner U, Baborowski M, Götz R, Stachel S (2008) Bewertung der Risiken durch feststoffgebun­dene Schadstoffe im Elbeeinzugsgebiet. Im Auftrag von Flussgebietsgemeinschaft Elbe und Hamburg Port Authority, Mai 2008, 349 S. <http://www.tideelbe.de/files/elbestudie_ii_mai_2008_klein.pdf>

[44] Anonym (1995) Dioxin-Bilanz für Hamburg. Studie der Universität Bayreuth und Trischler & Partner GmbH im Auf­trag der Umweltbehörde Hamburg, Sept. 1995. [https://www.hamburg.de/contentblob/112354/data/dioxinbilanz.­pdf](https://www.hamburg.de/contentblob/112354/data/dioxinbilanz.pdf)

[45] Götz R, Steiner D, Friesel P, Roch K, Walkow F, Maaß V, Reincke H (1998) Dioxin (PCDD/F) in the River Elbe – investigations of their origin by multivariate statistical methods. Chemosphere 37:1997-2002

[46] Götz R, Lauer R (2003) Analysis of sources of dioxin contamination in sediments and soils using multivariate statisti­cal methods and neural networks. Environ Sci Technol 37:5559-5565

[47] Uhlig S, Simon K, Kunath K (2008) Dioxine und Furane in Elbe, Mulde und Spittelwasser: Statistische Analyse der Kongenerenmuster. Quo data, Gesellschaft für Qualitätsmanagement und Statistik mbH, Anl. 1 zur HPA-Risikostudie, 28.05.2008, 60 S. [http://www.tideelbe.de/files/anlage_1_d ioxine_und_furane_in_elbe_mulde_und_spittelwasser.pdf](http://www.tideelbe.de/files/anlage_1_d%20ioxine_und_furane_in_elbe_mulde_und_spittelwasser.pdf)

[48] Stachel B, Götz R (2008) Die Kontamination von Elbaueböden, Elbefischen sowie Futter- and Lebensmitteln mit Dioxinen und dioxin-ähnlichen PCB, Anlage 13 zur HPA-Riskostudie, 28. Mai 2008, 26 S. [http://www.tideelbe.de/­files­/­an­lage_13_sonderkapitel_dioxine.pdf](http://www.tideelbe.de/files/anlage_13_sonderkapitel_dioxine.pdf)

[49] Stachel B, Christoph EH, Götz R, Herrmann T, Krüger F, Kühn T, Lay J, Löffler J, Päpke O, Reincke H, Schröter-Kermani C, Schwartz R, Steeg E, Stehr D, Uhlig S, Umlauf G (2006): Contamination of the alluvial plain, feeding-stuffs and foodstuffs with polychlorinated dibenzo-p-dioxins, polychlorinated dibenzofurans (PCDD/Fs), dioxin-like polychlorinated biphenyls (DL-PCBs) and mercury from the River Elbe in the light of the flood event in August 2002. Science Total Environ 364:96-112

[50] Stachel B, Christoph EH, Götz R, Herrmann T, Krüger F, Kühn T, Lay J, Löffler J, Päpke O, Reincke H, Schröter-Kermani C, Schwartz R, Steeg E, Stehr D, Uhlig S, Umlauf G (2007): Dioxins and dioxin-like PCBs in different fish from the river Elbe and its tributaries, Germany. J Hazard Mat 148:199-209

[51] Anonym (2009a) Bewirtschaftungsplan nach Artikel 13 der Richtlinie 2000/60/EG für den deutschen Teil der Fluss­gebietsgemeinschaft Elbe, 11. November 2009

[52] Stachel B, Götz R, Mariani G, Umlauf G (2010) Dioxine und PCBs in Feststoffen aus der Elbe, ihren Nebenflüssen und der Nordsee (Längsprofilaufnahme 2008, Kurzfassung), in: Anonym (Bearbeiter: Bergemann, M, Gaumert T) Elbebericht 2008 - Ergebnisse des nationalen Überwachungsprogramms Elbe der Bundesländer über den ökologischen und chemischen Zustand der Elbe nach EG-WRRL. Sonderkapitel 5.4, S. 64-70. FGG Elbe, Mai 2010, Magdeburg

[53] Anonym (2009b) Hintergrundpapier zur Ableitung der überregionalen Bewirtschaftungsziele für die Oberflächen­gewässer im deutschen Teil der Flussgebieteseinheit Elbe für den Belastungsschwerpunkt Schadstoffe. FGG Elbe, 02.04.2009, Magdeburg. <http://www.elsa-elbe.de/assets/download/FGG-Elbe-Hintergrundpapier-Schadstoffe-2009.pdf>

[54] Peschel H (2009) Regionale Schwerpunkte der Maßnahmenplanung in Sachsen-Anhalt. Informationsveranstaltung zur Bewirtschaftungsplanung im deutschen Einzugsgebiet der Elbe. 23.04.2009. [http://www.elsa-elbe.de/assets/download­/FGG-Elbe-Hintergrundpapier-Schadstoffe-2009.pdf](http://www.elsa-elbe.de/assets/download/FGG-Elbe-Hintergrundpapier-Schadstoffe-2009.pdf)

[55] Götz R, Bauer OH, Friesel P, Herrmann T, Jantzen E, Kutzke M, Lauer R, Paepke O, Roch K, Rohweder U, Schwartz R, Sievers S, Stachel B (2007): Vertical profile of PCDD/Fs, dioxin-like PCBs, other PCBs, PAHs, chlorobenzenes, DDX, HCHs, organotin compounds and chlorinated ethers in dated sediment/soil cores from flood-plains of the river Elbe, Germany. Chemosphere 67:592-603

[56] Umlauf G, Stachel B, Mariani G, Götz R (2011) Dioxins and PCBs in solid matter from the River Elbe, its tributaries and the North Sea (longitudinal profile, 2008). EUR – Scientific and Technical Research series – ISSN 1018-5593 (print), 2011, 118 p.

[57] Stachel B, Mariani G, Umlauf G, Götz R (2011) Dioxine und PCBs in Feststoffen aus der Elbe, ihren Nebenflüssen und der Nordsee (Längsprofilaufnahme 2008) mit Unterstützung der Flussgebietsgemeinschaft Elbe, Sept. 2011. FGG Elbe, Redaktion: Frank Krüger

[58] Anonym (2014b) Sedimentmanagementkonzept der FGG Elbe. Vorschläge für eine gute Sedimentmanagementpraxis im Elbegebiet zur Erreichung überregionaler Handlungsziele. Flussgebietsgemeinschaft Elbe. 25.11.2013. 383 S. [http://www.fgg-elbe.de/tl_files/Downloads/EG_WRRL/hgi/hgd_bp2/sedimentmanagementkonzept_fgg_­final.­pdf](http://www.fgg-elbe.de/tl_files/Downloads/EG_WRRL/hgi/hgd_bp2/sedimentmanagementkonzept_fgg_final.pdf)

[59] Anonym (2014c) Sedimentmanagementkonzept der IKSE. Vorschläge für eine gute Sedimentmanagementpraxis im Elbegebiet zur Erreichung überregionaler Handlungsziele. Int Kommission Schutz der Elbe. Magdeburg, 202 S. [http://­www.ikse-mkol.org/­file­admin/download/Abschlussbericht-Sediment/IKSE_Abschluss­bericht­%­20Sedi­ment_­web.­pdf](http://www.ikse-mkol.org/fileadmin/download/Abschlussbericht-Sediment/IKSE_Abschlussbericht%20Sediment_web.pdf)

[60] Maack B (2011) Dioxin-Skandale – Das Überall-Gift. Spiegel-Online vom 05.01.2011. [http://www.spiegel.de/eines­tages/­dioxin-skandale-a-946974.html](http://www.spiegel.de/einestages/dioxin-skandale-a-946974.html)

[61] Anonym (1983) Blutender Berg – jahrelang verharmlosten Hamburger Behörden die Umweltgefahren, die vom höch­sten Müllberg der Nation ausgehen. Der Spiegel 51/83, 26-25. <http://www.spiegel.de/spiegel/print/d-13855446.html>

[62] Wolf K (1986) The Hamburg-Georgswerder dumping ground – situation, problems and administrative arrangements for producing a rehabilitation plan. In: Assink JW, Van den Brink WJ (eds) Contaminated Soil‚ pp 723-728. Martinus Nijhoff Dordrecht

[63] Anonym (2010c) 25 Jahre Sanierung der Deponie Georgswerder – Umgang mit baulich gesicherten Altlasten: Erkennt­nisse und Perspektiven. Fachtagung vom 10./11. Juni 2010, Behörde für Stadtentwicklung und Umwelt, Amt für Umweltschutz, Abt Bodenschutz/Altlasten, Hamburg.184 S. [https://www.hamburg.de/contentblob/­2356970/data/­tagungsband-25-jahre-dgw.pdf](https://www.hamburg.de/contentblob/2356970/data/tagungsband-25-jahre-dgw.pdf)

[64] Förstner U (1991) Sanierung von Deponien am Beispiel der Deponie Georgswerder. In: Albach H, Schade D, Sinn H (Hrsg) Technikfolgenforschung und Technikfolgenabschätzung. S. 295-319. Springer Berlin

[65] Anonym (1986) Leitbild für die schweizerische Abfallwirtschaft. Schriftenreihe Umweltschutz Nr. 51. Eidgenössische Kommission für Abfallwirtschaft. Bundesamt für Umweltschutz, Bern

[66] Förstner U (2003) Endlagerqualität, Verwertung und Nachhaltigkeit. In: Förstner U, Grathwohl P (Hrsg) Ingenieurgeo­chemie. S. 35-42. Springer-Verlag Berlin

[67] Anonym (2014d) Ehemaliges Werk der Fa. Boehringer Ingelheim. Behörde für Stadtentwicklung und Umweltschutz Hamburg. <https://www.hamburg.de/contentblob/142908/data/boehringer.pdf>

[68] Anonym (1983) Grube ohne Grenze. Jahrelang schickten westeuropäische Firmen und Städte Sondermüll auf eine grenznahe DDR-Kippe. Das Gift droht nun auf dem Wasserweg zurück-zukommen. Der Spiegel 29/1983 <http://www.spiegel.de/spiegel/print/d-14021294.html>

[69] Anonym (1994a) Der Müll und die Moneten. Während das Land Mecklenburg-Vorpommern für Altlasten zahlt, streichen Privatfirmen die Gewinne ein; jetzt durchleuchten Staatsanwälte das Geflecht. Der Spiegel v. 21.02.1994 <http://www.spiegel.de/spiegel/print/d-13684593.html>

[70] Anonym (1993b) Ein Sieb, ein Loch – ein neues Gutachten zur Skandaldeponie Schönberg setzt Schwerins christ­demo­kratische Umweltministerin Petra Uhlmann unter Druck. Der Spiegel 12/1993, 30-31. [http://www.spiegel.­de/­spiegel/print/d-13855446.html](http://www.spiegel.de/spiegel/print/d-13855446.html)

[71] Anonym (2014c) Wer karrt denn Kochsalz durch halb Europa? Berliner Zeitung v. 04.01.2014 <http://www.berliner-zeitung.de/archiv/der-verdacht--dass-tonnenweise-gift-auf-der-deponie-schoenberg-gelagert-wurde-laesst-sich-nicht-entkraeften-wer-karrt-denn-kochsalz-durch-halb-europa-,10810590,8798802.html> 04.01.1994

[72] Anonym (2014d) 1979: Der Ihlenberg wird zur Deponie. NDR, 30.01.2014. [http://www.ndr.-de/kultur/geschichte/­schauplaetze/1979-Der-Ihlenberg-wird-zur-Deponie,ihlenberg143.html](http://www.ndr.-de/kultur/geschichte/schauplaetze/1979-Der-Ihlenberg-wird-zur-Deponie,ihlenberg143.html)

[73] Biener E, Sasse T, Wemhoff T, Jacobsen N (2014) . Erfahrungen bei der Umsetzung des Konzeptes „Deponie auf Deponie“. 30. Fachtagung „Die sichere Deponie – Geokunststoffe im Umweltschutz“. [http://www.akgws.de/sites/­default/files/h_jacobsen_biener_sasse_wemhoff_gws-2014.pdf](http://www.akgws.de/sites/default/files/h_jacobsen_biener_sasse_wemhoff_gws-2014.pdf)

[74] Scharff H (2010) Die nachhaltige Deponie – Strategien zur Stilllegung und Nachsorge in den Niederlanden. Deponie­technik 35, 13 Seiten (Hamburg, Februar 2010). Verlag Abfall aktuell

[75] Buchwald H (1994) Bitterfeld – ein Park voller Müll. Der Vorzeige-Chemiestandort im Dilemma. Firmen entsorgen illegal. Abwässer sind vergiftet. FOCUS Magazin, Nr. 35 v. 29.08.194

[76] Großmann J, Poetke D, Nitschke F, Dommaschk M, Drangmeister J, Thear W, Hirsch D (2007) Ergebnisbericht zum Grund- und Oberflächenwassermonitoring im Großraum Bitterfeld/Wolfen. Berichtszeitraum 2005/2006. Ökologisches Großprojekt Bitterfeld/Wolfen, im Auftrag der Landesanstalt für Altlastenfreistellung Sachsen-Anhalt, 31.8.2007, 71 S

[77] Poetke D, Nitschke F, Hirsch D. (2007) Belastungen in Schachtgraben und Spittelwasser sowie in Mulde und Elbe. Sach­standsbericht zu den Untersuchungen des Jahres 2007 am RRB D02. Stand Oktober 2007. Ökologisches Großpro­jekt Bitterfeld/Wolfen., im Auftrag der Landesanstalt für Altlastenfreistellung Sachsen-Anhalt, 30.10.2007, 18 Seiten, GICON Dresden (unveröffentlicht).

[78] Anonym (2008a) Analyse und Bewertung der technischen Möglichkeiten für eine Problemlösung im Bereich der Oberflächenwässer des Grundwasserkörpers in Bitterfeld. Erstellt für Großmann Ingenieur Consult GmbH (GICON), 19. Juni 2008, 50 Seiten, WISUTEC Chemnitz (unveröffentlicht)

[79] Anonym (2008b) Gewässerunterhaltungsmaßnahme Wiederherstellung des Abflussprofils des Schachtgrabens. Abschlussdokumentation. Erstellt für P-D ChemiePark Bitterfeld Wolfen GmbH/Landesanstalt für Altlastenfreistellung Sachsen-Anhalt, 05. November 2008, 27 Seiten, WISUTEC Chemnitz (unveröffentlicht)

[80] Förstner U, Salomons W (2010) Sediment research, management and policy. J. Soils Sediments 10:1440-1452

[81] Keil M, Großmann J, Weiß H (2011) Comment on „Sediment research, management and policy – a decade of JSS. J Soils Sediments 11:542

[82] Möbius J (2002) Einrichtung und Überwachung eines Testfelds zur Abdeckung kontaminierten Baggerguts mit aktiven Barriere-Systemen, Sportboothafen Hitzacker. Antrag auf Förderung einer Forschungs- und Entwicklungsmaßnahme. Fa. Josef Möbius Bau-Aktiengesellschaft Hamburg (mit Arbeitsbereich Umweltschutztechnik der TUHH), 41 Seiten, Hamburg 2002

[83] Büttner O, Böhme M, Rode M (2009) Teilprojekt 3: Hydraulische Modellierung. Abschn 3.8 Überflutung des Sale­gaster Forsts durch das Hochwasser der Mulde. In: v Tümpling W, Rode M, Böhme M, Gläßer C, Matthies M, Schanze J (Hrsg) SARISK – Entwicklung eines Schadstoffausbreitungsmodells zur stoffbezogenen Risikoanalyse und –bewer­tung extremer Hochwasserereignisse am Beispiel des Landkreises und der Stadt Bitterfeld. Endbericht des Verbund­projektes. BMBF-Förderkennzeichen PTJ 0330690 A-D. Seiten 49-80.

[84] Lersner H v (1985) Rechtliche Instrumente der Umweltpolitik. In: Jänicke M, Simonis UE, Weigmann G (Hrsg.) Wissen für die Umwelt. S. 195-214. Walter de Gruyter Verlag Berlin

[85] Bahlburg CH (2005) Hochwasser und andere Katastrophen – Was haben wir gelernt? In: Karl H, Pohl J, Zimmermann H (Hrsg) Risiken in Umwelt und Technik – Vorsorge durch Raumplanung. Akademie für Raumforschung und Landes­planung. Forschungs- und Sitzungsberichte, Band 223, S. 3-14. Hannover (ISBN 3-88838-052-9)

[86] Grünewald U (2005) Vorsorge gegenüber Naturrisiken: Nach den Augustfluten 2002 in Mittel- und Zentraleuropa – Hochwasservorsorge in Deutschland. In: ibid. Band 233, 78-85

[87] Rulkens WH (2001) An overview of soil and sediment treatment research in the Netherlands. In Stegmann R. et al. (eds) Treatment of Contaminated Soil: Fundamentals, Analysis, Applications, pp. 21-34, Springer-Verlag, Berlin

[88] Anonym (1992b) Development Programme for Treatment Processes for Polluted Aquatic Sediments in the Nether­lands (POSW I). Phase I (1989-1990). PO Box 17. 8200 AA Lelystad, The Netherlands.

[89] Anonym (1997b) Development Programme for Treatment Processes for Contaminated Sediments (Phase II). Final Report nr 97.051. ISBN 90 369 50 97 X. PO Box 17. 8200 AA Lelystad, The Netherlands.

[90] Grotenhuis T, Smit M, Malina G, Kasperek R, Szdzuj J, Satijn B, Joziasse J (2005) Management scenarios for conta­minated sediments at megasites. In Uhlmann O, Annokkée GJ, Arendt F (Eds) ConSoil 2005, Proc. 9th Int FZK/TNO Conf on Soil-Water Systems, Bordeaux 3-7 Oct 2005, Theme E, pp. 2513-2522 ()

[91] Großmann J, Sievers J, Ritter A, Keil M, Weiß H (2005) The Bitterfeld Megasite in Germany – an example for a risk-based management approach. In: Uhlmann O, Annokkée GJ, Arendt F (eds) ConSoil 2005 – Proc 9th International FZK/TNO Conference on Soil-Water Systems, Bordeaux 3–7 October 2005, Theme F, pp 2620–2627

[92] Anonym (2009c) Kz 05.022.0014. Ausschreibungsanzeiger Sachsen-Anhalt vom 29. Mai 2009, S. 37/38 Wettbewerb­licher Dialog „Frachtreduzierung überwiegend schwebstoffgebundener Schadstoffe der im Grundwasserkörper VM 2-4 gelegenen Fließgewässer mit dem Schwerpunkt Spittelwasser/Schachtgraben“. Pilotprojekt Bitterfeld-Wolfen zur Um­setzung der WRRL im Land Sachsen-Anhalt

[93] Förstner U (2008) Maßnahmen. Kapitel 6 in: S. Heise et al.: Bewertung von Risiken durch fest-stoffgebundene Schad­stoffe im Elbeeinzugsgebiet, S. 247-341. Studie im Auftrag der Hamburg Port Authority, erstellt von BIS/TuTech, Ham­­burg 2008

[94] Bley S, Gerth J, Neumann-Hensel H, Ruttkowski V (2006) Methoden der Erfassung, Bewertung und Prognose der intrinsisch/zeitlich verstärkten Schadstoffrückhaltung in kontaminierten Sedimenten (KORA, TV 6.1). Schlussbericht des gleichnamigen Projekts an der TU Hamburg-Harburg und Fa. Fintelmann & Meyer, Hamburg. Förderkennzeichen: 0330519. Laufzeit des Vorhabens: 01.10.2003-31.12.2006

[95] Jacobs P (2013) Frachtreduktion Spittelwasser – Machbarkeitsstudie im Auftrag der Landesanstalt für Altlasten­freis­tellung Sachsen-Anhalt. Folienserie anl. Workshop „Sedimentmanagement der FGG Elbe“, Hamburg, 17. Dezember 2013. Tauw GmbH NL Berlin

[96] Vellinga T (2004) From dredged material management to sediment management. J Soils Sediments 4(4)215

[97] Salomons W, Brils J (Eds, 2004) Contaminated sediments in European River Basins. European Sediment Research Network SedNet, EC Contract No. EVKI-CT-2001-0002. Key Action 1.4.1 Abatement of water pollution from con­taminated land, landfills and sediments. TNO Den Helder/The Netherlands, 80 p.

[98] Heininger P (2014) Schadstoffbelastung aus dem Oberstrom. Forum Strombau und Sedimentmanagement Tideelbe, Hamburg, 4. April 2014. 48 S. <http://www.dialogforum-tideelbe.de/wp-content/uploads/2014/04/FOSUST-3_Sitzung-Anlage-2-TOP-4-P_Heininger-Schadstoffbelastung-Oberstrom.pdf>

[99] Netzband A et al. (2007) Sediment management: an essential element of River Basin Management Plans. J Soils Sediments 7(2)117-132

[100] Anonym (2009e) Auftaktveranstaltung zum „Sedimentmanagementkonzept Sachsen-Anhalt“, der Schlüsselregion im IKSE-Konzept (am 12./13. Mai 2009 in Dresden beschlossen die Delegationsleiter der Intern. Kommission zum Schutz der Elbe die Gründung einer IKSE-Expertengruppe „Sediment-Management“) am 25.09.2009 in Magdeburg; Vortrag U. Hursie, Referat 27, MLU Sachsen-Anhalt, 22 Folien, 2.3 MB

[101] Krüger F, Scholz M, Kreibich M, Baborowski M (2014) Sedimentrückhalt in den Elbauen. Studie zur Erarbeitung des Sedimentrückhaltes in Auen als Teilfunktion des Sedimenttransportgeschehens an der Elbe. Abschlussbericht im Rah­men von „Schad­stoffsanierung Elbesedimente – ELSA“, Behörde für Stadtentwicklung und Umwelt in Hamburg, 73 S. <http://www.elsa-elbe.de/assets/download/fachstudien/Fachstudie-Elbauen.pdf>

[102] Hillebrand G, Claus E (2013) Untersuchung belasteter Sedimente in Buhnenfeldern der Elbe und Staustufen der Saale. Abschlussworkshop. [http://www.elsa-elbe.de/assets/download/abschluss-workshop_12_2013/5_Hillebrand­_Elbebuhnenfelder_Saalestaustufen_2.pdf](http://www.elsa-elbe.de/assets/download/abschluss-workshop_12_2013/5_Hillebrand_Elbebuhnenfelder_Saalestaustufen_2.pdf)

[103] Heise S (2013) Durchführung einer Sondierungsuntersuchung zum Risiko durch eine Schadstoffremobilisierung aus Seitenstrukturen der Elbe. Abschlussbericht. Hochschule für Angewandte Wissenschaften Hamburg. 42 S. <http://elsa-elbe.de/assets/download/fach-studien/Fachstudie_Seitenstrukturen_ELSA_Heise.pdf>

[104] Junge FW (2013) Schadstoffsenke Muldestausee – Aktuelles Potenzial und jüngste Entwicklung seit 2002. Abschluss­bericht Fa. Junge-Erdwissen i.R. des Projekts ELSA der Freien und Hansestadt Hamburg, Taucha, den 06.11.2013. 95 S. [http://elsa-elbe.de/assets/download/fachstudien/Fach­studie_Muldestausee.pdf](http://elsa-elbe.de/assets/download/fachstudien/Fachstudie_Muldestausee.pdf)

[105] Kasimir P, Claus E (2013) Bestandsaufnahme belasteter Altsedimente in der Saale. Vom Wasser 111(4)145-146

[106] Anonym (2012) Bestandsaufnahme belasteter Altsedimente in ausgewählten Gewässern Sachsen-Anhalts. Phase II. Sedimentbeprobung und Sedimentuntersuchungen. G.E.O.S. Ingenieurgesellschaft mbH, im Auftrag von: Landes­betrieb für Hochwasserschutz und Wasserwirtschaft Sachsen-Anhalt; 30.11.2012; 252 S. [http://www.lhw.sachsen-anhalt.de/file-adminBibliothek/Politik_und_Verwaltung/Landesbetriebe/LHW/neu_PDF/5.1/-Dokumen­te­_GLD/Sedi-mentmanagement/Fachgutachten_GEOS/TEIL_II_Bericht_Sedimentuntersuchung.pdf](http://www.lhw.sachsen-anhalt.de/file-adminBibliothek/Politik_und_Verwaltung/Landesbetriebe/LHW/neu_PDF/5.1/-Dokumente_GLD/Sedi-mentmanagement/Fachgutachten_GEOS/TEIL_II_Bericht_Sedimentuntersuchung.pdf)

[107] Wieprecht S et al. (2013) Ermittlung des Remobilisierungspotentials belasteter Altsedimente in ausgewählten Gewäs­sern Sachsen-Anhalts. Institut für Wasser- und Umweltsystemmodellierung (IWS). Lehrstuhl für Wasserbau und Wassermengenwirtschaft der Universität Stuttgart, im Auftrag von: Landesbetrieb für Hochwasserschutz und Wasser­wirtschaft Sachsen-Anhalt; Stuttgart, den 11.06.2013; 153 S. [http://www.lhw.sachsen-Anhalt.de/fileadmin/­Biblio­thek/-politik_und_Verwaltung/Landesbetriebe/LHW/neu_PDF/5.1/Dokumente_GLD/Sediment-management/Fach­gut­achten_IWS/Bericht_Remobilisierungspotential_05-2013.pdf](http://www.lhw.sachsen-Anhalt.de/fileadmin/Bibliothek/-politik_und_Verwaltung/Landesbetriebe/LHW/neu_PDF/5.1/Dokumente_GLD/Sediment-management/Fachgutachten_IWS/Bericht_Remobilisierungspotential_05-2013.pdf)

[108] Anonym (2014e) Entwurf der Aktualisierung des Bewirtschaftungsplans nach § 83 WHG bzw. Artikel 13 der Richt­linie 2000/60/EF für den deutschen Teil der Flussgebietseinheit Elbe für den Zeitraum von 2016 bis 2021. Fluss­gebiets­gemeinschaft Elbe. Magdeburg, 22.12.2014, 238 S.

[109] Werther J, Dreuscher H, Hilligardt R (1984) Aufstromklassierung und maschinelle Entwässerung des Hamburger Hafenschlicks. Veröff. Fachseminar Baggergut, Strom- und Hafenbau, Wirtschaftbehörde der Freien und Hansestadt Hamburg, 27.02.-01.03.1984. S. 183-202

[110] Förstner U (2013) Von der Greiferprobe bis zur In-situ Sanierung – 50 Jahre Forschung an kontaminierten Gewässer­sedimenten. Vom Wasser 111(4) 132-140

[111] Förstner U, Apitz S (2007) Sediment remediation: U.S. focus on capping and monitored natural attenuation – 4th International Battelle Conference on Remediation of Contaminated Sediments. J Soils Sediments 7 (6): 351-358

[112] Jafvert CT, Rogers JE (1991) Biological remediation of contaminated sediments with special emphasis on the Great Lakes : report of a workshop, Manitowoc, Wisconsin, July 17-19, 1990, Environmental Research Laboratory, Office of Research and Development, U.S. Environmental Protection Agency, 181 p, Athens, Ga. [

[113] Anonymous (1997d) Contaminated Sediments in Ports and Waterways: Cleanup Strategies and Technologies: Com­mit­tee on Contaminated Marine Sediments, Marine Board, Commission on Engineering and Technical Systems. Natio­nal Research Council. National Academy Press. 295 p, Washington D.C.

[114] Anonymous (1997e) National Conference on Management and Treatment of Contaminated Sediments, Proceeedings Cincinnati, OH May 13-14, 1997. Technology Transfer and Support Division. National Risk Management Research Laboratory, Office of Research and Development, U.S. Environmental Protection Agency, EPA/625/R-98/001, August 1998, 155 p, Cincinnati, OH

[115] Palermo MR (2001) A state of the art overview of contaminated sediment remediation in the United States. In: International Conference on Remediation of Contaminated Sediments, 10-12 October 2001, Venice, Italy. 10 p. [http://www.envirotools.msu.edu/factsheets/­remediationofcs/­VeniceConference-OverviewofSedRemUS-wCR.PDF](http://www.envirotools.msu.edu/factsheets/remediationofcs/VeniceConference-OverviewofSedRemUS-wCR.PDF)

[116] Anonym (2005d) Contaminated Sediment Remediation Guidance for Hazardous Waste Sites. Kapitel 5 “Monitored Natural Recovery” (4-1 bis 4-11). Weitere Superfund-Technologien für Sedimente: „In-Situ Capping“(5-1 bis 5-14) und „Dredging & Excavation“(6-1 bis 6-36). US Environmental Protection Agency. EPA-540-R-05-012. Dec 2005

[117] Anonym (2007b) Sediment Dredging at Superfund Megasites: Assessing the Effectiveness. Committee on Sediment Dredging at Superfund Megasites. Board on Environmental Studies and Toxicology, Division on Earth and Life Stu­dies, National Research Council oft he National Academies. National Academy Press. 294 p, Washington, D.C.

[118] Anonym (2013b) Sediment Remedy Effectiveness and Recontamination: Selected Case Studies. Association of State and Territorial Solid Waste Management Officials ASTSWMO Sediment Focus Group, 85 pp. [http://www.astswmo­.­org­/­Files/Policies_and_Publications/CERCLA_and­_Brownfields/2013-04-Sediment_Remedy_Effectiveness_and_­Re­contamination.pdf](http://www.astswmo.org/Files/Policies_and_Publications/CERCLA_and_Brownfields/2013-04-Sediment_Remedy_Effectiveness_and_Recontamination.pdf)

[119] Anonym (2013c) Use of Amendments for In Situ Remediation at Superfund Sediment Sites. United States Environ­mental Protection Agency, Office of Superfund Remediation and Technology Innovation. OSWER Directive 9200.2-128FS, 61 p. EPA April 2013

[120] Anonym (2004b) Case Studies of Environmental Dredging Projects. Vol. 5: Appendix in: Engineering Performance Standards Hudson River PCBs Superfund Site. 146 p. Prepared for U.S. Army Corps of Engineers, Kansas City District, USACE Contract No. DACW41-02-D-0003, on behalf of U.S. Environmental Protection Agency, Region 2. Prepared by Malcolm Pirnie, Inc. 104 Corporate Park Drive, White Plains, New York 10602 and TAMS Consultants, Inc. an Earth Tech Company 300 Broadacres Drive Bloomfield, New Jersey 07003, April 2004. [http://www.clu-in.org/­download/contaminantfocus/sediments/huson-river-pcb-case-studies-FP5001.pdf](http://www.clu-in.org/download/contaminantfocus/sediments/huson-river-pcb-case-studies-FP5001.pdf)

[121] Stevens M (2011) Why is the water quality in the River Doe Lea so poor? Dissertation for the Degree of Master of Science, University of Sheffield, Department of Civil & Structural Engineering, Septmber 2011, 132 p. Including Appendix C: River sediment monitoring and dioxin analysis in the Doe Lea: A literature survey of Environment Agency studies between 1991 and 1998

[122] Edwards AMC (2001) River nd estuary management issues in the Humber catchment. In: Huntley D, Leeks G, Waling D (eds) Land-Ocean Interaction: Measuring and Modelling Fluxes from River Basins to Coastal Seas; pp 8-31. IWA Publishing, London

[123] Anonym (1994b) Distribution of PCDDs and PCDFs in surface freshwater systems. AEA Technology, Nationl River Authority, R&D Note 242, Bristol, UK

[124] Anonym (1995) River Doe Lea restoration study. National River Authority. Ove Arup & Partners: Leeds, UK.

[125] Anonym (1998) Contaminated sediments in the Sheffield and South Yorkshire Navigation (SSYN): Risk assessment of options. Scott Wilson Ltd. British Waterways, Gloucester, UK.

[126] Lake I, Foxll C, Lovett A, Fernandes A, Dowding A, White S, Rose M (2005) Effects of river flooding on PCDD/F and PCB levels in cow’s milk, soil, and grass. Environ Sci Technol 39(23) 9033-9038

[127] Lexartza-Artza I, Lerner D, Tkachenko N (2009) Preliminary study of the Doe Lea catchment for the Doe Lea Pro­ject. Catchment Science Centre, The University of Sheffield, August 2009, 48 p

[128] Verta M, Kiviranta H, Salo S, Malve O, Korhonen M, Verkasalo PK, Ruokojärvi P, Rossi E, Hanski A, Päätalo K, Vartiainen T. (2009) A decision framework for possible remediation of contaminated sediments in the River Kymijoki, Finland. Environ Sci Pollut Res Int. 16(1):95-105

[129] Weber R, Gaus C, Tysklind M, Johnston P, Forter M, Hollert H, Heinisch E, Holoubek I, Lloyd-Smith M, Masunaga S, Mocarelli P, Santillo D, Seike N, Symons R, Torres JPM, Verta M, Varbelow G, Vijgen J, Watson A, Costner P, Woelz P, Wycisk P, Zenneg M (2008) Dioxin- and POP-contaminated sites—contemporary and future relevance and challenges. Environ Sci Pollut Res 15:363–393

[130] Anonym (2014f) Hudson River PCBs Summer 2014. (i) Where Are We [131], (ii) Environmental Dredging [132], (iii) Summary of Presentations [133], and many other reports 2006-2014. Hudson River Sloop Clearwater. [http://www­.clearwater.org/ea/pcb-contamination/](http://www.clearwater.org/ea/pcb-contamination/)

[131] Jones AM (2014) Superfund cleanup in the Hudson River: Where are we? An update through the 2014 season. Fo­­lien­serie, 9 Folien. Hudson River PCB Forum, Marist College, Cornell Boathouse, November 11, 2014 [http://www.­clear­water.org/wp-content/uploads/2009/09/2014-11-11-HR-Superfund-Project-Update1.pdf](http://www.clearwater.org/wp-content/uploads/2009/09/2014-11-11-HR-Superfund-Project-Update1.pdf)

[132] Anonym (2014g) Hudson River PCB Information: Environmental Dredging. Hudson River Sloop Clearwater, News & Bulletins. <http://www.clearwater.org/news/dredging.html>

[133] Anonym (2014h) Summary of Presentations. 2014 Hudson River PCB Forum, Marist College Boathouse, November 11, 2014. [http://www.clearwater.org/wp-content/uploads/2009/09/­Summary-of-PCB-Forum-Presentations-11.11.14.pdf](http://www.clearwater.org/wp-content/uploads/2009/09/Summary-of-PCB-Forum-Presentations-11.11.14.pdf)

[134] Garvey EA, Atmadja J, Accardi-Dey AM (2007) Dioxins in the Newark Bay Complex – Newark, New Jersey; in: Foote EA, Durell GS (eds) Remediation of Contaminated Sediments 2007. Paper A-027, 31 p. Proc. 4th Intern Conf Remediation of Contaminated Sediments, Savannah, Ga., January 2007. ISBN 978-1-57477-159-8. Published by Battelle Press, Columbus , OH

[135] Lowe S, Abood K, Ko J, Wakeman T (2005) A sediment budget analysis of Newark Bay. The Technical Proceedings oft he IMarEstT, Part C3. J Mar Sci Environ, August 2005. In [134]

[136] Anonym (2014i) The Passaic River’s Polluted Past, May 2014. U.S. EPA, Region 2, 4 p [http://www.epa.gov/­region­­2­/passaicriver/pdf/pollution_problems_overview_fact_sheet__english_may_2014.pdf](http://www.epa.gov/region2/passaicriver/pdf/pollution_problems_overview_fact_sheet__english_may_2014.pdf)

[137] Bopp RF, Gross ML, Tong H, Simpson HJ, Monson SJ, Deck BL, Moster FC (1991) A major incident of dioxin con­tamination: Sediments of New Jersey estuaries. Environ Sci Technol 25(5): 951-956

[138] Anonym (2014j) Lower Passaic River Restoration Project. U.S. Environmental Protection Agency, Region 2. [http://­www.epa.gov/region2/passaicriver/](http://www.epa.gov/region2/passaicriver/)

[139] Anonym (2014k) An Overview of EPA’s Cleanup Plan, May 2014. U.S. EPA, Section 2. [http://www.epa.gov/region­2/­passaicriver/pdf/prap_overview_fact_sheet_english_june_2014.pdf](http://www.epa.gov/region2/passaicriver/pdf/prap_overview_fact_sheet_english_june_2014.pdf)

[140] Anonym (2014l) An Overview oft he Options for Cleaning up Contaminated Sediment. [http://www.epa.gov/region2/­passaicriver/pdf/cleanup_options_overview_fact_sheet_english_may_2014.pdf](http://www.epa.gov/region2/passaicriver/pdf/cleanup_options_overview_fact_sheet_english_may_2014.pdf)

[141] Shea D (1988) Developing national sediment quality criteria. Environ Sci Technol 22: 11256-1261

[142] Förstner U (2003) Geochemical techniques on contaminated sediments – river basin view. Environ Sci Pollut Res 10 (1):58-68

[143] Anonym (2005e) Die Elbe und ihr Einzugsgebiet – ein geographisch-hydrologischer und wasserwirtschaftlicher Über­blick. Internationale Kommission zum Schutz der Elbe. Magdeburg 258 S.

[144] Anonym (2006) Information zur Sanierung der Altlasten des Braunkohlebergbaus in den neuen Ländern. Bundes­ministerium für Umwelt, Naturschutz und Reaktorsicherheit. Bericht, 8 Seiten

[145] Grünewald U, Uhlmann W (2004) Zur Entwicklung der Wasserbeschaffenheit in den Lausitzer Tagebauseen – Aus­gangspunkt, Stand und Perspektiven. World of Mining – Surface & Underground 56(2):115-125

[146] Grünewald U (2001) Sanierung des regionalen Wasser- und Stoffhaushaltes. Erarbeitung von Prognosen zur Ent­wick­lung der Wassermenge und –beschaffenheit, Ableitung von Risiken und Handlungsbedarf. In: GEOAgentur Berlin Brandenburg (Hrsg.) in Kooperation mit Forschungs-zentrum Bergbaufolgelandschaften der BTU Cottbus. InfoForum Rekultivierung. Dokumentation 6, Nov. 2001, S. 57-66

[147] Gröschke A, Uhlmann W, Rolland W, Grünewald U (2002) Hydrochemische Entwicklung Lausitzer Tagebauseen während der Flutung am Beispiel des Restloches Gräbendorf. Hydrologie und Wasserbewirtschaftung 46(6):256-267

[148] Zschiedrich K, Benthaus F-C (2005): Bergbausanierung – bergbauliche und wasserwirtschaft­liche Aufgaben. Fach­tagung zur Nachsorge betriebsbedingter Boden- und Grundwasserschäden des Bergbaus nach der endgültigen Betriebs­stilllegung. Proc. DGFZ, ISSN 1430-0176; Heft 27, S. 94-113. Dresden

[149] Luckner L (2006a) Lausitz: Sanierung einer Bergbaufolgelandschaft. Teil 1. Die Lösung des Wassermengen­pro­b­lems­. WWT 3/2006, S. 33-37

[150] Luckner L (2006b) Lausitz: Sanierung einer Bergbaufolgelandschaft. Teil 2. Das Problem mit der Wasserqualität. Die Neutralisation der sauren Restseen. WWT 4/2006, S. 10-16

[151] Klapper H, Friese K, Scharf B, Schimmele M, Schultze M (1998) Ways of controlling acid by ecotech-nology. In: Geller W, Klapper H, Salomons W (Eds.) Acid Mine Lakes – Acid Mine Drainage, Limnology and Reclamation. Chapter 22, pp. 401-416. Springer Verlag Heidelberg

[152] Klapper H (2002) Mining lakes: Generation, loading and water quality control. In: Mudroch A, Stottmeister U, Kennedy C, Klapper H. (Eds.) Remediation of Abandoned Surface Coal Mining Sites. pp. 57-110. Springer Verlag Heidelberg

[153] Reichel F, Uhlmann W (1995) Möglichkeiten und Grenzen der Beeinflussung der Wasser-beschaffenheit in Tagebau­restlöchern bei aufsteigendem Grundwasser am Beispiel der Lausitzer Bergbaufolgelandschaft.- In: Proc 4. Dresdener Grundwasserforschungstage, Coswig, 24./25. Oktober 1995, Band II, S. 39-51

[155] Pedersen TF, McNee JJ, Flather DH, Mueller B, Pelletier CA (1998) Geochemical behaviour of submerged pyrite-rich tailings in Canadian lakes. In: Geller W, Klapper H, Salomons W (Eds.) Acid Mine Lakes – Acid Mine Drainage, Limnology and Reclamation. Chapter 6, pp. 87-125. Springer Heidelberg

[156] Paul M (2003) Geochemische In-situ-Stabilisierung von Bergbaualtlasten. In: Förstner U, Grathwohl P Ingenieur­geochemie – Natürlicher Abbau und Rückhalt, Stabilisierung von Massenabfällen. S. 298-329. Springer Berlin

[157] Zerling L, Müller A, Jendryschik K, Hanisch C, Arnold A (2001) Der Bitterfelder Muldestausee als Schadstoffsenke – Entwicklung der Schwermetallbelastung 1992 bis 1997. Abhandlungen der Sächsischen Akademie der Wissenschaf­ten zu Leipzig 59(4): 69 S.

[158] Czegka W, Hanisch C, Junge F, Zerling L, Baborowski M (2006) Changes in uranium concentration in the Weisse Elster River as a mirror of the remediation in the former WISMUT mining area. In: Merkel BJ, Hasche-Berger A (Eds) Uranium in the Environment. S. 875-884. Springer Berlin

[159] Hammer J (1986) Zur Geochemie ausgewählter Elemente und zu deren Bindungsverhältnissen im Kupferschiefer der Sangerhäuser Mulde. Dissertation, TU-Bergakademie Freiberg

[160] Haack U, Plimer I (1998) Zum Stoffbestand der Kupferschiefer Erzlagerstätten Mansfeld-Hettstedt-Eisleben. Mitt Geol Sachsen-Anhalt 4: 153-162

[161] Baborowski M, Bozau E (2006) Impact of former mining activities on the uranium distribution in the River Saale (Germany). Appl Geochem 21: 1073-1082

[162] Baborowski M, Mages M, Hiltscher C, Matschullat J, Guhr H (2006) Former mining activities influence uranium con­­centrations om the Elbe river near Magdeburg. In: Merkel BJ, Hasche-Berger A (Hrsg) Uranium in the Environment. S. 585-592. Springer Berlin

[163] Schreck P, Wennrich R, Stärk MJ, Schubert M, Weiß H (2004) Mansfeld – the contribution of a mining-affected catchment area to regional riverine pollution. UFZ-Bericht 18/2004, UFZ Leipzig-Halle GmbH, Leipzig, pp. 169-170

[164] Klemm W, Greif A, Knittel U (2004) AP 3.7: Schwermetall- und Arsenverlagerung in der Freiberger und Zwickauer Mulde. In: Geller W et al. (Hrsg.) Schadstoffbelastung nach dem Elbe-Hoch-wasser 2002. Endbericht des Ad-hoc Ver­bundprojekts, S. 159-172

[165] Broekaert JAC, Siemens V, Krugmann T, Schlenker A (2004) AP 3.8: Schwermetall- und Arsenverlagerung in der Vereinigten Mulde. In: Geller W et al. (Hrsg.) Schadstoffbelastung nach dem Elbe-Hochwasser 2002. Endbericht des Ad-hoc Verbundprojekts, S. 173-182

[166] Jacobs PH (2003) Kontaminierte Sedimente und Baggergut: Aktive Barriere-Systeme für die Subaquatische Lage­rung und Abdeckung. Dissertation an der Technischen Universität Hamburg-Harburg. 3. Dezember 2003. 198 S.

[167] Jacobs PH (2002) A new rechargeable dialysis sampler for monitoring subaqeus in-situ sediment caps. Wat Res 36 (13): 121-3129

[168] Jacobs PH, Förstner U (1999) Concept of subaqueous in-situ capping of contaminated sediments with active barrier systems (ABS) using natural and modified zeolites. Wat Res 33(9):2083-2087.

[169] Jacobs P, Förstner U (2001) Managing contaminated sediments. IV: Subaqueous storage and capping of dredged material. J Soils Sediments 1(4):205-212

[170] Jacobs PH, Förstner U (2003) Gewässersedimente und Baggergut. In: Förstner U, Grathwohl P Ingenieurgeochemie – Natürlicher Abbau und Rückhalt, Stabilisierung von Massenabfällen. S. 330-360. Springer Verlag Berlin

[171] Jacobs PH, Förstner U, Prestel H, Nießner R (2001) On-site Porenwasser-Probenahme und Schwermetall-Analytik mittels Kopplung von Dialyseprobenahme und laserinduzierter Fluoreszensspektroskopie: Feldstudie Vollert-Süd. Wasserchemische Gesellschaft, Jahrestagung 2001 Bad Wildungen, 21.-23.5.2001. S. 455-461. Karlsruhe

[172] Jacobs PH, Waite TD (2003) The role of aqueous iron and manganse in subaqueous active barrier systems containing ntural clinoptilolite. Chemosphere 54(3)313-324

[173] Magar VS, Wenning RJ (2006) The role of natural recovery in sediment remediation. Integrated Envir Assessment and Management 2:66-74

[174] Evison L, Greenberg M, Logan M, Magar V, Nadeau S, Reible D (2007) Definition and demonstration of remedy effectiveness: What worked and what didn’t (Panel Discussion). In: Foote EA, Durell GS (Eds.) Remediation of Contaminated Sediments 2007. Proc 4th Int Conf on Remediation of Contaminated Sediments (Savannah, Georgia, January 2007). Battelle Press, Columbus, OH

[175] Kern U, Westrich B (Bearbeiter, 1999) Methoden zur Erkundung, Untersuchung und Bewertung von Sedimentabla­gerungen und Schwebstoffen in Gewässern. Hrsg. vom Arbeitskreis „Schweb- und Schadstoffe in Fließgewässern“ (Leiter: U. Förstner) des Deutschen Verbands für Wasser­wirtschaft und Kulturbau e.V. (DVWK, jetzt: DWA), DVWK-Schriften Nr. 128, 418 S.

[176] Förstner U, Gerth J, Bley S, Neumann-Hensel H (2007) Methoden der Erfassung, Bewertung und Prognose der intrinsisch/zeitlich verstärkten Schadstoffrückhaltung in kontaminierten Sedimenten (KORA, TV 6). 138 Seiten. Förderkennzeichen 0330519. Laufzeit 01.10.2003 bis 31.12.2006. BMBF, Projektträger Jülich 2007.

[177] Schwartz R, Gerth J, Neumann-Hensel H, Bley S, Förstner U (2006) Assessment of highly polluted fluvisol in the Spittelwasser floodplain, based on national guideline values and MNR-criteria. J Soils Sediments 6 (3): 145-155

[178] Anonym (1994c): AG Boden. Bodenkundliche Kartieranleitung, 4 Aufl., Schweizerbart'sche Verlagsbuchhandlung, Stuttgart, 392 p.

[179] Ghosh U, Luthy R, Cornelissen G, Werner D, Menzie CA (2011) In-situ sorbent amendments: A new direction in contaminated sediment management. Environ Sci Technol 45(4)1163-1168

[180] van Noort PCM, Koelmans AA (2012) Nonequilibrium of organic compounds in sediment-water systems. Conse­quences for risk assessment and remediation measures. Environ. Sci. Technol. 2012, 46, 10900-10908

[181] Anonym (2011a) Common Implementation Strategy for the Water Framework Directive (2000/60/EC: Technical Guidance for Deriving Environmental Quality Standards, Guidance Document No. 27, European Communities, 204 p.

[182] Kupryianchyk D, Rakowska MI, Reible D, Harmsen J, Cornelissen G, van Veggel M, Hale SE, Grotenhuis T, Koel­mans AA (2015) Positioning activated carbon amendment technologies in a novel framework for sediment manage­ment. Integr Environ Assess Manag 11(2)221-234

[183] Patmont C, Ghosh U, LaRosa P, Menzie C, Luthy R, Greenberg M, Cornelissen G, Eek E, Collins J, Hull J, Hjartland T, Glaza E, Bleiler J, Quadrini J (2015) In situ sediment treatment using activated carbon: A demonstrated sediment cleanup technology. Integr Environ Assess Manag 11(2)195-207

[184] Anonymous (2013d) Superfund remedial program review action plan. U.S. Environmental Protection Agency, Wash. D.C., 43 pages. [http://www.epa.gov/superfund/cleanup/pdfs/­Final_SPR_Action_Plan-11_26_2013_%282%29.pdf](http://www.epa.gov/superfund/cleanup/pdfs/Final_SPR_Action_Plan-11_26_2013_%282%29.pdf)

[185] Anonymous (2014m) Contaminated Sediments Remediation. Remedy Selection for Contaminated sediments. August 2014, 525 pages. Prepared by Interstate Technology and Regulatory Council. Contaminated Sediments Team. ITRC Guidance Document. <http://www.itrcweb.org/contseds_remedy-selection/Content/Resources/CSRPDF.pdf>

[186] Cornelissen G*, Amstaetter K, Hauge A, Schaanning M, Beylich B, Gunnarsson J, Breedveld G, Oen A, Eek E (2012) Large-scale field study on thin-layer capping of marine PCDD/F contaminated sediments in Grenlandfjords, Norway: Physicochemical effects. Environ Sci Technol 46:12030–12037. *[Norwegian Geotechnological Institute, Oslo, Norway; **Gerard Cornelissen: 367.22 Total Impact Points*]

[187] Rakowska MI, Kupryianchyk D, Harmsen J, Grotenhuis JTC, Koelmans AA* (2012). In situ remediation of contami­nated sediments using carbonaceous materials. Environ Toxicol Chem 31:693–704. *[Department of Aquatic Ecology and Water Quality, Wageningen, The Nethderlands. **Albert Koelmans: 445.73 Total Impact Points*]

[188] Gilmour CC*, Riedel GS, Riedel G, Kwon S, Lands R, Brown SS, Menzie CA, Ghosh U (2013) Activated cabon mitigates mercury and methyl mercury bioavailability in contaminated sediments. Environ Sci Technol 47(22):13001-13010 *[United States Geological Survey, Reston Va, and Smithsonian Environmental Research Center, Edgewater Md; **Cynthia Gilmore; 190.38 Total Impact Points*]

[189] Choi Y, Cho Y-M, Werner D*, Luthy RG (2014) In situ sequestration of hydrophobic organic contaminants in sedi­ments under stagnant contact with activated carbon. 2 Mass transfer modeling. Environ Sci Technol 48(3)1843-1850 *[School of Civil Engineering and Geosciences, Newcastle University, Newcastle upon Tyne UK; **David Werner: 166.75 Total Impact Points*]

[190] Jahnke A, McLeod M, Wickström H, Mayer Ph* (2014) Equilibrium sampling to determine the thermodynamic poten­tial for bioaccumulation of persistent organic pollutants from sediment. Environ Sci Technol 48(19)11352-11359 *[Department of Environmental Engineering, Technical University of Denmark, Kongens Lyngby, Denmark; **Philipp Mayer: 413.40 Total Impact Points*]

[191] Friedman CL, Lohmann R* (2014) Comparing sediment equlibrium partitioning and passive sampling techniques to estimate benthic biota PCDD/F concentrations in Newark Bay, New Jersey (U.S.A.). Environ Poll 186: 172-179, *[University of Rhode Island Graduate School of Oceanography, Narragansett RI, USA; **Rainer Lohmann: 391.88 Total Impact Points*]

[192] Apell JN, Gschwend PM* (2014) Validating the use of performance reference compounds in passive samplers to assess porewater concentrations in sediment beds. Environ Sci Technol 48(17) 10301-10307 *[Massachusetts Institute of Techno­logy, Cambridge, Mass; **Philip Gschwend: 380.27 Total Impact Points*]

[193] Thompson JM, Hsieh C, Luthy RG* (2015) Modeling uptake of hydrophobic organic contaminants into polyethylene passive sampler. Environ Sci Technol. 49(4):2270-2277: *[Stanford University, Department of Civil and Environmen­tal Engineering, Stanford Ca, USA; **Richard Luthy:* *580.92 Total Impact Points*]

[194] Lampert DJ, Thomas G, Reible DD* (2015) Internal and external transport significance for predicting contaminant uptake rates in passive samplers. Chemosphere 119:910-916. *[Department of Civil and Environmental Engineering, Texas Tech University, Lubbock Tx; **Danny D Reible: 316.88 Total Impact Points*]

[195] Peijnenburg WJGM*, Teasdale PR, Reible D, Mondon J, Bennett WW, Campbell PGC** (2014) Passive sampling methods for contaminated sediments: State of the science for metals. Integr Environ AssessManag 10(2)179-196. *[National Institute for Public Health and the Environment, Utrecht, The Netherlands; **WJGM Peijnenburg: 543.33 Total Impact Points*]; **[Institut nationale de la Recherche Scientifique, Eau Terre Environnement, Québec, Canada; *Peter Campbell, 590.48 Total Impact Points*]

[196] Lydy MJ, Landrum PJ*, Oen AMP, Allinson M, Smedes F, Harwood AD, Li H, Maruya KA, Liu J (2014) Passive sampling methods for contaminated sediments: state of the science for organic contaminants. Integr Environ Assess Manag 10(2)167-178. *[Center for Fisheries, Aquaculture and Aquatic Sciences and Department of Zoology, Southern Illinois University, Carbondale, Illinois USA; **Peter Landrum: 430.14 Total Impact Points*]

[197] Ghosh U*, Driscoll SK, Burgess RM, Jonker MTO, Reible D, Gobas F,Choi Y, Apitz SE, Maruya KA, Gala WR, Mortimer M, Bega C (2014) Passive sampling methods for contaminated sediments: Practical guidance for selection, calibration, and implementation. Integr Environ Assess Manag 10(2)210-223. *[Department of Chemical, Biochemical, and Environmental Engineering, University of Maryland Baltimore County, Baltimore Md, USA; **Upal Ghosh: 204.09 Total Impact Points*]

[198] Salomons W (2005) Sediments in the catchment-coast continuum. J Soils Sediments 5(1):2-8

[199] Schipper C, Schout P (2003) De weg naar implementatie von de Chemie-Toxiciteit-Toets. Werkdocumnt: RIKZ/­2003­.­036. AKWA/RIKZ 04.005, ISBN: 36934761, 82 p.

[200] Babut M, Oen A, Hollert H, Apitz SE, Heise E, White S (2006) Priorization at river basin scale, risk assessment at local scale: suggested approaches. In: Heise S (ed) Sediment Risk Assessmeng and Communication, pp 107-151. Else­vier, Amsterdam

[201] Westrich B (2007) Sustainable sediment management. In: Westrich B, Förstner U (eds) Sediment Dynamic and Pollu­tant Mobility in Rivers. An Interdisciplinary Approach. Chap. 2.1, pp 35-49. Springer. Berlin

[202] Heise S, Förstner U, Westrich B, Jancke T, Karnahl J, Salomons W (eds, 2004) Inventory of Historical Contaminated Sediment in Rhine Basin and its Tributaries. Technical University of Hamburg-Harburg and University of Stuttgart, on behalf of the Port of Rotterdam. October 2004, Hamburg, 225 p.

[203] Schönberger H (2004) HCB in the River Rhine. In:[107] Chapter 4.6, pp 138-145. Technical University of Hamburg-Harburg and University of Stuttgart, on behalf of the Port of Rotterdam. October 2004, Hamburg

[204] Stronkhorst J (2003) Ecotoxicological effects of Dutch harbour sediments. PhD Thesis, Free University Amster­dam

[205] Heise S, Förstner U (2006) Risks from historical contaminated sediments in the Rhine Basin. Water Air Soil Pollut Focus 6:625-636

[206] de Beijer P (2004) Perspective on sediment management in European rivers. Presentation of the Port of Rotterdam at the SedNet Conference, November 25-26, 2004, Venice. <http://sednet.org/download/Stakeholders_de_Beijer_Day2.pdf>

[207] Anonym (2004c) Umstrittene Sedimentspülung am Rhein – Chemie-Altlast von Hochrhein wandert bis zur Nordsee. Neue Zürcher Zeitung 30.12.2004. <http://www.nzz.ch/aktuell/startseite/articleCHNMJ-1.352525>

[208] Anonym ((2005f) Relocation of sediment from Iffezheim weir. Complaint to EU. Amsterdam, 27 April 2005, Lyck­lama, T., representing the NGOs Stichting Reinwater, Stichting De Noordzee, Waddenvereniging, Gelderse Milieu­fede­­ra­tie, Natuur en Milieufederatie Utrecht, Rhein Kolleg: [www.reinwater.nl/docs/Iffezheim.pdf](http://www.reinwater.nl/docs/Iffezheim.pdf)

[209] Anonym (2011b) Rheinschlamm landet diesmal in den Niederlanden. Badisches Tagblatt, Artikel vom 19.02.2011

[210] Anonym (2009d) Sedimentmanagementplan Rhein. Zusammenfassung. Internationale Kommission zum Schutz des Rheins. Bericht Nr. 175. 101 S. <http://www.iksr.org/uploads/media/Bericht_Nr_175d_01.pdf>

[211] Anonym (2008c) Richtlinie 2008/56/EG des Europäischen Parlaments und des Rates vom 17.06.2008 zur Schaf­fung eines Ordnungsrahmens für Maßnahmen der Gemeinschaft im Bereich der Meeresumwelt (Meeresstrategie-Rahmen­richtlinie). Amtsblatt der Europäischen Union vom 25.6.2008. L 164/19

[212] Anonym (2009e/2013) Gemeinsame Übergangsbestimmungen zum Umgang mit Baggergut in den Küstengewässern. August 2009, 39 Seiten. [http://www.htg-baggergut.de/Downloads/Ueber­gangsbestimmungen_Baggergut_Kuesten­gewaesser.pdf](http://www.htg-baggergut.de/Downloads/Uebergangsbestimmungen_Baggergut_Kuestengewaesser.pdf)

[213] Anonym (2008d) Strombau- und Sedimentmanagementkonzept für die Tideelbe. Hamburg Port Authority und Was­ser- und Schifffahrtsverwaltung des Bundes. 39 S., 1. Juni 2008. [http://www.hamburg-port-authority.de/de/­presse/­studien-und-berichte/Documents/SB-SM-Konzept-HPA-WSV.pdf](http://www.hamburg-port-authority.de/de/presse/studien-und-berichte/Documents/SB-SM-Konzept-HPA-WSV.pdf)

[214] Murray LA, Breedveld GD (2011) River engineering and sediment management concept for the tidal Elbe River. Task 2: Evaluaion of handling contaminated sediments from the perspective of ecology and economic efficiency.

[215] Anonym (o.J.) ELSA – Schadstoffsanierung Elbsedimente. Projektinformation. [http://www.elsa-elbe.de/projekt­information.html](http://www.elsa-elbe.de/projektinformation.html)

[216] Medek J et al. (2014) SedBiLa – Bedeutung der Bilina als historische und aktuelle Schadstoffquelle für das Sedimentmanagement im Einzugsgebiet der Elbe. Studie im Auftrag der Freien und Hansestadt Hamburg. 80 S. [http://elsa-elbe.de/assets/download/fachstudien/Fach­studie_Sedbila_DE.pdf](http://elsa-elbe.de/assets/download/fachstudien/Fachstudie_Sedbila_DE.pdf)

[217] De Haar U (1978) Die Arbeit der Senatskommission für Wasserforschung 1957-1977. Senatskommission für Wasserforschung. Mitteilung I. Harald Boldt Verlag, Boppard

[218] Förstner U, Winkler HA (Bearb, 1982) Untersuchung über das Vorkommen und Verhalten von Metalle und Metalloi­den in Oberflächenwässern und Trinkwässern. Kap. I.1.7 Spurenmetalle in Schwebstoffen und Sedimenten, S. 25-32 in: Förstner U, De Haar U, Jüttner F, Müller H, Sonnenborn M, Winkler, HA (Hrsg.) Schadstoffe im Wasser. Metalle – Phenole – Algenbürtige Schadstoffe. DFG, Kommission für Wasserforschung Mitt. IV. Harald Boldt Verlag, Boppard

[219] Hellmann H (1970) Die Charakterisierung von Sedimenten auf Grund ihres Gehaltes an Spurenmetallen. Deutsche Gewässerkundl. Mitt. 14: 160-164

[220] Anonym (1988) Feststofftransport in Fließgewässern - Berechnungsverfahren für die Ingenieurpraxis. DVWK-Schriften Nr. 87, Verlag Paul Parey Hamburg

[221] Anonym (1992c) Studie über die Umlagerung von Sedimenten in Wasserstraßen. DVWK-Materialien, H 2/92. Bonn

[222] Anonym (1993c) Verlandung von Flußstauhaltungen. DVWK-Schriften Nr. 105, Verlag Paul Parey Hamburg

[223] Anonym (1997c) Wasserwirtschaftliche Bedeutung der Festlegung und Freisetzung von Nährstoffen durch Sedimente in Fließgewässern. DVWK-Schriften Nr. 115, Verlag Paul Parey, Hamburg

[224] Westrich B (1988) Hydromechanische Einflussfaktoren auf das Transportverhalten kontaminierter Schwebstoffe in Flüssen. DVWK Mitteilungen, Heft 9. Bonn

[225] Kern U, Westrich B (Hrsg.,1999) Methoden zur Erkundung, Untersuchung und Bewertung von Sedimentumlagerun­gen und Schwebstoffen in Gewässern. DVWK Schriften Nr 128. Hennef

[226] Förstner U , Ackermann F, Alberti J, Calmano W, Frimmel F, Kornatzki KH, Leschber R, Roßknecht H, Schleichert U, Tent L (1987) Allgemeine Problematik und heutiger Stand der Entwicklung von Sedimentqualitäts­kriterien. Z Was­ser- Abwasserforsch 20:54-59

[227] Calmano W (Hrsg, 2001) Untersuchung und Bewertung von Sedimenten. Springer, Berlin

[228] Förstner U, Müller G (1974) Schwermetalle in Flüssen und Seen als Ausdruck der Umweltverschmutzung. 225 S. Springer-Verlag Berlin

[229] Anonym (1973) Schatz auf Grund – Verseuchen giftige Metalle schon das Trinkwasser? Heidelberger Wissenschaft­ler melden alarmierende Befunde über Westdeutschlands Flüsse nach Bonn. Der Spiegel 3/1973, S. 114, 15.1.1973 <http://www.spiegel.de/spiegel/print/d-42713576.html>

[230] Westrich B (1988) Fluvialer Feststofftransport – Auswirkung auf die Morphologie und Bedeutung für die Gewässer­güte. SchrR Wasser-Abwasser Bd 22, 173 S. Oldenbourg Verlag München

[231] Westrich B, Haag I, Kern U (2000) Mobilität von Schadstoffen in den Sedimenten staugeregelter Flüsse – Dynamik und Bilanzierung von Schwebstoffen und Schwermetallen in einer Stauhaltungskette, Forschungsbericht FZKA-BWPLUS. Fzk: PW 96 182. März 2000. [http://www.fachdokumente.lubw.baden-wuerttemberg.de/servlet/is/40070/­PW96189SBer­.pdf?­command=download-Content&filename=PW96189SBer.pdf&FIS=203](http://www.fachdokumente.lubw.baden-wuerttemberg.de/servlet/is/40070/PW96189SBer.pdf?command=download-Content&filename=PW96189SBer.pdf&FIS=203)

[232] Schleichert U (1975) Schwermetallgehalte der Schwebstoffe des Rheins bei Koblenz im Jahresablauf. Dt Gewässerkd Mitt 19:150-157

[233] Hollert H, Dürr M, Erdinger L, Braunbeck T (2000) Cytotoxicity of settling particulate matter (SPM) and sediments oft he Neckar river (Germany) during a winter flood. Environ Toxicol Chem 19:528-534

[234] Calmano W, Förstner U (Hrsg, 1996) Sediments and Toxic Substances – Environmental Effects and Ecotoxicity. Springer-Verlag Berlin

[235] Kausch H, Michaelis W (Hrsg., 1996) Suspended Particulate Matter in Rivers and Estuaries. Advances in Limnology 47, 573 p, Schweizerbart’sche Verlagsbuchhandlung Stuttgart

[236] Stigliani WM (1988) Changes in valued capacities of soils and sediments as indicators of nonlinear and time-delayed environmental effects. Environ Monitoring Assess 10:245-307

[237] Stigliani WM (1991) Chemical time bombs: definition, concepts, and examples. Executive report 16 (CTB basic docu­m­ent). IIASA Laxenburg, Austria, 23 p.

[238] Förstner U (1996) Contaminated sediments and remediation – geochemical perspective. In: Novotny V, Somlyódy L (Eds) Remediation and Management of Degraded River Basins with Emphasis on Central and Eastern Europa. NATO ASI Series, Partnership Sub-Series, 2. Environment, Vol 3, pp 231-253. Springer Berlin

[239] Förstner U (1993) Sediment problems related to chemical time bombs - three conceptual approaches. In: ter Meulen GBR, Stigliani WM, Salomons W, Bridges EM (Eds.) Chemical Time Bombs - European State-of-the-Art Conference on Delayed Effects of Chemicals in Soils and Sediments, pp. 129-146. Foundation for Ecodevelopment "Stichting Mon­dial Alternatief" Hoofdorp/The Netherlands. ISBN 90-71111-62-8.

[240] Salomons W (1995) Long-term strategies for handling contaminated sites and large-scale areas. In: Salomons W, Stigliani WM (Eds) Biogeodynamic of pollutants in soils and sediments – risk assessment of delayed and non-linear responses; pp 1-30. Springer Berlin

[241] Förstner U (1995) Non-linear release of metals from aquatic sediments. In: Salomons W, Stigliani WM (Eds) Biogeo­dynamic of pollutants in soils and sediments – risk assessment of delayed and non-linear responses; pp 247-307. Sprin­ger Berlin

[242] Eijsackers H (1995) How to manage accumulated contaminants.In: Salomons W, Stigliani WM (Eds. Biogeodynamic of pollutants in soils and sediments – risk assessment of delayed and non-linear responses; pp 309-329, Springer Berlin

[243] Stigliani WM (1995) Global perspectives and risk assessment. In: Salomons W, Stigliani WM (Eds. Biogeodynamic of pollutants in soils and sediments – risk assessment of delayed and non-linear responses; pp 331-343, Springer Berlin

[244] Hong J (1995) Characteristics and Mobilization of Heavy Metals in Anoxic Sediments oft he Elbe River during Re­sus­pension/Oxidation. Dissertation an der Technischen Universität Hamburg-Harburg, 157 S.

[245] Anonym (1994c): Die Belastung der Elbe. Teil I - Elbenebenflüsse. Statusberichte der vom Bundesministerium für Bildung, Wissenschaft, Forschung und Technologie geförderten Elbe - Nebenflüsse Verbundvorhaben. Forschungs­zentrum Karlsruhe GmbH, Projekträger Wassertechnologie und Schlammbehandlung (Hrsg.). 170 S.

[246] Furrer R (1998): Ergebnisse des BMBF-Verbundvorhabens: Geogener Background im Elbe-Einzugsgebiet, in: Geller W, Puncochar P, Bornhöft D, Boucek J, Feldmann H, Guhr H, Mohaupt V, Simon M, Smart’ak J, Spoustova J, Uhl­mann O (Hrsg.) Gewässerschutz in Einzugsgebiet der Elbe. 8. Magdeburger Gewässerschutzseminar, S. 78 - 81, B. G. Teubner Verlagsgesellschaft Stuttgart Leipzig

[247] Kluge A (1994) Bestandsaufnahme und Schwermetallbelastung in Wasser und Sediment der Mulde in den Jahren 1991 -1993, in Guhr H, Prange A, Puncochar P, Wilken R -D, Büttner B (Hrsg.) Die Elbe im Spannungsfeld zwischen Ökologie und Ökonomie. 6. Magdeburger Gewässerschutzseminar, S. 78 - 83, B. G. Teubner Verlagsgesellschaft Stutt­gart Leipzig

[248] Müller G, Furrer R (1994) Belastungspotential Elbenebenflüsse. Schwermetalle in den Sedimenten der Elbe und ihrer Zuflüss,. in: Guhr H, Prange A, Puncochar P, Wilken R-D, Büttner B (Hrsg.) Die Elbe im Spannungsfeld zwischen Öko­logie und Ökonomie. 6. Magdeburger Gewässerschutzseminar, S. 69 - 77, B.G. Teubner Verlagsgesellschaft Stutt­gart Leipzig

[249] Rudis M, Trejtnar K (1996) Sedimente in den Staubecken des tschechischen Elbeabschnitts, in: Prange A, Wilken R-D, von Tümpling U, Spoustova J, Punchochar P, Lencova E (Hrsg.) Ökosystem Elbe - Zustand, Entwicklung und Nut­zung. 7. Magdeburger Gewässerschutzseminar, Tagungsband, S. 177 - 182

[250] Einax J, Truckenbrodt D, Matschullat J, Naumann U (1996) Zur Schadstoffbelastung der Saale und der Schwarzen Elster. in: Prange A, Wilken R-D, von Tümpling U, Spoustova J, Punchochar P, Lencova E. (Hrsg.) Ökosystem Elbe - Zustand, Entwicklung und Nutzung. 7. Magdeburger Gewässerschutzseminar, Tagungsband, S. 154 - 158

[251] Heininger P, Pelzer J (1998) Trends und Verteilungsmuster in der Schadstoffbelastung von Sedimenten aus östlichen Bundeswasserstraßen. Acta hydrochim hydrobiol 26(4) 218 - 225

[252] Prange A et al. (1997) Erfassung und Beurteilung der Belastung der Elbe mit Schadstoffen. Abschlußbericht zum BMBF - Forschungsvorhaben: 02-WT 9355/4, Band 1-3, GKSS-Forschungszentrum Geesthacht

[253] Müller A, Hanisch C, Zerling L, Lohse M, Walther A (1998) Schwermetalle im Gewässersystem der Weißen Elster - Natürliche und anthropogene Elementverteilung im Sediment, im Schwebstoff und in der gelösten Phase. Abh Sächs Akad der Wissenschaften zu Leipzig. Mathematisch-naturwissenschaftliche Klasse. Band 58, Heft 6, 199 S., Leipzig

[254] Müller A, Wessels M (1999) The flood in the Odra River 1997 – Impact of suspended solids on water quality. Acta hydrochim hydrobiol 27: 316-320

[255] Müller, G., Yahia, A. (1992) Schadstoffbelastung in Böden von Hochwasserüberflutungsflächen des Rheins. Litera­tur­studie der Universität Heidelberg im Auftrag des Landesamtes für Wasserwirtschaft Rheinland-Pfalz

[256] Miehlich G (1987) Substratgenese und Systematik von Böden der Hamburger Flussmarsch. Mitt Dtsch Bodenkdl Ges 55/II: 801-803

[257] Schuster, J., Miehlich, G. (1989) Tideabhängige Konzentrationsveränderungen in Prielwässern als Ausdruck von Aus­tauschvorgängen zwischen Vordeichsland und Elbeästuar, Mitt Dtsch Bodenkdl Ges 59/I: 483-488

[258] Miehlich G (1994) Auen und Marschen als Senke für belastete Sedimente der Elbe. In: Guhr, H. et al. (Hrsg.) Die Elbe im Spannungsfeld zwischen Ökologie und Ökonomie. S. 307-312. Teubner Verlag Leipzig

[259] Friese K, Witter B, Miehlich G, Rode M (Hrsg. 2000) Stoffhaushalt von Auenökosystemen – Böden und Hydrologie, Schadstoffe, Bewertungen, 438 S. Springer Verlag Berlin

[260] Anonym (2000) Schadstoffdynamik in Einzugsgebieten. Projekt III.B.09 (1999-2002). Sächsische Akademie der Wissenschaften zu Leipzig. Informationen zur Öffentlichen Frühjahrssitzung am 14. April 2000 in Leipzig

[261] Gröngröft, A. (1992) Untersuchung des Sickerwasser- und Stoffeintrags aus Hafenschlick-Spülfeldern in den oberen Grundwasserleiter der Hamburger Elbmarsch. Dissertation Universität Hamburg 1991. Hamburger Bodenkundliche Arbeiten Band 17

[262] Kofod, M. (1994) Die Bedeutung frühdiagenetischer Prozesse für die Porenwasserzusammen­setzung in anaeroben Baggerschlämmen. Dissertation Universität Hamburg 1994. Hamburger Bodenkundliche Arbeiten Band 28

[263] Hille J, Ruske R, Scholz RW, Walkow F (Hrsg., 1992) Bitterfeld – modellhafte ökologische Bestandsaufnahme einer kontaminierten Industrieregion. Erich Schmidt Verlag Berlin

[264] Krapp L, Ruske R (1992) Geologische Verhältnisse von Bitterfeld und ihre Relevanz zu Kontaminationen des Grund­wassers und Bodens. In: Hille J, Ruske R, Scholz R, Walkow F (Hrsg) Bitterfeld – modellhafte ökologische Bestands­aufnahme einer kontaminierten Industrieregion. S. 85-92. Erich Schmidt Verlag Berlin

[265] Köhler HJ, Krapp L, Braun M (1992) Konzepte und Handlungsprioritäten zur Siche-rung und Sanierung von Altabla­ge­rungen, Deponien und Grundwasser im Großraum Bitterfeld/Wolfen. In: Hille J, Ruske R, Scholz RW, Walkow F (Hrsg) Bitterfeld – modellhafte ökologische Bestandsaufnahme einer kontaminierten Industrieregion. S. 203-210. Erich Schmidt Verlag Berlin

[266] Scholz RW, Nothbaum N, May TW, Brockmann R, Bode H, Deubel K-H, Hippe U (1992) Klassifikation CKW-kon­ta­minierter Flächen im Überschwemmungsgebiet der Mulde und Elbe. In: Hille J, Ruske R, Scholz RW, Walkow F (Hrsg.) Bitterfeld – modellhafte ökologische Bestandsaufnahme einer kontaminierten Industrieregion. S. 171-182. Erich Schmidt Verlag Berlin

[267] Walkow F (1996) The dioxin pollution of Bitterfeld. Lecture held at the meeting „Chemistry, Man and Environment – 20th Anniversary of the Seveso Accident“ Milan, October 2000

[268] Franke S, Heinzel N, Specht M, Francke W (2004) AP 3.10: Organische Schadstoffe im Gebiet der Unteren Mulde – Non Target Screening: Analytik von Wasser, Grundwasser und Sediment – Ergebnisse und Konsequenzen. In: Geller W. et al. (Hrsg.) Schad-stoffbelastung nach dem Elbe-Hochwasser 2002. Endbericht des Ad-hoc-Verbundprojekts, S. 206-223

[269] Schwartz R, Kozerski HP (2004) AP 4.2: Bestimmung des Gefahrenpotentials feinkörniger Buhnenfeldsedimente für die Wasser- und Schwebstoffqualität der Elbe sowie den Stoffeintrag in Auen. In: Geller W. et al. (Hrsg.) Schadstoff­belastung nach dem Elbe-Hochwasser 2002. Endbericht des Ad-hoc-Verbundprojekts, S. 258-274

[270] Pepelnik R, Niedergesäß R, Erbslöh B, Aulinger A, Prange A (2004) AP 3.2: Längsprofiluntersuchungen zur Beurtei­lung von Auswirkungen des Hochwassers vom August 2002 auf die Wasser- und Sedimentqualität der Elbe. In: Geller W. et al. (Hrsg.) Schadstoffbelastung nach dem Elbe-Hochwasser 2002. Endbericht des Ad-hoc-Verbundprojekts, S. 82-100

[271] Baborowski M, von der Kammer F, Friese K (2004) Teilprojekt 5: Kolloide und Schadstoffe (Schwermetalle) in der Elbe bei Hochwasserereignissen. In: Geller W. et al. (Hrsg.) Schadstoff­belastung nach dem Elbe-Hochwasser 2002. Endbericht des Ad-hoc-Verbundprojekts, S. 287-304

[272] Förstner U, Jacobs P (2004) Schwermetall-Freisetzung aus belasteten Überflutungsflächen in Folge (bio-)chemischer und physikalischer Einflüsse. In: Geller, W. et al. (Hrsg.) Schadstoff­belastung nach dem Elbe-Hochwasser 2002. AP 4.5. S. 275-286. BMBF/-UFZ Magdeburg.

[273] Witt O, Westrich B (2003) Quantification of erosion rates for undisturbed cohesive sediment cores by image analysis. Hydrobiologia 494(1-3)271-276

[274] Witt O (2004) Erosionsstabilität von Gewässersedimenten mit Auswirkungen auf den Stofftransport bei Hochwasser am Beispiel ausgewählter Staustufen am Oberrhein. Institut für Wasserbau, Universität Stuttgart, Mitteilungen H 127

[275] Joziasse J, van der Gun J (2000) In situ remediation of contaminated sediments: Conceivable and feasible? In: Conta­mi­nated Soil 2000, Vol I, pp. 516-522. Thomas Telford, London

[276] Förstner U et al.: BMBF-Verbundprojekt SEDYMO – Feinsedimentdynamik und Schadstoffmobilität in Fließge­wäs­sern 01.05.2002–31.07.2006, Schlussbericht, Hamburg, 2006

[277] Westrich B, Förstner U (Eds. 2007) Sediment Dynamics and Pollutant Mobility in Rivers – An Interdisciplinary Approach. Springer, Berlin

[278] Thomas R, Meybeck M (1992) The use of particulate material, in: Chapman D (ed) Water Quality Assessment, A Guide to the Use of Biota, Sediments and Water in Environmental Monitoring, Chapter 4, pp 121-170, Chapman & Hall, London

[279] Apitz S, White S (2003) A conceptual framework for river-basin-scale sediment management. J Soils Sediments 3:132-138

[280] Bergmann H, Maass V (2007) Sediment regulations and monitoring programmes in Europe, in Sediment Risk Management and Communication, in: Heise S (ed) Sustainable Management of Sediment Resources, Vol. 3, pp 207-231, Elsevier, Amsterdam

[281] Förstner U (2004) Traceability of sediment analysis. Trends Anal Chem 23 (3):217-236.

[282] Förstner U, Heise S (2006) Assessing and managing contaminated sediments: requirements on data quality – from molecular to river basin scale. Croatica Chim Acta 79(1):5-15.

[283] Heise S, Förstner U (2007) Risk assessment on contaminated sediments in river basins – theoretical considerations and pragmatic approach. J Environ Monit 9: 943-952

[284] Förstner U, Heise S, Ahlf W, Westrich B (2008) Data quality assurance of sediment monitoring. In: Quevauviller P, Borchers U, Thompson KC, Simonart T (eds) The Water Framework Directive. Ecological and Chemical Status Monitoring, pp 371-386. John Wiley, Chichester, UK.

[285] Ahlf W, Feiler U, Heininger P, Heise S (2008) Monitoring sediment quality using toxicity tests as primary tools for any risk assessment. In: Quevauviller P, Borchers U, Thompson KC, Simonart T (eds) The Water Framework Direc­tive. Ecological and Chemical Status Monitoring, pp 255-269. John Wiley, Chichester, UK.

[286] Chapman PM (2000) The sediment quality triad: Then, now and tomorrow. Int J Environ Poll 13:351-356

[287] DiToro DM, Mahony JD, Hansen DJ, Scott KJ, Carlson AR, Ankley GT (1992) Acid volatile sulfide predicts the acute toxicity of cadmium and nickel in sediments, Environ Sci Technol 26: 96-101

[288] Mudroch A, Burbonniere RA (1994) Sediment preservation, processing, and storage. In: Mudroch A, MacKnight SD (eds) Technologies for Aquatic Sediments Sampling, 2nd ed, pp 131-169. Lewis Publ, Boca Raton FL

[289] Carr RS, Nipper M (eds, 2001) Summary of a SETAC Technical Workshop on Porewater Toxicity Testing: Biologi­cal, Chemical, and Ecological Considerations with a Review of Methods and Applications, and Recommendations for Future Areas of Research, Society of Environmental Toxicology and Chemistry, Pensacola, FL

[290] Quevauviller Ph (ed, 2002) Methodologies for Soil and Sediment Fractionation Studies, The Royal Society of Chemi­stry, Cambridge, UK

[291] Haag I, Kern U, Westrich B (2001), Erosion investigation and sediment quality measurement for a comprehensive risk assessment of contaminated aquatic sediment. Sci Total Environ 266:249-257

[292] Quevauviller P (2004) Traceability of environmental chemicl measurements. Trends Anal Chem 23:171-177

[293] Gerbersdorf SU, Jancke T, Westrich B (2007) Sediment properties for assessing the erosion risk of contaminated rive­rine sites. J Soils Sediments7: 25-35

[294] Smit MPJ, Grotenhuis T, Bruning H, Rulkens WH (2008) Desorption of dieldrin from field aged sediments: Simula­ting flood events. J Soils Sediments 8: 80-85

[295] De Weert J, Streminska M, Hua D, Grotenhuis T,Langenhoff A,Rijnaarts H (2010) Nonylphenol mass transfer from field-aged sediments and subsequent biodegradation in reactors mimicking different river conditions. J Soils Sedi­ments 10:77-88

[296] Wölz J, Fleig M, Schulze T, Maletz S, v Varel UL,Reifferscheid G, Kühlers D, Braunbeck T, Brack W, Hollert H (2010): Impact of contaminants bound to suspended particulate matter in the context of flood events. J Soils Sediments 10: 1174-1185

[297] Brinkmann M, Hudjetz J, Cofalla C, Roger S, Kammann U, Giesy JP, Hecker M, Wiseman S, Zhang X, Wölz J, Schüttrumpf H, Hollert H (2010) A combined hydraulic and toxicological approach to assess resuspended sediments during simulated flood events. Part I – multiple biomarkers in rainbow trout. J Soils Sediments 10: 1347-1361

[298] Schüttrumpf H, Brinkmann M, Cofalla C, Frings RM, Gerbersdorf SU, Hecker M, Hudjetz S, Kammann U, Lennartz G, Roger S, Schäffer A, Hollert H (2011) A new approach to investigate the interactions between sediment transport and ecotoxicological processes during flood events. Environ Sciences Europe 23: 39

[299] S.U. Gerbersdorf, H. Hollert, M. Brinkmann, S. Wieprecht, H. Schüttrumpf, W. Manz: Anthropogenic pollutants affect ecosystem services of freshwater sediments: The need for a “triad plus x” approach. J Soils Sediments 2011, 1099-1114

[300] Hollert H, Brinkmann M, Hudjetz S, Cofalla C, Schüttrumpf H (2014) Hochwasser – ein unterschätztes Risiko (Schadstoffe als „Zeitbomben“ im Sediment). Biologie in unserer Zeit 1/2014, S. 44-51

**Literatur II**^[[17]](#footnote-17)^

Ahlf W, Feiler U, Heininger P, Heise S (2008) Monitoring sediment quality using toxicity tests as primary tools for any risk assessment. In: Quevauviller P, Borchers U, Thompson KC, Simonart T (eds) The Water Framework Directive. Ecol­o­gi­cal and Chemical Status Monitoring, pp 255-269. John Wiley, Chichester, UK [285]

Anonym (1973) Schatz auf Grund – Verseuchen giftige Metalle schon das Trinkwasser? Heidelberger Wissenschaftler mel­den alarmierende Befunde über Westdeutschlands Flüsse nach Bonn. Der Spiegel 3/73, S. 114, 15.1.1973 **[229]**

Anonym (1983) Blutender Berg – jahrelang verharmlosten Hamburger Behörden die Umweltgefahren, die vom höchsten Müllberg der Nation ausgehen. Der Spiegel 51/83, S. 25-26 **[61]**

Anonym (1983) Grube ohne Grenze. Jahrelang schickten westeuropäische Firmen und Städte Sondermüll auf eine grenz­nahe DDR-Kippe. Das Gift droht nun auf dem Wasserweg zurückzukommen. Der Spiegel 29/1983 **[68]**

Anonym (1986) Leitbild für die schweizerische Abfallwirtschaft. Schriftenreihe Umweltschutz Nr. 51. Eidgenössische Kommission für Abfallwirtschaft. Bundesamt für Umweltschutz, Bern [65]

Anonym (1988) Feststofftransport in Fließgewässern - Berechnungsverfahren für die Ingenieurpraxis. DVWK-Schriften Nr. 87, Verlag Paul Parey Hamburg [220]

Anonym (1990) Das gibt Revolte. Neue Hiobsbotschaften aus Bitterfeld und Umgebung: Die Dioxinwerte liegen höher als einst in Seveso. Die Fakten will niemand so recht wahrhaben. Der Spiegel 40/1990, S. 46-54. **[1]**

Anonym (1992a) Alles durch den Ausguss – die ostdeutschen Behörden schaffen es nicht, ihre Umweltprobleme unter Kontrolle zu bringen. Der Spiegel 9/92, S. 82-85, 24.02.1992 **[9]**

Anonym (1992b) Development Programme for Treatment Processes for Polluted Aquatic Sediments in the Netherlands (POSW I). Phase I (1989-1990). PO Box 17. 8200 AA Lelystad, The Netherlands [88]

Anonym (1992c) Studie über die Umlagerung von Sedimenten in Wasserstraßen. DVWK-Materialien, H 2/92. Bonn [221]

Anonym (1993a) Machbarkeitsstudie zur Sanierung des Spittelwassersediments. UBS Schwerin, IGB Hamburg im Auf-trag des Landratsamtes Bitterfeld, Juli 1993. (unveröffentlicht).[7]

Anonym (1993b) Ein Sieb, ein Loch – ein neues Gutachten zur Skandaldeponie Schönberg setzt Schwerins christdemo­kratische Umweltministerin Petra Uhlmann unter Druck. Der Spiegel 12/1993, S. 30-31 **[70]**

Anonym (1993c) Verlandung von Flußstauhaltungen. DVWK-Schriften Nr. 105, Verlag Paul Parey Hamburg [222]

Anonym (1994a) Der Müll und die Moneten. Während das Land Mecklenburg-Vorpommern für Altlasten zahlt, streichen Privatfirmen die Gewinne ein; jetzt durchleuchten Staatsanwälte das Geflecht. Der Spiegel v. 21.02.1994 [69]

Anonym (1994b) Distribution of PCDDs and PCDFs in surface freshwater systems. AEA Technology, National River Authority, R&D Note 242, Bristol, UK [123]

Anonym (1994c) Die Belastung der Elbe. Teil I - Elbenebenflüsse. Statusberichte der vom Bundesministerium für Bil­dung, Wissenschaft, Forschung und Technologie geförderten Elbe-Verbundvorhaben. Forschungszentrum Karlsruhe GmbH, Projekträger Wassertechnologie und Schlammbehandlung (Hrsg.). 170 S. [245]

Anonym (1994c): AG Boden. Bodenkundliche Kartieranleitung, 4 Aufl., Schweizerbart'sche Verlagsbuchhandlung, Stutt­gart, 392 p [178]

Anonym (1995) Dioxin-Bilanz für Hamburg. Studie der Universität Bayreuth und Trischler & Partner GmbH im Auftrag der Umweltbehörde Hamburg, Sept. 1995.**[44]**

Anonym (1997a) Aktuelle Bewertung der Gefahrensituation des Spittelwassers I, 8/1997. Staatliches Amt für Umwelt-schutz Dessau/Wittenberg. (unveröffentlicht) [10].

Anonym (1997b) Development Programme for Treatment Processes for Contaminated Sediments (Phase II). Final Report nr 97.051. ISBN 90 369 50 97 X. PO Box 17. 8200 AA Lelystad, The Netherlands [89]

Anonym (1997c) Wasserwirtschaftliche Bedeutung der Festlegung und Freisetzung von Nährstoffen durch Sedimente in Fließgewässern. DVWK-Schriften Nr. 115, Verlag Paul Parey, Hamburg [223]

Anonym (1997d) Contaminated Sediments in Ports and Waterways: Cleanup Strategies and Technologies: Committee on Contaminated Marine Sediments, Marine Board, Commission on Engineering and Technical Systems. National Research Council. National Academy Press. 295 p, Washington D.C. [113]

Anonym (1997e) National Conference on Management and Treatment of Contaminated Sediments, Proceeedings Cincin­na­ti, OH May 13-14, 1997. Technology Transfer and Support Division. National Risk Management Research Laboratory, Office of Research and Development, U.S. Environmental Protection Agency, EPA/625/R-98/001, August 1998, 155 p, Cincinnati, OH [114]

Anonym (1998) Contaminated sediments in the Sheffield and South Yorkshire Navigation (SSYN): Risk assessment of options. Scott Wilson Ltd. British Waterways, Gloucester, UK [125]

Anonym (2000a) Richtlinie 2000/60/EG des Europäischen Parlaments und des Rates vom 23.10.2000 zur Schaffung eines Ordnungsrahmens für Maßnahmen der Gemeinschaft im Bereich der Wasserpolitik (ABl. L 327 v 22.12.2000, S. 1) [4]

Anonym (2000b) Aktuelle Bewertung der Gefahrensituation des Spittelwassers II, 10/2000. Staatliches Amt für Umwelt­schutz Dessau/Wittenberg. (unveröffentlicht) [11]

Anonym (2000c) Contaminated Soil 2000, Case Study: Comparison of Solutions for a Large Contamination Based on Different National Policies. 7th International FZK/TNO Conference on Contaminated Soil. Leipzig. 163 p. [20]

Anonym (2000d) Case study of contaminated land management at Bitterfeld by UK team (Bob Harris, Mary Harris, Naomi Earl, Richard Swannell & Jonathan Smith. 18 Seiten [21]

Anonym (2000e) National approaches for a Bitterfeld Case – Danish Contribution (Tom Heron [team leader], Lizzi Andersen & Lars Kaalund). 16 Seiten [22]

Anonym (2000f) Fallstudie Bitterfeld. Umgang mit Kontaminationen in Flusseinzugs- und Überschwemmungsgebieten am Beispiel des Niederungsgebietes „Spittelwasser“ Bitterfeld. Deutscher Beitrag zum Fallstudienvergleich ConSoil 2000 vom 18.09.-22.09.2000 in Leipzig, 27 S. (Arbeitsgruppe des BMBF-Projektträgers für Abfallwirtschaft und Altlasten­sanierung: Wittmann U [Projektorganisation], Förstner U [Wissenschaftlicher Projektleiter], Gier S, Delschen Th, Franzius V, Frauenstein J, Fuchs E, Gläßer W, Lindemann M, Müller R, Meiners G, Wetzel V, Wilke B [24]

Anonym (2004a) EU-UmwHaftRL – EU-Umwelthaftungsrichtline. Richtlinie 2004/35/EG des Europäischen Parlaments und des Rates vom 21.04.2004 über Umwelthaftung zur Vermeidung und Sanierung von Umweltschäden. Amtsblatt der Europäischen Union Nr. L143 vom 30.04.2004. S. 56-75 [25]

Anonym (2004b) Case Studies of Environmental Dredging Projects. Vol. 5: Appendix in: Engineering Performance Standards Hudson River PCBs Superfund Site. 146 p. Prepared for U.S. Army Corps of Engineers, Kansas City District, USACE Contract No. DACW41-02-D-0003, on behalf of U.S. Environmental Protection Agency, Region 2. Prepared by Malcolm Pirnie, Inc. 104 Corporate Park Drive, White Plains, New York 10602 and TAMS Consultants, Inc. an Earth Tech Company 300 Broadacres Drive Bloomfield, New Jersey 07003, April 2004 **[120]**

Anonym (2004c) Umstrittene Sedimentspülung am Rhein – Chemie-Altlast von Hochrhein wandert bis zur Nordsee. Neue Zürcher Zeitung 30.12.2004 **[207]**

Anonym (2005a) EU-Forschungsvorhaben WELCOME (Water, Environment and Landscape Management at Conta-minated Megasites). Abschlussbericht 2005 [32]

Anonym (2005b) Freistellungsfinanzierte Altlastensanierung – Neue Herausforderungen in fachlicher, administrativer und finanzplanerischer Hinsicht. Tagung der Landesanstalt für Altlastenfreistellung des Landes Sachsen-Anhalt und des Ingenieurtechnischen Verbandes Altlasten, 9. Juni 2005 Magdeburg. 185 S. **[34]**

Anonym (2005c) Chemischer Zoo – seit der Jahrhundertflut kommen die Hinterlassenschaften der DDR-Giftküche Bitterfeld an die Oberfläche. Der Spiegel v. 23.12.2005, S. 36 [40]

Anonym (2005d) Contaminated Sediment Remediation Guidance for Hazardous Waste Sites. Kapitel 5 “Monitored Natural Recovery” (4-1 bis 4-11). Weitere Superfund-Technologien für Sedimente: „In-Situ Capping“(5-1 bis 5-14) und „Dred­ging & Excavation“(6-1 bis 6-36). United States Environmental Protection Agency. EPA-540-R-05-012. Dezember 2005 [116]

Anonym (2005e) Die Elbe und ihr Einzugsgebiet – ein geographisch-hydrologischer und wasserwirtschaftlicher Überblick. Internationale Kommission zum Schutz der Elbe. Magdeburg 258 S. [143]

Anonym (2005f) Relocation of sediment from Iffezheim weir. Complaint to EU. Amsterdam, 27 April 2005, Lycklama, T., representing the NGOs Stichting Reinwater, Stichting De Noordzee, Waddenvereniging, Gelderse Milieufederatie, Natuur en Milieufederatie Utrecht, Rhein Kolleg **[208]**

Anonym (2006) Information zur Sanierung der Altlasten des Braunkohlebergbaus in den neuen Ländern. Bundesministe­rium für Umwelt, Naturschutz und Reaktorsicherheit. Bericht, 8 Seiten [144]

Anonym (2007a) USchadG – Umweltschadensgesetz. Gesetz zur Umsetzung der Richtlinie des Europäischen Parlaments und des Rates über Umwelthaftung zur Vermeidung und Sanierung von Umweltschäden vom 10. Mai 2007. Bunde­s­gesetzblatt Teil I Nr. 19, S. 666-671 [26]

Anonym (2007b) Sediment Dredging at Superfund Megasites: Assessing the Effectiveness. Committee on Sediment Dred­ging at Superfund Megasites. Board on Environmental Studies and Toxicology, Division on Earth and Life Studies, National Research Council oft he National Academies. National Academy Press. 294 p, Washington, D.C [117] .

Anonym (2008a) Analyse und Bewertung der technischen Möglichkeiten für eine Problemlösung im Bereich der Ober­flächenwässer des Grundwasserkörpers in Bitterfeld. Erstellt für Großmann Ingenieur Consult GmbH (GICON), 19. Juni 2008, 50 Seiten, WISUTEC Chemnitz (unveröffentlicht) [78]

Anonym (2008b) Gewässerunterhaltungsmaßnahme Wiederherstellung des Abflussprofils des Schachtgrabens. Abschluss­dokumentation. Erstellt für P-D ChemiePark Bitterfeld Wolfen GmbH/Landesanstalt für Altlastenfreistellung Sachsen-Anhalt, 05. November 2008, 27 Seiten, WISUTEC Chemnitz (unveröffentlicht) [79]

Anonym (2008c) Richtlinie 2008/56/EG des Europäischen Parlaments und des Rates vom 17.06.2008 zur Schaffung eines Ordnungsrahmens für Maßnahmen der Gemeinschaft im Bereich der Meeresumwelt (Meeresstrategie-Rahmenricht­linie). Amtsblatt der Europäischen Union vom 25.6.2008. L 164/19 [211]

Anonym (2008d) Strombau- und Sedimentmanagementkonzept für die Tideelbe. Hamburg Port Authority und Wasser- und Schifffahrtsverwaltung des Bundes. 39 S., 1. Juni 2008 **[213]**

Anonym (2009a) Bewirtschaftungsplan nach Artikel 13 der Richtlinie 2000/60/EG für den deutschen Teil der Flussgebiets­gemeinschaft Elbe, 11. November 2009 [51]

Anonym (2009b) Hintergrundpapier zur Ableitung der überregionalen Bewirtschaftungsziele für die Oberflächengewässer im deutschen Teil der Flussgebieteseinheit Elbe für den Belastungsschwerpunkt Schadstoffe, Abschlussbericht, FGG Elbe, 02.04.2009, Magdeburg. **[53]**

Anonym (2009c) Kz 05.022.0014. Ausschreibungsanzeiger Sachsen-Anhalt vom 29. Mai 2009, S. 37/38 Wettbewerblicher Dialog „Frachtreduzierung überwiegend schwebstoffgebundener Schadstoffe der im Grundwasserkörper VM 2-4 gele­genen Fließgewässer mit dem Schwerpunkt Spittelwasser/Schachtgraben“. Pilotprojekt Bitterfeld-Wolfen zur Umsetz­ung der WRRL im Land Sachsen-Anhalt [92]

Anonym (2009d) Sedimentmanagementplan Rhein. Zusammenfassung. Internationale Kommission zum Schutz des Rheins. Bericht Nr. 175. 101 S. **[210]**

Anonym (2009e) Auftaktveranstaltung zum „Sedimentmanagementkonzept Sachsen-Anhalt“, der Schlüsselregion im IKSE-Konzept (am 12./13. Mai 2009 in Dresden beschlossen die Delegationsleiter der Intern. Kommission zum Schutz der Elbe die Gründung einer IKSE-Expertengruppe „Sediment-Management“) am 25.09.2009 in Magdeburg; Vortrag U. Hursie, Referat 27, MLU Sachsen-Anhalt, 22 Folien, 2.3 MB [100]

Anonym (2009e/2013) Gemeinsame Übergangsbestimmungen zum Umgang mit Baggergut in den Küstengewässern. August 2009, 39 Seiten.**[212]**

Anonym (2010a) 10 Jahre Landesanstalt für Altlastenfreistellung in Sachsen-Anhalt. Magdeburg, 32 S. [**[3**](http://www.laf-lsa.de/fileadmin/Bibliothek/Politik_und_Verwaltung/MLU/LAF/Dokumente/20100127_Broschuere_LAF_fuer_Inter-net.pdf%20%5b3)**]**

Anonym (2010b) Landesbetrieb für Hochwasserschutz und Wasserwirtschaft Sachsen-Anhalt Gewässerkundlicher Landesdienst (LHW). Nr. 1/2010, 35 S. **[39]**

Anonym (2010c) 25 Jahre Sanierung der Deponie Georgswerder – Umgang mit baulich gesicherten Altlasten: Erkennt-nisse und Perspektiven. Fachtagung vom 10./11. Juni 2010, Behörde für Stadtentwicklung und Umwelt, Amt für Umweltschutz, Abt Bodenschutz/Altlasten, Hamburg.184 S.**[63]**

Anonym (2011a) Common Implementation Strategy for the Water Framework Directive (2000/60/EC: Technical Guidance for Deriving Environmental Quality Standards, Guidance Document No. 27, European Communities 2011, 204 S.[181]

Anonym (2011b) Rheinschlamm landet diesmal in den Niederlanden. Badisches Tagblatt, Artikel vom 19.02.2011 [209]

Anonym (2012) Bestandsaufnahme belasteter Altsedimente in ausgewählten Gewässern Sachsen-Anhalts. Phase II. Sedi­mentbeprobung und Sedimentuntersuchungen. G.E.O.S. Ingenieurgesellschaft mbH, im Auftrag von: Landesbetrieb für Hochwasserschutz und Wasserwirtschaft Sachsen-Anhalt; 30.11.2012; 252 S. **[106]**

Anonym (2013a) Richtlinie 2013/39/EU des Europäischen Parlaments und des Rates vom 12.08.2013 zur Änderung der Richtlinien 2000/60/EG und 2008/105/EG in Bezug auf prioritäre Stoffe im Bereich der Wasserpolitik. ABl. L 226 vom 24.08.2013, 17 S. [14]

Anonym (2013b) Sediment Remedy Effectiveness and Recontamination: Selected Case Studies. Association of State and Territorial Solid Waste Management Officials ASTSWMO Sediment Focus Group, 85 pp. **[118]**

Anonym (2013c) Use of Amendments for In Situ Remediation at Superfund Sediment Sites. United States Environmental Protection Agency, Office of Superfund Remediation and Technology Innovation. OSWER Directive 9200.2-128FS, 61 p. EPA April 2013 [119]

Anonym (2013d) Superfund remedial program review action plan. U.S. Environmental Protection Agency, Wash. D.C., 43 pages **[184]**

Anonym (2014a): Sedimentmanagement Tideelbe - Strategien und Potenziale - Systemstudie II. Ökologische Auswir-kungen der Unterbringung von Feinmaterial. Band 1 (2), Endbericht. Im Auftrag des Wasser- und Schifffahrtsamtes Hamburg. Bundesanstalt für Gewässerkunde, Koblenz, BfG-1763. DOI: 10.5675/BfG-1763 [13]

Anonym (2014b) Sedimentmanagementkonzept der FGG Elbe. Vorschläge für eine gute Sedimentmanagementpraxis im Elbe­­­gebiet zur Erreichung überregionaler Handlungsziele. Flussgebietsgemeinschaft Elbe. 25.11.2013. Magdeburg, 383 S. **[58]**

Anonym (2014c) Sedimentmanagementkonzept der IKSE. Vorschläge für eine gute Sedimentmanagementpraxis im Elbe­gebiet zur Erreichung überregionaler Handlungsziele. Int Kommission zum Schutz der Elbe. Magdeburg, 202 S. **[59]**

Anonym (2014c) Wer karrt denn Kochsalz durch halb Europa? Berliner Zeitung v. 04.01.2014  **[71]**

Anonym (2014d) 1979: Der Ihlenberg wird zur Deponie. NDR, 30.01.2014. **[72]**

Anonym (2014d) Ehemaliges Werk der Fa. Boehringer Ingelheim. Behörde für Stadtentwicklung und Umweltschutz Hamburg. **[67]**

Anonym (2014e) Entwurf der Aktualisierung des Bewirtschaftungsplans nach § 83 WHG bzw. Artikel 13 der Richtlinie 2000/60/EF für den deutschen Teil der Flussgebietseinheit Elbe für den Zeitraum von 2016 bis 2021. Flussgebiets­gemeinschaft Elbe. Magdeburg, 22.12.2014, 238 S. [108]

Anonym (2014f) Hudson River PCBs Summer 2014. (i) Where Are We [131], (ii) Environmental Dredging [132], (iii) Summary of Presentations [133], and many other reports 2006-2014. Hudson River Sloop Clearwater.**[130]**

Anonym (2014g) Hudson River PCB Information: Environmental Dredging. Hudson River Sloop Clearwater, News & Bulletins. **[132]**

Anonym (2014h) Summary of Presentations. 2014 Hudson River PCB Forum, Marist College Boathouse, November 11, 2014. **[133]**

Anonym (2014i) The Passaic River’s Polluted Past, May 2014. U.S. EPA, Region 2, 4 p **[136]**

Anonym (2014j) Lower Passaic River Restoration Project. U.S. Environmental Protection Agency, Region 2 **[138]**

Anonym (2014k) An Overview of EPA’s Cleanup Plan, May 2014. U.S. EPA, Section 2 **[139]**

Anonym (2014l) An Overview of the Options for Cleaning up Contaminated Sediment **[140]**

Anonym (2014m) Contaminated Sediments Remediation. Remedy Selection for Contaminated sediments. Prepared by Interstate Technology and Regulatory Council. Contaminated Sediments Team. ITRC Guidance Document August 2014, 525 pages **[185]**

Anonym (o.J.) ELSA – Schadstoffsanierung Elbsedimente. Projektinformation. [http://www.elsa-elbe.de/projekt-informa­tion.html](http://www.elsa-elbe.de/projekt-information.html) **[215]**

Apell JN, Gschwend PM* (2014) Validating the use of performance reference compounds in passive samplers to assess porewater concentrations in sediment beds. Environ Sci Technol 48(17) 10301-10307 [192]

Apitz S, White S (2003) A conceptual framework for river-basin-scale sediment management. J Soils Sediments 3:132-138 [279]

Baborowski M, Bozau E (2006) Impact of former mining activities on the uranium distribution in the River Saale (Ger­many). Appl Geochem 21: 1073-1082 [161]

Baborowski M, Mages M, Hiltscher C, Matschullat J, Guhr H (2006) Former mining activities influence uranium concen­trations om the Elbe river near Magdeburg. In: Merkel BJ, Hasche-Berger A (Hrsg) Uranium in the Environment. S. 585-592. Springer Berlin [162]

Baborowski M, von der Kammer F, Friese K (2004) Teilprojekt 5: Kolloide und Schadstoffe (Schwermetalle) in der Elbe bei Hochwasserereignissen. In: Geller W. et al. (Hrsg.) Schadstoff¬belastung nach dem Elbe-Hochwasser 2002. End­bericht des Ad-hoc-Verbundprojekts, S. 287-304 [271]

Babut M, Oen A, Hollert H, Apitz SE, Heise E, White S (2006) Priorization at river basin scale, risk assessment at local scale: suggested approaches. In: Heise S (ed) Sediment Risk Assessment and Communication, pp 107-151. Elsevier, Amsterdam [200]

Bahlburg CH (2005) Hochwasser und andere Katastrophen – Was haben wir gelernt? In: Karl H, Pohl J, Zimmermann H (Hrsg) Risiken in Umwelt und Technik – Vorsorge durch Raumplanung. Akademie für Raumforschung und Landes-planung. Forschungs- und Sitzungsberichte, Band 223, S. 3-14. Hannover (ISBN 3-88838-052-9) [85]

Bergmann H, Maass V (2007) Sediment regulations and monitoring programmes in Europe, in Sediment Risk Management and Communication, in: Heise S (ed) Sustainable Management of Sediment Resources, Vol. 3, pp 207-231, Elsevier, Amsterdam [280]

Biener E, Sasse T, Wemhoff T, Jacobsen N (2014) . Erfahrungen bei der Umsetzung des Konzeptes „Deponie auf Deponie“. 30. Fachtagung „Die sichere Deponie – Geokunststoffe im Umweltschutz“.[73]

Bley S, Gerth J, Neumann-Hensel H, Ruttkowski V (2006) Methoden der Erfassung, Bewertung und Prognose der intrin­sisch/­zeitlich verstärkten Schadstoffrückhaltung in kontaminierten Sedimenten (KORA, TV 6.1). Schlussbericht des gleichnamigen Projekts an der TU Hamburg-Harburg und Fa. Fintelmann & Meyer, Hamburg. Förderkennzeichen: 0330519. Laufzeit des Vorhabens: 01.10.2003-31.12.2006 [94]

Böhme M, Krüger F, Ockenfeld K, Geller W (Hrsg., 2005) Schadstoffbelastung nach dem Elbe-Hochwasser 2002. Broschüre UFZ – Umweltforschungszentrum Leipzig-Halle GmbH, Department. Fließgewässerökologie. 101 S. [41]

Bonnenberg H (2010) Meine Erfahrungen bei der Treuhandanstalt und ihren Folgegesellschaften. Vortrag anlässlich der Anhörung der Bundestagsfraktion DIE LINKE. Marie Elisabeth Lüders Haus des Bundestags, Berlin, 19. April 2010. 16 S. **[2]**

Bopp RF, Gross ML, Tong H, Simpson HJ, Monson SJ, Deck BL, Moster FC (1991) A major incident of dioxin con-tamination: Sediments of New Jersey estuaries. Environ Sci Technol 25(5): 951-956 [137]

Breuer R (2008) Sedimentmanagement für die Elbe. Rechtsgutachten, 234 S. Nomos Verlag Baden-Baden **[5]**

Brinkmann M, Hudjetz J, Cofalla C, Roger S, Kammann U, Giesy JP, Hecker M, Wiseman S, Zhang X, Wölz J, Schütt­rumpf H, Hollert H (2010) A combined hydraulic and toxicological approach to assess resuspended sediments during simulated flood events. Part I – multiple biomarkers in rainbow trout. J Soils Sediments 10:1347-1361 [297]

Broekaert JAC, Siemens V, Krugmann T, Schlenker A (2004) AP 3.8: Schwermetall- und Arsenverlagerung in der Ver­einig­ten Mulde. In: Geller W et al. (Hrsg.) Schadstoffbelastung nach dem Elbe-Hochwasser 2002. Endbericht des Ad-hoc Verbundprojekts, S. 173-182 [165]

Buchwald H (1994) Bitterfeld – ein Park voller Müll. Der Vorzeige-Chemiestandort im Dilemma. Firmen entsorgen illegal. Abwässer sind vergiftet. FOCUS Magazin, Nr. 35 v. 29.08.1994 [75]

Büttner O, Böhme M, Rode M (2009) Teilprojekt 3: Hydraulische Modellierung. Abschn 3.8 Überflutung des Salegaster Forsts durch das Hochwasser der Mulde. In: v Tümpling W, Rode M, Böhme M, Gläßer C, Matthies M, Schanze J (Hrsg) SARISK – Entwicklung eines Schadstoffausbreitungsmodells zur stoffbezogenen Risikoanalyse und –bewer-tung extremer Hochwasserereignisse am Beispiel des Landkreises und der Stadt Bitterfeld. Endbericht des Verbund-projektes. BMBF-Förderkennzeichen PTJ 0330690 A-D. Seiten 49-80 [83]

Calmano W (Hrsg, 2001) Untersuchung und Bewertung von Sedimenten. Springer, Berlin [227]

Calmano W, Förstner U (Hrsg 1996) Sediments and Toxic Substances – Environmental Effects and Ecotoxicity. Springer-Verlag Berlin [234]

Carr RS, Nipper M (eds, 2001) Summary of a SETAC Technical Workshop on Porewater Toxicity Testing: Biological, Chemical, and Ecological Considerations with a Review of Methods and Applications, and Recommendations for Future Areas of Research, Society of Environmental Toxicology and Chemistry, Pensacola, FL [289]

Chapman PM (2000) The sediment quality triad: Then, now and tomorrow. Int J Environ Poll 13:351-356 [286]

Choi Y, Cho Y-M, Werner D*, Luthy RG (2014) In situ sequestration of hydrophobic organic contaminants in sedi-ments under stagnant contact with activated carbon. 2 Mass transfer modeling. Environ Sci Technol 48(3)1843-1850 [189]

Cornelissen G*, Amstaetter K, Hauge A, Schaanning M, Beylich B, Gunnarsson J, Breedveld G, Oen A, Eek E (2012) Large-scale field study on thin-layer capping of marine PCDD/F contaminated sediments in Grenlandfjords, Norway: Physicochemical effects. Environ Sci Technol 46:12030–12037 [186]

Czegka W, Hanisch C, Junge F, Zerling L, Baborowski M (2006) Changes in uranium concentration in the Weisse Elster River as a mirror of the remediation in the former WISMUT mining area. In: Merkel BJ, Hasche-Berger A (Eds) Uranium in the Environment. S. 875-884. Springer Berlin [158]

de Beijer P (2004) Perspective on sediment management in European rivers. Presentation of the Port of Rotterdam at the SedNet Conference, November 25-26, 2004, Venice. <http://sednet.org/download/Stakeholders_de_Beijer_Day2.pdf> **[206]**

De Haar U (1978) Die Arbeit der Senatskommission für Wasserforschung 1957-1977. Senatskommission für Wasserfor­sch­ung. Mitteilung I. Harald Boldt Verlag, Boppard [217]

De Weert J, Streminska M, Hua D, Grotenhuis T,Langenhoff A,Rijnaarts H (2010) Nonylphenol mass transfer from field-aged sediments and subsequent biodegradation in reactors mimicking different river conditions. J Soils Sediments 10:77-88 [295]

De Wit N, Rijnaarts H, Faber J, Ykema K, Ouboter St, Satijn B (2000) Solutions for the Spittelwasser Pollutions – The Dutch Contribution for the Case Comparison Bitterfeld. A plan to be presented at ConSoil 2000 – FZK International Conference on Contaminated Land, 18-22 September 2000, Leipzig [23]

DiToro DM, Mahony JD, Hansen DJ, Scott KJ, Carlson AR, Ankley GT (1992) Acid volatile sulfide predicts the acute toxicity of cadmium and nickel in sediments, Environ Sci Technol 26:96-101[287]

Edwards AMC (2001) River nd estuary management issues in the Humber catchment. In: Huntley D, Leeks G, Waling D (eds) Land-Ocean Interaction: Measuring and Modelling Fluxes from River Basins to Coastal Seas; pp 8-31. IWA Publishing, London [122]

Eijsackers H (1995) How to manage accumulated contaminants.In: Salomons W, Stigliani WM (Eds) Biogeodynamic of pollutants in soils and sediments – risk assessment of delayed and non-linear responses; pp 309-329, Springer Berlin [242]

Einax J, Truckenbrodt D, Matschullat J, Naumann U (1996) Zur Schadstoffbelastung der Saale und der Schwarzen Elster. in: Prange A, Wilken R-D, von Tümpling U, Spoustova J, Punchochar P, Lencova E.(Hrsg) Ökosystem Elbe - Zustand, Entwicklung und Nutzung. 7. Magdeburger Gewässerschutzseminar, Tagungsband, S. 154 – 158 [250]

Evers EHG, Laane RWPM, Groenefeld GJJ (1996) Levels, temporal trends and risks of dioxins and related com-pounds in the Dutch aquatic environment. Organohalogen Compounds. 28:117–122 [18]

Evison L, Greenberg M, Logan M, Magar V, Nadeau S, Reible D (2007) Definition and demonstration of remedy effectiveness: What worked and what didn’t (Panel Discussion). In: Foote EA, Durell GS (Eds) Remediation of Contaminated Sediments 2007. Proc 4th Int Conf on Remediation of Contaminated Sediments (Savannah, Georgia, January 2007). Battelle Press, Columbus, OH [174]

Förstner U (1991) Sanierung von Deponien am Beispiel der Deponie Georgswerder. In: Albach H, Schade D, Sinn H (Hrsg) Technikfolgenforschung und Technikfolgenabschätzung. S. 295-319. Springer Berlin [64]

Förstner U (1993) Sediment problems related to chemical time bombs - three conceptual approaches. In: ter Meulen GBR, Stigliani WM, Salomons W, Bridges EM (Eds.) Chemical Time Bombs - European State-of-the-Art Conference on Delayed Effects of Chemicals in Soils and Sediments, pp. 129-146. Foundation for Ecodevelopment "Stichting Mon­dial Alternatief" Hoofdorp/The Netherlands. ISBN 90-71111-62-8 [239]

Förstner U (1995) Non-linear release of metals from aquatic sediments. In: Salomons W, Stigliani WM (Eds) Biogeodyna­mic of pollutants in soils and sediments – risk assessment of delayed and non-linear responses; pp 247-307. Springer Berlin [241]

Förstner U (1996) Contaminated sediments and remediation – geochemical perspective. In: Novotny V, Somlyódy L (Eds) Remediation and Management of Degraded River Basins with Emphasis on Central and Eastern Europa. NATO ASI Series, Partnership Sub-Series, 2. Environment, Vol 3, pp 231-253. Springer Berlin [238]

Förstner U (2003) Endlagerqualität, Verwertung und Nachhaltigkeit. In: Förstner U, Grathwohl P (Hrsg) Ingenieurgeo-chemie. S. 35-42. Springer-Verlag Berlin [66]

Förstner U (2003) Geochemical techniques on contaminated sediments – river basin view. Environ Sci Pollut Res 10 (1):58-68 [142]

Förstner U (2004) Traceability of sediment analysis. Trends Anal Chem 23 (3):217-236 [281] .

Förstner U (2008) Maßnahmen. Kapitel 6 in: S. Heise et al.: Bewertung von Risiken durch feststoffgebundene Schadstoffe im Elbeeinzugsgebiet, S. 247-341. Studie im Auftrag der Hamburg Port Authority, erstellt von BIS/TuTech, Hamburg 2008 [93]

Förstner (2011) Was ist aus der Dioxin-Altlast im Spittelwasser geworden? Die Rolle der Landesanstalt für Altlasten-freistellung von Sachsen-Anhalt bei der Umsetzung der EG Wasserrahmenrichtlinie im Flusseinzugsgebiet der Elbe, dargestellt am Beispiel des Pilotprojekts Bitterfeld-Wolfen, Dokumentation, 16 Seiten. <http://www.dioxindb.de/doku-mente/Dioxin_Spittelwasser_Dokumentation_Foerstner_2011> **[19]**

Förstner U (2013) Von der Greiferprobe bis zur In-situ Sanierung – 50 Jahre Forschung an kontaminierten Gewässer-sedimenten. Vom Wasser 111(4) 132-140 [110]

Förstner U, Apitz S (2007) Sediment remediation: U.S. focus on capping and monitored natural attenuation – 4th Inter­national Battelle Conference on Remediation of Contaminated Sediments. J Soils Sediments 7 (6):351-358 [111]

Förstner U, Heise S (2006) Assessing and managing contaminated sediments: requirements on data quality – from molecular to river basin scale. Croatica Chim Acta 79(1):5-15.[282]

Förstner U, Jacobs P (2004) Schwermetall-Freisetzung aus belasteten Überflutungsflächen in Folge (bio-)chemischer und physikalischer Einflüsse. In: Geller, W. et al. (Hrsg.) Schadstoffbelastung nach dem Elbe-Hochwasser 2002. AP 4.5. S. 275-286. BMBF/-UFZ Magdeburg [272]

Förstner U, Müller G (1974) Schwermetalle in Flüssen und Seen als Ausdruck der Umweltverschmutzung. 225 S. Springer-Verlag Berlin [228]

Förstner U, Salomons W (2010) Sediment research, management and policy. J. Soils Sediments 10:1440-1452 [80]

Förstner U, Winkler HA (Bearb, 1982) Untersuchung über das Vorkommen und Verhalten von Metalle und Metalloiden in Oberflächenwässern und Trinkwässern. Kap. I.1.7 Spurenmetalle in Schwebstoffen und Sedimenten, S. 25-32 in: Först¬ner U, De Haar U, Jüttner F, Müller H, Sonnenborn M, Winkler, HA (Hrsg.) Schadstoffe im Wasser. Metalle – Phenole – Algenbürtige Schadstoffe. DFG, Kommission für Wasserforschung Mitt. IV. Harald Boldt Verlag, Boppard [218]

Förstner U , Ackermann F, Alberti J, Calmano W, Frimmel F, Kornatzki K, Leschber R, Roßknecht H, Schleichert U, Tent L (1987) Allgemeine Problematik und heutiger Stand der Entwicklung von Sedimentqualitätskriterien. Z Wasser- Abwas­serforsch 20:54-59 [226]

Förstner U, Westrich B et al.:(2006) BMBF-Verbundprojekt SEDYMO – Feinsedimentdynamik und Schadstoffmobilität in Fließ­gewässern, 01.05.2002–31.07.2006, Schlussbericht, Hamburg Oktober 2006 [276]

Förstner U, Gerth J, Bley S, Neumann-Hensel H (2007) Methoden der Erfassung, Bewertung und Prognose der intrin­sisch/­zeitlich verstärkten Schadstoffrückhaltung in kontaminierten Sedimenten (KORA Teilvorhaben V 6). 138 Seiten, Fz 0330519. Laufzeit 01.10.2003 bis 31.12.2006. BMBF, Projektträger Jülich 2007 [176]

Förstner U, Heise S, Ahlf W, Westrich B (2008) Data quality assurance of sediment monitoring. In: Quevauviller P, Borchers U, Thompson KC, Simonart T (eds) The Water Framework Directive. Ecological and Chemical Status Monitoring, pp 371-386. John Wiley, Chichester, UK [284]

Franke S, Heinzel N, Specht M, Francke W (2004) AP 3.10: Organische Schadstoffe im Gebiet der Unteren Mulde – Non Target Screening: Analytik von Wasser, Grundwasser und Sediment – Ergebnisse und Konsequenzen. In: Geller W. et al. (Hrsg.) Schadstoffbelastung nach dem Elbe-Hochwasser 2002. Endbericht des Ad-hoc-Verbundprojekts, S. 206-223 [268]

Friedman CL, Lohmann R* (2014) Comparing sediment equlibrium partitioning and passive sampling techniques to esti­mate benthic biota PCDD/F concentrations in Newark Bay, New Jersey (U.S.A.). Environ Poll 186:172-179 [191]

Friese K, Witter B, Miehlich G, Rode M (Hrsg, 2000) Stoffhaushalt von Auenökosystemen – Böden und Hydrologie, Schad­stoffe, Bewertungen, 438 S. Springer Verlag Berlin [259]

Furrer R (1998) Ergebnisse des BMBF-Verbundvorhabens „Geogener Background im Elbe-Einzugsgebiet“, in: Geller W, Puncochar P, Bornhöft D, Boucek J, Feldmann H, Guhr H, Mohaupt V, Simon M, Smart’ak J, Spoustova J, Uhlmann O (Hrsg) Gewässerschutz in Einzugsgebiet der Elbe. 8. Magdeburger Gewässerschutzseminar, S. 78 - 81, B. G. Teubner Verlagsgesellschaft Stuttgart Leipzig [246]

Garvey EA, Atmadja J, Accardi-Dey AM (2007) Dioxins in the Newark Bay Complex – Newark, New Jersey; in: Foote EA, Durell GS (eds) Remediation of Contaminated Sediments 2007. Paper A-027, 31 p. Proc. 4th Intern Conf Reme­di­ation of Contaminated Sediments, Savannah, Ga., January 2007. ISBN 978-1-57477-159-8. Published by Battelle Press, Columbus , OH [134]

Gerbersdorf SU, Jancke T, Westrich B (2007) Sediment properties for assessing the erosion risk of contaminated riverine sites. J Soils Sediments 7: 25-35 [293]

Gerbersdorf SU, H. Hollert, M. Brinkmann, S. Wieprecht, H. Schüttrumpf, W. Manz: Anthropogenic pollutants affect ecos¬ystem services of freshwater sediments: The need for a “triad plus x” approach. J Soils Sediments 2011, 1099-1114 [299]

Gerth J, Förstner U (2011) Kontaminierte Gewässersedimente – 6.1.8 Projekt Flussauensedimente im KORA-Themen-verbund. In: Kern U, Westrich B (Hrsg.) Kontaminierte Gewässersedimente – Strategie, Fallbeispiele, Empfehlungen. DWA-Themen T 3/2011. Deutsche Vereinigung für Wasserwirtschaft, Abwasser und Abfall e.V. Hennef, S. 119-135 [28]

Ghosh U*, Driscoll SK, Burgess RM, Jonker MTO, Reible D, Gobas F,Choi Y, Apitz SE, Maruya KA, Gala WR, Mortimer M, Bega C (2014) Passive sampling methods for contaminated sediments: Practical guidance for selection, calibration, and implementation. Integr Environ Assess Manag 10(2)210-223 [197]

Ghosh U, Luthy R, Cornelissen G, Werner D, Menzie CA (2011) In-situ sorbent amendments: A new direction in contami­nated sediment management. Environ Sci Technol 45(4)1163-1168 [179]

Gilmour CC*, Riedel GS, Riedel G, Kwon S, Lands R, Brown SS, Menzie CA, Ghosh U (2013) Activated cabon mitigates mercury and methyl mercury bioavailability in contaminated sediments. Environ Sci Technol 47(22):13001-13010 [188]

Götz R, Lauer R (2003) Analysis of sources of dioxin contamination in sediments and soils using multivariate statisti-cal methods and neural networks. Environ Sci Technol 37:5559-5565 [46]

Götz R, Bauer OH, Friesel P, Herrmann T, Jantzen E, Kutzke M, Lauer R, Paepke O, Roch K, Rohweder U, Schwartz R, Sievers S, Stachel B (2007): Vertical profile of PCDD/Fs, dioxin-like PCBs, other PCBs, PAHs, chlorobenzenes, DDX, HCHs, organotin compounds and chlorinated ethers in dated sediment/soil cores from flood-plains of the river Elbe, Germany. Chemosphere 67:592-603 [55]

Götz R, Steiner D, Friesel P, Roch K, Walkow F, Maaß V, Reincke H (1996) Dioxin in the River Elbe – investigations of their origin by multivariate statistical methods. Organohalogene Compounds 27: 440-443 [6]

Götz R, Steiner D, Friesel P, Roch K, Walkow F, Maaß V, Reincke H (1998) Dioxin (PCDD/F) in the River Elbe – investi­gations of their origin by multivariate statistical methods. Chemosphere 37:1997-2002 [45]

Gröngröft A (1992) Untersuchung des Sickerwasser- und Stoffeintrags aus Hafenschlick-Spülfeldern in den oberen Grund­wasserleiter der Hamburger Elbmarsch. Dissertation Universität Hamburg 1991. Hamburger Bodenkundliche Arbeiten Band 17 [261]

Gröschke A, Uhlmann W, Rolland W, Grünewald U (2002) Hydrochemische Entwicklung Lausitzer Tagebauseen während der Flutung am Beispiel des Restloches Gräbendorf. Hydrologie und Wasserbewirtschaftung 46(6):256-267 [147]

Großmann J (2005) in Zitat[Anonym (2005b) **[34]** Seiten 45-54 [36]

Großmann J, Poetke D, Nitschke F, Dommaschk M, Drangmeister J, Thear W, Hirsch D (2007) Ergebnisbericht zum Grund- und Oberflächenwassermonitoring im Großraum Bitterfeld/Wolfen. Berichtszeitraum 2005/2006. Ökologisches Großprojekt Bitterfeld/Wolfen, im Auftrag der Landesanstalt für Altlastenfreistellung Sachsen-Anhalt, 31.8.2007, 71 S [76]

Großmann J, Ritter A, Sievers J, Keil M, Bielke A, Weiß H (2005) Entwicklung einer integrierten Managementstrategie (IMS) für Altlastengroßstandorte und Anwendung auf den Modellstandort des Ökologischen Großprojektes Bitterfeld. Altlasten Spektrum 4/2005, 181-186 [31]

Großmann J, Sievers J, Ritter A, Keil M, Weiß H (2005) The Bitterfeld Megasite in Germany – an example for a risk-based management approach. In: Uhlmann O, Annokkée GJ, Arendt F (eds) ConSoil 2005 – Proc 9th International FZK/TNO Conference on Soil-Water Systems, Bordeaux 3–7 October 2005, Theme F, pp 2620–2627 [91]

Grotenhuis T, Smit M, Malina G, Kasperek R, Szdzuj J, Satijn B, Joziasse J (2005) Management scenarios for contamina­ted sediments at megasites. In Uhlmann O, Annokkée GJ, Arendt F (Eds) ConSoil 2005, Proc. 9th Int FZK/TNO Conf on Soil-Water Systems, Bordeaux 3-7 Oct 2005, Theme E, pp. 2513-2522 [90]

Grünewald U (2001) Sanierung des regionalen Wasser- und Stoffhaushaltes. Erarbeitung von Prognosen zur Entwicklung der Wassermenge und –beschaffenheit, Ableitung von Risiken und Handlungsbedarf. In: GEOAgentur Berlin Branden­burg (Hrsg.) in Kooperation mit Forschungs-zentrum Bergbaufolgelandschaften der BTU Cottbus. InfoForum Rekulti­vierung. Dokumentation 6, Nov. 2001, S. 57-66 [146]

Grünewald U (2005) Vorsorge gegenüber Naturrisiken: Nach den Augustfluten 2002 in Mittel- und Zentraleuropa – Hochwasservorsorge in Deutschland. In: Akademie für Raumforschung und Landesplanung. Forschungs- und Sitzungsberichte, Band 223, S. 78-85. Hannover (ISBN 3-88838-052-9) [86]

Grünewald U, Uhlmann W (2004) Zur Entwicklung der Wasserbeschaffenheit in den Lausitzer Tagebauseen – Aus-gangspunkt, Stand und Perspektiven. World of Mining – Surface & Underground 56(2):115-125 [145]

Haack U, Plimer I (1998) Zum Stoffbestand der Kupferschiefer Erzlagerstätten Mansfeld-Hettstedt-Eisleben. Mitt Geol Sachsen-Anhalt 4: 153-162 [160]

Haag I, Kern U, Westrich B (2001), Erosion investigation and sediment quality measurement for a comprehensive risk assessment of contaminated aquatic sediment. Sci Total Environ 266:249-257 [291]

Hammer J (1986) Zur Geochemie ausgewählter Elemente und zu deren Bindungsverhältnissen im Kupferschiefer der Sangerhäuser Mulde. Dissertation, TU-Bergakademie Freiberg [159]

Harbodt K (2006) History until 1945. In: Friedrich HE, Mordike BL (Eds) Magnesium Technology – Metallurgy, Design Data, Applications. 685 p. Springer, Berlin [17]

Heininger P (2009) Maßnahmen zur Reduzierung der Schadstoffbelastung. Ad hoc-AG „Schadstoffe“ der AG OW der FGG Elbe. Begleitung der Anhörung „Bewirtschaftungsplanung im deutschen Einzugsgebiet der Elbe“, Hamburg, 24.04.2009. Folie #29. Bundesanstalt für Gewässerkunde, Koblenz. [33]

Heininger P (2014) Schadstoffbelastung aus dem Oberstrom. Forum Strombau und Sedimentmanagement Tideelbe, Hamburg, 4. April 2014. 48 S. **[98]**

Heininger P, Pelzer J (1998) Trends und Verteilungsmuster in der Schadstoffbelastung von Sedimenten aus östlichen Bun­des­wasserstraßen. Acta hydrochim hydrobiol 26(4) 218 – 225 [251]

Heise S (2013) Durchführung einer Sondierungsuntersuchung zum Risiko durch eine Schadstoffremobilisierung aus Seitenstrukturen der Elbe. Abschlussbericht. Hochschule für Angewandte Wissenschaften Hamburg. 42 S. **[103]**

Heise S, Förstner U (2006) Risks from historical contaminated sediments in the Rhine Basin. Water Air Soil Pollut Focus 6:625-636 [205]

Heise S, Förstner U (2007) Risk assessment on contaminated sediments in river basins – theoretical considerations and pragmatic approach. J Environ Monit 9: 943-952 [283]

Heise S, Claus E, Heininger P, Krämer T, Krüger F, Schwartz R, Förstner U (2005) Studie zur Schadstoffbelastung der Sedimente im Elbeeinzugsgebiet. Ursachen und Trends. Im Auftrag der Hamburg Port Authority, Nov. 2005, 168 S. [42]

Heise S, Förstner U, Westrich B, Jancke T, Karnahl J, Salomons W (eds, 2004) Inventory of Historical Contaminated Sedi­ment in Rhine Basin and its Tributaries. Technical University of Hamburg-Harburg and University of Stuttgart, on behalf of the Port of Rotterdam. October 2004, Hamburg, 225 p. [202]

Heise S, Krüger F, Förstner U, Baborowski M, Götz R, Stachel S (2008) Bewertung der Risiken durch feststoffgebundene Schadstoffe im Elbeeinzugsgebiet. Im Auftrag von Flussgebietsgemeinschaft Elbe und Hamburg Port Authority, Mai 2008, 349 S. [43]

Hellmann H (1970) Die Charakterisierung von Sedimenten auf Grund ihres Gehaltes an Spurenmetallen. Dt Gewässer­kundl. Mitt 14: 160-164 [219]

Hille J, Ruske R, Scholz RW, Walkow F (Hrsg, 1992) Bitterfeld – modellhafte ökologische Bestandsaufnahme einer konta­minierten Industrieregion. Erich Schmidt Verlag Berlin [263]

Hillebrand G, Claus E (2013) Untersuchung belasteter Sedimente in Buhnenfeldern der Elbe und Staustufen der Saale. Abschlussworkshop. [102]

Hollert H, Brinkmann M, Hudjetz S, Cofalla C, Schüttrumpf H (2014) Hochwasser – ein unterschätztes Risiko (Schadstof­fe als „Zeitbomben“ im Sediment). Biologie in unserer Zeit 1/2014, S. 44-51 [300]

Hollert H, Dürr M, Erdinger L, Braunbeck T (2000) Cytotoxicity of settling particulate matter (SPM) and sediments of the Neckar river (Germany) during a winter flood. Environ Toxicol Chem 19:528-534 [233]

Hong J (1995) Characteristics and Mobilization of Heavy Metals in Anoxic Sediments of the Elbe River during Resuspen­sion/Oxidation. Dissertation an der Technischen Universität Hamburg-Harburg, 157 S.[244]

Hoth N, Rammlmair D, Gerth J, Häfner F (2008) Leitfaden “Natürliche Schadstoffminderungs-prozesse an großräumigen ergbaukippen/-halden und Flussauensedimenten. Empfehlungen zur Untersuchung und Bewertung der natürlichen Quelltermminimierung. 124 Seiten + Anlage 1 (Förstner U: „Erkenntnisse zu Monitored Natural Recovery an Flusssedimenten“, 19 S.). BMBF-Förderschwerpunkt „Kontrollierter natürlicher Rückhalt und Abbau von Schadstoffe bei der Sanierung kontaminierter Grundwasser und Böden“ (KORA), TU Bergakademie Freiberg und DECHEMA, Freiberg/Frankfurt, November 2008 [29]

Jacobs PH (2002) A new rechargeable dialysis sampler for monitoring subaqeus in-situ sediment caps. Wat Res 36 (13): 3121-3129 [167]

Jacobs PH (2003) Kontaminierte Sedimente und Baggergut: Aktive Barriere-Systeme für die Subaquatische Lage-rung und Abdeckung. Dissertation an der Technischen Universität Hamburg-Harburg. 3. Dezember 2003. 198 S. [166]

Jacobs P (2013) Frachtreduktion Spittelwasser – Machbarkeitsstudie im Auftrag der Landesanstalt für Altlastenfreistellung Sachsen-Anhalt. Folienserie anl. Workshop „Sedimentmanagement der FGG Elbe“, Hamburg, 17. Dezember 2013. Tauw GmbH NL Berlin [95]

Jacobs PH, Förstner U (1999) Concept of subaqueous in-situ capping of contaminated sediments with active barrier sys­tems (ABS) using natural and modified zeolites. Wat Res 33(9):2083-2087 [168]

Jacobs P, Förstner U (2001) Managing contaminated sediments. IV: Subaqueous storage and capping of dredged material. J Soils Sediments 1(4):205-212 [169]

Jacobs PH, Förstner U (2003) Gewässersedimente und Baggergut. In: Förstner U, Grathwohl P (eds) Ingenieurgeochemie – Natür­licher Abbau und Rückhalt, Stabilisierung von Massenabfällen. S. 330-360. Springer Verlag Berlin [170]

Jacobs PH, Waite TD (2003) The role of aqueous iron and manganse in subaqueous active barrier systems containing ntural clinoptilolite. Chemosphere 54(3)313-324 [172]

Jacobs P, Krautter N, Diesner K (2013) Frachtreduzierung Spittelwasser – Abschlussbericht. Oberflächenwasser-, Sediment- und Auenuntersuchungen; Bewertung und Ableitung von Maßnahmeerfordernissen. Projektleitung: Dr. Patrick Jacobs, Tauw GmbH, Berlin; 21. Oktober 2013. Im Auftrag der Landesanstalt für Altlastenfreistellung des Landes Sachsen-Anhalt. Berlin, 232 Seiten **[16]**

Jacobs PH, Förstner U, Prestel H, Nießner R (2001) On-site Porenwasser-Probenahme und Schwermetall-Analytik mittels Kopplung von Dialyseprobenahme und laserinduzierter Fluoreszensspektroskopie: Feldstudie Vollert-Süd. Wasser­chemische Gesellschaft, Jahrestagung 2001 Bad Wildungen, 21.-23.5.2001. S. 455-461. Karlsruhe [171]

Jafvert CT, Rogers JE (1991) Biological remediation of contaminated sediments with special emphasis on the Great Lakes : report of a workshop, Manitowoc, Wisconsin, July 17-19, 1990, Environmental Research Laboratory, Office of Research and Development, U.S. Environmental Protection Agency, 181 p, Athens, Ga. [112]

Jahnke A, McLeod M, Wickström H, Mayer Ph* (2014) Equilibrium sampling to determine the thermodynamic potential for bioaccumulation of persistent organic pollutants from sediment. Environ Sci Technol 48(19)11352-11359 [190]

Jones AM (2014) Superfund cleanup in the Hudson River: Where are we? An update through the 2014 season. Folienserie, 9 Folien. Hudson River PCB Forum, Marist College, Cornell Boathouse, November 11, 2014 **[131]**

Joziasse J, van der Gun J (2000) In situ remediation of contaminated sediments: Conceivable and feasible? In: Contamina­ted Soil 2000, Vol I, pp. 516-522. Thomas Telford, London [275]

Junge FW (2013) Schadstoffsenke Muldestausee – Aktuelles Potenzial und jüngste Entwicklung seit 2002. Abschluss­bericht Fa. Junge-Erdwissen i.R. des Projekts ELSA der Freien und Hansestadt Hamburg, Taucha, den 06.11.2013. 95 S. **[104]**

Kasimir P, Claus E (2013) Bestandsaufnahme belasteter Altsedimente in der Saale. Vom Wasser 111(4)145-146 [105]

Kausch H, Michaelis W (Hrsg, 1996) Suspended Particulate Matter in Rivers and Estuaries. Advances in Limnology 47, 573 p, Schweizerbart’sche Verlagsbuchhandlung Stuttgart [235]

Keil M, Großmann J, Weiß H (2011) Comment on „Sediment research, management and policy – a decade of JSS. J Soils Sediments 11:542 [81]

Kern U, Westrich B (Bearbeiter, 1999) Methoden zur Erkundung, Untersuchung und Bewertung von Sedimentablagerun­gen und Schwebstoffen in Gewässern. Hrsg. vom Arbeitskreis „Schweb- und Schadstoffe in Fließgewässern“ (Leiter: U. Förstner) des Deutschen Verbands für Wasserwirtschaft und Kulturbau e.V. (DVWK, jetzt: DWA), DVWK-Schrif­ten Nr. 128, 418 S. [175]

Klapper H (2002) Mining lakes: Generation, loading and water quality control. In: Mudroch A, Stottmeister U, Kennedy C, Klapper H. (Eds) Remediation of Abandoned Surface Coal Mining Sites. pp 57-110. Springer Verlag Heidelberg [152]

Klapper H, Friese K, Scharf B, Schimmele M, Schultze M (1998) Ways of controlling acid by ecotechnology. In: Geller W, Klapper H, Salomons W (Eds.) Acid Mine Lakes – Acid Mine Drainage, Limnology and Reclamation. Chapter 22, pp. 401-416. Springer Verlag Heidelberg [151]

Klemm W, Greif A, Knittel U (2004) AP 3.7: Schwermetall- und Arsenverlagerung in der Freiberger und Zwickauer Mulde. In: Geller W et al. (Hrsg.) Schadstoffbelastung nach dem Elbe-Hochwasser 2002. Endbericht des Ad-hoc Ver-bundprojekts, S. 159-172 [164]

Kluge A (1994) Bestandsaufnahme und Schwermetallbelastung in Wasser und Sediment der Mulde in den Jahren 1991-1993, in Guhr H, Prange A, Puncochar P, Wilken R -D, Büttner B (Hrsg) Die Elbe im Spannungsfeld zwischen Öko­logie und Ökonomie. 6. Magdeburger Gewässerschutzseminar, S. 78 - 83, B. G. Teubner Verlagsgesellschaft Stuttgart Leipzig [247]

Kofod, M. (1994) Die Bedeutung frühdiagenetischer Prozesse für die Porenwasserzusammensetzung in anaeroben Bagger­schlämmen. Dissertation Universität Hamburg 1994. Hamburger Bodenkundliche Arbeiten Band 28 [262]

Köhler HJ, Krapp L, Braun M (1992) Konzepte und Handlungsprioritäten zur Siche-rung und Sanierung von Altablage­run­gen, Deponien und Grundwasser im Großraum Bitterfeld/Wolfen. In: Hille J, Ruske R, Scholz RW, Walkow F (Hrsg) Bitterfeld – modellhafte ökologische Bestandsaufnahme einer kontaminierten Industrieregion. S. 203-210. Erich Schmidt Verlag Berlin [265]

Krapp L, Ruske R (1992) Geologische Verhältnisse von Bitterfeld und ihre Relevanz zu Kontaminationen des Grund-wassers und Bodens. In: Hille J, Ruske R, Scholz R, Walkow F (Hrsg) Bitterfeld – modellhafte ökologische Bestands-aufnahme einer kontaminierten Industrieregion. S. 85-92. Erich Schmidt Verlag Berlin [264]

Krüger F, Scholz M, Kreibich M, Baborowski M (2014) Sedimentrückhalt in den Elbauen. Studie zur Erarbeitung des Sedimentrückhaltes in Auen als Teilfunktion des Sedimenttransportgeschehens an der Elbe. Abschlussbericht im Rah-men von „Schadstoffsanierung Elbesedimente – ELSA“, Behörde für Stadtentwicklung und Umwelt in Hamburg, 73 S. **[101]**

Kupryianchyk D, Rakowska MI, Reible D, Harmsen J, Cornelissen G, van Veggel M, Hale SE, Grotenhuis T, Koelmans AA (2015) Positioning activated carbon amendment technologies in a novel framework for sediment manage-ment. Integr Environ Assess Manag 11(2)221-234 [182]

Lake I, Foxll C, Lovett A, Fernandes A, Dowding A, White S, Rose M (2005) Effects of river flooding on PCDD/F and PCB levels in cow’s milk, soil, and grass. Environ Sci Technol 39(23) 9033-9038 [126]

Lampert DJ, Thomas G, Reible DD* (2015) Internal and external transport significance for predicting contaminant uptake rates in passive samplers. Chemosphere 119:910-916 [194]

Lersner H v (1985) Rechtliche Instrumente der Umweltpolitik. In: Jänicke M, Simonis UE, Weigmann G (Hrsg.) Wissen für die Umwelt. S. 195-214. Walter de Gruyter Verlag Berlin [84]

Lexartza-Artza I, Lerner D, Tkachenko N (2009) Preliminary study of the Doe Lea catchment for the Doe Lea Project, Catchment Science Centre, The University of Sheffield, August 2009, 48 p [127]

Lindemann M (2000) Das ökologische Großprojekt Bitterfeld-Wolfen – eine Bilanz aus fachlicher Sicht. Altlasten Spektrum 4/2000, S. 208-214 [8]

Lindemann M, Wittmann U (2000) Fallstudie Spittelwasser in der Region Bitterfeld. Eine Altlast – vier Konzepts: Ein Nachtrag. TerraTech 6/2000, S. 20-21 [12]

Lowe S, Abood K, Ko J, Wakeman T (2005) A sediment budget analysis of Newark Bay. The Technical Proceedings oft he IMarEstT, Part C3. J Mar Sci Environ, August 2005. [135] In: Garvey et al. (2007) [134]

Luckner L (2006a) Lausitz: Sanierung einer Bergbaufolgelandschaft. Teil 1. Die Lösung des Wassermengenproblems. WWT 3/2006, S. 33-37 [149]

Luckner L (2006b) Lausitz: Sanierung einer Bergbaufolgelandschaft. Teil 2. Das Problem mit der Wasserqualität. Die Neutralisation der sauren Restseen. WWT 4/2006, S. 10-16 [150]

Lydy MJ, Landrum PJ*, Oen AMP, Allinson M, Smedes F, Harwood AD, Li H, Maruya KA, Liu J (2014) Passive samp­ling methods for contaminated sediments: state of the science for organic contaminants. Integr Environ Assess Manag 10(2)167-178 [196]

Maack B (2011) Dioxin-Skandale – Das Überall-Gift. Spiegel-Online vom 05.01.2011 **[60]**

Magar VS, Wenning RJ (2006) The role of natural recovery in sediment remediation. Integrated Envir Assessment and Management 2:66-74 [173]

Medek J et al. (2014) SedBiLa – Bedeutung der Bilina als historische und aktuelle Schadstoffquelle für das Sedimentmana­gement im Einzugsgebiet der Elbe. Studie im Auftrag der Freien und Hansestadt Hamburg. 80 S. **[216]**

Michels J, Stuhrmann M, Frey C, Koschitzky H-P (Hrsg., 2008) Handlungsempfehlungen mit Methodensammlung. Natürliche Schadstoffminderung bei der Sanierung von Altlasten. VEGAS, Institut für Wasserbau, Universität Stutt-gart, DECHEMA e.V. Frankfurt. ISBN-13 978-3-89746-092-0. 363 Seiten [27]

Miehlich G (1987) Substratgenese und Systematik von Böden der Hamburger Flussmarsch. Mitt Dtsch Bodenkdl Ges 55/II: 801-803 [256]

Miehlich G (1994) Auen und Marschen als Senke für belastete Sedimente der Elbe. In: Guhr, H. et al. (Hrsg) Die Elbe im Spannungsfeld zwischen Ökologie und Ökonomie. S. 307-312. Teubner Verlag Leipzig [258]

Milch W (2005) „Altlastensanierung in Sachsen-Anhalt unter Berücksichtigung der Wasser¬rahmenrichtlinie und der neuen Wassergesetzgebung des Landes“. In: Zitat [Anonym (2005b)**[34]**, Seiten 3-12 [38]

Möbius J (2002) Einrichtung und Überwachung eines Testfelds zur Abdeckung kontaminierten Baggerguts mit aktiven Barriere-Systemen, Sportboothafen Hitzacker. Antrag auf Förderung einer Forschungs- und Entwicklungsmaßnahme. Fa. Josef Möbius Bau-Aktiengesellschaft Hamburg (mit Arbeitsbereich Umweltschutztechnik der TUHH), 41 Seiten, Hamburg 2002 [82]

Mudroch A, Burbonniere RA (1994) Sediment preservation, processing, and storage. In: Mudroch A, MacKnight SD (eds) Technologies for Aquatic Sediments Sampling, 2nd ed, pp 131-169. Lewis Publ, Boca Raton FL [288]

Müller A Wessels M (1999) The flood in the Odra River 1997 – Impact of suspended solids on water quality. Acta hydro­chim hydrobiol 27:316-320 [254]

Müller A, Hanisch C, Zerling L, Lohse M, Walther A (1998) Schwermetalle im Gewässersystem der Weißen Elster – natür­liche und anthropogene Elementverteilung im Sediment, im Schwebstoff und in der gelösten Phase. Abh Sächs Akademie der Wissenschaften zu Leipzig. Mathematisch -naturwissenschaftliche Klasse. Band 58, Heft 6, 199 S., Leipzig [253]

Müller G, Furrer R (1994) Belastungspotential Elbenebenflüsse. Schwermetalle in den Sedimenten der Elbe und ihrer Zuflüss,. in: Guhr H, Prange A, Puncochar P, Wilken R-D, Büttner B (Hrsg) Die Elbe im Spannungsfeld zwischen Ökologie und Ökonomie. 6. Magdeburger Gewässerschutzseminar, S. 69 - 77, B.G. Teubner Verlagsgesellschaft Stuttgart Leipzig [248]

Müller G, Yahia A (1992) Schadstoffbelastung in Böden von Hochwasserüberflutungsflächen des Rheins. Literaturstudie der Universität Heidelberg im Auftrag des Landesamtes für Wasserwirtschaft Rheinland-Pfalz [255]

Murray LA, Breedveld GD (2011) River engineering and sediment management concept for the tidal Elbe River. Task 2: Evaluaion of handling contaminated sediments from the perspective of ecology and economic efficiency [214]

Arup O et al. (1995) River Doe Lea restoration study - National River Authority. Ove Arup & Partners: Leeds, UK. [124]

Netzband A et al. (2007) Sediment management: an essential element of River Basin Management Plans. J Soils Sediments 7(2)117-132 [99]

Palermo MR (2001) A state of the art overview of contaminated sediment remediation in the United States. In: Inter­national Conference on Remediation of Contaminated Sediments, 10-12 October 2001, Venice, Italy. 10 p. [115]

Patmont C, Ghosh U, LaRosa P, Menzie C, Luthy R, Greenberg M, Cornelissen G, Eek E, Collins J, Hull J, Hjartland T, Glaza E, Bleiler J, Quadrini J (2015) In situ sediment treatment using activated carbon: A demonstrated sediment cleanup technology. Integr Environ Assess Manag 11(2)195-207 [183]

Paul M (2003) Geochemische In-situ-Stabilisierung von Bergbaualtlasten. In: Förstner U, Grathwohl P Ingenieur-geochemie – Natürlicher Abbau und Rückhalt, Stabilisierung von Massenabfällen. S. 298-329. Springer Berlin [156]

Pedersen TF, McNee JJ, Flather DH, Mueller B, Pelletier CA (1998) Geochemical behaviour of submerged pyrite-rich tailings in Canadian lakes. In: Geller W, Klapper H, Salomons W (Eds.) Acid Mine Lakes – Acid Mine Drainage, Limnology and Reclamation. Chapter 6, pp 87-125. Springer Heidelberg [155]

Peijnenburg WJGM*, Teasdale PR, Reible D, Mondon J, Bennett WW, Campbell PGC* (2014) Passive sampling methods for contaminated sediments: State of the science for metals. Integr Environ Assess Manag 10(2)179-196 [195]

Pepelnik R, Niedergesäß R, Erbslöh B, Aulinger A, Prange A (2004) AP 3.2: Längsprofiluntersuchungen zur Beurteilung von Auswirkungen des Hochwassers vom August 2002 auf die Wasser- und Sedimentqualität der Elbe. In: Geller W. et al. (Hrsg.) Schadstoffbelastung nach dem Elbe-Hochwasser 2002. Endbericht des Ad-hoc-Verbundprojekts, S. 82-100 [270]

Peschel H (2009) Regionale Schwerpunkte der Maßnahmenplanung in Sachsen-Anhalt. Informationsveranstaltung zur Bewirtschaftungsplanung im deutschen Einzugsgebiet der Elbe. 23.04.2009 [54]

Poetke D, Nitschke F, Hirsch D. (2007) Belastungen in Schachtgraben und Spittelwasser sowie in Mulde und Elbe. Sachstandsbericht zu den Untersuchungen des Jahres 2007 am RRB D02. Stand Oktober 2007. Ökologisches Großprojekt Bitterfeld/Wolfen., im Auftrag der Landesanstalt für Altlastenfreistellung Sachsen-Anhalt, 30.10.2007, 18 Seiten, GICON Dresden (unveröffentlicht) [77]

Prange A et al. (1997) Erfassung und Beurteilung der Belastung der Elbe mit Schadstoffen. Abschlußbericht zum BMBF - Forschungsvorhaben: 02-WT 9355/4, Band 1-3, GKSS-Forschungszentrum Geesthacht [252]

Quevauviller P (2004) Traceability of environmental chemicl measurements. Trends Anal Chem 23:171-177 [292]

Quevauviller Ph (ed, 2002) Methodologies for Soil and Sediment Fractionation Studies, The Royal Society of Chemistry, Cambridge, UK [290]

Rakowska MI, Kupryianchyk D, Harmsen J, Grotenhuis JTC, Koelmans AA* (2012). In situ remediation of contaminated sediments using carbonaceous materials. Environ Toxicol Chem 31:693–704 [187]

Reichel F, Uhlmann W (1995) Möglichkeiten und Grenzen der Beeinflussung der Wasser-beschaffenheit in Tagebaurest­löchern bei aufsteigendem Grundwasser am Beispiel der Lausitzer Bergbaufolgelandschaft. In: Proc 4. Dresdener Grundwasserforschungstage, Coswig, 24./25. Oktober 1995, Band II, S. 39-51 [153]

Rijnaarts H, ter Meer J (2005) The WELCOME Integral Management Strategy for large scale historical soil and ground­water contamination. TNO, Knowledge for Business, NATO-CCMS, June 2005, Ottawa. 23 Folien **[30]**

Rijnaarts HHM (2005) in Zitat [Anonym (2005b)[34] Seiten 55-64 [37]

Rudis M, Trejtnar K (1996) Sedimente in den Staubecken des tschechischen Elbeabschnitts, in: Prange A, Wilken R-D, von Tümpling U, Spoustova J, Punchochar P, Lencova E (Hrsg.) Ökosystem Elbe - Zustand, Entwicklung und Nutzung. 7. Magdeburger Gewässerschutzseminar, Tagungsband, S. 177 – 182 [249]

Rulkens WH (2001) An overview of soil and sediment treatment research in the Netherlands. In Stegmann R. et al. (eds) Treatment of Contaminated Soil: Fundamentals, Analysis, Applications, pp. 21-34, Springer-Verlag, Berlin [87]

Salomons W (1995) Long-term strategies for handling contaminated sites and large-scale areas. In: Salomons W, Stigliani WM (Eds) Biogeodynamic of pollutants in soils and sediments – risk assessment of delayed and non-linear responses; pp 1-30. Springer Berlin [240]

Salomons W (2005) Sediments in the catchment-coast continuum. J Soils Sediments 5(1):2-8 [198]

Salomons W, Brils J (Eds, 2004) Contaminated sediments in European River Basins. European Sediment Research Network SedNet, EC Contract No. EVKI-CT-2001-0002. Key Action 1.4.1 Abatement of water pollution from con-taminated land, landfills and sediments. TNO Den Helder/The Netherlands, 80 p. [97]

SAW (eds 2000) Schadstoffdynamik in Einzugsgebieten. Projekt III.B.09 (1999-2002). Sächsische Akademie der Wissenschaften zu Leipzig. Informationen zur Öffentlichen Frühjahrssitzung am 14. April 2000 in Leipzig [260]

Scharff H (2010) Die nachhaltige Deponie – Strategien zur Stilllegung und Nachsorge in den Niederlanden. Deponie-technik 35, 13 Seiten (Hamburg, Februar 2010). Verlag Abfall aktuell [74]

Schipper C, Schout P (2003) De weg naar implementatie von de Chemie-Toxiciteit-Toets. Werkdocumnt: RIKZ/-2003.036. AKWA/RIKZ 04.005, ISBN: 36934761, 82 p. [199]

Schleichert U (1975) Schwermetallgehalte der Schwebstoffe des Rheins bei Koblenz im Jahresablauf. Dt Gewässerkd Mitt 19:150-157 [232]

Scholz RW, Nothbaum N, May TW, Brockmann R, Bode H, Deubel K-H, Hippe U (1992) Klassifikation CKW-kon-tami­nierter Flächen im Überschwemmungsgebiet der Mulde und Elbe. In: Hille J, Ruske R, Scholz RW, Walkow F (Hrsg.) Bitterfeld – modellhafte ökologische Bestandsaufnahme einer kontaminierten Industrieregion. S. 171-182. Erich Schmidt Verlag Berlin [266]

Schönberger H (2004) HCB in the River Rhine. In:[202] Chapter 4.6, pp 138-145. Technical University of Hamburg-Har­burg and University of Stuttgart, on behalf of the Port of Rotterdam. October 2004, Hamburg [203]

Schreck P, Wennrich R, Stärk MJ, Schubert M, Weiß H (2004) Mansfeld – the contribution of a mining-affected catchment area to regional riverine pollution. UFZ-Bericht 18/2004, UFZ Leipzig-Halle GmbH, Leipzig, pp. 169-170 [163]

Schuster J, Miehlich G (1989) Tideabhängige Konzentrationsveränderungen in Prielwässern als Ausdruck von Austausch­vorgängen zwischen Vordeichsland und Elbeästuar, Mitt Dtsch Bodenkdl Ges 59/I: 483-488 [257]

Schüttrumpf H, Brinkmann M, Cofalla C, Frings RM, Gerbersdorf SU, Hecker M, Hudjetz S, Kammann U, Lennartz G, Roger S, Schäffer A, Hollert H (2011) A new approach to investigate the interactions between sediment transport and ecotoxicological processes during flood events. Environ Sciences Europe 23:39 [298]

Schwartz R, Gerth J, Neumann-Hensel H, Bley S, Förstner U (2006) Assessment of highly polluted fluvisol in the Spittelwasser floodplain, based on national guideline values and MNR-criteria. J Soils Sediments 6(3):145-155 [177]

Schwartz R, Kozerski HP (2004) AP 4.2: Bestimmung des Gefahrenpotentials feinkörniger Buhnenfeldsedimente für die Wasser- und Schwebstoffqualität der Elbe sowie den Stoffeintrag in Auen. In: Geller W. et al. (Hrsg.) Schadstoff-belastung nach dem Elbe-Hochwasser 2002. Endbericht des Ad-hoc-Verbundprojekts, S. 258-274 [269]

Shea D (1988) Developing national sediment quality criteria. Environ Sci Technol 22:11256-1261 [141]

Smit MPJ, Grotenhuis T, Bruning H, Rulkens WH (2008) Desorption of dieldrin from field aged sediments: Simulating flood events. J Soils Sediments 8:80-85 [294]

Stachel B, Götz R (2008) Die Kontamination von Elbaueböden, Elbefischen sowie Futter- and Lebensmitteln mit Dioxinen und dioxin-ähnlichen PCB, Anlage 13 zur HPA-Riskostudie, 28. Mai 2008, 26 S. [48]

Stachel B, Christoph EH, Götz R, Herrmann T, Krüger F, Kühn T, Lay J, Löffler J, Päpke O, Reincke H, Schröter-Kermani C, Schwartz R, Steeg E, Stehr D, Uhlig S, Umlauf G (2006): Contamination of the alluvial plain, feeding-stuffs and foodstuffs with polychlorinated dibenzo-p-dioxins, polychlorinated dibenzofurans (PCDD/Fs), dioxin-like polychlori­na­ted biphenyls (DL-PCBs) and mercury from the River Elbe in the light of the flood event in August 2002. Science Total Environ 364:96-112 [49]

Stachel B, Christoph EH, Götz R, Herrmann T, Krüger F, Kühn T, Lay J, Löffler J, Päpke O, Reincke H, Schröter-Kermani C, Schwartz R, Steeg E, Stehr D, Uhlig S, Umlauf G (2007): Dioxins and dioxin-like PCBs in different fish from the river Elbe and its tributaries, Germany. J Hazard Mat 148:199-209 [50]

Stachel B, Götz R, Mariani G, Umlauf G (2010) Dioxine und PCBs in Feststoffen aus der Elbe, ihren Nebenflüssen und der Nordsee (Längsprofilaufnahme 2008, Kurzfassung), in: Anonym (Bearbeiter: Bergemann, M, Gaumert T) Elbebericht 2008 - Ergebnisse des nationalen Überwachungsprogramms Elbe der Bundesländer über den ökologischen und chemischen Zustand der Elbe nach EG-WRRL sowie der Trendentwicklung von Stoffen und Schadstoffgruppen. Sonderkapitel 5.4, S. 64-70. Flussgebietsgemeinschaft Elbe, Mai 2010, Magdeburg [52]

Stachel B, Mariani G, Umlauf G, Götz R (2011) Dioxine und PCBs in Feststoffen aus der Elbe, ihren Nebenflüssen und der Nordsee (Längsprofilaufnahme 2008) mit Unterstützung der Flussgebietsgemeinschaft Elbe, Sept. 2011. FGG Elbe, Redaktion: Frank Krüger [57]

Stevens M (2011) Why is the water quality in the River Doe Lea so poor? Dissertation for the Degree of Master of Science, University of Sheffield, Department of Civil & Structural Engineering, Septmber 2011, 132 p. Including Appendix C: River sediment monitoring and dioxin analysis in the Doe Lea: A literature survey of Environment Agency studies between 1991 and 1998 [121]

Stigliani WM (1988) Changes in valued capacities of soils and sediments as indicators of nonlinear and time-delayed environmental effects. Environ Monitoring Assess 10:245-307 [236]

Stigliani WM (1991) Chemical time bombs: definition, concepts, and examples. Executive report 16 (CTB basic docu­ment). IIASA Laxenburg, Austria, 23 p. [237]

Stigliani WM (1995) Global perspectives and risk assessment. In: Salomons W, Stigliani WM (Eds) Biogeodynamic of pollutants in soils and sediments – risk assessment of delayed and non-linear responses; pp 331-343, Springer Berlin [243]

Stronkhorst J (2003) Ecotoxicological effects of Dutch harbour sediments. PhD Thesis, Free University of Amsterdam [204]

Thomas R, Meybeck M (1992) The use of particulate material, in: Chapman D (ed) Water Quality Assessment, A Guide to the Use of Biota, Sediments and Water in Environmental Monitoring, Chap 4, pp 121-170, Chapman & Hall, Lon­don [278]

Thompson JM, Hsieh C, Luthy RG* (2015) Modeling uptake of hydrophobic organic contaminants into polyethylene passive sampler. Environ Sci Technol. 49(4):2270-2277 [193]

Uhlig S, Simon K, Kunath K (2008) Dioxine und Furane in Elbe, Mulde und Spittelwasser: Statistische Analyse der Kongenerenmuster. Quo data, Gesellschaft für Qualitätsmanagement und Statistik mbH, Anl. 1 zur HPA-Risikostudie, 28.05.2008, 60 S. [47]

Umlauf G, Stachel B, Mariani G, Götz R (2011) Dioxins and PCBs in solid matter from the River Elbe, its tributaries and the North Sea (longitudinal profile, 2008). EUR – Scientific and Technical Research series – ISSN 1018-5593 (print), 2011, 118 p. [56]

van Noort PCM, Koelmans AA (2012) Nonequilibrium of organic compounds in sediment-water systems. Consequences for risk assessment and remediation measures. Environ Sci Technol 46:10900-10908 [180]

Vellinga T (2004) From dredged material management to sediment management. J Soils Sediments 4(4)215 [96]

Verta M, Kiviranta H, Salo S, Malve O, Korhonen M, Verkasalo PK, Ruokojärvi P, Rossi E, Hanski A, Päätalo K, Vartiai­nen T. (2009) A decision framework for possible remediation of contaminated sediments in the River Kymijoki, Fin­land. Environ Sci Pollut Res Int. 16(1):95-105 [128]

Walkow F (1996) The dioxin pollution of Bitterfeld. Lecture held at the meeting „Chemistry, Man and Environment – 20th Anniversary of the Seveso Accident“, Milan, October 2000 [267]

Weber R, Gaus C, Tysklind M, Johnston P, Forter M, Hollert H, Heinisch E, Holoubek I, Lloyd-Smith M, Masunaga S, Mocarelli P, Santillo D, Seike N, Symons R, Torres JPM, Verta M, Varbelow G, Vijgen J, Watson A, Costner P, Woelz P, Wycisk P, Zenneg M (2008) Dioxin- and POP-contaminated sites—contemporary and future relevance and challenges. Environ Sci Pollut Res 15:363–393 [129]

Wenzel C (2012) Die EG-Meeresstrategie-Rahmenrichtlinie (MSRL) – Bedeutung für die Bewertung von Sediment-kontaminationen. In: Magdeburger Gewässerschutzseminar 2012 „Die Elbe und ihre Sedimente“. Hamburg 10./11. Oktober 2012, S. 72-76. 15 Folien **[15]**

Wernicke P (2005) in Zitat [Anonym (2005b)**[ 34]** Seiten I-III [35]

Werther J, Dreuscher H, Hilligardt R (1984) Aufstromklassierung und maschinelle Entwässerung des Hamburger Hafen­schlicks. Veröff. Fachseminar Baggergut, Strom- und Hafenbau, Wirtschaftbehörde der Freien und Hansestadt Hamburg, 27.02.-01.03.1984. S. 183-202 [109]

Westrich B (1988) Fluvialer Feststofftransport – Auswirkung auf die Morphologie und Bedeutung für die Gewässergüte. SchrR Wasser-Abwasser Bd 22, 173 S. Oldenbourg Verlag München [230]

Westrich B (1988) Hydromechanische Einflussfaktoren auf das Transportverhalten kontaminierter Schwebstoffe in Flüs­sen. DVWK Mitteilungen, Heft 9. Bonn [224]

Westrich B (2007) Sustainable sediment management. In: Westrich B, Förstner U (eds) Sediment Dynamic and Pollutant Mobility in Rivers. An Interdisciplinary Approach. Chap. 2.1, pp 35-49. Springer, Berlin [201]

Westrich B, Förstner U (eds 2007) Sediment Dynamics and Pollutant Mobility in Rivers – An Interdisciplinary Approach. Springer, Berlin [277]

Westrich B, Haag I, Kern U (2000) Mobilität von Schadstoffen in den Sedimenten staugeregelter Flüsse – Dynamik und Bilanzierung von Schwebstoffen und Schwermetallen in einer Stauhaltungskette, Forschungsbericht FZKA-BWPLUS. Fzk: PW 96 182 [231]

Wieprecht S et al. (2013) Ermittlung des Remobilisierungspotentials belasteter Altsedimente in ausgewählten Gewäs-sern Sachsen-Anhalts. Institut für Wasser- und Umweltsystemmodellierung (IWS). Lehrstuhl für Wasserbau und Wassermengenwirtschaft der Universität Stuttgart, im Auftrag von: Landesbetrieb für Hochwasserschutz und Wasser-wirtschaft Sachsen-Anhalt; Stuttgart, den 11.06.2013; 153 S. **[107]**

Witt O (2004) Erosionsstabilität von Gewässersedimenten mit Auswirkungen auf den Stofftransport bei Hochwasser am Beispiel ausgewählter Staustufen am Oberrhein. Institut für Wasserbau, Universität Stuttgart, Mitteilungen H 127 [274]

Witt O, Westrich B (2003) Quantification of erosion rates for undisturbed cohesive sediment cores by image analysis. Hydro­biologia 494(1-3)271-276 [273]

Wolf K (1986) The Hamburg-Georgswerder dumping ground – situation, problems and administrative arrangements for producing a rehabilitation plan. In: Assink JW, Van den Brink WJ (eds) Contaminated Soil‚ pp 723-728. Martinus Nijhoff Dordrecht [62]

Wölz J, Fleig M, Schulze T, Maletz S, v Varel UL,Reifferscheid G, Kühlers D, Braunbeck T, Brack W, Hollert H (2010): Impact of contaminants bound to suspended particulate matter in the context of flood events. J Soils Sediments 10: 1174-1185 [296]

Zerling L, Müller A, Jendryschik K, Hanisch C, Arnold A (2001) Der Bitterfelder Muldestausee als Schadstoffsenke – Entwicklung der Schwermetallbelastung 1992 bis 1997. Abhandlungen der Sächsischen Akademie der Wissen­schaften zu Leipzig 59(4): 69 S. [157]

Zschiedrich K, Benthaus F-C (2005): Bergbausanierung – bergbauliche und wasserwirtschaftliche Aufgaben. Fachtagung zur Nachsorge betriebsbedingter Boden- und Grundwasserschäden des Bergbaus nach der endgültigen Betriebsstill­legung. Proc. DGFZ, ISSN 1430-0176; Heft 27, S. 94-113. Dresden [148]

1. In einem Interview zum 10jährigen Bestehen der Landesanstalt für Altlastenfreistellung des Landes Sachsen-Anhalt stellte deren Geschäftsführer am 01.12.2009 fest, dass trotz der getätigten Ausgaben von insgesamt 950 Millionen € erst 170 Millionen € verbraucht worden waren [↑](#footnote-ref-1)
2. Entnahme- und Transportverfahren: Josef Möbius Baugesellschaft, RBS Straßenbau, Städtereinigung Holtmeyer und TUSEK-Bau; Behandlungsverfahren: Harbauer und Otto Oeko-Tech; Gesamtkonzepte: Bilfinger + Berger Umweltbau, H.R.E. Hanseatische Reinigungs- und Entsorgungsgesellschaft, IWT Ingenieurgesellschaft Wasser- und Tiefbau Kluge Umweltschutz, KONTEC Nassbaggertechnik, Rethmann Sanierungsdienste, SWE Entsorgungsbetriebe KG, WESTAB Holding und Züblin Umwelttechnik. [↑](#footnote-ref-2)
3. Es war anlässlich der Präsentation des deutschen Beitrags zum Fallstudienvergleich die Meinung geäußert worden, die Grundwasserfragen hätten stärker einbezogen werden müssen. Dem steht die Feststellung der STAU-Mitarbeiterin Martina Lindemann entgegen [11]: „Die Untersuchungen der konkreten hydrogeologischen/hydrologischen Bedingungen in Bitter­feld im Rahmen des ökologischen Großprojekts Bitterfeld-Wolfen führten zu der Einschätzung, dass kurzfristig eine Be­einflussung der Oberflächengewässer durch kontaminiertes Bitterfelder Grundwasser nicht erfolgt. Somit ist es auch nicht erforderlich, die Beurteilung der großräumigen Grundwassersanierung in die Sanierungskonzepte für das Spittelwasser einzubeziehen“. Speziell für Dioxin gilt, dass wegen der extrem geringen Wasserlöslichkeit dieser Substanzen ein Aus­tausch zwischen Grund- und Oberflächenwässern praktisch keine Rolle spielt. [↑](#footnote-ref-3)
4. Keller I (2009) Verschneidung von EG-Wasserrahmenrichtlinie und Meeresstrategie-Rahmenrichtlinie. Überregionales Bewirtschaftungsziel Schadstoffe in der Flussgebietsgemeinschaft Elbe. Im Auftrag der Behörde für Stadtentwicklung und Umwelt der Freien und Hansestadt Hamburg, Abteilung Gewässerschutz, Hamburg, Juni 2009, 46 S. [↑](#footnote-ref-4)
5. Stachel B, Götz R (2008) „Die Kontamination von Elbaueböden, Elbefischen sowie Futter- and Lebensmitteln mit Dioxi­nen und dioxin-ähnlichen PCB“, Anlage 13 zur HPA-Riskostudie^5^, Mai 2008, 26 S. [http://www.tideelbe.de/­files/anlage­_­13_-_­son­derkapitel_dioxine.pdf](http://www.tideelbe.de/files/anlage_13_-_sonderkapitel_dioxine.pdf) [48] [↑](#footnote-ref-5)
6. Anonym (2008) Sächsisches Landesamt für Umwelt und Geologie. Dioxindaten 2006-2007, Dresden *(nach Zitat 4)* [↑](#footnote-ref-6)
7. Tabelle 5-3 auf Seite 115 Bewirtschaftungsplan FGG Elbe mit der Überschrift „Schadstoffe mit überregionaler Bedeutung in der Flussgebietseinheit Elbe und Reduzierungsbetrag gegenüber dem Bezugsjahr 2006 bis zur vollständigen Einhaltung der Umweltnormen an den Bilanzmessstellen (Angaben in %) [↑](#footnote-ref-7)
8. In dieser Antwort schrieben Keil et al. [81]: „In 2002/2003, within the Ecological Mega-Project site, extensive analyses of dioxin pollution were conducted. The remediation of the contaminated sites was completed in 2005“. Einen Beleg dafür geben die Autoren nicht und die Referenzen in den unveröffentlichten Studien von LAF und GICON [76-79] enthalten

   keine Hinweise auf derartige Arbeiten im Zeitraum von 2002 bis 2006. [↑](#footnote-ref-8)
9. Büttner O, Böhme M, Rode M (2009) Teilprojekt 3: Hydraulische Modellierung. Abschn 3.8 Überflutung des Salegaster Forsts durch das Hochwasser der Mulde. In: v Tümpling W, Rode M, Böhme M, Gläßer C, Matthies M, Schanze J (Hrsg) SARISK – Entwicklung eines Schadstoffausbreitungsmodells zur stoffbezoge­nen Risikoanalyse und -bewertung extremer Hochwasserereignisse am Beispiel des Landkreises und der Stadt Bitter­feld. Endbericht des Verbundprojektes. BMBF-Förderkennzeichen PTJ 0330690 A-D. Seiten 49-80. <http://www.ufz.de/data/BTFEnd10676.pdf> [↑](#footnote-ref-9)
10. Anonym (2012) Bestandsaufnahme belasteter Altsedimente in ausgewählten Gewässern Sachsen-Anhalts. Phase II. Sedimentbeprobung und Sedimentuntersuchungen. G.E.O.S. Ingenieurgesell­schaft mbH, im Auftrag von: Landesbetrieb für Hochwasserschutz und Wasserwirtschaft Sachsen-Anhalt; Halle, den 30.11.2012; 252 S. [106] [↑](#footnote-ref-10)
11. Wieprecht S et al. (2013) Ermittlung des Remobilisierungspotentials belasteter Altsedimente in ausgewählten Gewässern Sachsen-Anhalts. Institut für Wasser- und Umwelt­systemmodellierung (IWS). Lehrstuhl für Wasserbau und Wasser­mengenwirtschaft der Universität Stuttgart, im Auftrag von: Landesbetrieb für Hochwasserschutz und Wasserwirtschaft Sachsen-Anhalt; Stuttgart, den 11.06.2013; 153 S. [107] [↑](#footnote-ref-11)
12. Diese *Defizite bei der Durchführung von Sanierungsmaßnahmen* erklären sich nur zum Teil durch einen unzureichenden technischen Entwicklungsstand oder finanzielle Restriktionen. Neben dem insgesamt geringeren Problembewusstsein für diese Form von Altlasten könnte die Ursache mangelnder Handlungsbereitschaft darin liegen, dass in Europa für dieses Thema verschiedene *Richtlinien* zuständig sind – Wasserrahmenrichtlinie, Deponierichtlinie, Abfallkatalog – und einzelne Landesbehörden dies zum Anlass nehmen, die anspruchsvolle, von Expertenwissen gprägte Thematik einfach auszublenden [110]. Um derartige Hindernisse bei der Bewältigung eines der vordringlichsten Umweltprobleme, der Sanierung historisch kontaminierter Sedimente, zu überwinden, bedarf es einer *pragmatischen Vorgehensweise*, bei der das Know How von For­schungsinstitutionen, international tätigen Consultingfirmen und wissenschaftlich-technischen Gesellschaften zusammen­geführt wird. [↑](#footnote-ref-12)
13. An der Technologie-Entwicklung im Umfeld der Anwendungen von Aktivkohle, vorzugsweise bei der Stabilisierung von PCDD- und PCB-belasteten Sedimenten sind eine relativ große Zahl von hochrangigen Wissenschaftlern aus den biogeo­chemischen und umwelttechnischen Disziplinen beteiligt (weitere Beispiele [186-190]; dasselbe gilt – z.T. überlappend – für Forscher, die sich bei den jüngsten Fortschritten im Bereich der passiven Beprobungsmethoden (PSM) einen Namen gemacht haben [191-197]. Beide Sets von Zitaten geben dafür Beispiele, jeweils mit *Impact Points in ResearchGate*). [↑](#footnote-ref-13)
14. An der IIASA hatte auch Heinrich Bonnenberg, der später die Altlastenpolitik der Treuhandanstalt maßgeblich bestimmte [2], gearbeitet. Dieses im weiten Sinne für Zukunftsforschung zustänige Institut, das derzeit von 22 Nationalen Mitgliederorganisationen in Afrika, den Amerikas, Asien, Europa und Ozeanienfinanziert wird, war nach der Wende verstärkt mit Fragen der umwelttechnisch-ökonomischen Entwicklung in Mittel- und Osteuropa befasst, bspw. mit der Sanierung und Bewirtschaftung von geschädigten Flusseinzugsgebieten [238]. Man hätte erwarten können, dass gerade die Landesanstalt für Altlastenfreistellung – durch die Forschungsarbeiten im EU-Projekt Welcome [89, 90] und den Wett­bewerblichen Dialog zum Thema „Systemverständnis“ (Kapitel 7.2 und 7.3) – mit dem Konzept der Chemischen Zeit­bombe und damit einem zentralen Aspekt von *Sedimentaltlasten* vertraut gewesen wäre. [↑](#footnote-ref-14)
15. Bei Herrn Dr. Dietfried Donnert, vormals am Institut für Technische Chemie des Forschungszentrums Karlsruhe, seit 10 Jahren internationaler Behörden- und Regierungsberater, bedanke ich mich für die Durchsicht des Manuskripts (UF). [↑](#footnote-ref-15)
16. Numerische Gliederung der Referenzen; *Abschnitt 8.4: herausgehobene Autoren [↑](#footnote-ref-16)
17. A – Z Alphabetische Gliederung (**[1] – [231] fett** = elektronische Verweise siehe numerische Gliederung) [↑](#footnote-ref-17)
